# Quantifying Reductive Amination in Nonenzymatic Amino Acid Synthesis

**DOI:** 10.1002/anie.202212237

**Published:** 2022-10-21

**Authors:** Robert J. Mayer, Joseph Moran

**Affiliations:** ^1^ Institut de Science et d'Ingénierie Supramoléculaires (ISIS) CNRS UMR 7006 Université de Strasbourg 8 Allée Gaspard Monge 67000 Strasbourg France; ^2^ Institut Universitaire de France (IUF) 75005 Paris France

**Keywords:** Amino Acids, Hydride Transfer, Kinetics, Metabolism, Reductive Amination

## Abstract

Amino acid biosynthesis initiates with the reductive amination of α‐ketoglutarate with ammonia to produce glutamate. However, the other α‐keto acids derived from the glyoxylate and Krebs cycles are converted into amino acids by transamination, rather than by reductive amination. Why is only one amino acid synthesized by reductive amination and not the others? To explore this question, we quantified the inherent reactivities of keto acids in nonenzymatic reduction and reductive amination by using BH_3_CN^−^ as a model nucleophile. Biological α‐keto acids were found to show pronounced nonenzymatic reactivity differences for the formation of amino acids (α‐ketoglutarate<oxaloacetate≈pyruvate≪glyoxylate). Accordingly, the flow of ammonia passes through the least reactive α‐keto acid of the Krebs cycle. One possible explanation for this choice is the position of the corresponding amino acid, glutamate, at the top of the thermodynamic landscape for subsequent transamination reactions.

## Graphical abstract

Why does biochemical amino acid synthesis proceed the way it does? Kinetic and mechanistic experiments with a model hydride donor were used to determine the intrinsic electrophilic reactivities of keto acids in reduction and reductive amination reactions. Comparing the nonenzymatic reactivity trends with those found in biology provides new insight into the structure of amino acid metabolism.

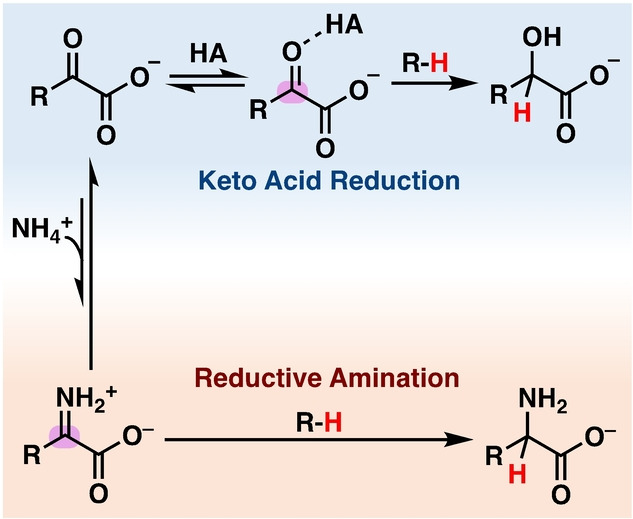

## Introduction

**Figure 1:**
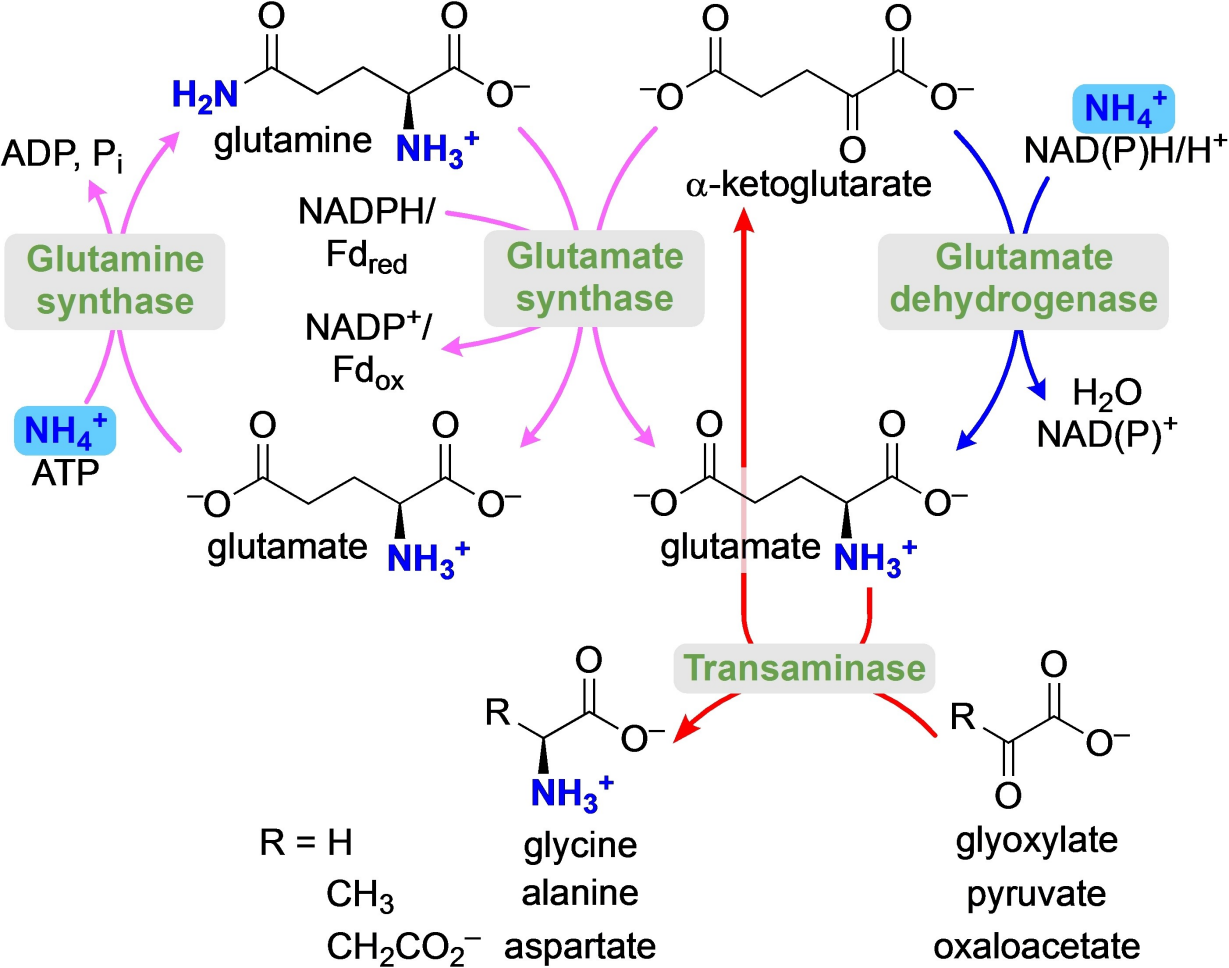
The flow of ammonia in amino acid biosynthesis proceeds through reductive amination for α‐ketoglutarate (blue/purple arrows) but through transamination for pyruvate and oxaloacetate (red arrows).

Biological metabolism exhibits a number of regularities whose origins remain unexplained. Ammonia flows into metabolism during amino acid biosynthesis, but this flow is channeled through certain α‐keto acids and not through others; the reasons for which remain unclear.^[1]^ To be specific, in animal tissues and in some bacteria, glutamate is made by direct reductive amination of α‐ketoglutarate with NH_4_^+^ and NAD(P)H, catalyzed by glutamate dehydrogenase (Figure 1, blue arrows).[^1a^, ^2^] In plants and most bacteria, the NH_4_^+^ for reductive amination of α‐ketoglutarate is indirectly obtained by the hydrolysis of glutamine, catalyzed by glutamate synthase, with the NH_4_^+^ then being channeled through the enzyme to a second active site for reductive amination (Figure 1, purple arrows).[^1a^, ^3^] Glutamine is then regenerated from glutamate and NH_4_^+^ by glutamine synthase, which consumes ATP. However, although other α‐keto acids, such as glyoxylate, pyruvate, or oxaloacetate, could in principle also undergo reductive amination by dehydrogenase enzymes, this typically does not happen.^[4]^ Instead, synthesis of glycine, alanine, and aspartate from the corresponding keto acids proceeds via transamination reactions using glutamate as an amine donor (Figure 1, red arrows),^[1]^ a reaction that can also proceed in the absence of enzymes catalyzed by abiotic catalysts.[^5^, ^6^]

**Scheme 1:**
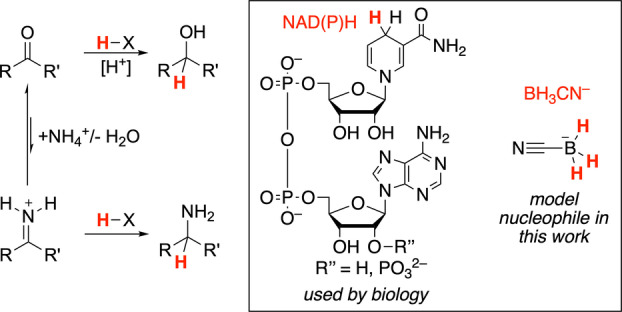
Reduction of ketones or iminium ions (reductive amination) and reducing agents.

From an evolutionary standpoint, it is not clear why the biochemical selectivity for the reductive amination of α‐ketoglutarate over pyruvate or oxaloacetate emerged. One hypothesis is that underlying nonenzymatic reactivity trends were preserved and amplified by enzymes. However, the underlying reactivity trends for reductive amination of α‐keto acids have not yet been studied. In this work, we thus set out to test whether the preference for a glutamate‐based amino acid synthesis in biology might be derived from inherent selectivity in nonenzymatic reductive amination reactions (Scheme 1).

So far, nonenzymatic reductive aminations have been reported under homogeneous^[7]^ or heterogeneous catalysis,^[8]^ photochemistry,^[9]^ electrochemistry,^[10]^ oxidative decarboxylation,^[11]^ and with nucleophilic hydride species.[^12^, ^13^] While NADH or dihydronicotinamides would be ideal model nucleophiles to investigate the reactivity of keto acids under nonenzymatic conditions, initial experiments showed this to be unfeasible.^[14]^ Outside of enzymes, NADH and dihydronicotinamides do not undergo hydride transfer toward keto acids which can at least in part be attributed to their hydrolysis even under the mildly acidic conditions which are required to activate carbonyl compounds.^[15]^ Additionally, the nonenzymatic reactivity of dihydronicotinamides toward carbonyl groups in water is low and only a few reports exist on their reactions with highly electrophilic species.^[16]^

To circumvent these issues, we envisioned to apply BH_3_CN^−^ as a model nucleophile to quantify the relative reactivity of keto acids and the related iminium ions in hydride transfer reactions, as BH_3_CN^−^ is hydrolytically stable even at acidic pH and known to be a suitable nucleophile for both reduction and reductive amination reactions in water.[^13^, ^17^, ^18^] Previous studies of nucleophile‐electrophile recombinations have shown that relative electrophilic reactivity is independent of the nucleophile.^[19]^ While this assumption has been verified most extensively for carbenium ions and acceptor‐substituted olefins, it is not generally established for nucleophile additions to carbonyl groups in water which might be subject to general catalysis. However, we found that the relative electrophilicities of various ketones determined toward carbanions in DMSO follow the same trends as observed both in their reduction rates with BH_4_^−^ in dioxane/water and in their rates of hydration (Figure S1).^[20]^ Furthermore, linear relationships of the rate constants of nucleophile additions in water had previously been reported for different ketones, additionally suggesting that the relative electrophilicities are independent of the nucleophile also for ketones.^[20d]^ Thus, we assume that relative electrophilicities of keto acids and iminium ions observed with BH_3_CN^−^ should also be somehow reflective of unobstructed nonenzymatic hydride transfer with other nucleophilic hydride species like NAD(P)H.

Using BH_3_CN^−^ as model nucleophile, we thus set out to investigate the mechanism and to quantify the reactivity trends of the direct reduction of α‐keto acids by means of kinetic measurements and competition experiments as a function of pH and buffer composition. Next, we studied the underlying equilibria of imine and iminium ion formation from keto acids and quantified their reactivity within reductive amination. Lastly, we compared the relative reactivities of all investigated species to test whether fundamental differences in reactivity can explain the biochemical preference for the reductive amination of α‐ketoglutarate.

## Results and Discussion

### Reduction of α‐Keto Acids

**Scheme 2:**
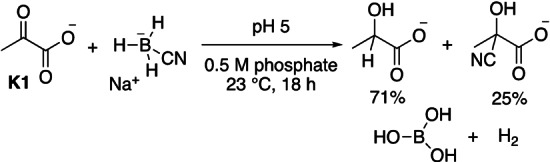
Products of the reaction of **K1** with NaBH_3_CN. See the Supporting Information p. S5 for the spectroscopic analysis.

Initially, the products of the reaction of pyruvate with NaBH_3_CN were studied. NMR spectroscopic analysis of the reaction of pyruvate (**K1**) with stoichiometric NaBH_3_CN in aqueous phosphate solution (0.5 M, pH 5) showed the formation of 71 % lactate, hydrogen gas, boric acid, 25 % of cyanohydrin and 4 % unreacted **K1** after a reaction time of 18 h (Scheme 2).[^21^, ^22^, ^23^] The formation of cyanohydrin proved to be reversible and consequently the lactate concentration was found to slowly increase over days. At pH 4, however, cyanohydrin formation with **K1** was found to be negligibly small and, accordingly, pH 4 was chosen as pH value for our subsequent kinetic studies.

**Figure 2:**
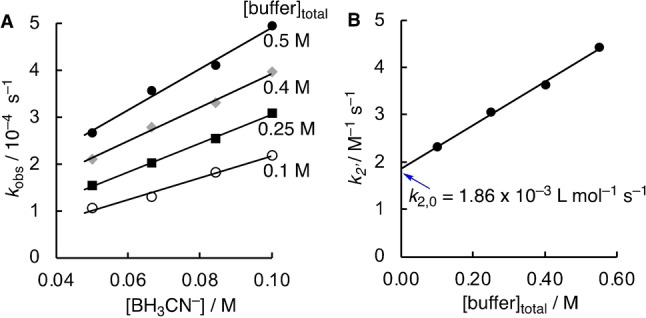
A) Correlations of *k*_obs_ for the reaction of pyruvate (**K1**, 5.0 mM) with [BH_3_CN^−^] for different concentrations of acetate buffer at pH 4, 20 °C, *I*=1.0 (NaCl) used to determine *k*_2′_. B) Correlation of *k*_2′_ with the buffer concentration yields *k*_2,0_ as intercept with the ordinate.

The kinetics of the reaction of pyruvate with BH_3_CN^−^ were followed by using UV/Vis spectroscopy to measure the disappearance of the carbonyl absorption band of **K1** in acetate buffer at pH 4. By using a large excess of BH_3_CN^−^ over **K1**, pseudo‐first order kinetics resulted which allowed to determine the rates *k*_obs_. Correlation of *k*_obs_ with the concentration of [BH_3_CN^−^] afforded apparent second‐order rate constants *k*_2′_ (Figure 2A). The rate constants *k*_2′_ are additionally dependent on the concentration of the buffer, implying that the reaction is subject to general acid‐base catalysis (Figure 2B).^[24]^ To allow a consistent comparison of the reduction rates of various keto acids with BH_3_CN^−^, the correlation of *k*_2′_ with the buffer concentration was finally used to determine the buffer‐independent second‐order rate constants *k*_2,0_ from the intersection with the ordinate.

**Figure 3:**
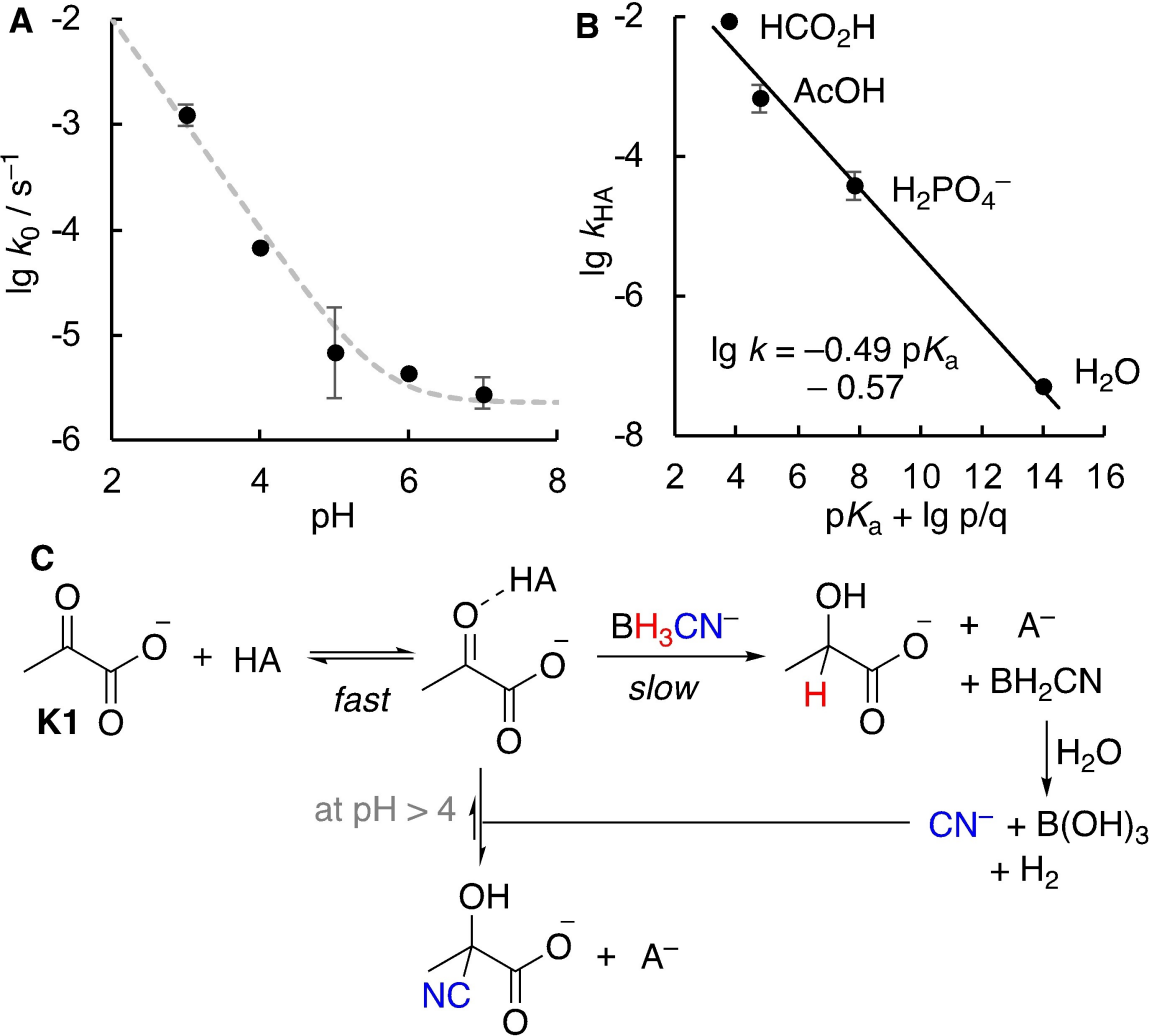
A) pH‐rate profile for the reaction of **K1** (5 mM) with NaBH_3_CN (50 mM) from buffer independent rates *k*_0_. The dashed line corresponds to the fit to the equation *k*_0_=*k*_w_+*k*_H+_[H^+^] with *k*_w_=(2.73±0.85)×10^−6^ s^−1^, *k*_H+_=0.78±0.19 M^−1^ s^−1^. B) Brønsted plot of lg *k*_HA_ versus the statistically corrected p*K*_a_ values. C) Proposed mechanism for the reaction of BH_3_CN^−^ with **K1**.

Next, we compared the reactivity of the reduction reaction of different keto acids based on the model reaction with BH_3_CN^−^ in an analogous way as shown for pyruvate (**K1**). Table 1 lists the buffer‐independent second order rate constants *k*_2,0_ for the reaction of BH_3_CN^−^ with the different keto acids **K1**–**K4** at pH 4. To identify the reasons for the observed reactivity differences of **K1**–**K4** and to verify how they hold under different pH and buffer concentrations, initially the pH‐rate profile for the reaction of **K1** with BH_3_CN^−^ was studied (Figure 3A).^[25]^ The observed pH‐dependency could be analyzed by the empirical equation *k*_0_=*k*_w_+*k*_H+_[H^+^], where an acid‐dependent reaction (via *k*_H+_) dominates at lower pH and is characterized by a slope of −1, while a “water‐catalyzed” background reaction (via *k*_w_) takes over at neutral pH. Lastly, we correlated the rate constants *k*_HA_ for catalysis by the protonated component of the buffer with the corresponding p*K*_a_ values (Figure 3B). The slope of the resulting Brønsted‐plot of α=−0.49 suggests the involvement of a proton‐transfer during the transition state of the reaction.

**Table 1:** Buffer‐independent second‐order rate constants *k*_2,0_ for the reaction of **K1**–**K4** with NaBH_3_CN at 20 °C, pH 4 and *I*=1.0 (NaCl).

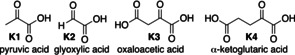		
Electrophile	*k*_2,0_ [L mol^−1^ s^−1^]	*k*_2, rel._^[a]^
**K1**	(1.86±0.07)×10^−3^	1
**K2** ^[b]^	(5.57±0.16)×10^−3^	3.0
**K3**	(1.58±0.07)×10^−1^	85
**K4**	(3.93±0.10)×10^−2^	21

[a] Calculated relative to the rate with **K1**. [b] Mostly present in the form of the hydrate in aqueous solution.

Both the buffer and pH‐dependency as well as the slope observed in the Brønsted plot support a mechanism like the one depicted in Figure 3C that relies on general acid catalysis, in contrast to one proceeding exclusively via specific catalysis. Accordingly, the reaction rate is composed of different terms that will have a different contribution depending on the pH as well as the presence or absence of a buffer species HA [Eq. 1]. 

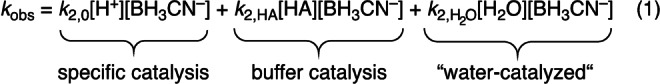




In 0.5 M acetate buffer at pH 4, catalysis by the buffer accounts for 58 % of the reaction rate with the remaining contribution being mostly due to specific catalysis (cf. Figure 2B). In turn, at pH 5 more then 96 % of the reaction rate is due to the buffer‐catalyzed reaction. At neutral pH, specific catalysis is insignificant and the reaction will mostly be due to buffer catalysis and a background reaction enabled by water. General acid catalysis was previously reported for the addition step of sufficiently strong nucleophiles to carbonyls compounds.^[26]^ Furthermore, the hydride transfer of an *N*‐benzylated dihydronicotinamide to hexachloroacetone in ethanol was proposed to be subject to general acid catalysis.^[27]^

**Figure 4:**
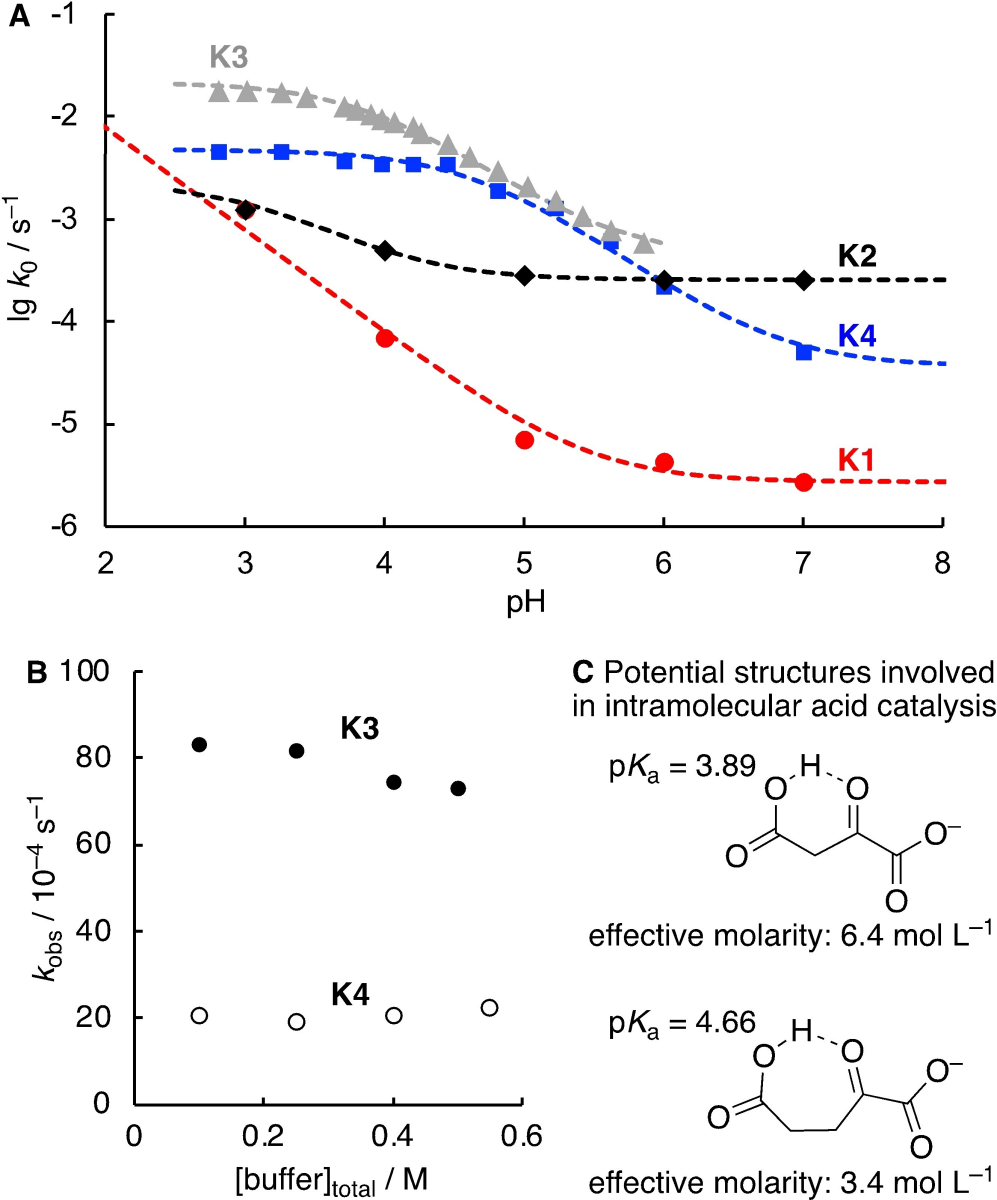
A) pH‐rate profiles for the reaction of **K1**–**K4** with NaBH_3_CN (50 mM). The dashed lines correspond to the fitted equation for *k*_0_ (see the Supporting Information). B) Dependence of *k*_obs_ for the reaction of **K3** and **K4** (5 mM) with NaBH_3_CN (50 mM) on the total concentration of acetate buffer at pH 4, 20 °C and *I*=1.0 (NaCl). C) Intramolecular acid catalysis and effective molarities.

Based on the insights on the mechanism of the reduction of **K1**, we next studied the pH‐rate profiles as well as the buffer dependency of the reactions with the other keto acids **K2**‐**4**. At pH 4, glyoxylate (**K2**) was found to be only slightly more electrophilic than **K1** and the reduction reaction is analogously subject to general catalysis (see Supporting Information, p. S19). However, the reactivity trends change drastically with decreasing acidity and at neutral pH the reduction of **K2** is two orders of magnitude faster than that of **K1** (Figure 4A). The significantly higher rate of the water‐catalyzed reaction at pH 7 of **K2** compared to **K1** is not surprising given the intrinsic higher reactivity of aldehydes compared to ketones.^[20a]^ In aqueous solution, glyoxylate is almost predominantly present as a hydrate and it is the small amount of free aldehyde which is reacting.^[28]^ To exclude that the dehydration reaction of glyoxylate hydrate is rate‐determining under our reaction conditions and thus, a main reason for the pH‐rate behavior, we determined the rate of dehydration by exchange NMR spectroscopy (see the Supporting Information p. S33). In support of our kinetic analysis, we found that at pH 4 and 7 the rate of dehydration is faster than that of the reduction reaction.

Comparison of the buffer‐independent second‐order rate constants *k*_2,0_ indicate that at pH 4 oxaloacetate (**K3**) and α‐ketoglutarate (**K4**) react approximately 85 and 21 times faster than **K1** (Table 1). In contrast to **K1** and **K2**, the rates *k*_obs_ for the reduction of both **K3** and **K4** were found to be independent of the concentration of the buffer (Figure 4B). The pH‐rate profiles show titration of the terminal carboxylate of **K3** and **K4** at their respective p*K*_aH_ values but are otherwise consistent with an acid‐dependent reaction (Figure 4A, grey and red points). Accordingly, we attribute the significantly higher reduction rates of **K3** and **K4** compared to **K1** observed at acidic pH to the terminal carboxylate groups of **K3** and **K4** being involved in intramolecular acid catalysis (Figure 4C).^[29]^ If intramolecular acid catalysis is a significant contribution to the reaction, then the loss of intermolecular general acid catalysis would be predicted and *k*_obs_ should not increase with the buffer concentration, conforming to our experimental observations. Intramolecular acid catalysis might now also be used to explain the relative reactivities of **K3** and **K4** at acidic pH which is also illustrated by the differences in the effective molarities (Figure 4C, Supporting Information p. S27/30). An intramolecular hydrogen‐bond forms a six‐membered ring in **K3**, but a similar hydrogen bond in **K4** forms a less favorable seven‐membered ring (Figure 4C).

When comparing the buffer‐independent pH‐rate profiles of all four keto acids (Figure 4A), it becomes clear that the reactivity ordering of **K1**–**K4** is largely dependent on the pH value. These trends will additionally change with buffer concentration, as the reduction rate of **K3** and **K4** is independent on the buffer, while it is the opposite for **K1** and **K2**.

**Figure 5:**
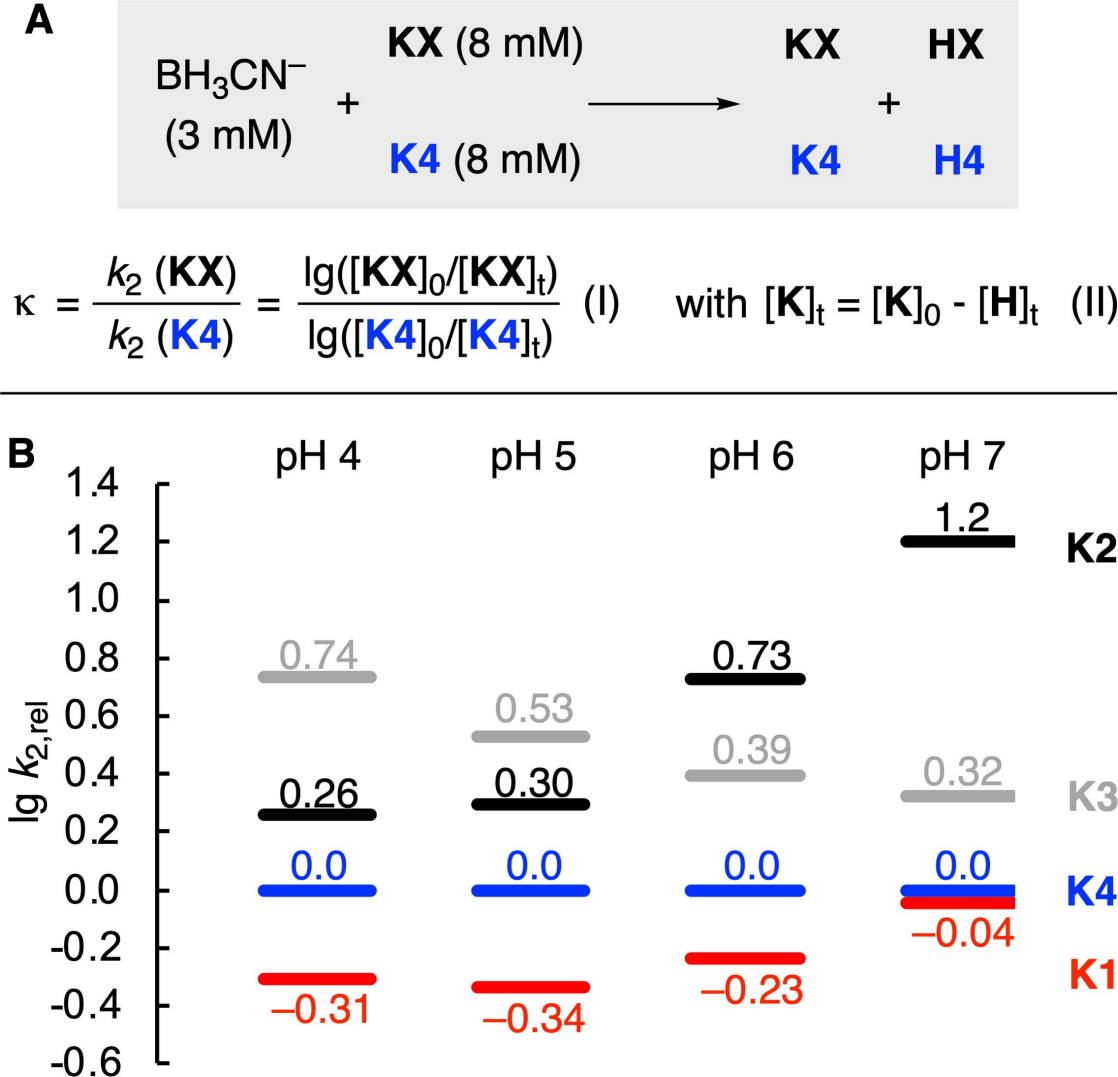
A) Competition experiments to analyze the relative rates of keto acid reduction. B) Rates lg *k*_2,rel_ for the keto acid reduction relative to the reduction of **K4** based on pairwise competition experiments of **K1**, **K2** and **K3** with **K4** in 0.5 M acetate or phosphate buffer at pH 4–7 (to aid the comparison with Figure 4A, the same colors were used for the different keto acids).

To further verify how the reactivity trends change with pH in the presence of buffer, competition experiments were performed. Additionally, these experiments allow to verify the trends obtained in our kinetic analysis. The readily occurring aldol addition reactions between keto acids **K1**‐**K3** renders a direct competition experiment between all four keto acids impossible.^[30]^ Instead, we performed pairwise competition experiments of a limiting amount of BH_3_CN^−^ reacting with **K4** and **K1**–**3** (Figure 5A). Analysis of the ratio of keto acids before and after the reaction ([**K**]_0_/[**K**]_t_) for both competing electrophiles allows to calculate the competition constants κ which corresponds to the ratio of the second order rate constants [Figure 5A, I].^[31]^ Due to significant overlap of the keto acid resonances in the NMR spectra after the reaction, the concentration [**K**]_t_ was derived from the concentration of the hydroxy acid [**H**]_t_ [Figure 5A, II]. In this case, this treatment allows to subtract the contribution of side reactions (e.g. cyanohydrin formation) and decomposition of the keto acids (e.g. decarboxylation of oxaloacetate). Based on the competition constants for all three pairwise reactions, the relative reactivities of all species was assigned (Figure 5B).

Comparing the relative rates of all keto acids with another in the presence of 0.5 M buffer shows that the absolute differences in reactivities are far smaller than in the buffer‐independent reaction (cf. Figure 4A). This can be explained by the large buffer dependency of the reduction rates of **K1** and **K2** while the reduction of **K3** and **K4** is insensitive to buffer effects (cf. Figure 4B). As a result, at neutral pH the reduction of glyoxylate (**K2**) is kinetically dominating despite being unfavored at acidic pH and in the absence of a buffer.

### Equilibria for Imine/Iminium Ion Formation in Solution

**Scheme 3:**
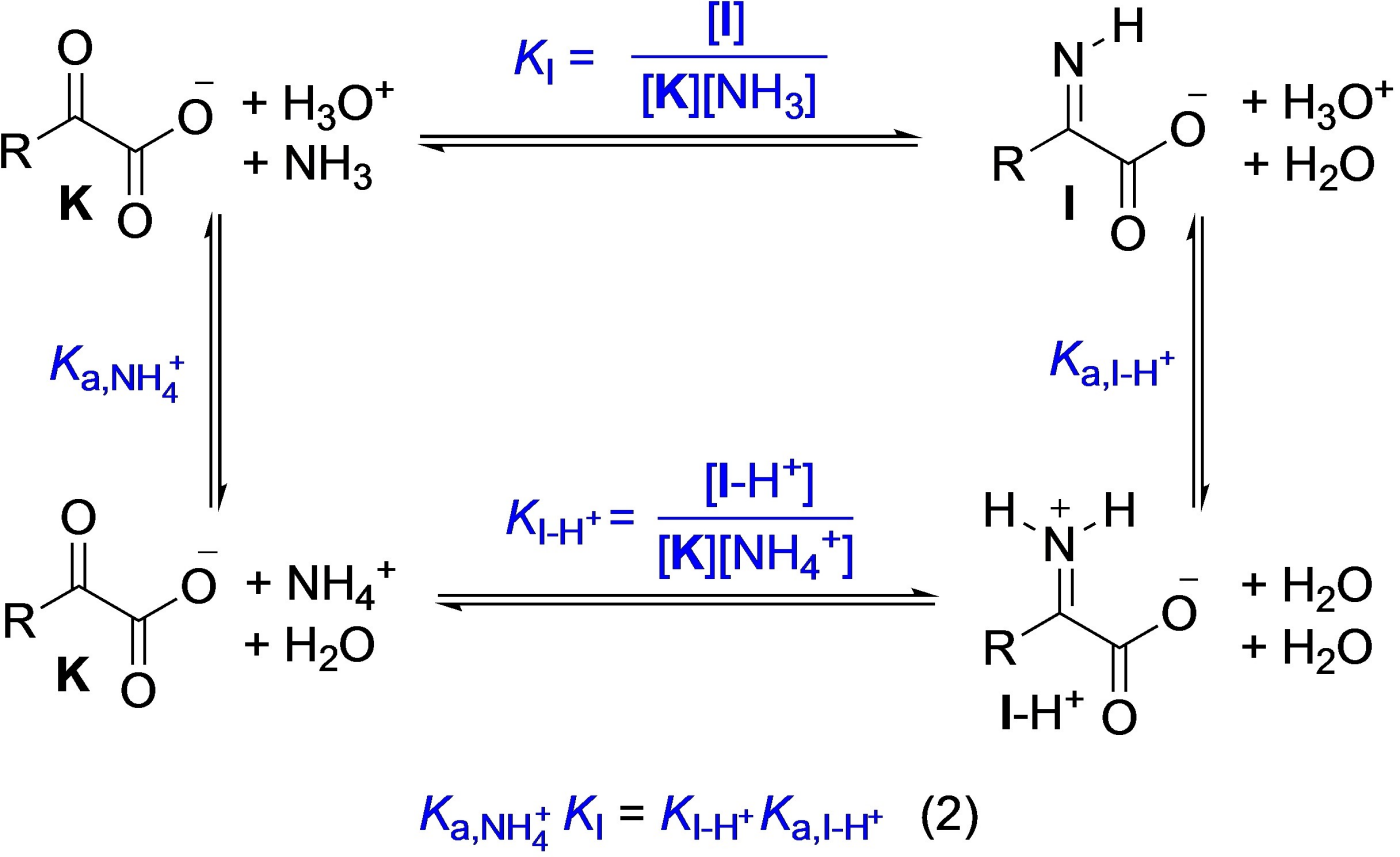
Equilibria for imine/iminium formation from α‐keto acids and NH_3_/NH_4_^+^.

The equilibrium for the formation of imine **I** or iminium ion **I**‐H^+^ from keto acids and ammonia can be described by the reactions in Scheme 3. However, quantitative insight into imine or iminium formation from α‐keto acids and ammonia is scarce: The equilibrium constant *K*_I_ for imine formation in the reaction of pyruvate with ammonia has been reported by using polarographic measurements in aqueous solution at pH 9.25 and was found to be *K*=0.25 at 0 °C.[^32^, ^33^]

As the equilibrium constant *K*_I_ for imine formation from α‐ketoglutarate was not yet reported, we attempted to determine it, as well as that for pyruvate, by ^1^H NMR titrations at buffered alkaline pH and at constant ionic strength (*I=*1.5). As the iminium ions **I**‐H^+^ are acidic (p*K*_a_≈5–9), titrations at pH=10.2 will predominantly reflect the formation of the imine **I**. Analysis of the NMR shift of the CH_3_ resonance of pyruvate and the β‐CH_2_ resonance of α‐ketoglutarate as a function of the NH_3_ concentration gave rise to shallow binding isotherms (see the Supporting Information on p. S42 for a detailed discussion including IR spectroscopy and DFT computations). Analysis of the binding isotherms at 23 °C provided the equilibrium constants for imine formation for both pyruvate (*K*=0.104±0.008) and α‐ketoglutarate (*K*=0.060±0.025), the former of which is in reasonable agreement with the value reported for pyruvate at 0 °C from polarography (*K*=0.25).[^32^, ^34^]

To determine the equilibrium constants *K*_**I**‐H+_ for iminium ion (**I**‐H^+^) formation, which is pertinent in neutral or acidic solution, the thermodynamic cycle described in Scheme 3 and Equation (2) was used. As the acidity of NH_4_^+^ (*K*_a,NH4+_) is known, the knowledge of the acidity of **I**‐H^+^ (*K*_a,I‐H+_) allows to calculate *K*_I‐H+_. However, due to their instability in aqueous solution, the acidity of iminium ions cannot be determined experimentally in a straightforward way. To solve this issue, we turned to DFT computations anchored to a set of reference compounds (see the Supporting Information, Tables S18/19) which allowed us to assign an acidity of p*K*_a_=7.8±0.3 to the iminium ion of pyruvate (for other iminium ions, see Table 3).

**Figure 6:**
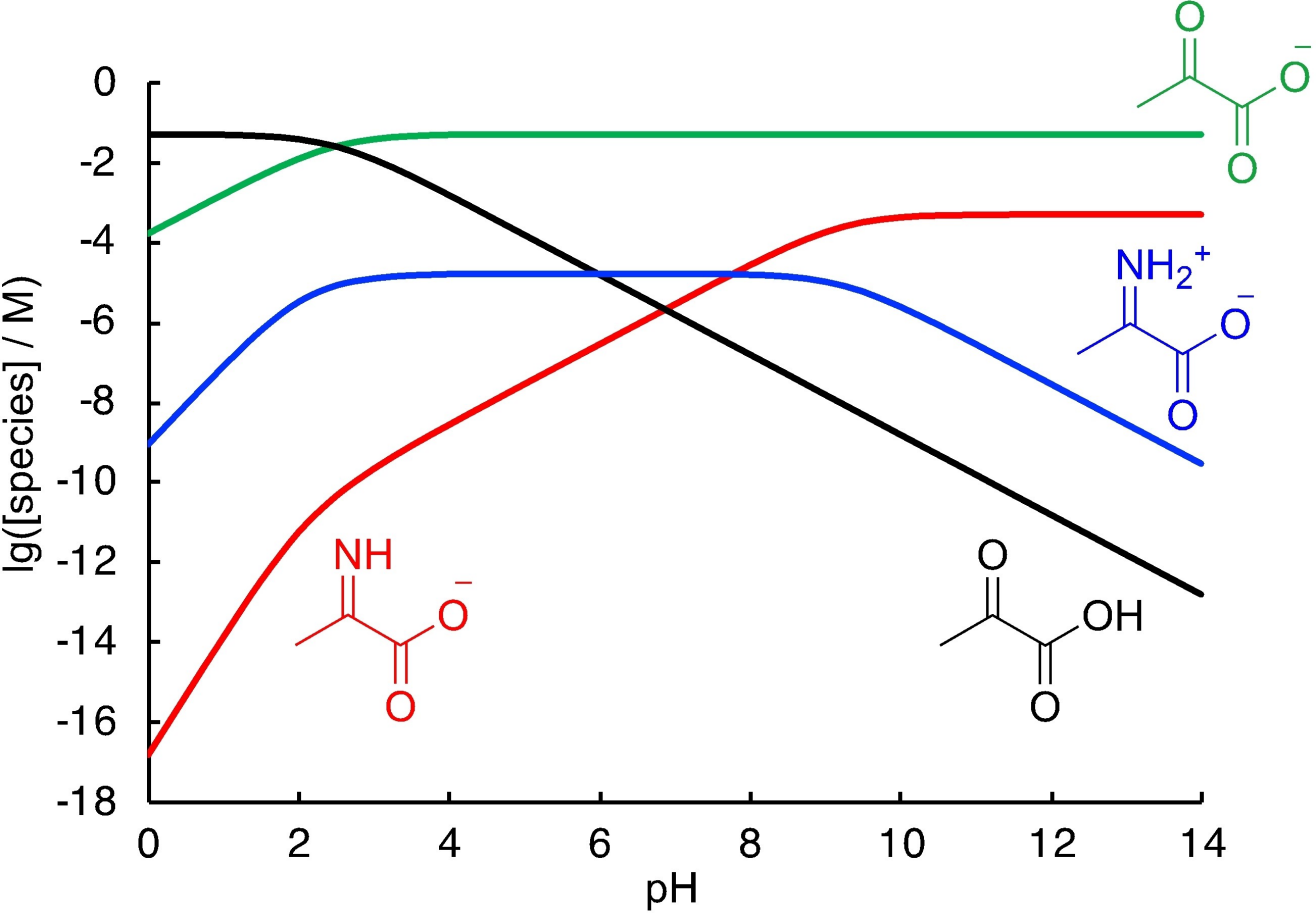
Species distribution for the system pyruvate (50 mM)/NH_3_ (100 mM) as a function of pH with *K*_I_=0.104, p*K*_a,NH4+_=9.25, p*K*_a,**I1**‐H+_=7.78 and p*K*_a,**K1**_=2.49.

From the equilibria in Scheme 3, the pH‐dependent species distribution was calculated as shown in Figure 6 on the example of pyruvate (for the analogous analysis of α‐ketoglutarate, see the Supporting Information Figure S22). Comparing the relative amounts of imine and iminium ion as a function of pH, the iminium ion dominates between pH 4 and 8, where its concentration stays almost constant. Accordingly, general catalysis, as observed for the reduction of keto acids, does not play a significant role below pH 8, as the imine is fully protonated.

### Kinetics of Reductive Amination

**Figure 7:**
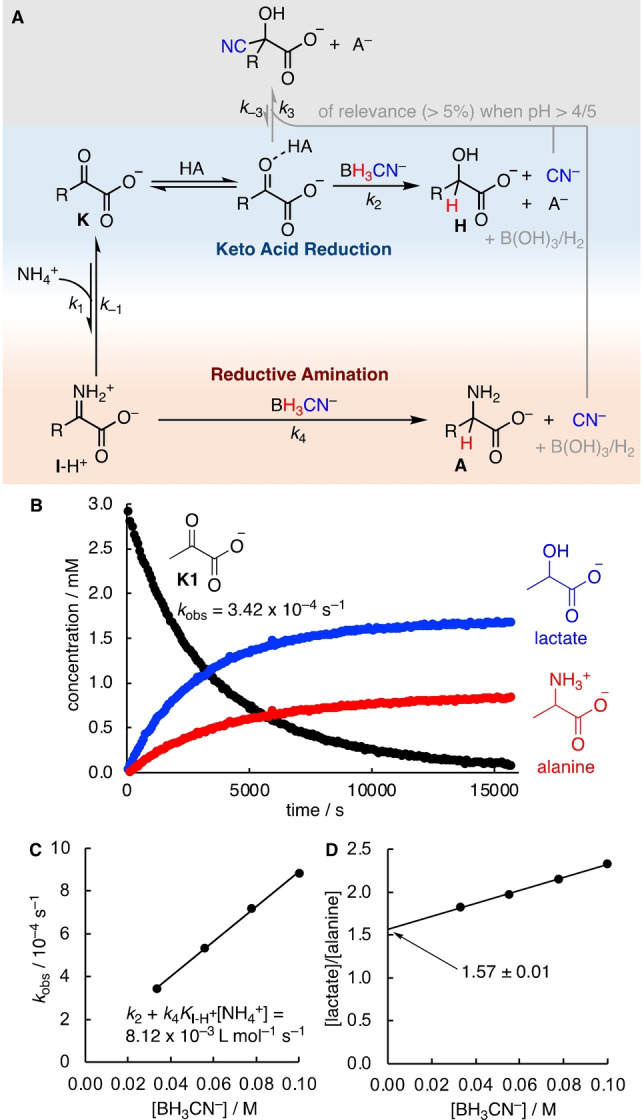
A) Mechanistic model for reductive amination. B) ^1^H NMR kinetics of the reaction of pyruvate (**K1**, 3.3 mM) with NaBH_3_CN (33.3 mM) and NH_4_Cl (0.5 M) in 0.5 M phosphate solution, pH 4 at 20 °C. C) Correlation of *k*_obs_ and [BH_3_CN^−^] with [NH_4_Cl]=0.5 M at pH 4. D) Correlation of the experimental ratio [lactate]/[alanine] at pH 4 from quantitative ^1^H NMR spectroscopy vs. [BH_3_CN^−^].

Treating an α‐keto acid with a reductant in the presence of NH_4_^+^ results in competing reduction of the keto acid and the iminium ion formed in equilibrium (Figure 7A). When using BH_3_CN^−^ as a reductant, cyanide is formed as by‐product along with the hydroxy and amino acids. Cyanohydrin formation thus becomes a third potentially competing pathway, as already discussed above for the reduction of keto acids. However, for **K1** and **K2**, cyanohydrin formation was found to be negligible at pH 4, while, for **K3** and **K4**, only traces could be detected at pH 5. To study the kinetics of the reductive amination of pyruvate (**K1**) with NaBH_3_CN in the presence of NH_4_Cl, measurements were therefore performed at pH 4 (Figure 7). To reduce buffer‐catalysis of the keto acid reduction, we additionally used phosphate solution instead of acetate buffer, as under these conditions the H_2_PO_4_^−^ ion is a weaker acid catalyst.

In the following, we briefly describe our analysis of the kinetics of reductive amination; a more detailed explanation with full derivations is provided in the Supporting Information. For the mechanistic model of Figure 7A, under conditions where cyanohydrin formation is negligible, the disappearance of the keto acid **K** is described by Equation (3), which consists of two terms.

On one hand, **K** undergoes direct reduction through acid‐catalyzed reaction with BH_3_CN^−^, as described by the rate constant *k*_2_ (cf. the section on keto acid reduction above). On the other hand, **K** is in equilibrium with the iminium ion **I**‐H^+^ which is reduced by BH_3_CN^−^ via the rate constant *k*_4_. Combining the definition of the equilibrium for iminium formation from **K** and NH_4_^+^ [Eq. (4)] with the rate law for disappearance of **K** [Eq. (3)] results in Equation (5). 

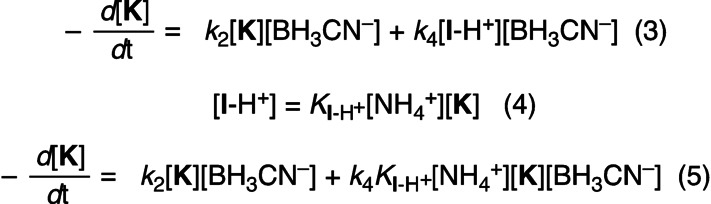




Equation (5) can be simplified under pseudo‐first order conditions when there is a large excess of BH_3_CN^−^ and NH_4_^+^ and, accordingly, the concentrations of BH_3_CN^−^ and NH_4_^+^ will not change largely during the reaction. With the definition of the pseudo‐first order rate constants *k*_2ψ_ [Eq. (6)] and *k*_4ψ_ [Eq. (7)], Equation (5) thus simplifies to Equation (8).

A rate law like Equation (8) can be solved analytically for the disappearance of **K** [Eq. (9)] as well as for the formation of the products **H** and **A**.^[35]^ Thus, *k*_obs_ for the disappearance of **K1** in Figure 7B corresponds to the sum *k*_2ψ_+*k*_4ψ_ and the product ratios of **H** and **A** can be used to differentiate the terms *k*_2ψ_ and *k*_4ψ_.

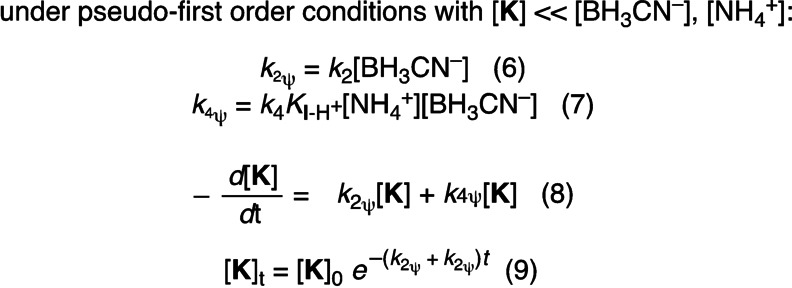




In a series of measurements with constant concentration of NH_4_^+^ but with variation of the excess of BH_3_CN^−^, a correlation of *k*_obs_ (=*k*_2ψ_+*k*_4ψ_) vs [BH_3_CN^−^] should have a slope of *k*_2_+*k*_4_ *K*_I‐H+_[NH_4_^+^]. These two terms relate to the sum in Equation (5): The first term describes the contribution of the general acid catalyzed and pH‐dependent keto acid reduction. The second term accounts for the reductive amination, the rate of which should not change largely between pH 4 and 8 due to the constant concentration of the iminium ion (cf. Figure 6). Indeed, experimental analysis of the reductive amination of pyruvate (**K1**) showed the expected linear correlation (Figure 7C).

In a series of measurements with different excesses of BH_3_CN^−^, the product ratio of hydroxy to amino acid was used to differentiate the two terms of *k*_obs_=*k*_2ψ_+*k*_4ψ_, which in turn also differentiates the sum in Equation (5) into *k*_2_ and *k*_4_ *K*_I‐H+_[NH_4_^+^]. At pH 7, the product ratio of hydroxy to amino acid was found to be independent of [BH_3_CN^−^] in line with the mechanism in Figure 7A. At pH 4, a slight dependency on [BH_3_CN^−^] was observed and the lactate/alanine ratio at [BH_3_CN^−^]=100 mM was 27 % higher than at [BH_3_CN^−^]=33 mM (Figure 7D). We attribute this discrepancy to the rate of iminium formation (via *k*_1_) that becomes slower due to the decreased amount of free NH_3_ present at pH 4. To ascertain our analysis, we extrapolated the product ratio of hydroxy and amino acid at [BH_3_CN^−^]=0 from the linear correlation of the product ratios and [BH_3_CN^−^] (Figure 7D). The product ratio at [BH_3_CN^−^]=0 corresponds to reaction conditions under which the reduction step is clearly rate limiting, as the reaction is so slow that full equilibration of **K** and **I**‐H^+^ is achieved. This product ratio was finally used to separate the terms in Equation (5). A more detailed explanation of our approach and a verification with numerical simulations is shown in the Supporting Information on page S48–50.

Table 2 summarizes the values of *k*_2_ and *k*_4_ *K*_I‐H+_[NH_4_^+^] as well as the associated errors determined in this way for the reductive amination of pyruvate (**K1**). For the other keto acids **K2**‐**K4**, the measurements were performed in an analogous way as described for **K1**, using the same concentration of NH_4_Cl. However, the reductive aminations of **K3** and **K4** were studied at pH 5, as, at pH 4, the kinetics were found to result almost exclusively from keto acid reduction. The product *k*_4_ *K*_I‐H+_ allows us to rank the relative reactivities of keto acids **K1**–**4** in the reductive amination under an identical NH_4_^+^ excess. The formation of aspartate and alanine is faster by factors of 1.7 and 1.9, respectively, than the formation of glutamate. The rate for reductive amination of **K2** is 11 times larger than for **K1**.

**Table 2:** Second‐order rate constants *k*_2_ and *k*_4_ *K*_I‐H+_ for hydroxy (**H**) and amino acid (**A**) formation in the reaction of **K1**–**4** with NaBH_3_CN in the presence of NH_4_Cl (0.5 M) determined by ^1^H NMR spectroscopy at 20 °C in 0.5 M phosphate solution.^[a]^

Species	*k*_2_+*k*_4_ *K*_I‐H+_[NH_4_^+^]	[**H**]/[**A**]	*k*_2_ [L mol^−1^ s^−1^]	*k*_4_ *K*_I‐H+_[NH_4_^+^] [L mol^−1^ s^−1^]	p*K*_a,I‐H+_^[b]^	*K*_I_	*K*_I‐H+_^[e]^
**K1**	(8.12±0.21)×10^−3^	1.57±0.01	(5.0±0.1)×10^−3^ (pH 4)	(3.2±0.1)×10^−3^	7.8±0.3	0.104±0.008 [c]	(4±3)×10^−3^
**K2**	(3.96±0.26)×10^−2^	1.12±0.02	(2.1±0.1)×10^−2^ (pH 4)	(1.9±0.1)×10^−2^	5.3±0.3	[d]	
**K3**	(1.94±0.06)×10^−2^	5.78±0.27	(1.7±0.1)×10^−2^ (pH 5)	(2.9±0.1)×10^−3^	8.9±0.4	[d]	
**K4**	(8.66±0.30)×10^−3^	4.24±0.30	(7.0±0.3)×10^−3^ (pH 5)	(1.7±0.1)×10^−3^	8.7±0.4	0.061±0.025 [c]	(2±2)×10^−2^

[a] All errors correspond to standard errors. For values calculated from multiple entries, the errors were calculated from propagation of the individual errors. [b] Extrapolated from experimentally anchored DFT computations (see the Supporting Information). [c] Determined by ^1^H NMR titrations (this work). [d] Could not be determined.^[34]^ [e] Calculated as *K*_I‐H+_=*K*_a,NH4+_ *K*_I_/*K*_a,I‐H+_.

### Competition Experiments of Reductive Amination

**Figure 8:**
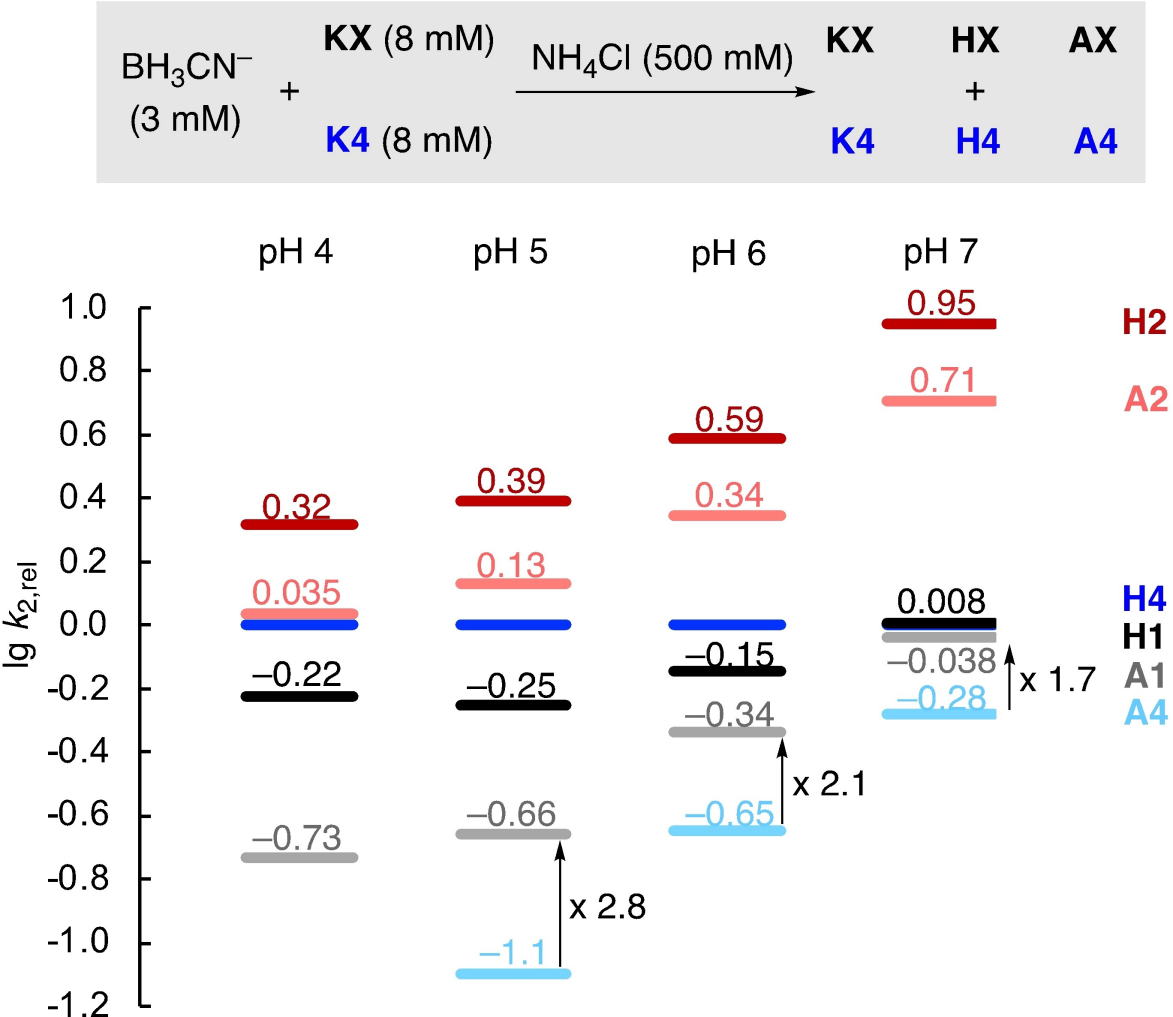
Rates lg *k*_2,rel_ for keto acid reduction and reductive amination relative to the reduction of **K4** based on pairwise competition experiments of **K1**, **K2** and **K3** with **K4** in 0.5 M acetate (pH 4/5) or phosphate buffer (pH 6/7).

The pH‐dependent species distribution suggests that the amount of iminium ion in solution stays stationary for pyruvate and α‐ketoglutarate within pH 4 and 7 (Figure 6, Figure S22). Accordingly, the reactivity ordering of the reductive amination reaction should stay constant within this pH range. As direct kinetic studies cannot be used above pH 4–5 due to experimental limitations, we turned to competition experiments to verify our model. Binary mixtures of keto acids were subjected to conditions of reductive amination and the product distribution was analyzed to determine the competition constants for the reaction to both hydroxy acid **H** and amino acids **A** under these conditions (Figure 8, see the Supporting Information for full details).[^31^, ^36^]

Figure 8 shows that increasing the pH enhances the rate of reductive amination with respect to keto acid reduction for alanine (**A1**) and glutamate (**A4**) while the ratio stays almost constant in the case of glycine (**A2**). However, even for **A1** and **A4**, the ratio of amino vs. hydroxy acids is surprisingly low despite the large excess of NH_4_^+^, even at neutral pH. This contrasts with the high selectivity typically observed in reductive amination reactions with classical organic substrates.^[13]^ Accordingly, these results suggest that the reactivity difference of keto acids and their respective iminium ions is significantly smaller compared to that found with aliphatic ketones. Comparing the rate constants of the keto acids to undergo reductive amination confirms the intrinsically lower tendency to form glutamate (**A4**) compared to alanine (**A1**) also observed in the direct kinetic experiments (Table 2). When increasing the pH from 5 to 7, the ratio of the rate constants for the formation of glutamate vs. alanine is slightly reduced from 2.8 to 1.7. However, the changes in the relative reductive amination rates are far smaller than those observed for the keto acid reduction (cf. Figure 4A), which is in line with the relatively stationary concentration of iminium ions in the investigated pH range. In line with direct kinetic experiments, the high reactivity of glyoxylate (**K2**) compared with other keto acids at neutral pH to yield either glycine (**A2**) or glycolate (**H1**) is also found in competition experiments.

Lastly, competition experiments were used to study the buffer dependency of the reductive amination reaction in a mixture of **K1** and **K4** (Supporting Information, p. S61). When performing the reaction in 50 mM and 500 mM buffer, the observed changes were rather small and neither the ratio of glutamate (**A4**) to alanine (**A1**) nor the rates changed significantly.

### Thermochemistry of Reductive Amination

Experimental thermochemistry for reductive amination has previously been derived from kinetic measurements of the enzyme catalyzed reaction of NAD^+^ with glutamate or alanine.^[37]^ The Gibbs free energies of these reactions were combined by means of thermodynamic cycles with the known energetics of transamination to provide the Gibbs energies of reductive aminations for further keto acids (Table 3). The trends in thermodynamics of reductive amination (with NADH) are globally in line with the kinetics observed for the nonenzymatic reduction with BH_3_CN^−^: The fastest reductive amination is observed for the most exergonic substrate (glyoxylate/glycine), while the reaction is slowest (and least exergonic) for glutamate synthesis. However, the reductive amination of oxaloacetate proceeds slower (based on *k*_4_ *K*_**I‐H+**_) than expected from thermochemistry.

**Table 3:** Experimental Gibbs energies of reductive amination at 25 °C.

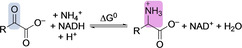	
R=	Δ*G* ^0^ [kJ mol^−1^]
H (glycine)	−89.73^[a]^
CH_3_ (alanine)	−75.15±0.80^[b]^
CH_2_COO^−^ (aspartate)	−79.53^[a]^
CH_2_CH_2_COO^−^ (glutamate)	−74.80±0.80^[c]^

[a] Calculated from a thermodynamic cycle relying on experimental equilibrium measurements (see the Supporting Information p. S66–S67). [b] Experimental number from ref. [37c]. [c] Experimental number from ref. [37b].

## Conclusion

Kinetic studies of the hydride transfer of BH_3_CN^−^ to C=O and C=N functionalities of α‐keto acids and related iminium ions were used to investigate the reaction mechanism and to study the relative electrophilic reactivities of these biologically relevant functionalities.

The relative electrophilic reactivities of keto acids in hydride transfer reactions are highly dependent on the pH as well as the presence or absence of buffer species that can act as general acid catalysts (Figure 4 and 5). In consequence, the observed reactivity differences can largely be attributed to the reactions proceeding via inter‐ vs. intramolecular acid catalysis: Intramolecular acid catalysis from internal carboxylic acid groups can be found for oxaloacetate (**K3**) and α‐ketoglutarate (**K4**), while only intermolecular catalysis can enhance the reaction rates of pyruvate (**K1**) or glyoxylate (**K2**) reductions.

Subjecting α‐keto acids to conditions of reductive amination resulted in a complex system of underlying reactions. Investigations of the equilibrium constants for imine/iminium formation and the acidities of the iminium ions were used to establish the pH‐dependent species‐distribution in the keto acid/NH_3_ system relevant for the understanding of the subsequent reduction reactions (Scheme 3, Figure 6). Analysis of the species distribution suggests an almost stationary concentration of iminium ions between pH 4 and 8. At pH 4, direct kinetic studies could be performed by ^1^H NMR spectroscopy and the product ratios were used to separate the kinetic contributions of the direct reduction from the reductive amination reaction (Figure 7). At [NH_4_^+^]=0.5 M, the reductive amination of glyoxylate, oxaloacetate and pyruvate are 11, 1.7 and 1.9 times faster, respectively, than that of α‐ketoglutarate (Table 2). The differences in intrinsic reactivities were furthermore verified by competition experiments conducted at pH 4–7. The competition experiments confirmed that the reactivity ordering of keto acids to undergo reductive amination does not change largely within this pH range. The contrasting pH‐dependency of keto acids to undergo reductive aminations or direct reductions can again be rationalized by acid catalysis. Reductive amination is predominantly governed by specific catalysis as the iminium ions are largely protonated at neutral pH and below. Consequently, reactivity differences are due to the underlying electronic nature of the substrate, rather than due to the mode of acid catalysis.

Extensive previous studies on the reactivities of electrophiles and nucleophiles have shown that electrophilicity is independent of the nature of the nucleophile. Accordingly, our kinetic and competition experiments with BH_3_CN^−^ as model nucleophile suggest that the reductive amination of α‐ketoglutarate over other keto acids is kinetically less favored than that of other keto acids; though, not by much. Moreover, the reductive amination of α‐ketoglutarate is also thermochemically less favored compared to pyruvate, oxaloacetate and glyoxylate. Accordingly, the usage of reductive amination to only synthesize glutamate within biological amino acid biosynthesis is not due to glutamate being intrinsically more accessible.

Why then did evolution select for amino acid biosynthesis to initiate with the kinetically and thermodynamically least favorable reductive amination? One possible answer lies within the thermodynamics of the subsequent transamination reactions.[^6^, ^37^] Despite their apparent similarities, glutamate is thermochemically a better amine‐donor when compared to alanine. Thus, formation of alanine from pyruvate and glutamate is exergonic. Even though the energetic preference in transamination is relatively small (Δ*G* ^0^=−1.04 kJ mol^−1^), synthesizing glutamate by reductive amination renders the subsequent transamination reactions to other α‐keto acids exergonic and might, therefore, establish glutamate as a high‐energy amine donor within amino acid synthesis.

## Conflict of interest

The authors declare no conflict of interest.

1

## Supporting information

As a service to our authors and readers, this journal provides supporting information supplied by the authors. Such materials are peer reviewed and may be re‐organized for online delivery, but are not copy‐edited or typeset. Technical support issues arising from supporting information (other than missing files) should be addressed to the authors.

Supporting Information

## Data Availability

The data that support the findings of this study are available in the supplementary material of this article.
