# Supplementary material for: Quantifying Reductive Amination in Nonenzymatic Amino Acid Synthesis
Source: Angew Chem Int Ed Engl. 2022 Oct 21;61(48):e202212237. doi: 10.1002/anie.202212237 (PMC9828492; doi:10.1002/anie.202212237)
Supplement: Supplementary file 1 — Supporting Information [file ANIE-61-0-s001.pdf]

Angewandte Chemie
Eine Zeitschrift der Gesellschaft Deutscher Chemiker

Supporting Information

Quantifying Reductive Amination in Nonenzymatic Amino Acid Synthesis

R. J. Mayer, J. Moran**

Contents

General **3**

Additional Figures **4**

Product Analysis of the Reaction of Pyruvate with $NaBH_3CN$ **5**

Kinetics of the Reaction of Keto Acids with $NaBH_3CN$ **7**

*Kinetics of the Reaction of Pyruvate (**K1**) with BH_3CN^-* *10*

*Data for the pH-Rate Profile for the Reaction of Pyruvate (**K1**) with BH_3CN^-* *13*

*Kinetics of the Reaction of Glyoxylate (**K2**) with BH_3CN^-* *19*

*Data for the pH-Rate Profile for the Reaction of Glyoxylate (**K2**) with BH_3CN^-* *22*

*Kinetics of the Reaction of Oxaloacetate (**K3**) with BH_3CN^-* *25*

*Data for the pH-Rate Profile for the Reaction of Oxaloacetate (**K3**) with BH_3CN^- (RM03-419)* *26*

*Kinetics of the Reaction of α-Ketoglutarate (**K4**) with BH_3CN^-* *28*

*Data for the pH-Rate Profile for the Reaction of α-Ketoglutarate (**K4**) with BH_3CN^- (RM03-448/454)* *29*

*pH-Dependency of Cyanohydrin Formation in the Reaction of Pyruvate (**K1**) with $NaBH_3CN$* *31*

*Exchange NMR Experiments of Hydrate Formation with Glyoxylate (**K2**)* *33*

Competition Experiments for Keto Acid Reduction *38*

Reductive Amination **42**

Equilibrium Constants for Imine Formation *42*

Kinetics of Reductive Amination – Mechanistic Analysis *45*

Species Distribution 45

Kinetic Model 48

Separation of Reaction Rates Based on the Product Ratio 50

Kinetics with Variation of [BH_3CN^-] *52*

Competition Experiments for Reductive Amination *61*

Thermochemistry of Reductive Amination **66**

DFT Computations **68**

Computation of $pK_{a,DFT}$ Values *68*

Reference Compounds for pK_a Determination of C=N-H Acids *69*

Acidity of Iminium Ions *73*

Gibbs Energy Profile for Imine Formation *74*

In-Situ IR Spectroscopy **76**

Geometries of Optimized Structures **77**

References **123**

General

Materials. Glyoxylic acid monohydrate (98%), α-ketoglutaric acid disodium salt dihydrate (≥98.0%), sodium pyruvate (ReagentPlus®, ≥99%), oxaloacetic acid (≥97%), sodium cyanoborohydride (PharmaGrade), and all other salts and reagents were purchased from Sigma-Aldrich.
Phosphate buffers (1 M) were prepared by mixing 1 M solutions of KH_2PO_4 (ReagentPlus®, Sigma-Aldrich), $KHPO_4$ (ACS reagent, ≥98%, Sigma-Aldrich) or phosphoric acid to the required pH as monitored by a pH meter calibrated before use. Acetate or formate buffers were prepared by neutralizing the respective amounts of acetic or formic acid with NaOH (VWR Chemicals, ACS Grade) while monitoring the pH. Water used for preparation of buffers or all other solutions was obtained from a Sartorius Arium purification system (18 MΩcm). To avoid potential contaminations (e.g. by metal ions), all reagents were handled with disposable plastic spatulas, and fresh vials were used wherever possible.

NMR Spectroscopy. NMR spectra were recorded on Bruker 400 or 500 MHz spectrometers equipped with Prodigy BBO cryoprobes at a sample temperature maintained at 23 °C. All studies in water were performed with a H_2O:D_2O mixture (11:1) if not noted otherwise, using qNMR grade dimethyl sulfone (DMS, Sigma-Aldrich, TraceCERT) or sodium trimethylsilylpropanesulfonate (DSS, Sigma-Aldrich, 97%) as internal standard. Water suppression in ^{1}H NMR spectra was achieved using the Bruker *noesygppr1d* pulse program acquiring 16 scans for each sample. For quantitative NMR spectra (qNMR), the relaxation delay d_1 was set to 30 s after having determined the T_1 relaxation times of all relevant resonances with the inversion recovery method. ^{1}H kinetics of fast reactions (< 120 min) were measured on a Bruker 400 MHz spectrometer equipped with a conventional BBO probe with the *zgesgp* or *noesygppr1d* pulse sequences using the multizg command with 4-16 scans per spectrum at 20 °C.

UV/Vis Kinetics. Spectra and kinetics were measured on a Jasco V-670 spectrophotometer equipped with a Peltier-cooled PAC-743 sample changer and an integrated magnetic stirrer at 20 °C. For measurements, Hellma 10 mm quartz glass cuvettes were employed containing a small magnetic stirring bar. Fast kinetics (< 10 min) were followed by continuously monitoring a single sample, while for slower measurements the parallel kinetic mode was employed where the sample-changer was used to continuously measure up to four samples simultaneously.

Kinetic Analysis. Concentration-time profiles were fitted to the single exponential $A[1 - \exp(-k_{obs}\,t)] + C$ for reactions with an exponential increase or the single exponential $A[\exp(-k_{obs}\,t)] + C$ for exponential decrease.

Error Analysis. All reported errors correspond to standard errors calculated from regression analyses. In case of quantities calculated from values for which standard errors are of relevance, errors were calculated by means of an uncertainty propagation using the partial derivatives.[S1]

Additional Figures

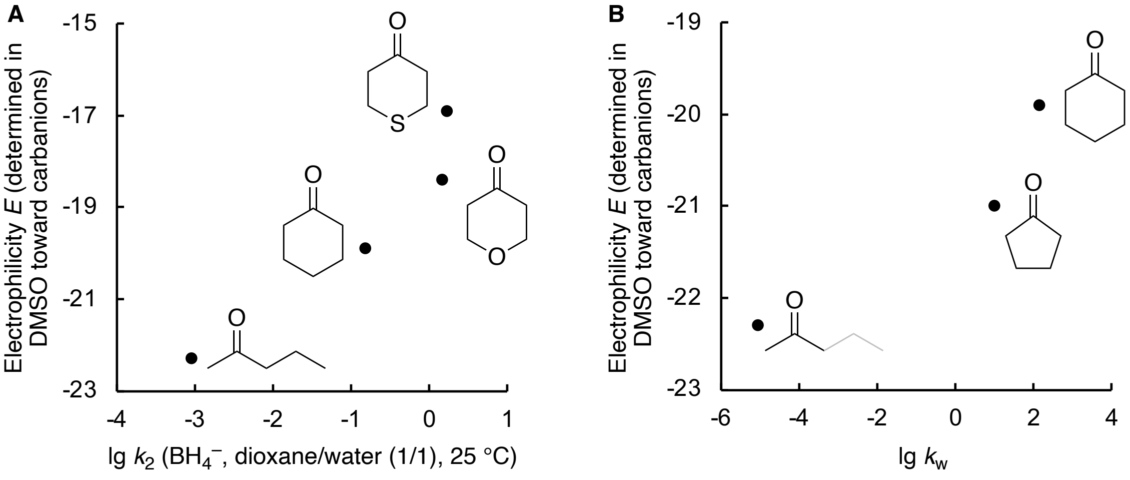

Figure S1. (A) Correlation of Mayr's electrophilicity parameters E[S2] vs. rate constants for the reaction of ketones with BH_4^- reported by Geneste et al. in dioxane/water (1/1).[S3] (B) Correlation of Mayr's electrophilicity parameters E vs. hydration rate constants lg k_w.[S4] *Note: lg k_w was only reported for acetone while an E parameter is only available for 2-pentanone.*

Product Analysis of the Reaction of Pyruvate with $NaBH_3CN$

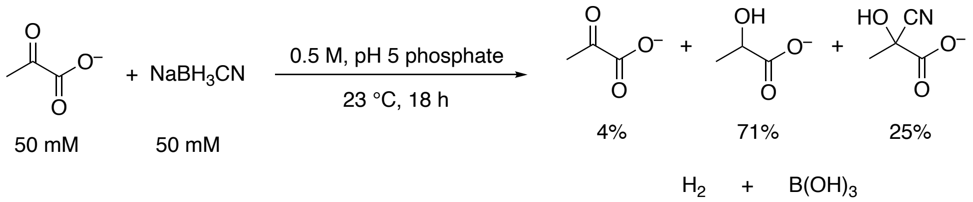

A NMR tube was charged from stock solutions to give a mixture of pyruvate (50 mM) and $NaBH_3CN$ (50 mM) in 0.5 M, pH 5 phosphate buffer containing 8.3% D_2O. After 18 h, the sample was analyzed by ^1H and ^{11}B NMR spectroscopy. Besides unreacted pyruvate (4%) and BH_3CN^-, lactate (71%), the cyanohydrin of pyruvate (25%) and H_2 were detected in the ^1H NMR spectra. ^{11}B NMR analysis additionally showed the formation of a signal at 19.5 ppm which was identified by comparison with an authentic sample as boric acid.

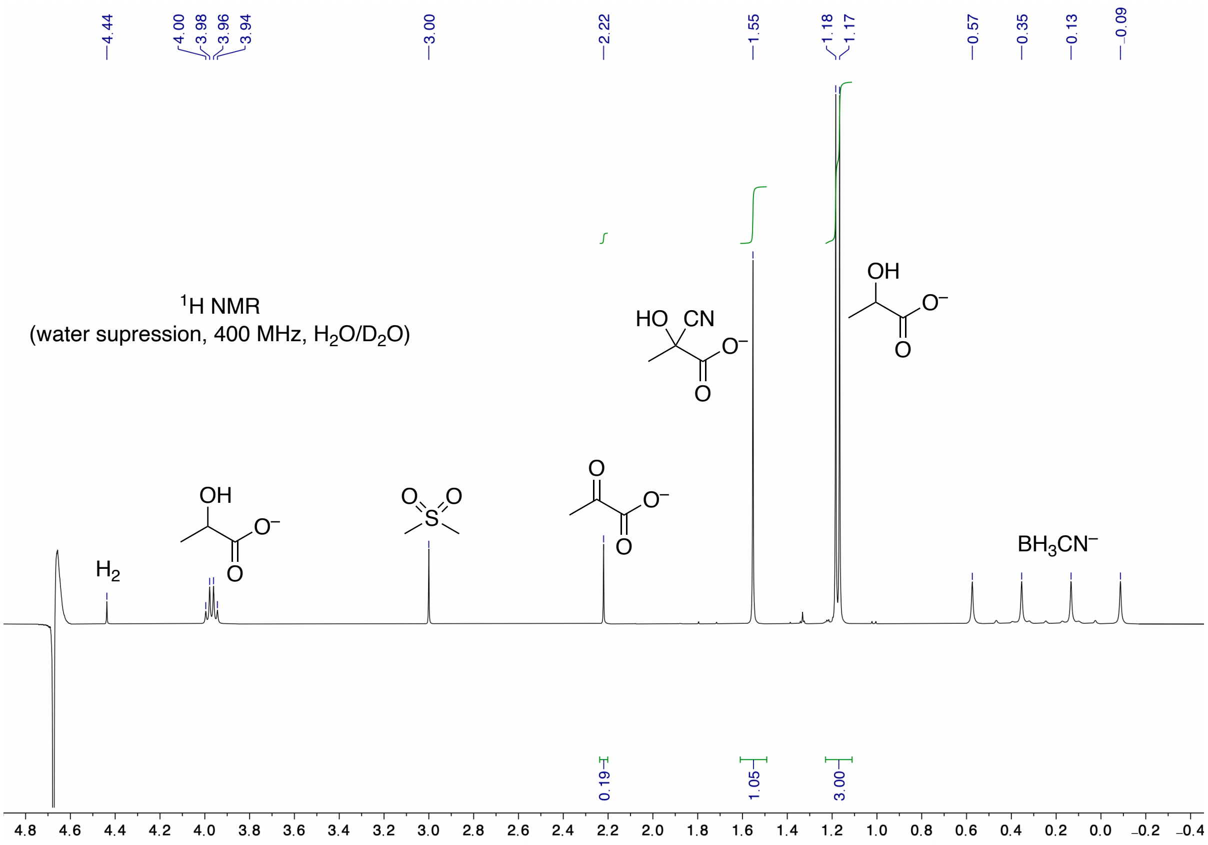

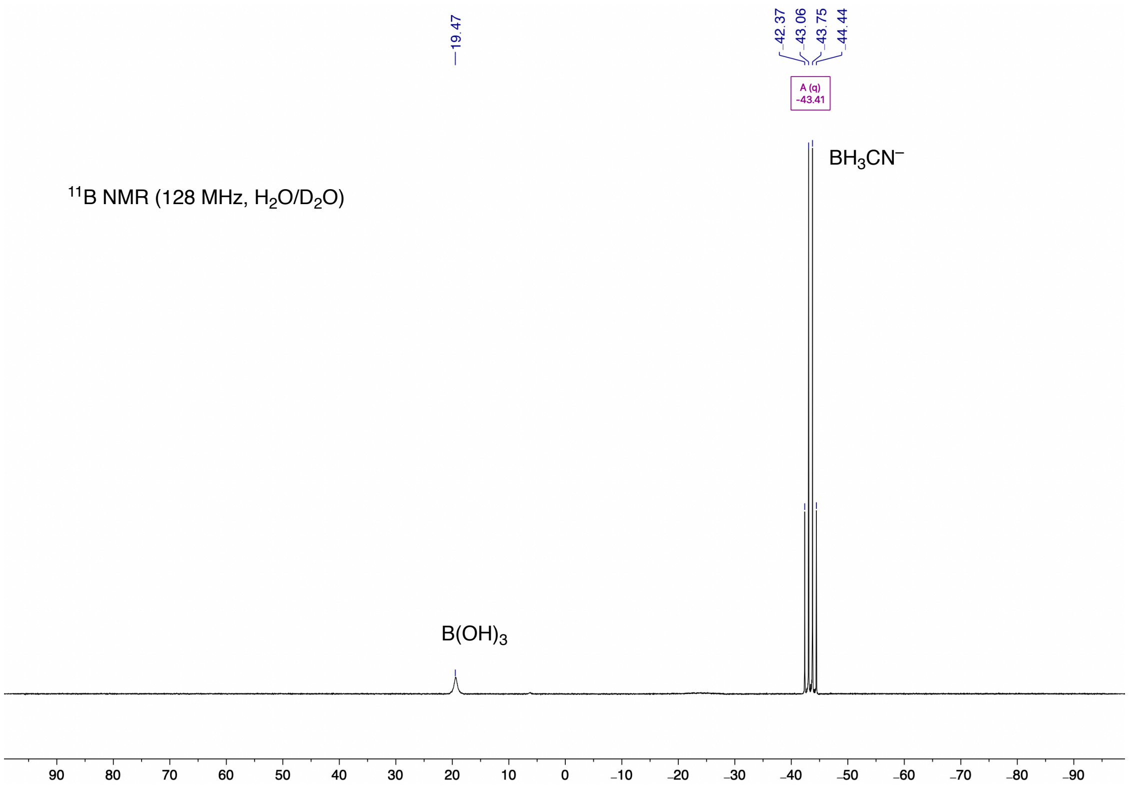
19.47
42.37
43.06
43.75
44.44
A (q)
-43.41
BH3CN−
11B NMR (128 MHz, H2O/D2O)
B(OH)3
90 80 70 60 50 40 30 20 10 0 -10 -20 -30 -40 -50 -60 -70 -80 -90

Kinetics of the Reaction of Keto Acids with $NaBH_3CN$

Initially, the reactions of all keto acids with $NaBH_3CN$ at pH 4 were followed by ^1H NMR spectroscopy to verify the formation of the correct reaction products under the conditions of the kinetic experiments mainly followed by UV/Vis spectroscopy.

NMR spectra showed that at pH 4 only traces of the cyanohydrin by-product formed in reactions of keto acids with BH_3CN^- (typically < 2% according to NMR spectra in phosphate buffer) and that the rates for the disappearance of the keto acid are accordingly identical with those for the formation of the hydroxy acid. Therefore, UV/Vis spectroscopy can be used to monitor the reaction kinetics as keto acids **K1**, **K3** and **K4** feature a sufficiently strong carbonyl absorbance. Due to the lack of a sufficiently strong UV absorbance of the carbonyl group of **K2**, the full set of buffer-dependent kinetics was determined by NMR spectroscopy.

Procedure for NMR Kinetics

NMR tubes were charged with a solution of dimethyl sulfone in D_2O (50 µL) and a stock solution of the keto acid, phosphate buffer and water. Lastly, the corresponding amount of a stock solution of $NaBH_3CN$ was added, the NMR tube sealed, mixed and a timer started. The NMR tube was inserted into the spectrometer which shortly before was locked and shimmed on an NMR sample of similar composition, and the measurement started. The time difference from mixing the sample and acquisition of the first spectrum was noted and used to correct the kinetics. For the kinetics, ^1H NMR spectra were continuously acquired with either ns = 4 (for oxaloacetic acid) or ns = 8 (for glyoxylate and α-ketoglutarate). Due to the longer reaction time, kinetics with pyruvate were measured on multiple samples in parallel using an autosampler that continuously changed the tubes. NMR spectra of the kinetics for representative conditions are depicted below for all four keto acids.

Spectra were analyzed with the Data Analysis module in *Mnova* after phase and baseline correction (see Figure S2-S5 for some examples) to obtain consistent integral values throughout the whole kinetic experiment. Absolute concentrations were then calculated from the raw data in *Excel* and the time/concentration data was analyzed by least-squares fitting as outlined in the general section.

Procedure for UV/Vis Kinetics

UV/Vis kinetics were acquired for four samples in parallel at T = 20.0 °C and stirring on 2000 µL scale. Quartz cuvettes were charged with a stirring bar, buffer, a sodium chloride solution (to maintain the ionic strength), water and the corresponding amount of a stock solution of $NaBH_3CN$. The background was recorded, a solution of the ketoacid was added and the measurement started. The absorbance of each sample was recorded every 12.15 s. The time-dependent decay of the keto acid absorbance was analyzed by least-squares fitting to afford the pseudo-first order rate constants k_{obs}. Correlations of k_{obs} vs. $[BH_3CN^-]$ were used to determine apparent second-order rate constants $k_{2'}$ for a specific buffer concentration. Finally, the correlation of $k_{2'}$ vs. the buffer concentration was used to determine the buffer-independent second-order rate constants $k_{2,0}$. All reported errors correspond to standard errors.

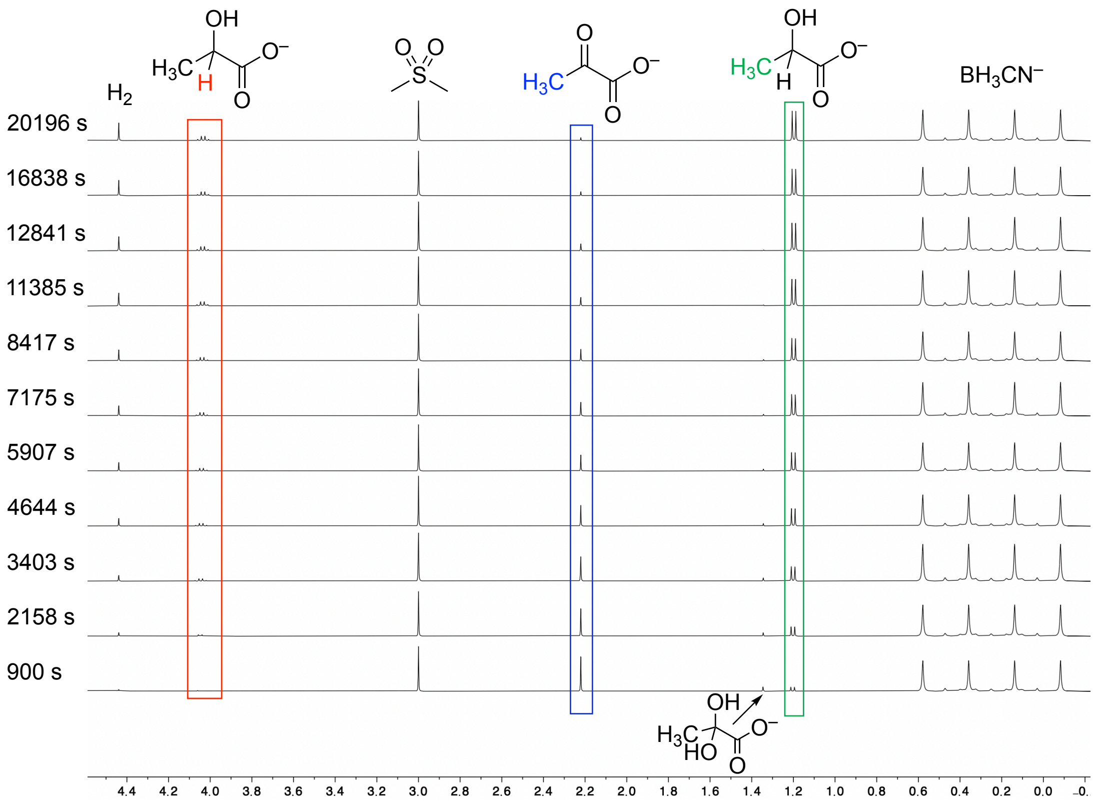

Figure S2. ^{1}H NMR kinetics (noesygppr1d, 400 MHz, ns = 16, 23 °C) of the reaction of **K1** (3.3 mM) with $NaBH_3CN$ (40.2 mM) in pH 4 phosphate solution (0.5 M) in H_2O containing 8.3% D_2O (RM02-315-3).

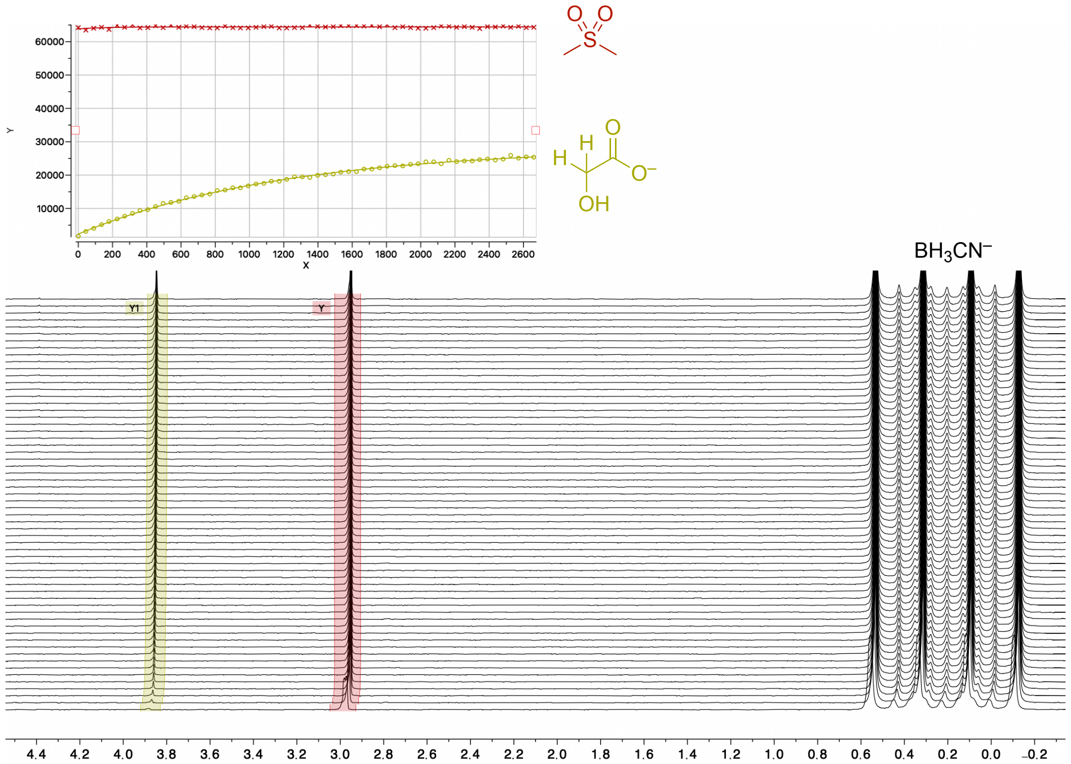

Figure S3. ^{1}H NMR kinetics (zgesgp, 400 MHz, ns = 8, 20 °C) of the reaction of **K2** (3.3 mM) with $NaBH_3CN$ (51.7 mM) in pH 4 phosphate solution (0.5 M) in H_2O containing 8.3% D_2O (RM02-312-4). *Insert on top: Unprocessed time-dependent peak areas from the data analysis module in MNOVA (correlation lines correspond to a tentative exponential fit).*

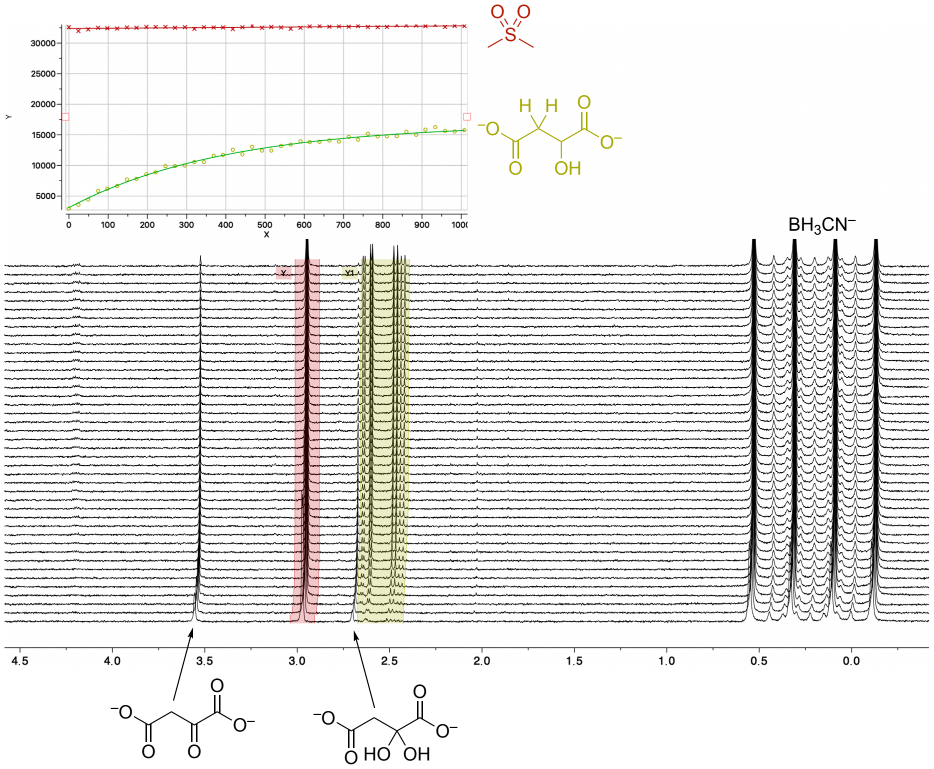

Figure S4. ^{1}H NMR kinetics (zgesgp, 400 MHz, ns = 4, 20 °C) of the reaction of **K3** (2.3 mM) with $NaBH_3CN$ (17.2 mM) in pH 4 phosphate solution (0.5 M) in H_2O containing 8.3% D_2O (RM02-313-1). *Insert on top: Unprocessed time-dependent peak areas from the data analysis module in MNOVA (correlation lines correspond to a tentative exponential fit).*

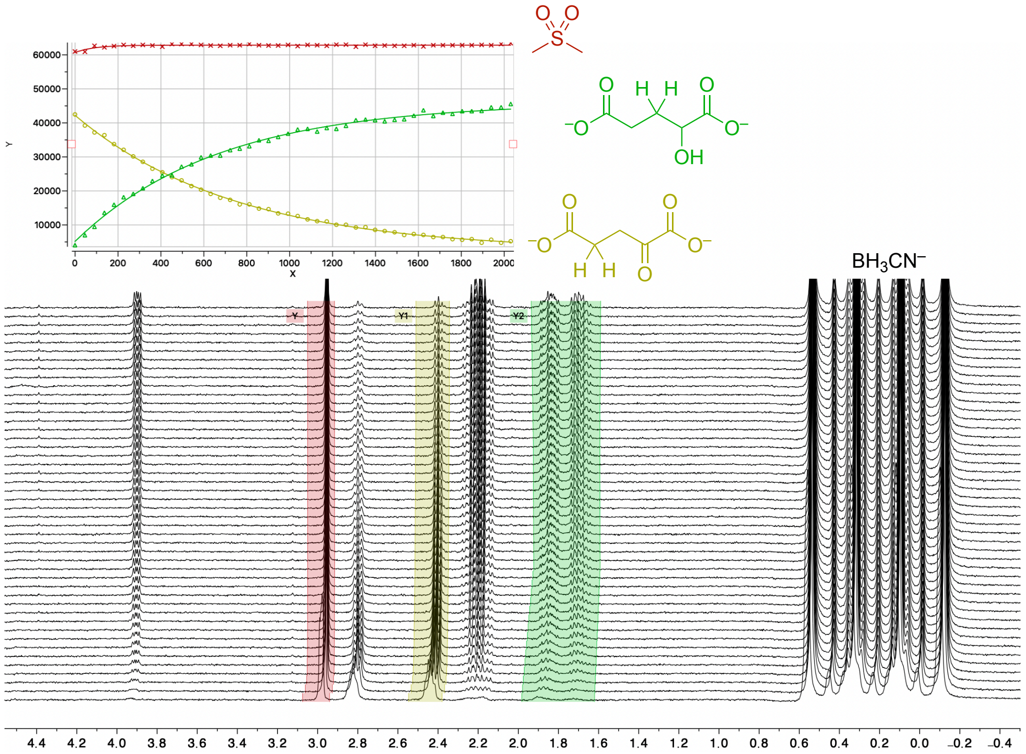

Figure S5. ^{1}H NMR kinetics (zgesgp, 400 MHz, ns = 8, 20 °C) of the reaction of **K4** (3.3 mM) with $NaBH_3CN$ (51.7 mM) in pH 4 phosphate solution (0.5 M) in H_2O containing 8.3% D_2O (RM02-310-4). *Insert on top: Unprocessed time-dependent peak areas from the data analysis module in MNOVA (correlation lines correspond to a tentative exponential fit)..*

Kinetics of the Reaction of Pyruvate (**K1**) with BH_3CN^-

Kinetics of pyruvate disappearance in the reaction of pyruvate with $NaBH_3CN$ followed by UV/Vis spectroscopy (317 nm) at 20.0 °C in pH 4 acetate solution (**$[buffer]_{total}$ = 0.10 M**) in H_2O (RM03-414). The ionic strength was maintained at I = 1 considering the concentration of AcO^- and $NaBH_3CN$.

[**K1**] / M	[$NaBH_3CN$] / M	$[buffer]_{total}$ / M	[HOAc] / M	[AcO^-] / M	[NaCl] / M	k_{obs} / s^{-1}
5.00×10^{-3}	5.00×10^{-2}	1.00×10^{-1}	8.52×10^{-2}	1.48×10^{-2}	9.35×10^{-1}	1.06×10^{-4}
5.00×10^{-3}	6.66×10^{-2}	1.00×10^{-1}	8.52×10^{-2}	1.48×10^{-2}	9.19×10^{-1}	1.29×10^{-4}
5.00×10^{-3}	8.42×10^{-2}	1.00×10^{-1}	8.52×10^{-2}	1.48×10^{-2}	9.01×10^{-1}	1.82×10^{-4}
5.00×10^{-3}	1.00×10^{-1}	1.00×10^{-1}	8.52×10^{-2}	1.48×10^{-2}	8.85×10^{-1}	2.18×10^{-4}

$k_{2'} = (2.32 \pm 0.22) \times 10^{-3}$ L mol^{-1} s^{-1}

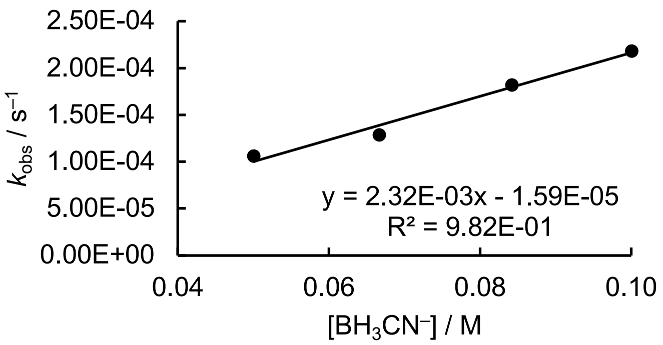

Kinetics of pyruvate disappearance in the reaction of pyruvate with $NaBH_3CN$ followed by UV/Vis spectroscopy (317 nm) at 20.0 °C in pH 4 acetate solution (**$[buffer]_{total}$ = 0.25 M**) in H_2O (RM03-414). The ionic strength was maintained at I = 1 considering the concentration of AcO^- and $NaBH_3CN$.

[**K1**] / M	[$NaBH_3CN$] / M	$[buffer]_{total}$ / M	[HOAc] / M	[AcO^-] / M	[NaCl] / M	k_{obs} / s^{-1}
5.00×10^{-3}	5.00×10^{-2}	2.50×10^{-1}	2.13×10^{-1}	3.70×10^{-2}	9.13×10^{-1}	1.54×10^{-4}
5.00×10^{-3}	6.66×10^{-2}	2.50×10^{-1}	2.13×10^{-1}	3.70×10^{-2}	8.96×10^{-1}	2.02×10^{-4}
5.00×10^{-3}	8.42×10^{-2}	2.50×10^{-1}	2.13×10^{-1}	3.70×10^{-2}	8.79×10^{-1}	2.53×10^{-4}
5.00×10^{-3}	1.00×10^{-1}	2.50×10^{-1}	2.13×10^{-1}	3.70×10^{-2}	8.63×10^{-1}	3.07×10^{-4}

$k_{2'} = (3.04 \pm 0.09) \times 10^{-3}$ L mol^{-1} s^{-1}

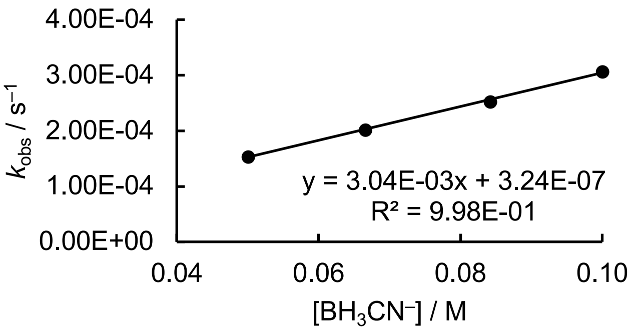

Kinetics of pyruvate disappearance in the reaction of pyruvate with $NaBH_3CN$ followed by UV/Vis spectroscopy (317 nm) at 20.0 °C in pH 4 acetate solution (**$[buffer]_{total}$ = 0.40 M**) in H_2O (RM03-414). The ionic strength was maintained at I = 1 considering the concentration of AcO^- and $NaBH_3CN$.

[**K1**] / M	[$NaBH_3CN$] / M	$[buffer]_{total}$ / M	[HOAc] / M	[AcO^-] / M	[NaCl] / M	k_{obs} / s^{-1}
5.00×10^{-3}	5.00×10^{-2}	4.00×10^{-1}	3.41×10^{-1}	5.92×10^{-2}	8.91×10^{-1}	2.10×10^{-4}
5.00×10^{-3}	6.66×10^{-2}	4.00×10^{-1}	3.41×10^{-1}	5.92×10^{-2}	8.74×10^{-1}	2.77×10^{-4}
5.00×10^{-3}	8.42×10^{-2}	4.00×10^{-1}	3.41×10^{-1}	5.92×10^{-2}	8.57×10^{-1}	3.29×10^{-4}
5.00×10^{-3}	1.00×10^{-1}	4.00×10^{-1}	3.41×10^{-1}	5.92×10^{-2}	8.41×10^{-1}	3.95×10^{-4}

$k_{2'} = (3.62 \pm 0.17) \times 10^{-3}$ L mol^{-1} s^{-1}

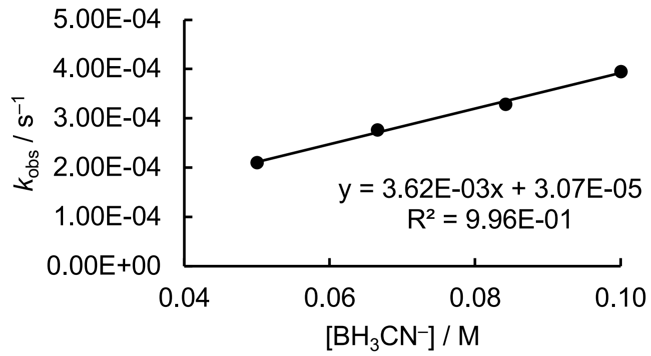

Kinetics of pyruvate disappearance in the reaction of pyruvate with $NaBH_3CN$ followed by UV/Vis spectroscopy (317 nm) at 20.0 °C in pH 4 acetate solution (**$[buffer]_{total}$ = 0.55 M**) in H_2O (RM03-414). The ionic strength was maintained at I = 1 considering the concentration of AcO^- and $NaBH_3CN$.

[**K1**] / M	[$NaBH_3CN$] / M	$[buffer]_{total}$ / M	[HOAc] / M	[AcO^-] / M	[NaCl] / M	k_{obs} / s^{-1}
5.00×10^{-3}	5.00×10^{-2}	5.50×10^{-1}	4.69×10^{-1}	8.14×10^{-2}	8.69×10^{-1}	2.65×10^{-4}
5.00×10^{-3}	6.66×10^{-2}	5.50×10^{-1}	4.69×10^{-1}	8.14×10^{-2}	8.52×10^{-1}	3.55×10^{-4}
5.00×10^{-3}	8.42×10^{-2}	5.50×10^{-1}	4.69×10^{-1}	8.14×10^{-2}	8.34×10^{-1}	4.10×10^{-4}
5.00×10^{-3}	1.00×10^{-1}	5.50×10^{-1}	4.69×10^{-1}	8.14×10^{-2}	8.19×10^{-1}	4.94×10^{-4}

$k_{2'} = (4.42 \pm 0.32) \times 10^{-3}$ L mol^{-1} s^{-1}

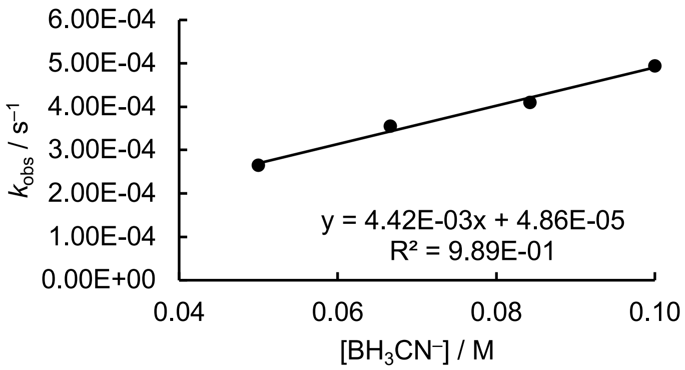

Correlation of $k_{2'}$ against the concentration of the buffer allows to determine the buffer-independent second-order rate constant for reduction $k_{2,0}$ from the intercept with the ordinate.

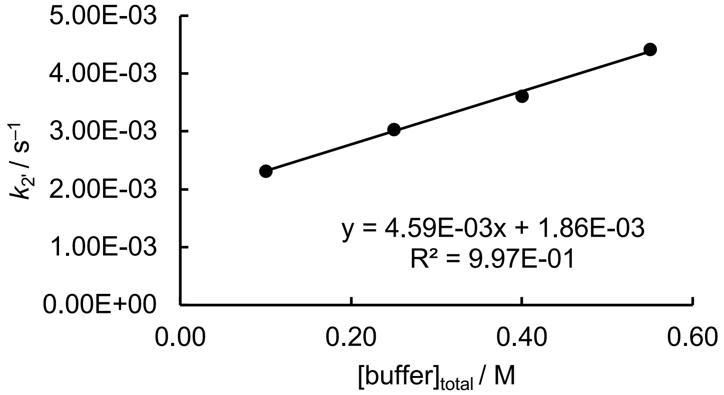

$k_{2,0} = (1.86 \pm 0.07) \times 10^{-3}$ L mol^{-1} s^{-1}

Data for the pH-Rate Profile for the Reaction of Pyruvate (**K1**) with BH_3CN^-

Measurements at constant pH but different concentration of buffers were used to determine the buffer-independent rates k_0 (at a concentration of [$NaBH_3CN$] = 50 mM) from the correlations of k_{obs} with the concentration of the acidic component of the buffer. Assuming that the buffers react predominantly via acid catalysis (mechanistically, base catalysis by the buffer seems implausible), the slope of these correlations provides an estimate for the catalytic constant of the buffer k_{HA}.

Kinetics of pyruvate disappearance in the reaction of pyruvate with $NaBH_3CN$ (50 mM) followed by UV/Vis spectroscopy (317 nm) at 20.0 °C in pH 3 formate buffer (0.1-0.55 M) in H_2O (RM03-409-pH3). The ionic strength was maintained at I = 1 considering the concentration of HCO_2^- and $NaBH_3CN$. The fraction of protonated and deprotonated acid was calculated using a pK_a value of 3.745.

[**K1**] / M	[$NaBH_3CN$] / M	[buffer]$_{total}$ / M	[HCO_2H] / M	[HCO_2^-] / M	[NaCl] / M	k_{obs} / s^{-1}
5.00×10^{-3}	5.00×10^{-2}	1.00×10^{-1}	8.48×10^{-2}	1.52×10^{-2}	9.35×10^{-1}	1.73×10^{-3}
5.00×10^{-3}	5.00×10^{-2}	2.50×10^{-1}	2.12×10^{-1}	3.81×10^{-2}	9.12×10^{-1}	3.24×10^{-3}
5.00×10^{-3}	5.00×10^{-2}	4.00×10^{-1}	3.39×10^{-1}	6.10×10^{-2}	8.89×10^{-1}	4.13×10^{-3}
5.00×10^{-3}	5.00×10^{-2}	5.50×10^{-1}	4.66×10^{-1}	8.39×10^{-2}	8.66×10^{-1}	5.00×10^{-3}

$k_0 = (1.21 \pm 0.27) \times 10^{-3}\ s^{-1}$

$k_{HA} = (8.42 \pm 0.86) \times 10^{-3}\ M^{-1}\ s^{-1}$

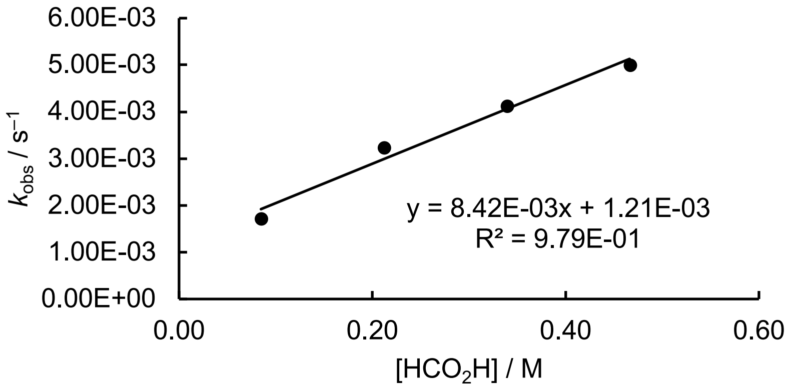

Kinetics of pyruvate disappearance in the reaction of pyruvate with $NaBH_3CN$ followed by UV/Vis spectroscopy (317 nm) at 20.0 °C in pH 4 acetate solution (0.1-0.5 M) in H_2O (RM03-414). The ionic strength was maintained at I = 1 considering the concentration of AcO^- and $NaBH_3CN$. The fraction of protonated and deprotonated acid was calculated using a pK_a value of 4.76.

[**K1**] / M	[$NaBH_3CN$] / M	[buffer]$_{total}$ / M	[HOAc] / M	[AcO^-] / M	[NaCl] / M	k_{obs} / s^{-1}
5.00×10^{-3}	5.00×10^{-2}	1.00×10^{-1}	8.52×10^{-2}	1.48×10^{-2}	9.35×10^{-1}	1.06×10^{-4}
5.00×10^{-3}	5.00×10^{-2}	2.50×10^{-1}	2.13×10^{-1}	3.70×10^{-2}	9.13×10^{-1}	1.54×10^{-4}
5.00×10^{-3}	5.00×10^{-2}	4.00×10^{-1}	3.41×10^{-1}	5.92×10^{-2}	8.91×10^{-1}	2.10×10^{-4}
5.00×10^{-3}	5.00×10^{-2}	5.50×10^{-1}	4.69×10^{-1}	8.14×10^{-2}	8.69×10^{-1}	2.65×10^{-4}

$k_0 = (6.83 \pm 0.31) \times 10^{-5}\ s^{-1}$

$k_{HA} = (4.17 \pm 0.10) \times 10^{-4}\ M^{-1}\ s^{-1}$

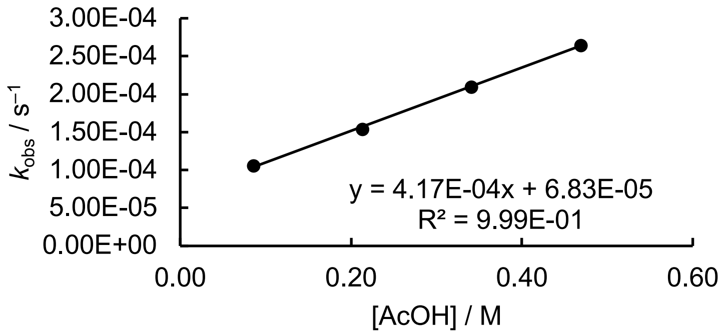

Kinetics of pyruvate disappearance in the reaction of pyruvate with $NaBH_3CN$ (50 mM) followed by ^1H NMR spectroscopy at 20.0 °C in pH 5 acetate buffer (0.1-0.5 M) in H_2O (RM03-409-pH5). The ionic strength was maintained at I = 1 considering the concentration of AcO^- and $NaBH_3CN$. The fraction of protonated and deprotonated acid was calculated using a pK_a value of 4.76.

[**K1**] / M	[$NaBH_3CN$] / M	[buffer]$_{total}$ / M	[HOAc] / M	[AcO^-] / M	[NaCl] / M	k_{obs} / s^{-1}
5.00×10^{-3}	5.00×10^{-2}	1.00×10^{-1}	3.65×10^{-2}	6.35×10^{-2}	8.87×10^{-1}	4.57×10^{-5}
5.00×10^{-3}	5.00×10^{-2}	2.33×10^{-1}	8.51×10^{-2}	1.48×10^{-1}	8.02×10^{-1}	8.87×10^{-5}
5.00×10^{-3}	5.00×10^{-2}	3.66×10^{-1}	1.34×10^{-1}	2.32×10^{-1}	7.18×10^{-1}	1.58×10^{-4}
5.00×10^{-3}	5.00×10^{-2}	5.00×10^{-1}	1.83×10^{-1}	3.17×10^{-1}	6.33×10^{-1}	1.92×10^{-4}

$k_0 = (6.84 \pm 11.2) \times 10^{-6}$ s^{-1}

$k_{HA} = (1.04 \pm 0.10) \times 10^{-3}$ M^{-1} s^{-1}

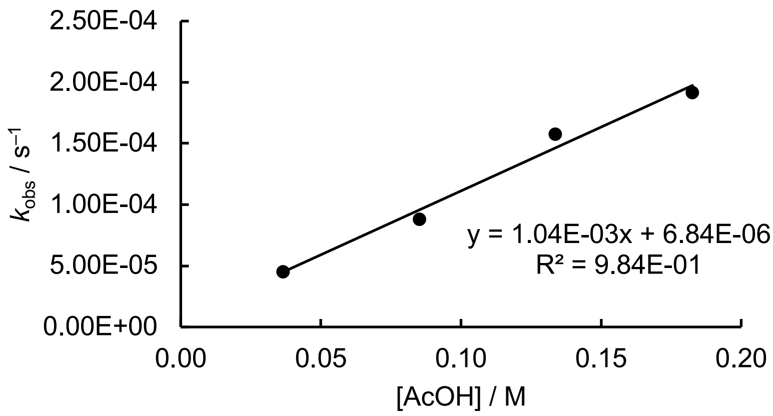

Kinetics of pyruvate disappearance in the reaction of pyruvate with $NaBH_3CN$ (50 mM) followed by ^{1}H NMR spectroscopy at 20.0 °C in pH 6 phosphate buffer (0.1-0.6 M) in H_2O (RM03-409-pH6). The ionic strength was maintained at I = 1 considering the concentration of HPO_4^{2-}, $H_2PO_4^-$ and $NaBH_3CN$. The fraction of protonated and deprotonated acid was calculated using a pK_a value of 7.52.

[**K1**] / M	[$NaBH_3CN$] / M	[buffer]$_{total}$ / M	[$H_2PO_4^-$] / M	[NaCl] / M	k_{obs} / s^{-1}
5.00×10^{-3}	5.00×10^{-2}	1.00×10^{-1}	9.71×10^{-2}	8.29×10^{-1}	1.00×10^{-5}
5.00×10^{-3}	5.00×10^{-2}	2.66×10^{-1}	2.58×10^{-1}	6.29×10^{-1}	2.00×10^{-5}
5.00×10^{-3}	5.00×10^{-2}	4.33×10^{-1}	4.20×10^{-1}	4.28×10^{-1}	2.98×10^{-5}
5.00×10^{-3}	5.00×10^{-2}	6.00×10^{-1}	5.82×10^{-1}	2.26×10^{-1}	3.92×10^{-5}

$k_0 = (4.29 \pm 0.24) \times 10^{-6}\ s^{-1}$

$k_{HA} = (6.02 \pm 0.06) \times 10^{-5}\ M^{-1}\ s^{-1}$

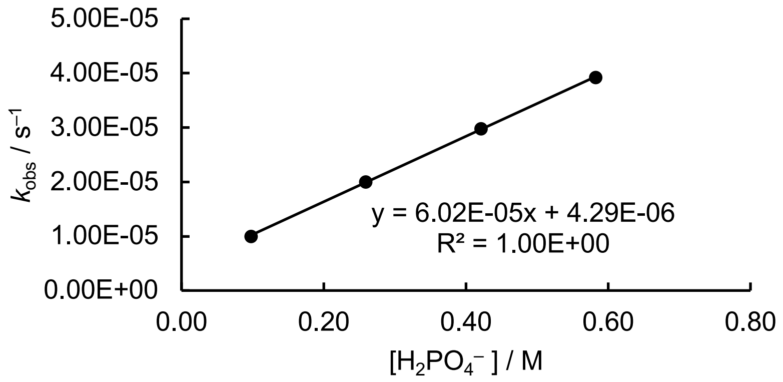

Kinetics of pyruvate disappearance in the reaction of pyruvate with $NaBH_3CN$ (50 mM) followed by ^{1}H NMR spectroscopy at 20.0 °C in pH 7 phosphate buffer (0.18-0.45 M) in H_2O (RM03-409-pH7). The ionic strength was maintained at I = 1 considering the concentration of HPO_4^{2-}, $H_2PO_4^-$ and $NaBH_3CN$. The fraction of protonated and deprotonated acid was calculated using a pK_a value of 7.52.

[**K1**] / M	[$NaBH_3CN$] / M	[buffer]$_{total}$ / M	[$H_2PO_4^-$] / M	[NaCl] / M	k_{obs} / s^{-1}
5.00×10^{-3}	5.00×10^{-2}	1.83×10^{-1}	1.41×10^{-1}	5.71×10^{-1}	5.91×10^{-6}
5.00×10^{-3}	5.00×10^{-2}	3.16×10^{-1}	2.43×10^{-1}	2.96×10^{-1}	9.00×10^{-6}
5.00×10^{-3}	5.00×10^{-2}	4.50×10^{-1}	3.46×10^{-1}	1.80×10^{-2}	1.09×10^{-5}

$k_0 = (2.69 \pm 0.87) \times 10^{-6}\ s^{-1}$

$k_{HA} = (2.43 \pm 0.34) \times 10^{-5}\ M^{-1}\ s^{-1}$

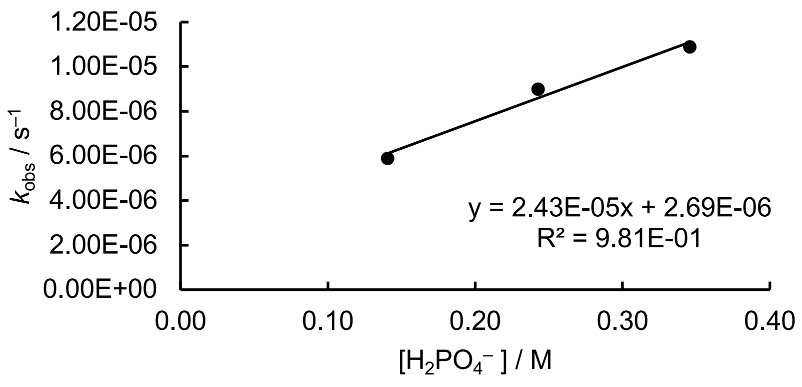

Analysis of the pH-rate profile

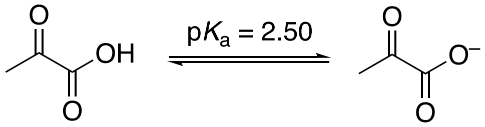

pH	k_0 / s^{-1}	lg k_0
3	(1.21 ± 0.27) x 10^{-3}	-2.92
4	(6.83 ± 0.31) x 10^{-5}	-4.17
5	(6.84 ± 11.2) x 10^{-6}	-5.16
6	(4.29 ± 0.24) x 10^{-6}	-5.37
7	(2.69 ± 0.87) x 10^{-6}	-5.57

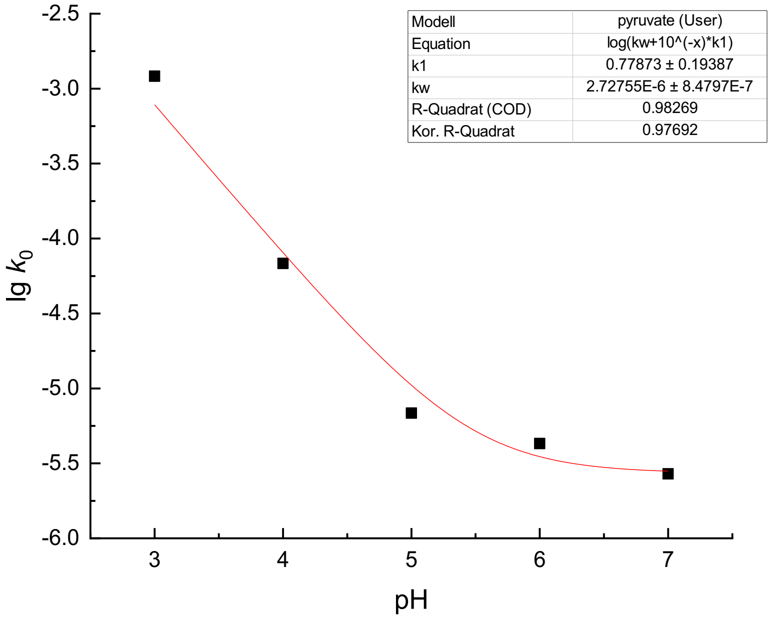

Figure S6. pH-rate profile for the reaction of **K1** with BH_3CN^- (50 mM). The pH-rate profile was analyzed with the relation $k_0 = k_1[H^+] + k_w$. Non-linear regression afforded values for k_1 = 0.78 ± 0.19 and k_w = (2.73 ± 0.85) x 10^{-6}.

Brønsted Plot

For both acetate and phosphate buffers, k_{HA} was determined at two different pH values resulting in values of k_{HA} differing by a factor of approximately 2.5. The reason for this discrepancy is unknown as for a purely acid catalyzed reaction k_{HA} should be independent of the pH; mechanistically, however, contributions of base-catalysis are not plausible. When we determined k_{HA} from the slope of correlations of k_{obs} vs. [acetate buffer] at further pH values, we observed a non-systematic scattering with values differing by a maximum of a factor of 4 which might in part be due to experimental error. We, therefore, decided to use the average of the k_{HA} values determined at different pH values to construct the Brønsted plot of the reaction.

Table S1. Values used to construct the Brønsted plot for reactions of **K1** with $[BH_3CN^-]$ = 50 mM at 20 °C.

Buffer	pK_a + lg p/q[a]	k_{HA} / M^{-1} s^{-1}	lg k_{HA}
HCO_2H	3.745	(8.42 ± 0.86) x 10^{-3}	−2.07
AcOH	4.76	(4.17 ± 0.10) x 10^{-4} (pH 4)<br>(1.04 ± 0.10) x 10^{-3} (pH 5)	−2.18 ± 0.20
$H_2PO_4^-$	7.52 + lg 2/1	(6.02 ± 0.06) x 10^{-5} (pH 6)<br>(2.43 ± 0.34) x 10^{-5} (pH 7)	−4.42 ± 0.20
H_2O	14[b]	2.47 x 10^{-6} s^{-1} / 55.5 M<br>(from pH-rate profile)	−7.3

[a] In Brønsted plots, the pK_a values of multiprotic acids have to be statistically corrected using the number of available protons *p* and the number of basic sites *q*. For our systems, this is only relevant for the $H_2PO_4^-$ ion. [b] In contrast to the analysis done by Brønsted, we decided to use the pK_a value of 14 for water as the usage of a value of 15.54 was recently shown to be thermodynamically incorrect.[S5]

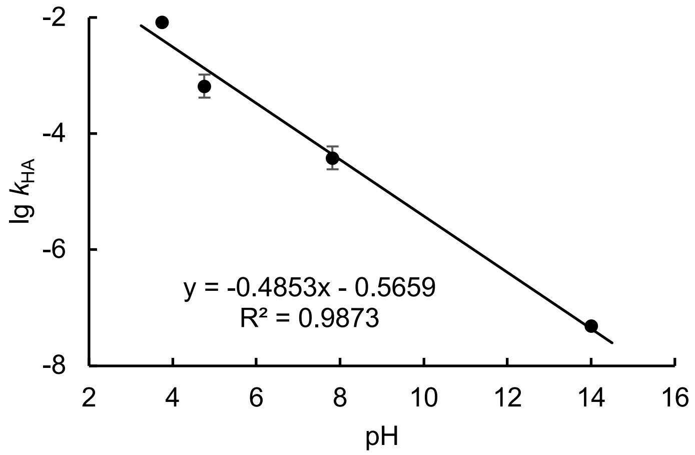

Figure S7. Brønsted plot for the reaction of **K1** with $[BH_3CN^-]$ = 50 mM at 20 °C.

Evaluation of the Effect of Boric Acid on the Reaction

As boric acid forms in the course of the reaction (cf. p. S6), it might affect the reaction rates due to its ability to react as Brønsted acid, Lewis acid, hydrogen bond donor and hydrogen bond acceptor. To test for a potential effect, we have investigated the reduction reaction of pyruvate with BH_3CN^- in 0.1 M acetate buffer (corresponding to the lowest buffer concentration that we have used) at pH 4 with different concentrations of added boric acid. As we have conducted the kinetic measurements in this project under pseudo-first order conditions at a keto acid concentration of typically 5 mM, a maximum of 5 mM $B(OH)_3$ should form in the reaction. Analysis of k_{obs} as a function of the concentration of added boric acid showed no significant change in the rate constants even with 6 x larger concentration of boric acid then present at the end of our "standard" experiments.

Kinetics of pyruvate disappearance in the reaction of pyruvate with $NaBH_3CN$ followed by UV/Vis spectroscopy (317 nm) at 20.0 °C in pH 4 acetate solution (**$[buffer]_{total}$ = 0.10 M**) in H_2O with different amounts of boric acid added (RM03-480). The ionic strength was maintained at I = 1 considering the concentration of AcO^- and $NaBH_3CN$. The stock solution of boric acid was adjusted to pH 4 to avoid changes in the pH.

[**K1**] / M	[$NaBH_3CN$] / M	[$B(OH)_3$]	$[buffer]_{total}$ / M	[HOAc] / M	[AcO^-] / M	[NaCl] / M	k_{obs} / s^{-1}
5.00×10^{-3}	1.00×10^{-1}	0	1.00×10^{-1}	8.52×10^{-2}	1.48×10^{-2}	8.85×10^{-1}	2.49×10^{-4}
5.00×10^{-3}	1.00×10^{-1}	1.00×10^{-2}	1.00×10^{-1}	8.52×10^{-2}	1.48×10^{-2}	8.85×10^{-1}	2.14×10^{-4}
5.00×10^{-3}	1.00×10^{-1}	2.00×10^{-2}	1.00×10^{-1}	8.52×10^{-2}	1.48×10^{-2}	8.85×10^{-1}	2.71×10^{-4}
5.00×10^{-3}	1.00×10^{-1}	3.00×10^{-2}	1.00×10^{-1}	8.52×10^{-2}	1.48×10^{-2}	8.85×10^{-1}	2.56×10^{-4}

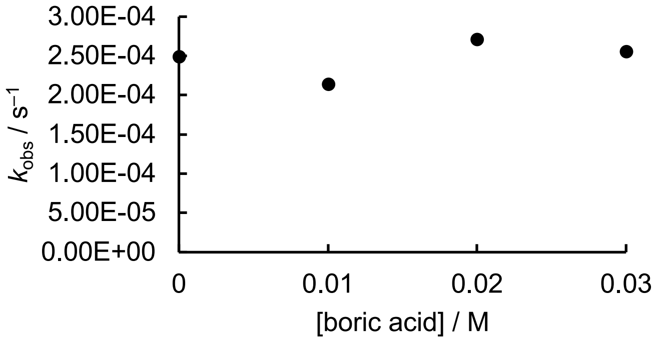

Kinetics of the Reaction of Glyoxylate (**K2**) with $BH_3CN^–$

Kinetics of glycolate formation in the reaction of glyoxylate with $NaBH_3CN$ followed by ^1H NMR spectroscopy at 20.0 °C in pH 4 acetate solution (**$[buffer]_{total}$ = 0.1 M**) in H_2O (RM03-408). The ionic strength was maintained at I = 1 considering the concentration of $AcO^–$ and $NaBH_3CN$.

[**K2**] / M	[$NaBH_3CN$] / M	$[buffer]_{total}$ / M	[HOAc] / M	[$AcO^–$] / M	[NaCl] / M	k_{obs} / $s^{–1}$
3.30 x $10^{–3}$	3.30 x $10^{–2}$	1.00 x $10^{–1}$	8.52 x $10^{–2}$	1.48 x $10^{–2}$	9.52 x $10^{–1}$	5.91 x $10^{–4}$
3.30 x $10^{–3}$	5.55 x $10^{–2}$	1.00 x $10^{–1}$	8.52 x $10^{–2}$	1.48 x $10^{–2}$	9.30 x $10^{–1}$	8.06 x $10^{–4}$
3.30 x $10^{–3}$	7.77 x $10^{–2}$	1.00 x $10^{–1}$	8.52 x $10^{–2}$	1.48 x $10^{–2}$	9.07 x $10^{–1}$	1.06 x $10^{–3}$
3.30 x $10^{–3}$	1.00 x $10^{–1}$	1.00 x $10^{–1}$	8.52 x $10^{–2}$	1.48 x $10^{–2}$	8.85 x $10^{–1}$	1.23 x $10^{–3}$

$k_{2'}$ = (9.73 ± 0.51) x $10^{–3}$ L $mol^{–1}$ $s^{–1}$

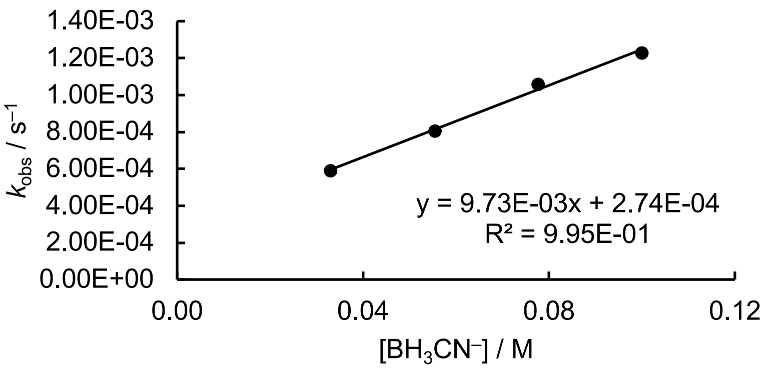

Kinetics of glycolate formation in the reaction of glyoxylate with $NaBH_3CN$ followed by ^1H NMR spectroscopy at 20.0 °C in pH 4 acetate solution (**$[buffer]_{total}$ = 0.233 M**) in H_2O (RM03-408). The ionic strength was maintained at I = 1 considering the concentration of $AcO^–$ and $NaBH_3CN$.

[**K2**] / M	[$NaBH_3CN$] / M	$[buffer]_{total}$ / M	[HOAc] / M	[$AcO^–$] / M	[NaCl] / M	k_{obs} / $s^{–1}$
3.30 x $10^{–3}$	3.30 x $10^{–2}$	2.33 x $10^{–1}$	1.99 x $10^{–1}$	3.45 x $10^{–2}$	9.33 x $10^{–1}$	8.39 x $10^{–4}$
3.30 x $10^{–3}$	5.55 x $10^{–2}$	2.33 x $10^{–1}$	1.99 x $10^{–1}$	3.45 x $10^{–2}$	9.10 x $10^{–1}$	1.19 x $10^{–3}$
3.30 x $10^{–3}$	7.77 x $10^{–2}$	2.33 x $10^{–1}$	1.99 x $10^{–1}$	3.45 x $10^{–2}$	8.88 x $10^{–1}$	1.57 x $10^{–3}$
3.30 x $10^{–3}$	1.00 x $10^{–1}$	2.33 x $10^{–1}$	1.99 x $10^{–1}$	3.45 x $10^{–2}$	8.66 x $10^{–1}$	1.82 x $10^{–3}$

$k_{2'}$ = (1.49 ± 0.09) x $10^{–2}$ L $mol^{–1}$ $s^{–1}$

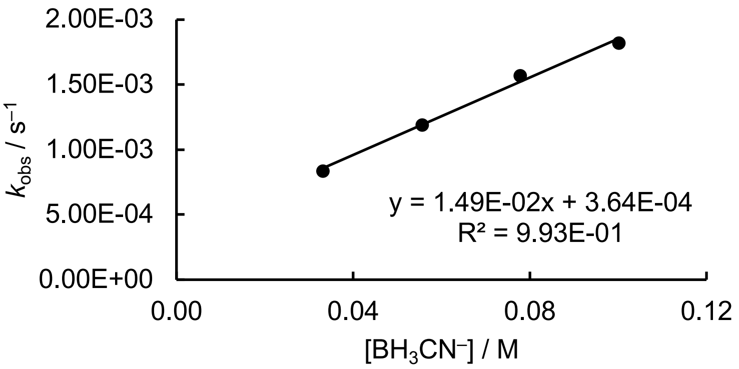

Kinetics of glycolate formation in the reaction of glyoxylate with $NaBH_3CN$ followed by ^1H NMR spectroscopy at 20.0 °C in pH 4 acetate solution (**$[buffer]_{total}$ = 0.366 M**) in H_2O (RM03-408). The ionic strength was maintained at I = 1 considering the concentration of AcO^- and $NaBH_3CN$.

[**K2**] / M	[$NaBH_3CN$] / M	$[buffer]_{total}$ / M	[HOAc] / M	[AcO^-] / M	[NaCl] / M	k_{obs} / s^{-1}
3.30 x 10^{-3}	3.30 x 10^{-2}	3.66 x 10^{-1}	3.12 x 10^{-1}	5.42 x 10^{-2}	9.13 x 10^{-1}	1.03 x 10^{-3}
3.30 x 10^{-3}	5.55 x 10^{-2}	3.66 x 10^{-1}	3.12 x 10^{-1}	5.42 x 10^{-2}	8.90 x 10^{-1}	1.56 x 10^{-3}
3.30 x 10^{-3}	7.77 x 10^{-2}	3.66 x 10^{-1}	3.12 x 10^{-1}	5.42 x 10^{-2}	8.68 x 10^{-1}	2.05 x 10^{-3}
3.30 x 10^{-3}	1.00 x 10^{-1}	3.66 x 10^{-1}	3.12 x 10^{-1}	5.42 x 10^{-2}	8.46 x 10^{-1}	2.57 x 10^{-3}

$k_{2'}$ = (2.29 ± 0.02) x 10^{-2} L mol^{-1} s^{-1}

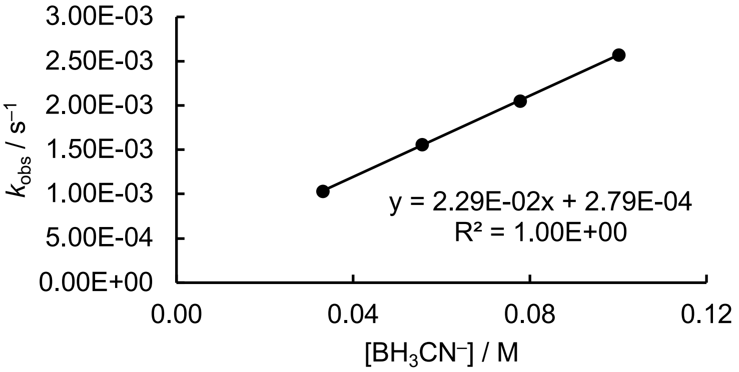

Kinetics of glycolate formation in the reaction of glyoxylate with $NaBH_3CN$ followed by ^1H NMR spectroscopy at 20.0 °C in pH 4 acetate solution (**$[buffer]_{total}$ = 0.500 M**) in H_2O (RM03-408). The ionic strength was maintained at I = 1 considering the concentration of AcO^- and $NaBH_3CN$.

[**K2**] / M	[$NaBH_3CN$] / M	$[buffer]_{total}$ / M	[HOAc] / M	[AcO^-] / M	[NaCl] / M	k_{obs} / s^{-1}
3.30 x 10^{-3}	3.30 x 10^{-2}	5.00 x 10^{-1}	4.26 x 10^{-1}	7.40 x 10^{-2}	8.93 x 10^{-1}	1.32 x 10^{-3}
3.30 x 10^{-3}	5.55 x 10^{-2}	5.00 x 10^{-1}	4.26 x 10^{-1}	7.40 x 10^{-2}	8.70 x 10^{-1}	1.98 x 10^{-3}
3.30 x 10^{-3}	7.77 x 10^{-2}	5.00 x 10^{-1}	4.26 x 10^{-1}	7.40 x 10^{-2}	8.48 x 10^{-1}	2.70 x 10^{-3}
3.30 x 10^{-3}	1.00 x 10^{-1}	5.00 x 10^{-1}	4.26 x 10^{-1}	7.40 x 10^{-2}	8.26 x 10^{-1}	3.02 x 10^{-3}

$k_{2'}$ = (2.61 ± 0.28) x 10^{-2} L mol^{-1} s^{-1}

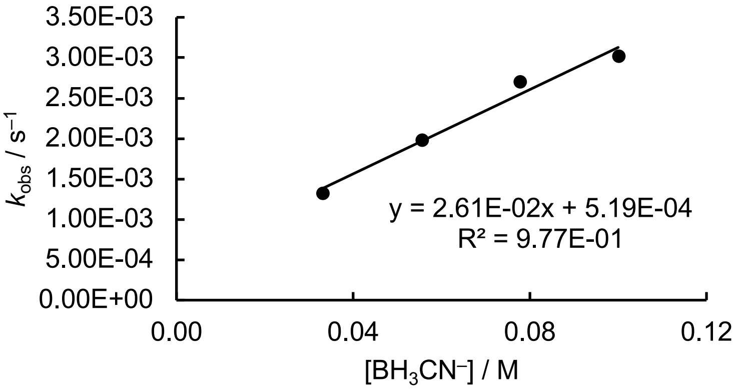

Correlation of $k_{2'}$ against the concentration of the buffer allows to determine the buffer-independent second-order rate constant for reduction, $k_{2,0}$ from the intercept.

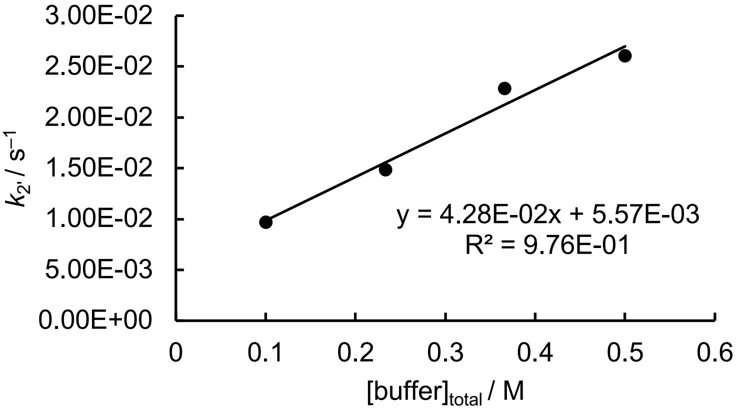

$k_{2,0}$ = (5.57 ± 0.16) x 10^{-3} L mol^{-1} s^{-1}

Data for the pH-Rate Profile for the Reaction of Glyoxylate (**K2**) with BH_3CN^-

Kinetics of glycolate formation in the reaction of glyoxylate with $NaBH_3CN$ (50 mM) followed by ^1H NMR spectroscopy at 20.0 °C in pH 3 formate buffer (0.1-0.5 M) in H_2O (RM03-416-pH3). The ionic strength was maintained at I = 1 considering the concentration of HCO_2^- and $NaBH_3CN$.

[**K1**] / M	[$NaBH_3CN$] / M	[buffer]$_{total}$ / M	[HCO_2H] / M	[HCO_2^-] / M	[NaCl] / M	k_{obs} / s^{-1}
5.00×10^{-3}	5.00×10^{-2}	1.00×10^{-1}	8.48×10^{-2}	1.52×10^{-2}	9.35×10^{-1}	1.80×10^{-3}
5.00×10^{-3}	5.00×10^{-2}	2.33×10^{-1}	1.97×10^{-1}	3.55×10^{-2}	9.14×10^{-1}	2.19×10^{-3}
5.00×10^{-3}	5.00×10^{-2}	3.66×10^{-1}	3.10×10^{-1}	5.58×10^{-2}	8.94×10^{-1}	3.30×10^{-3}
5.00×10^{-3}	5.00×10^{-2}	5.00×10^{-1}	4.24×10^{-1}	7.62×10^{-2}	8.74×10^{-1}	3.66×10^{-3}

$k_0 = (1.23 \pm 0.26) \times 10^{-3}\ s^{-1}$

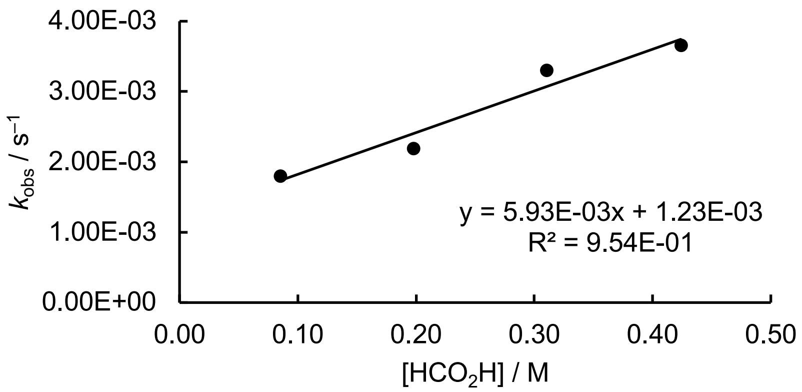

Kinetics of glycolate formation in the reaction of glyoxylate with $NaBH_3CN$ (50 mM) followed by ^1H NMR spectroscopy at 20.0 °C in pH 5 acetate buffer (0.1-0.5 M) in H_2O (RM03-445-pH5). The ionic strength was maintained at I = 1 considering the concentration of AcO^- and $NaBH_3CN$.

[**K1**] / M	[$NaBH_3CN$] / M	[buffer]$_{total}$ / M	[AcOH] / M	[AcO^-] / M	[NaCl] / M	k_{obs} / s^{-1}
5.00×10^{-3}	5.00×10^{-2}	1.00×10^{-1}	3.65×10^{-2}	6.35×10^{-2}	8.87×10^{-1}	4.49×10^{-4}
5.00×10^{-3}	5.00×10^{-2}	2.33×10^{-1}	8.51×10^{-2}	1.48×10^{-1}	8.02×10^{-1}	7.59×10^{-4}
5.00×10^{-3}	5.00×10^{-2}	3.66×10^{-1}	1.34×10^{-1}	2.32×10^{-1}	7.18×10^{-1}	9.70×10^{-4}
5.00×10^{-3}	5.00×10^{-2}	5.00×10^{-1}	1.83×10^{-1}	3.17×10^{-1}	6.33×10^{-1}	1.22×10^{-3}

$k_0 = (2.82 \pm 0.35) \times 10^{-4}\ s^{-1}$

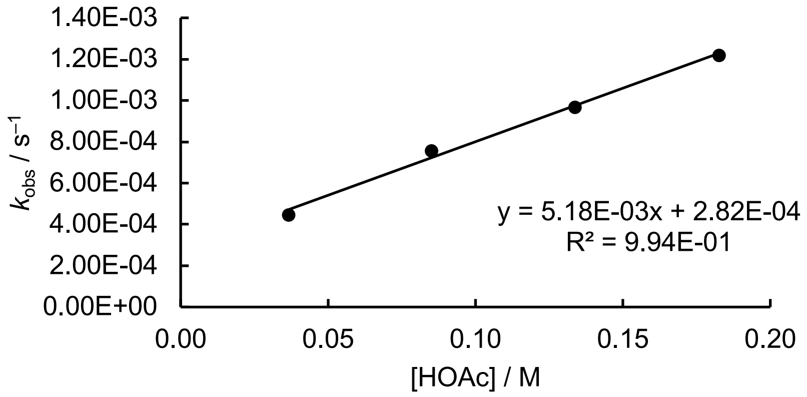

Kinetics of pyruvate disappearance in the reaction of pyruvate with $NaBH_3CN$ (50 mM) followed by ^1H NMR spectroscopy at 20.0 °C in pH 6 phosphate buffer (0.1-0.6 M) in H_2O (RM03-445-pH6). The ionic strength was maintained at I = 1 considering the concentration of HPO_4^{2-}, $H_2PO_4^-$ and $NaBH_3CN$.

[**K1**] / M	[$NaBH_3CN$] / M	[buffer]$_{total}$ / M	[$H_2PO_4^-$] / M	[NaCl] / M	k_{obs} / s^{-1}
5.00 × 10^{-3}	5.00 x 10^{-2}	1.00 x 10^{-1}	9.71 x 10^{-2}	8.29 x 10^{-1}	4.01 x 10^{-4}
5.00 × 10^{-3}	5.00 x 10^{-2}	2.33 x 10^{-1}	2.26 x 10^{-1}	6.69 x 10^{-1}	6.02 x 10^{-4}
5.00 × 10^{-3}	5.00 x 10^{-2}	3.66 x 10^{-1}	3.55 x 10^{-1}	5.09 x 10^{-1}	8.30 x 10^{-4}
5.00 × 10^{-3}	5.00 x 10^{-2}	5.00 x 10^{-1}	4.85 x 10^{-1}	3.47 x 10^{-1}	1.01 x 10^{-3}

k_0 = (2.49 ± 0.16) x 10^{-4} s^{-1}

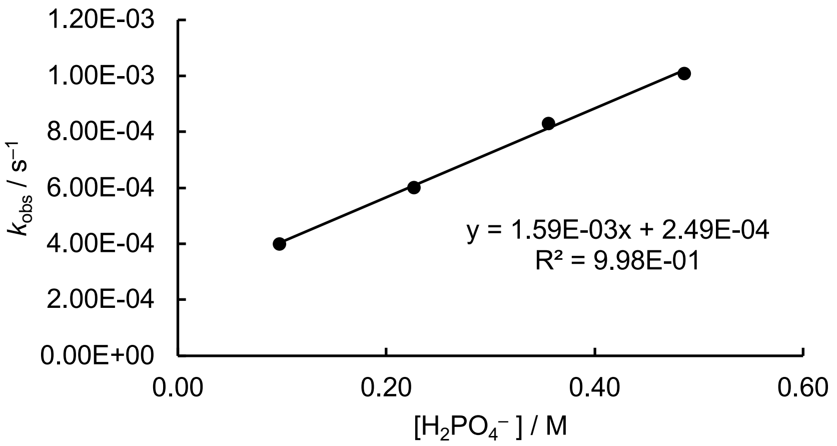

Kinetics of pyruvate disappearance in the reaction of pyruvate with $NaBH_3CN$ (50 mM) followed by ^1H NMR spectroscopy at 20.0 °C in pH 7 phosphate buffer (0.10-0.45 M) in H_2O (RM03-445-pH7). The ionic strength was maintained at I = 1 considering the concentration of HPO_4^{2-}, $H_2PO_4^-$ and $NaBH_3CN$.

[**K1**] / M	[$NaBH_3CN$] / M	[buffer]$_{total}$ / M	[$H_2PO_4^-$] / M	[NaCl] / M	k_{obs} / s^{-1}
5.00 x 10^{-2}	5.00 x 10^{-2}	5.00 x 10^{-2}	7.68 x 10^{-2}	7.43 x 10^{-1}	2.75 x 10^{-4}
5.00 x 10^{-2}	5.00 x 10^{-2}	5.00 x 10^{-2}	1.61 x 10^{-1}	5.15 x 10^{-1}	3.62 x 10^{-4}
5.00 x 10^{-2}	5.00 x 10^{-2}	5.00 x 10^{-2}	2.53 x 10^{-1}	2.67 x 10^{-1}	3.97 x 10^{-4}
5.00 x 10^{-2}	5.00 x 10^{-2}	5.00 x 10^{-2}	3.46 x 10^{-1}	1.80 x 10^{-2}	4.20 x 10^{-4}

k_0 = (2.55 ± 0.29) x 10^{-4} s^{-1}

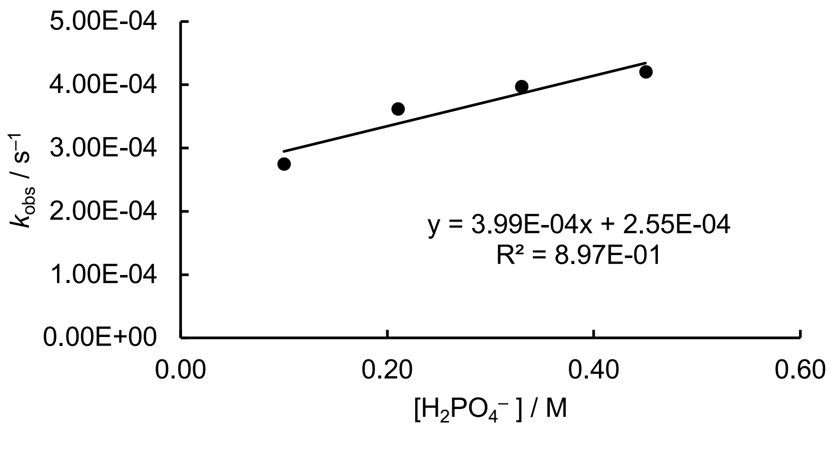

Table S2. Values used to construct the pH-rate profile for the reaction of **K2** with BH_3CN^- (50 mM) at zero-buffer concentration.

pH	k_0 / s^{-1}	lg k_0
3	(1.23 ± 0.26) x 10^{-3}	-2.91
4	4.92 x $10^{-4,a}$	-3.31
5	(2.82 ± 0.35) x 10^{-4}	-3.55
6	(2.49 ± 0.16) x 10^{-4}	-3.60
7	(2.55 ± 0.29) x 10^{-4}	-3.59

[a] calculated from the correlations of the buffer-dependent kinetics of **K2** with BH_3CN^- (see section above)

Analysis of the pH-rate profile

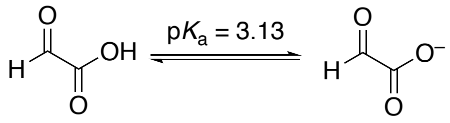

The pH-rate profile was analyzed with the relation:

$$k_0 = k_1[H^+] + k_w + \frac{k_2[H^+]}{K_a + [H^+]}$$

Consideration of the protonation equilibrium of glyoxylate was necessary to account for the S-shape of the curve. However, the value of k_1 could not be accurately determined due to the lack of data in the more acidic region. The analysis gave values for k_2 = (1.77 ± 0.43) x 10^{-3} and k_w = (2.55 ± 0.08) x 10^{-4}.

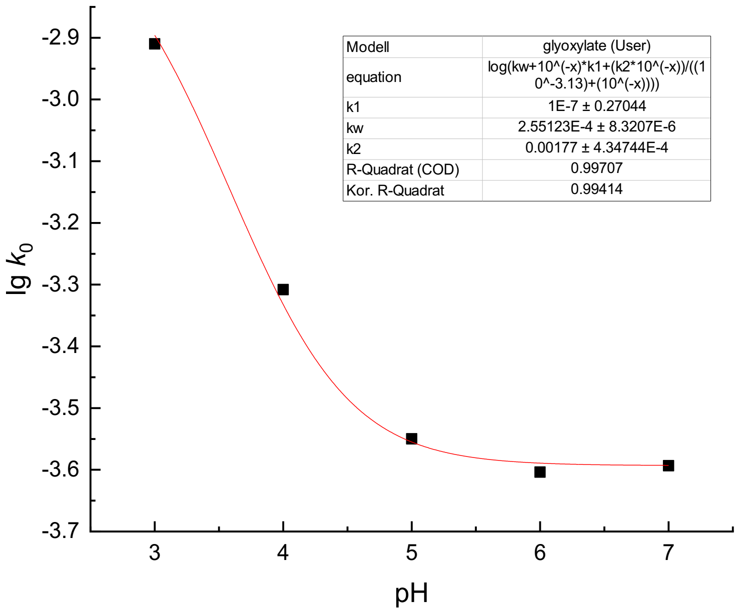

Figure S8. pH-rate profile for the reaction of **K2** with BH_3CN^- (50 mM). The pH-rate profile was analyzed with the relation $k_0 = k_1[H^+] + k_w + \frac{k_2[H^+]}{K_a+[H^+]}$. Non-linear regression afforded values for k_2 = (1.77 ± 0.43) x 10^{-3} and k_w = (2.55 ± 0.08) x 10^{-4}.

Kinetics of the Reaction of Oxaloacetate (**K3**) with BH_3CN^-

Kinetics of oxaloacetate disappearance in the reaction of oxaloacetate with $NaBH_3CN$ followed by UV/Vis spectroscopy (260 nm) at 20.0 °C in pH 4 acetate solution (0.1 M) in H_2O (RM03-410). The ionic strength was maintained at I = 1 considering the concentration of AcO^- and $NaBH_3CN$.

[**K3**] / M	[$NaBH_3CN$] / M	[buffer]$_{total}$ / M	[HOAc] / M	[AcO^-] / M	[NaCl] / M	k_{obs} / s^{-1}
3.00×10^{-3}	5.00×10^{-2}	1.00×10^{-1}	8.52×10^{-2}	1.48×10^{-2}	9.35×10^{-1}	7.34×10^{-3}
3.00×10^{-3}	6.66×10^{-2}	1.00×10^{-1}	8.52×10^{-2}	1.48×10^{-2}	9.19×10^{-1}	9.49×10^{-3}
3.00×10^{-3}	8.42×10^{-2}	1.00×10^{-1}	8.52×10^{-2}	1.48×10^{-2}	9.01×10^{-1}	1.27×10^{-2}
3.00×10^{-3}	1.00×10^{-1}	1.00×10^{-1}	8.52×10^{-2}	1.48×10^{-2}	8.85×10^{-1}	1.51×10^{-2}

$k_{2'} = k_{2,0} = (1.58 \pm 0.07) \times 10^{-3}$ L mol^{-1} s^{-1} (buffer independent, see experiment below)

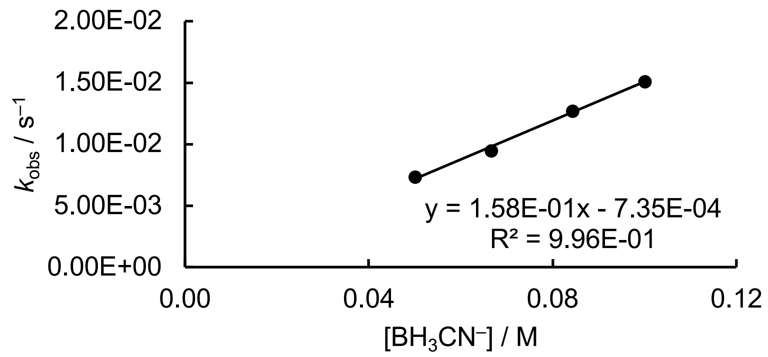

Buffer-dependency

Kinetics of oxaloacetate disappearance in the reaction of oxaloacetate with $NaBH_3CN$ followed by UV/Vis spectroscopy (260 nm) at 20.0 °C in pH 4 acetate solution (0.1-0.5 M) in H_2O (RM03-407). The ionic strength was maintained at I = 1 considering the concentration of AcO^- and $NaBH_3CN$.

[**K3**] / M	[$NaBH_3CN$] / M	[buffer]$_{total}$ / M	[HOAc] / M	[AcO^-] / M	[NaCl] / M	k_{obs} / s^{-1}
3.00×10^{-3}	5.00×10^{-2}	1.00×10^{-1}	8.52×10^{-2}	1.48×10^{-2}	9.35×10^{-1}	8.30×10^{-3}
3.00×10^{-3}	5.00×10^{-2}	2.50×10^{-1}	2.13×10^{-1}	3.70×10^{-2}	9.13×10^{-1}	8.15×10^{-3}
3.00×10^{-3}	5.00×10^{-2}	4.00×10^{-1}	3.41×10^{-1}	5.92×10^{-2}	8.91×10^{-1}	7.44×10^{-3}
3.00×10^{-3}	5.00×10^{-2}	5.00×10^{-1}	4.26×10^{-1}	7.40×10^{-2}	8.76×10^{-1}	7.29×10^{-3}

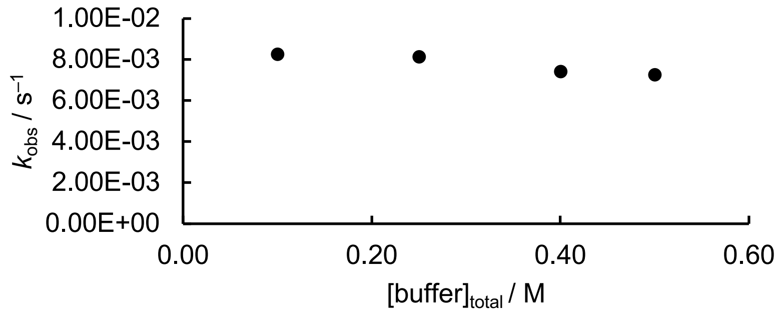

Experimentally, we reproducibly found a slight reduction of the reaction rate by approx. 10% when going from 0.1 to 0.5 M acetate buffer. The reason for this observation is unclear. Below a concentration of 0.25 M acetate buffer, the changes in rate were, however, not significant.

Data for the pH-Rate Profile for the Reaction of Oxaloacetate (K3) with BH_3CN^- (RM03-419)

The buffer-independency of the rate constants k_{obs} for the reaction of **K3** with BH_3CN^- was verified at pH 3, 4, 5 and 5.85. Accordingly, the k_{obs} values were used directly to construct the pH-rate profile as an extrapolation to a buffer concentration of zero was obsolete.
Note: Measurements at less acidic pH were hampered due to the long reaction time and the associated problems of competing decomposition of oxaloacetate.

Table S3. Values used to construct the pH-rate profile for the reaction of **K3** with BH_3CN^- (50 mM), 20 °C.

pH	buffer type	k_{obs} (= k_0)	lg k_{obs} = lg k_0
2.80	formate	1.79×10^{-2}	-1.75
3.01	formate	1.78×10^{-2}	-1.75
3.25	formate	1.72×10^{-2}	-1.76
3.44	formate	1.55×10^{-2}	-1.81
3.70	formate	1.26×10^{-2}	-1.90
3.90	formate	1.06×10^{-2}	-1.97
4.06	formate	8.79×10^{-3}	-2.06
4.26	formate	6.93×10^{-3}	-2.16
3.80	acetate	1.17×10^{-2}	-1.93
3.98	acetate	9.58×10^{-3}	-2.02
4.20	acetate	7.85×10^{-3}	-2.11
4.45	acetate	5.43×10^{-3}	-2.27
4.60	acetate	4.09×10^{-3}	-2.39
4.81	acetate	2.99×10^{-3}	-2.52
5.02	acetate	2.06×10^{-3}	-2.69
5.22	acetate	1.49×10^{-3}	-2.83
5.42	acetate	1.08×10^{-3}	-2.97
5.62	acetate	7.83×10^{-4}	-3.11
5.85	acetate	5.74×10^{-4}	-3.24

Analysis of the pH-rate profile

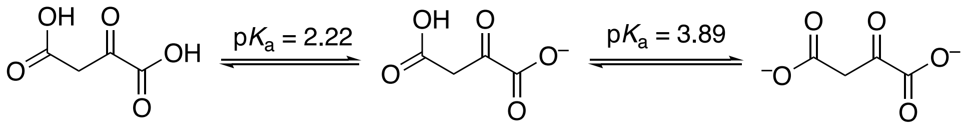

The pH-rate profile was analyzed with the relation:

$$k_0 = k_1[H^+] + k_w + \frac{k_2[H^+]}{K_a + [H^+]}$$

Non-linear fitting was only able to accurately fit the reaction of the species involved in the first protonation equilibrium (via pK_a = 3.89). Analysis gave values for k_2 = (2.13 ± 0.07) x 10^{-2} and k_w = (4.16 ± 0.35) x 10^{-5}.

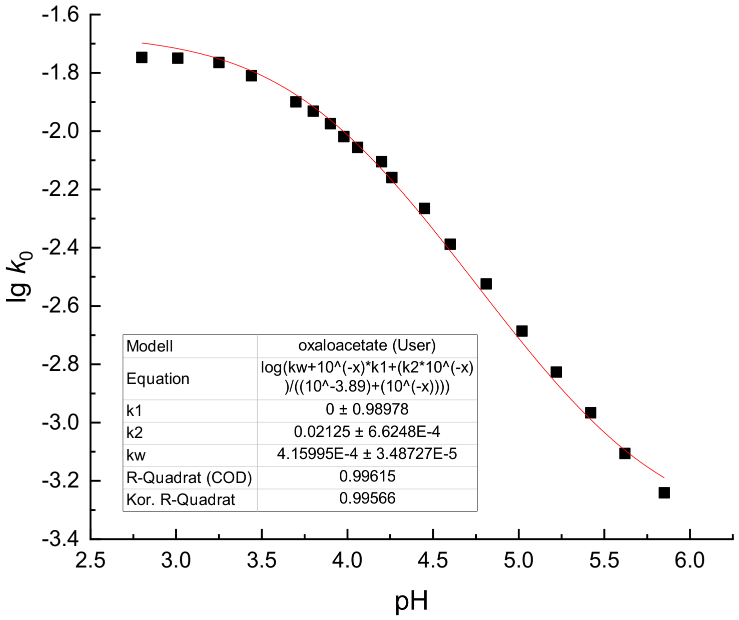

Figure S9. pH-rate profile for the reaction of **K3** with BH_3CN^- (50 mM). The pH-rate profile was analyzed with the relation $k_0 = k_1[H^+] + k_w + \frac{k_2[H^+]}{K_a+[H^+]}$. Non-linear regression afforded values for k_2 = (2.13 ± 0.07) x 10^{-2} and k_w = (4.16 ± 0.35) x 10^{-5}.

Calculation of the Effective Molarity

The effective molarity (EM) is a measure for the relative concentrations of the interactions of the functional groups within a molecue. It can be expressed as the ratio of the *intramolecular* rate compared to the *intermolecular* rate. The rate for the *intramolecular* acid-catalyzed reaction can be obtained from the plateau of the pH-rate profile as k = (2.13 ± 0.07) x 10^{-2} s^{-1}. On the other side, the rate of the *intermolecular* acid-catalyzed reaction of an acid catalyst with the same pK_a as oxaloacetate (3.89) can be calculated from the Brønsted plot in Figure S7. Accordingly, the effective molarity can be approximated as:

$$EM \approx \frac{2.13\ x\ 10^{-2}\ s^{-1}}{10^{-0.49*pK_a-0.57} L\ mol^{-1}\ s^{-1}} = \frac{2.13\ x\ 10^{-2}\ s^{-1}}{10^{-0.49*3.89-0.57} L\ mol^{-1}\ s^{-1}} = 6.4\ mol\ L^{-1}$$

Kinetics of the Reaction of α-Ketoglutarate (**K4**) with BH_3CN^-

Kinetics of α-ketoglutarate disappearance in the reaction of α-ketoglutarate with $NaBH_3CN$ followed by UV/Vis spectroscopy (320 nm) at 20.0 °C in pH 4 acetate solution (0.1 M) in H_2O (RM03-411). The ionic strength was maintained at I = 1 considering the concentration of AcO^- and $NaBH_3CN$.

[**K3**] / M	[$NaBH_3CN$] / M	[buffer]$_{total}$ / M	[HOAc] / M	[AcO^-] / M	[NaCl] / M	k_{obs} / s^{-1}
5.00 x 10^{-3}	5.00 x 10^{-2}	1.00 x 10^{-1}	8.52 x 10^{-2}	1.48 x 10^{-2}	9.35 x 10^{-1}	1.94 x 10^{-3}
5.00 x 10^{-3}	6.66 x 10^{-2}	1.00 x 10^{-1}	8.52 x 10^{-2}	1.48 x 10^{-2}	9.19 x 10^{-1}	2.55 x 10^{-3}
5.00 x 10^{-3}	8.42 x 10^{-2}	1.00 x 10^{-1}	8.52 x 10^{-2}	1.48 x 10^{-2}	9.01 x 10^{-1}	3.23 x 10^{-3}
5.00 x 10^{-3}	1.00 x 10^{-1}	1.00 x 10^{-1}	8.52 x 10^{-2}	1.48 x 10^{-2}	8.85 x 10^{-1}	3.91 x 10^{-3}

$k_{2'} = k_{2,0}$ = (3.93 ± 0.10) x 10^{-2} L mol^{-1} s^{-1} (buffer independent, see experiment below)

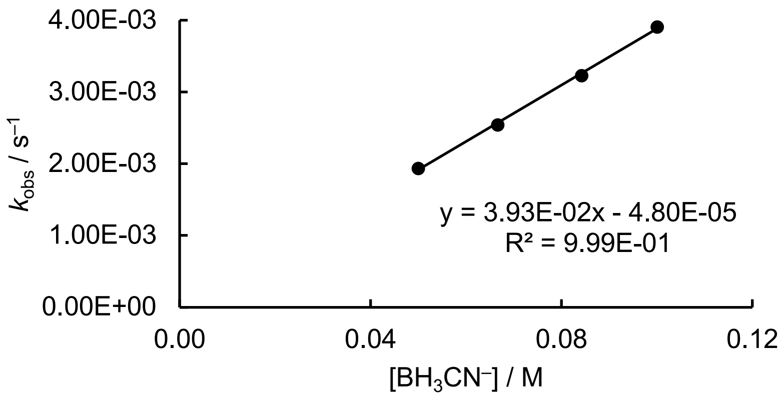

Buffer-dependency

Kinetics of α-ketoglutarate disappearance in the reaction of α-ketoglutarate with $NaBH_3CN$ followed by UV/Vis spectroscopy (320 nm) at 20.0 °C in pH 4 acetate solution (0.10-0.55 M) in H_2O (RM03-412). The ionic strength was maintained at I = 1 considering the concentration of AcO^- and $NaBH_3CN$.

[**K3**] / M	[$NaBH_3CN$] / M	[buffer]$_{total}$ / M	[HOAc] / M	[AcO^-] / M	[NaCl] / M	k_{obs} / s^{-1}
5.00 x 10^{-3}	5.00 x 10^{-2}	1.00 x 10^{-1}	8.52 x 10^{-2}	1.48 x 10^{-2}	9.35 x 10^{-1}	2.05 x 10^{-3}
5.00 x 10^{-3}	5.00 x 10^{-2}	2.50 x 10^{-1}	2.13 x 10^{-1}	3.70 x 10^{-2}	9.13 x 10^{-1}	1.92 x 10^{-3}
5.00 x 10^{-3}	5.00 x 10^{-2}	4.00 x 10^{-1}	3.41 x 10^{-1}	5.92 x 10^{-2}	8.91 x 10^{-1}	2.04 x 10^{-3}
5.00 x 10^{-3}	5.00 x 10^{-2}	5.50 x 10^{-1}	4.69 x 10^{-1}	8.14 x 10^{-2}	8.69 x 10^{-1}	2.22 x 10^{-3}

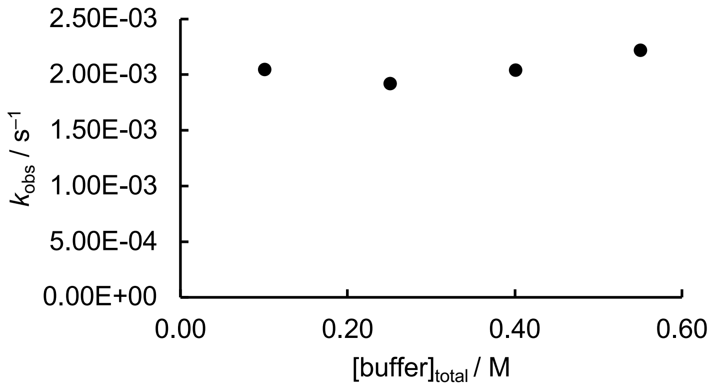

Data for the pH-Rate Profile for the Reaction of α-Ketoglutarate (**K4**) with BH_3CN^- (RM03-448/454)

The buffer-independency of the rate constants k_{obs} for the reaction of **K4** with BH_3CN^- was verified at pH 4 and 6. Accordingly, the k_{obs} values were used directly to construct the pH-rate profile as an extrapolation to a buffer concentration of zero was obsolete.

Table S4. Values used to construct the pH-rate profile for the reaction of **K4** with BH_3CN^- (50 mM), 20 °C.

pH	buffer type	k_{obs} (= k_0)	lg k_{obs} = lg k_0
2.80	formate	4.50×10^{-3}	-2.35
3.25	formate	4.52×10^{-3}	-2.34
3.70	formate	3.67×10^{-3}	-2.44
3.98	acetate	3.42×10^{-3}	-2.47
4.20	acetate	3.44×10^{-3}	-2.46
4.45	acetate	3.36×10^{-3}	-2.47
4.81	acetate	1.85×10^{-3}	-2.73
5.22	acetate	1.28×10^{-3}	-2.89
5.62	acetate	5.98×10^{-4}	-3.22
6.00	phosphate	2.17×10^{-4}	-3.66
7.00	phosphate	4.99×10^{-5}	-4.30

Analysis of the pH-rate profile

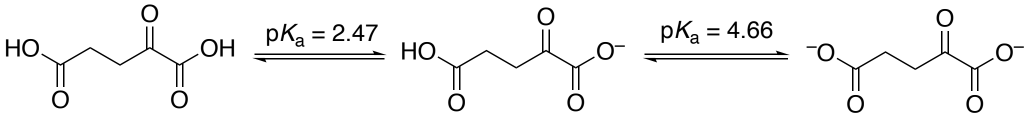

The pH-rate profile was analyzed with the relation:

$$k_0 = k_1[H^+] + k_w + \frac{k_2[H^+]}{K_a + [H^+]} + \frac{k_3[H^+]}{K_a + [H^+]}$$

Non-linear fitting was only able to accurately fit the reaction of the species involved in the first protonation equilibrium (via pK_a = 4.66). Analysis gave a value for $k_2 = (4.78 \pm 0.28) \times 10^{-3}$ and $k_w = (2.86 \pm 0.76) \times 10^{-5}$.

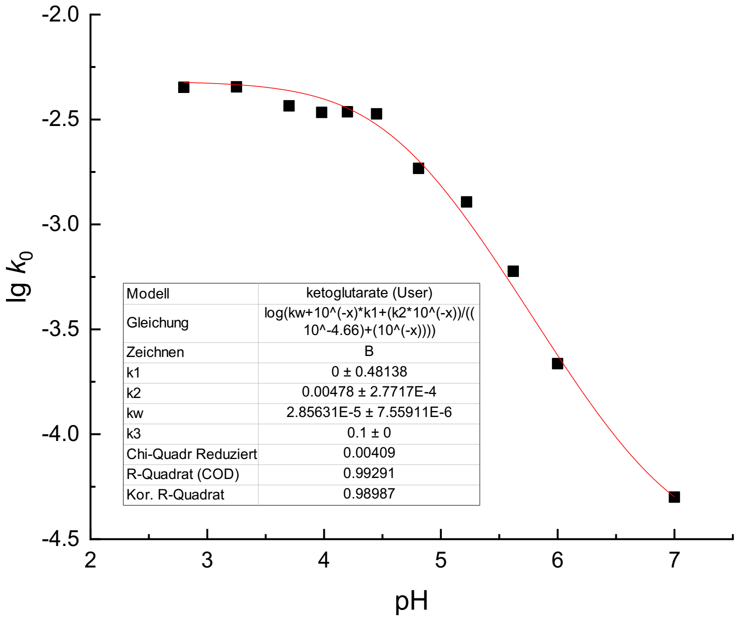

Figure S10. pH-rate profile for the reaction of **K4** with BH_3CN^- (50 mM). The pH-rate profile was analyzed with the relation $k_0 = k_1[H^+] + k_w + \frac{k_2[H^+]}{K_a+[H^+]} + \frac{k_3[H^+]}{K_a+[H^+]}$. Non-linear regression afforded values for k_2 = (4.78 ± 0.28) x 10^{-3} and k_w = (2.86 ± 0.76) x 10^{-5}.

Calculation of the Effective Molarity

The effective molarity (EM) is a measure for the relative concentrations of the interactions of the functional groups within a molecue. It can be expressed as the ratio of the *intramolecular* rate compared to the *intermolecular* rate. The rate for the *intramolecular* acid-catalyzed reaction can be obtained from the plateau of the pH-rate profile as k = (4.78 ± 0.28) x 10^{-3} s^{-1}. On the other side, the rate of the *intermolecular* acid-catalyzed reaction of an acid catalyst with the same pK_a as α-ketoglutarate (4.66) can be calculated from the Brønsted plot in Figure S7. Accordingly, the effective molarity can be approximated as:

$$EM \approx \frac{4.78\ x\ 10^{-3} s^{-1}}{10^{-0.49*pK_a-0.57} L\ mol^{-1}\ s^{-1}} = \frac{4.78\ x\ 10^{-3} s^{-1}}{10^{-0.49*4.66-0.57} L\ mol^{-1}\ s^{-1}} = 3.4\ mol\ L^{-1}$$

pH-Dependency of Cyanohydrin Formation in the Reaction of Pyruvate (K1) with $NaBH_3CN$

1.5 mL plastic tubes were charged with a stock solution of dimethyl sulfone, phosphate buffer (of a specific pH), pyruvate and $NaBH_3CN$ according to the table below. The pH of the samples was checked and re-adjusted when necessary. The contents were transferred to NMR tubes and the reaction progress followed by ^{1}H NMR spectroscopy. As seen in Figure S11, the cyanohydrin of pyruvate is observed as by-product in less acidic pH. While the amount of cyanohydrin relative to lactate is small for **K1** = 10 mM at pH 4 (5%), it is higher at pH 7.

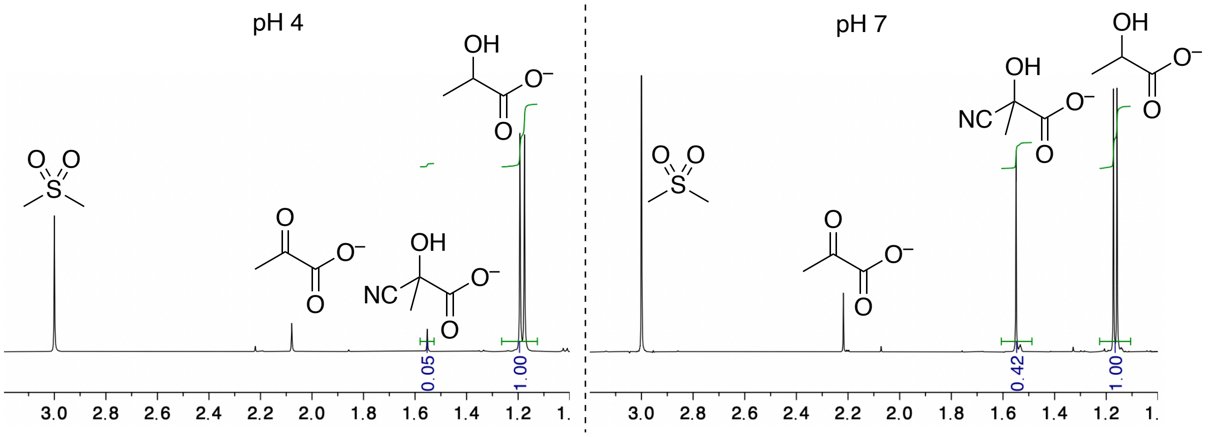

Figure S11. ^{1}H NMR spectra (noesygppr1d, 400 MHz, ns = 16, 23 °C) of the reaction of **K1** (10 mM) with $NaBH_3CN$ (100 mM) in pH 4 or pH 7 phosphate solution (0.5 M) in H_2O containing 8.3% D_2O (RM02-185/RM02-211).

When the formation of cyanohydrin becomes pronounced, it has an impact on the kinetics as the observed rates of disappearance of pyruvate will also reflect the rate of cyanohydrin formation.

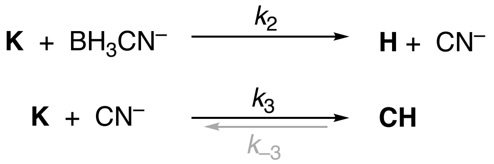

The observed decrease in the reaction rates with increasing pH will, accordingly, partially also reflect the more pronounced cyanohydrin formation at higher pH. To estimate the extent of this contribution, an approximated rate law can be derived (eq 1).

$$-\frac{d[\boldsymbol{K}]}{dt} = k_2[\boldsymbol{K}][BH_3CN^-] + k_3[\boldsymbol{K}][CN^-] \quad (1)$$

In the extreme case, the cyanohydrin formation can be assumed to be fast and irreversible relative to the reaction of pyruvate with BH_3CN^- (thus, the reverse reaction via k_{-3} is neglected). For such a situation, the steady-state approximation can be employed. Cyanide is formed in one reaction, and consumed by another.

$$\frac{d[CN]}{dt} = 0 \quad (2)$$

$$k_2[\boldsymbol{K}][BH_3CN] - k_3[\boldsymbol{K}][CN^-] = 0 \quad (3)$$

$$k_2[\boldsymbol{K}][BH_3CN] = k_3[\boldsymbol{K}][CN^-] \quad (4)$$

$$\frac{k_2}{k_3}[BH_3CN] = [CN^-] \quad (5)$$

$$-\frac{d[\boldsymbol{K}]}{dt} = k_2[\boldsymbol{K}][BH_3CN^-] + k_2[\boldsymbol{K}][BH_3CN] = 2k_2[\boldsymbol{K}][BH_3CN^-] \quad (6)$$

The observed rate constant for disappearance of pyruvate would, therefore, correspond to $2k_2$, thus, is a factor of two higher than expected. The reality somehow lies within the two extremes, as typically not a 1:1 but more often a 2:1 or 3:1 ratio of lactate : cyanohydrin was observed.

Accordingly, we performed our full kinetic analysis mostly at pH 4 to exclude any effects of the cyanohydrin side reaction. However, we did not correct the pH-rate profiles for the effect as a correction between factors 1-2 on the rate constants is relatively small given the large range covered by the pH-rate profiles of up to 3 orders of magnitude.

Exchange NMR Experiments of Hydrate Formation with Glyoxylate (K2)

Effect of pH on the Hydration Equilibrium

In water, glyoxylate (**K2**) is mostly present in the form of its hydrate. As the reduction of **K2** has a pronounced pH dependency, we set out to verify to which extent this is caused by a change on the hydration equilibrium.

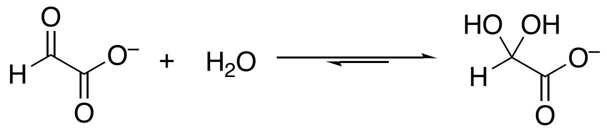

As shown in Figure S12, ^{1}H NMR analysis indicates that the ratio of glyoxylate to glyoxylate hydrate does not change significantly in the pH range from pH 4 to 7. However, the signals are noticeably broader at pH 7 compared to pH 4, suggesting a faster exchange between both species with increasing pH.

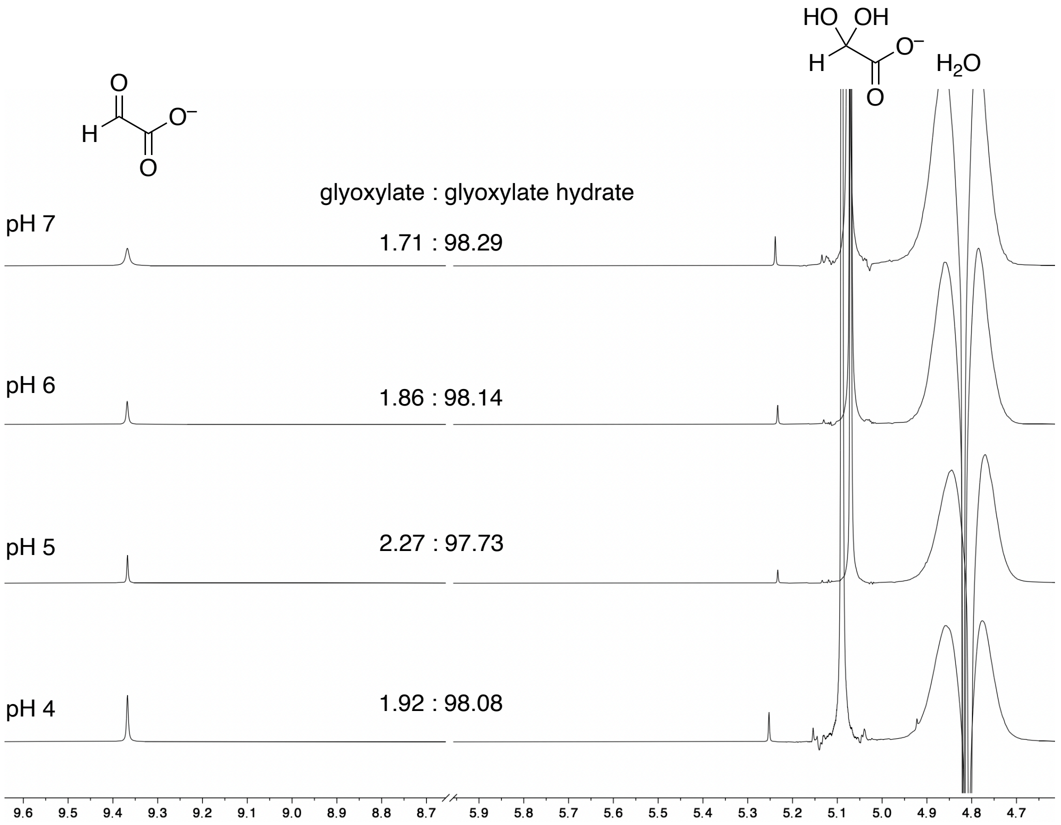

Figure S12. Quantitative ^{1}H NMR spectra (500 MHz, 8% D_2O in H_2O, d_1 = 50 s) of glyoxylate in 0.2 M acetate (pH 4 and 5) or phosphate (pH 6 and 7) buffers at 23 °C (RM03-446)

Measurement of the Exchange Rate (RM03-439)

Exchange measurement of glyoxylate were performed by EXSY NMR spectroscopy. A NMR tube was charged with glyoxylic acid monohydrate (ca 10 mg) and 600 µL of 0.1 M acetate buffer in D_2O (buffer prepared from AcOD in D_2O adjusted with a pH meter to a pH reading of 4.4 by addition of NaOD; corresponding approximately to a pD of 4.0). A second sample was prepared in an analogous way in phosphate buffer (prepared from D_3PO_4 in D_2O adjusted with a pH meter to a pH reading of 7.4 by addition of NaOD; corresponding approximately to a pD of 7.0). NMR spectra were recorded with the *noesyphpp* pulse sequence using mixing times (d8) of 0 ms and 800 ms. Space integrals of the 2D spectra were determined on identical chemical shift ranges with the Mnova software normalized to a non-exchanging impurity peak at 8.22 ppm. Exchange rates were calculated with the EXSYCalc software freely available from Mestrelab Research.

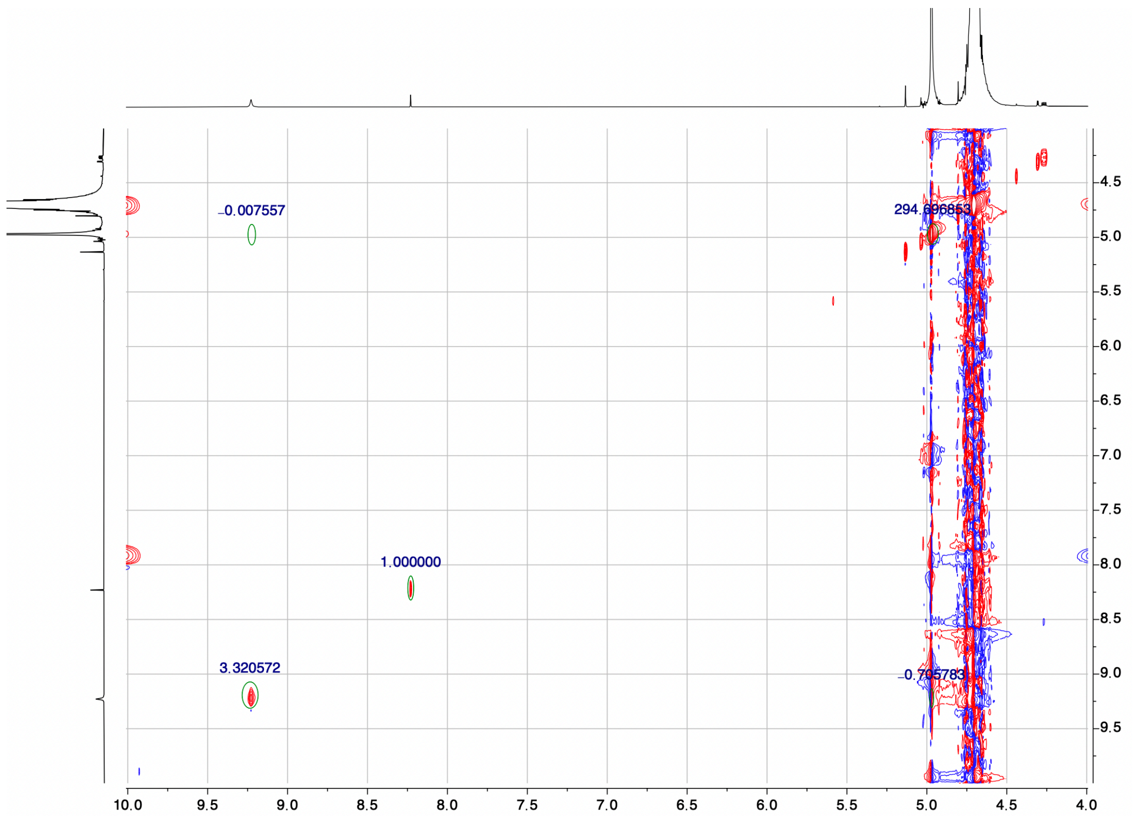

Figure S13. $^{1}H,^{1}H$ NOESY NMR (500 MHz, 0.1 M acetate buffer in D_2O, pD 4.4, 23 °C, mixing time d8: 0 ms) of **K2** and volume integrals relative to the one of a residual impurity at 8.22 ppm (set to 1.00).

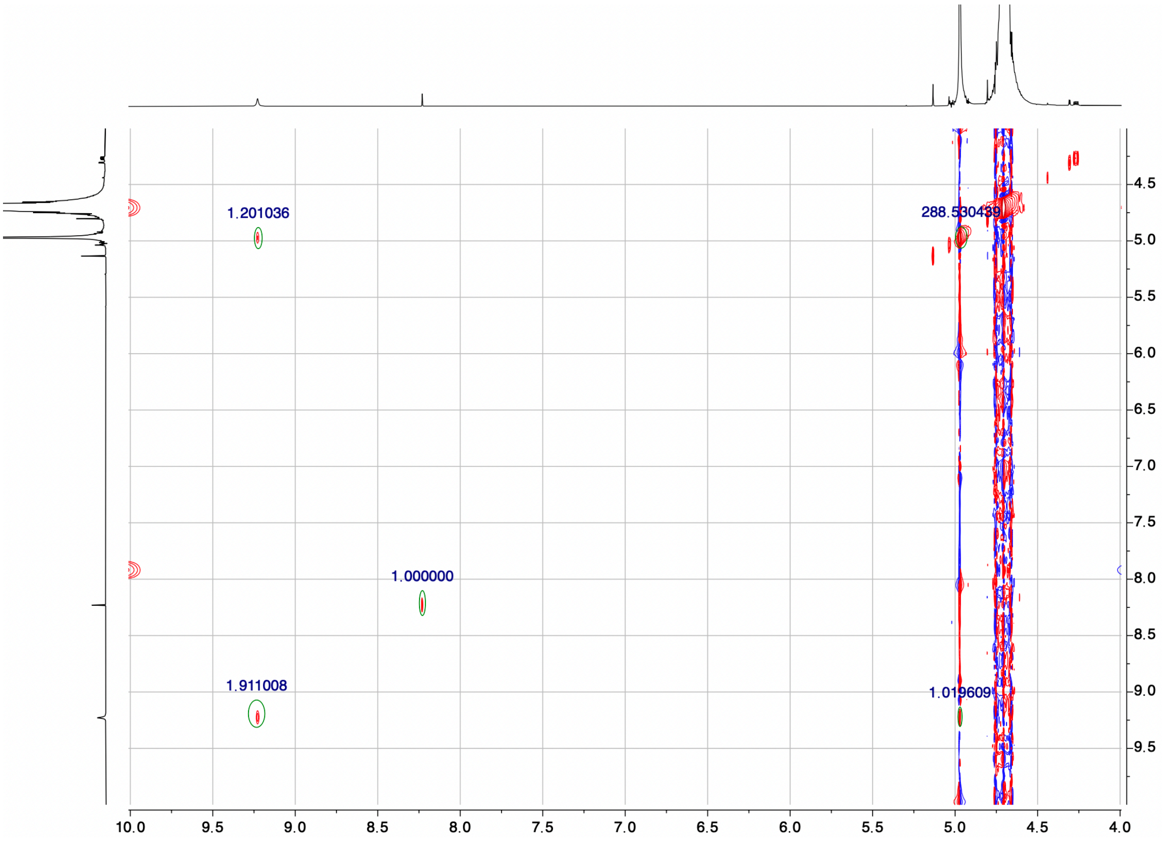

Figure S14. $^{1}H,^{1}H$ NOESY NMR (500 MHz, 0.1 M acetate buffer in D_2O, pD 4.4, 23 °C, mixing time d8: 800 ms) of **K2** and volume integrals relative to the one of a residual impurity at 8.22 ppm (set to 1.00).

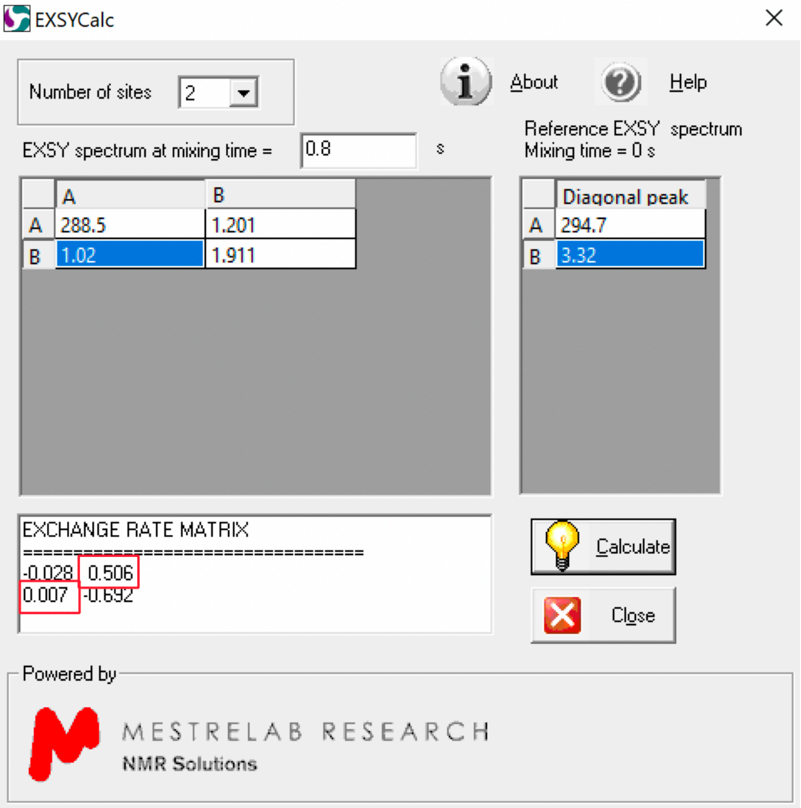

Figure S15. Output from the EXSYCalc software for the exchange rates (values in red boxes) calculated from the volume integrals from Figure S13 and S14.

The obtained value for the dehydration step of 0.007 s^{-1} is faster than then observed reaction rate in the reactions of **K2** with BH_3CN^- obtained in 0.1 M acetate buffer at pH 4 (0.00059-0.0012 s^{-1}). Accordingly, dehydration cannot be the rate-limiting step of the reaction.

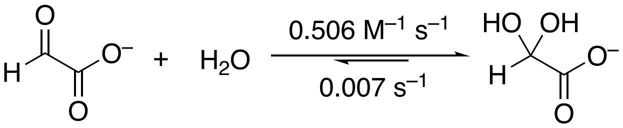

conditions: pD 4.4 acetate buffer, D_2O, 23 °C

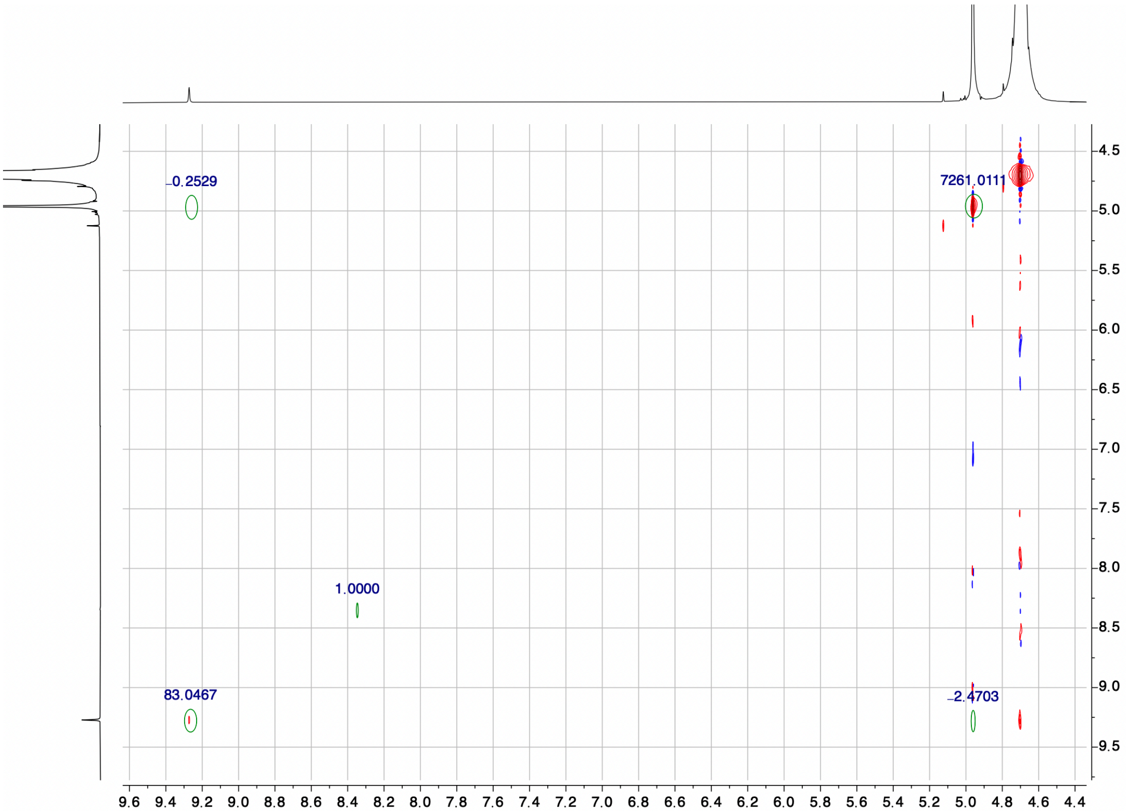

Figure S16. ^{1}H,^{1}H NOESY NMR (500 MHz, 0.1 M phosphate buffer in D_2O, pD 7.4, 23 °C, mixing time d8: 0 ms) of **K2** and volume integrals relative to the one of a residual impurity at 8.22 ppm (set to 1.00).

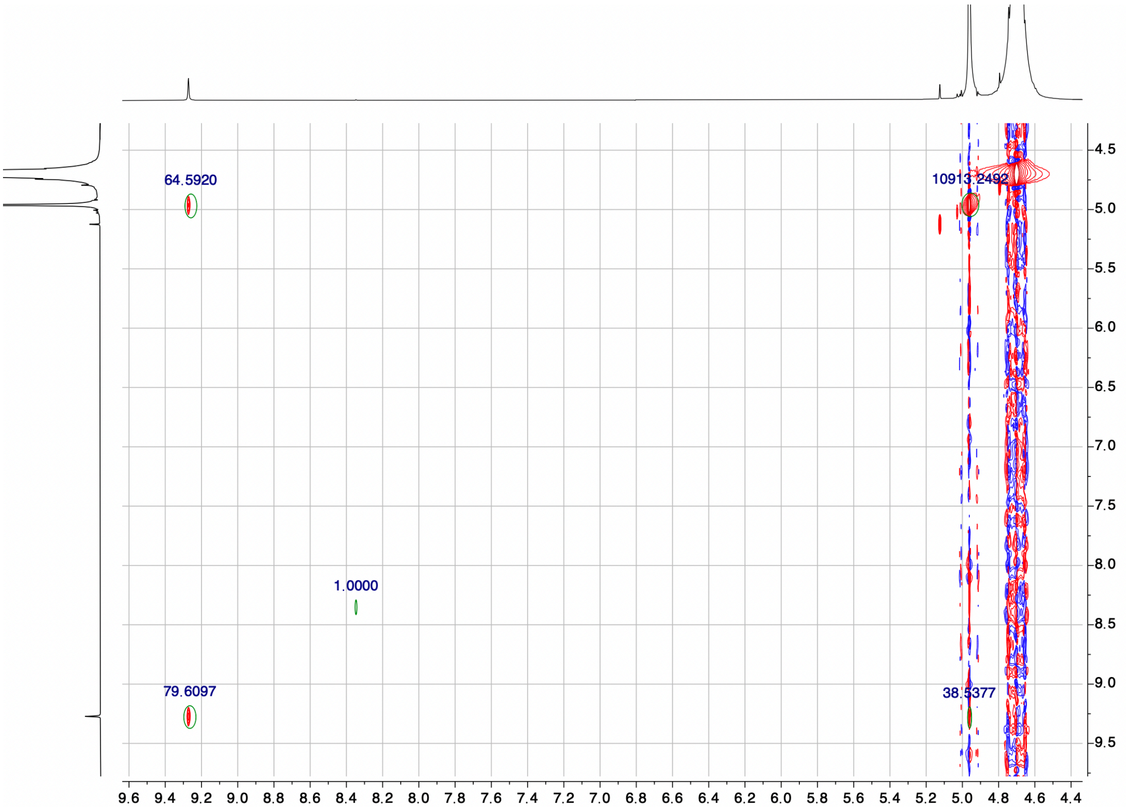

Figure S17. ^{1}H,^{1}H NOESY NMR (500 MHz, 0.1 M phosphate buffer in D_2O, pD 7.4, 23 °C, mixing time d8: 800 ms) of **K2** and volume integrals relative to the one of a residual impurity at 8.22 ppm (set to 1.00).

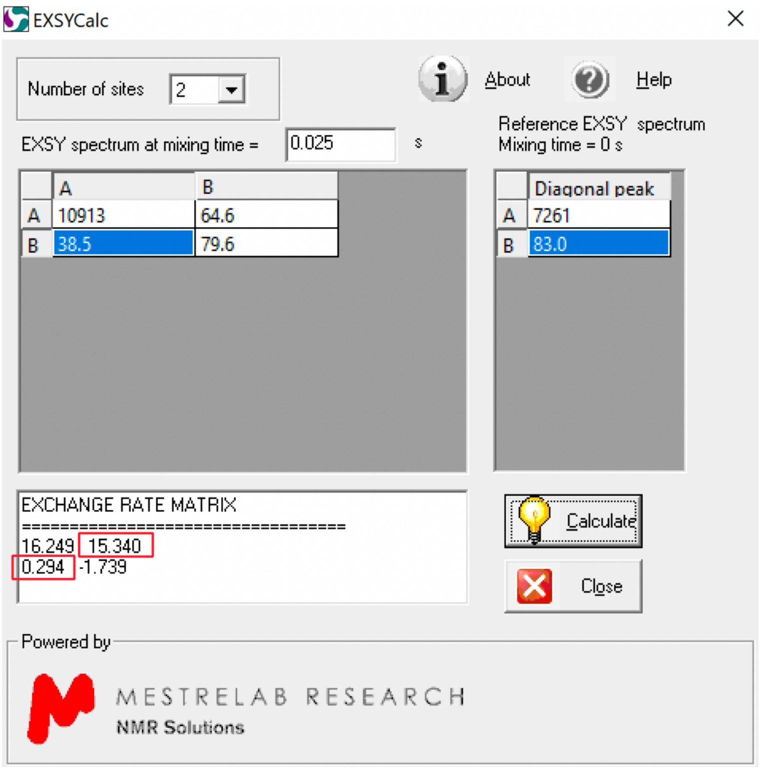

Figure S18. Output from the EXSYCalc software for the exchange rates (values in red boxes) calculated from the volume integrals from Figure S16 and S17.

As already indicated by the broadening of the ^{1}H NMR resonances of glyoxylate and glyoxylate hydrate at pH 7 compared to pH 4, exchange NMR analysis confirms the faster exchange rate at neutral pH.

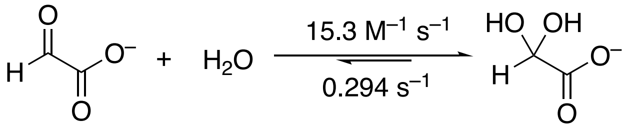

conditions: pD 7.4 phosphate buffer, D_2O, 23 °C

Competition Experiments for Keto Acid Reduction

Competition experiments were performed for the reaction of **K4**/**K1**, **K4**/**K2** and **K4**/**K3** with $NaBH_3CN$. The full procedure is exemplarily shown for the competition experiment between **K1**/**K4** with $NaBH_3CN$ and was analogously performed with the other two series of substrates.

Reaction mixture: A 5 mL glass vial was charged with α-ketoglutarate (disodium salt, 22.71 mg, 0.100 mmol) and sodium pyruvate (11.07 mg, 0.100 mmol). Water (1 mL) was added, and the contents of the vial mixed vigorously.

Reaction samples: NMR tubes were charged with the stock solution of keto acids (96 µL, resulting in ≈ 8 mM final concentration of each keto acid), buffer (300 µL from a 1 M stock solution; resulting in a final concentration of 500 mM), water (141 µL), a solution of DSS in D_2O of known concentration as integration standard (50 µL) and finally a stock solution of $NaBH_3CN$ (0.138 M, 13 µL) to give a total volume of 600 µL. The NMR tubes were sealed, mixed and kept in an air-conditioned laboratory (20-21 °C). ^1H NMR spectra were recorded after 30 min – 4 h (the reaction time is dependent on the pH). As buffers, 1 M stock solutions of acetate buffer (pH 4 and 5) as well as phosphate buffer (pH 6 and 7) were used.

Control sample: A separate NMR tube was charged with the stock solution of keto acids (96 µL, resulting in 8 mM final concentration of each keto acid), pH 7 phosphate buffer (from a 1 M stock solution, 30 µL), water (424 µL) and a solution of DSS in D_2O of known concentration as integration standard (50 µL).

NMR Analysis: Non-quantitative ^1H NMR spectra were recorded using a water-suppression sequence (noesygppr1d) using 64 scans on a 400 MHz instrument equipped with a nitrogen-cooled cryoprobe. For absolute quantification, the response factors of all analytes were separately determined from the integral ratios of the non-quantitative and quantitative NMR spectra where full relaxation was ensured by a sufficiently long delay time (30 s).

Determination of the competition constants from the NMR spectra – Full Description on the Reaction of K1/K4 at pH 4.

1. From the NMR sample without BH_3CN^-, the amount of the keto acids relative to the standard (integral set to 1.00) was determined.

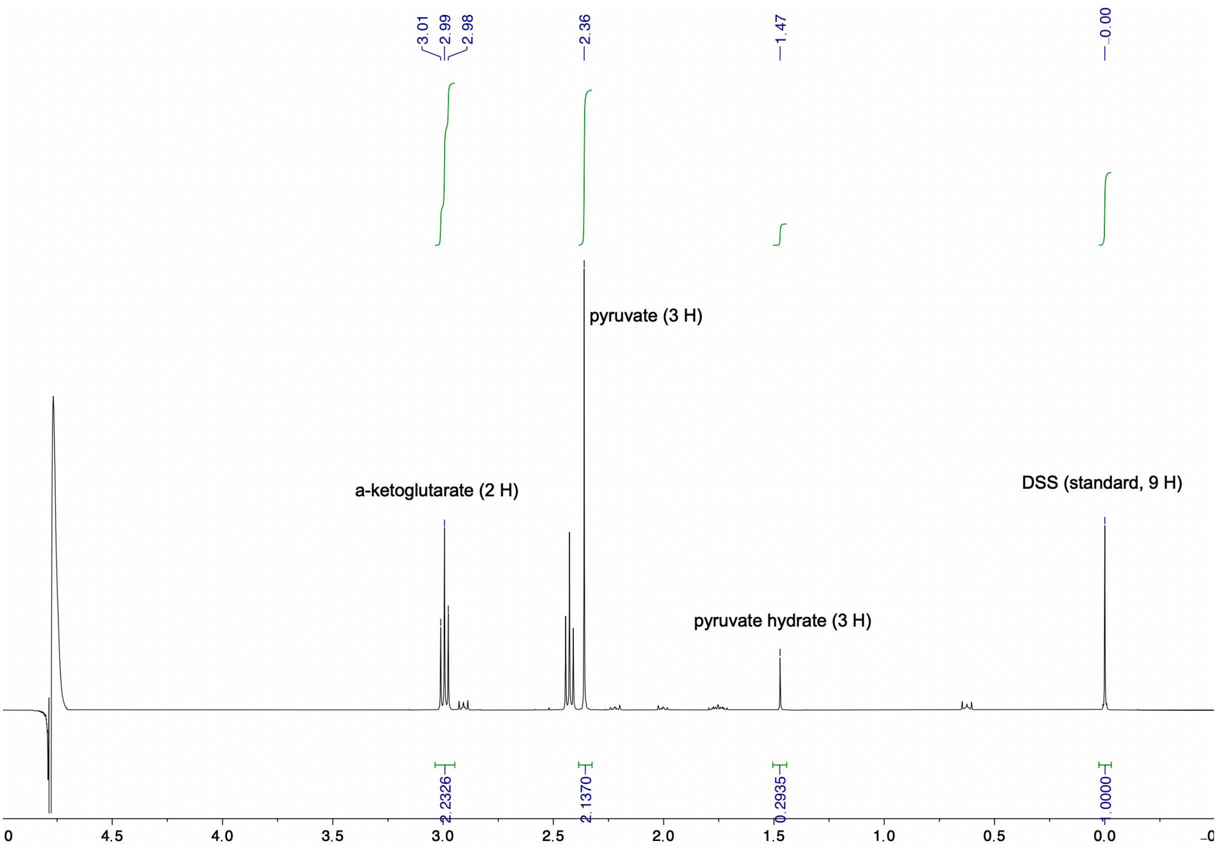

	**K1** (Pyruvate)	Pyruvate hydrate	**K4** (α-Ketoglutarate)
Corr. factor.[a]	1.437	0.806	0.818
Integral	2.137 (3 H)	0.2935 (3 H)	2.2326 (2 H)
Corr. integral[b]	3.081 (3 H)	0.237 (3 H)	1.826 (2 H)
Normalized	1.102 (1 H)[b]		0.913 (1 H)

[a] Correction factors from quantitative NMR spectroscopy. [b] calculated as product of the observed integral and the correction factor. [c] for pyruvate, the sum of the keto and hydrate form was taken.

2. For all samples at the specific pH values, accurate integration of the resonances of the remaining keto acids was not possible due to overlap with the resonances of the products. Additionally, by-products (e.g. due to cyanohydrin formation) or decomposition (e.g. decarboxylation of oxaloacetate) make using the actual ratio of keto acids after the reaction problematic. Instead, the formation of hydroxy acids **H** was analyzed which is accurately possible due to the well resolved CH(OH) resonances.

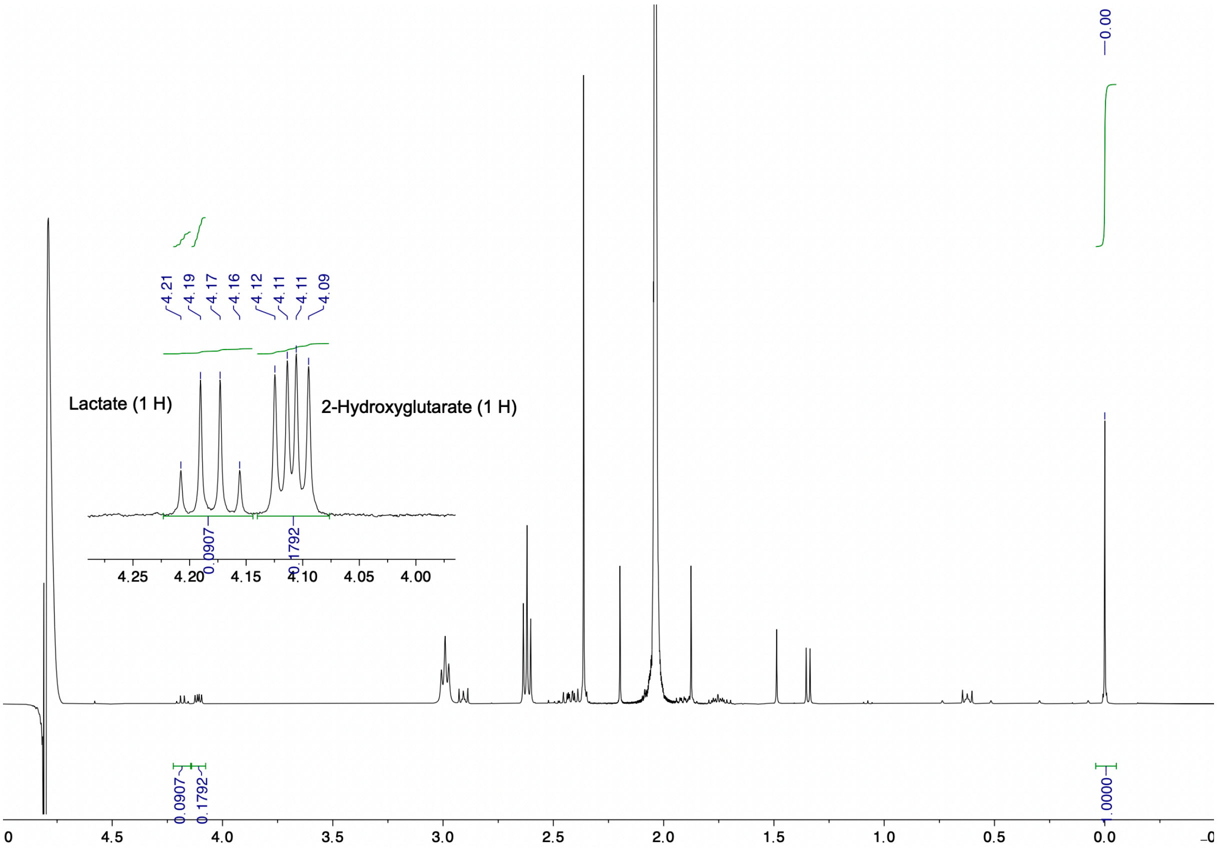

	Lactate (**H1**)	2-Hydroxyglutarate (**H4**)
Corr. factor.	1.23	0.996
Integral	0.0907 (1 H)	0.1792 (1 H)
Corr. integral	0.112 (1 H)	0.178 (1 H)

3. The amount of keto acids **K** after reaction $[\mathbf{K}]_t$ – which is *exclusively* due to reduction of the hydroxy acids – can now be calculated as $[\mathbf{K}]_t = [\mathbf{K}]_0 - [\mathbf{H}]_t$. Instead of concentrations, normalized integrals relative to the internal standard were used.

$[\mathbf{K}]_0$	$[\mathbf{K1}]_0 = 1.102$ (1 H)	$[\mathbf{K4}]_0 = 0.913$ (1 H)
$[\mathbf{H}]_t$	$[\mathbf{H1}]_t = 0.112$ (1 H)	$[\mathbf{H4}]_t = 0.178$ (1 H)
$[\mathbf{K}]_t = [\mathbf{K}]_0 - [\mathbf{H}]_t$	$[\mathbf{K1}]_t = 0.991$ (1 H)	$[\mathbf{K4}]_t = 0.735$ (1 H)

4. As the normalized integrals relative to the standard are directly proportional to concentrations, they can be directly substituted into the formula to analyze the competition experiments:[S6]

$$\kappa = \frac{k_2\,(\mathbf{K4})}{k_2\,(\mathbf{K1})} = \frac{\lg([\mathbf{K4}]_0/[\mathbf{K4}]_t)}{\lg([\mathbf{K1}]_0/[\mathbf{K1}]_t)} = \frac{\lg(0.913/0.735)}{\lg(1.102/0.991)} = 2.04$$

Table S5. Normalized and concentration-corrected integrals values before and after reaction for keto and hydroxy acids in the competition experiment of **K1**/**K4** with $NaBH_3CN$ used to calculate the pH-dependent competition constants. Buffer concentration: 0.5 M (for pH 4 and 5 acetate buffer was used; for pH 6 and 7 phosphate buffer). Experiment: RM03-422.

	pH	**K1**	**K4**	Lactate (**H1**)	2-Hydroxyglutarate (**H4**)	κ (**K4**/**K1**)
before reaction		1.102	0.913			
after reaction	4	0.991	0.735	0.112	0.178	**2.04**
	5	0.996	0.733	0.106	0.180	**2.17**
	6	1.066	0.862	0.036	0.051	**1.71**
	7	1.087	0.899	0.015	0.014	**1.10**

Table S6. Normalized and concentration-corrected integrals values before and after reaction for keto and hydroxy acids in the competition experiment of **K2**/**K4** with $NaBH_3CN$ used to calculate the pH-dependent competition constants. Buffer concentration: 0.5 M (for pH 4 and 5 acetate buffer was used; for pH 6 and 7 phosphate buffer). Experiment: RM03-425.

	pH	**K2**	**K4**	Glycolate (**K2**)	2-Hydroxyglutarate (**H4**)	κ (**K4**/**K2**)
before reaction		0.667	0.902			
after reaction	4	0.502	0.772	0.165	0.130	**0.550**
	5	0.483	0.767	0.184	0.136	**0.505**
	6	0.098	0.630	0.570	0.272	**0.186**
	7	0.114	0.807	0.554	0.095	**0.0628**

Table S7. Normalized and concentration-corrected integrals values before and after reaction for keto and hydroxy acids in the competition experiment of **K3**/**K4** with $NaBH_3CN$ used to calculate the pH-dependent competition constants. Buffer concentration: 0.5 M (for pH 4 and 5 acetate buffer was used; for pH 6 and 7 phosphate buffer). Experiment: RM03-420.

	pH	**K3**	**K4**	Malate (**K3**)	2-Hydroxyglutarate (**H4**)	κ (**K4**/**K3**)
before reaction		0.628	0.878			
after reaction	4	0.345	0.787	0.283	0.091	**0.183**
	5	0.384	0.759	0.244	0.119	**0.295**
	6	0.533	0.822	0.095	0.056	**0.403**
	7	0.608	0.865	0.020	0.013	**0.478**

Table S8. Relative rates (the reduction of **K4** was used as reference) calculated based on the competition constants κ from Tables S5-7 at pH4-7.[a]

	pH 4	pH 5	pH 6	pH 7
**K4**	1.00	1.00	1.00	1.00
**K1**	0.491	0.461	0.584	0.906
**K2**	1.82	1.98	5.37	15.9
**K3**	5.46	3.40	2.48	2.09

[a] e.g. κ (**K4**/**K1**) = 2.04. Accordingly, the rate for the formation of **K1** relative to **K4** is 1/κ = 1/2.04 = 0.491.

Reductive Amination

Equilibrium Constants for Imine Formation

Samples for the determination of K_I were prepared following the idea that when a solution of NH_4Cl is treated with NaOH as base, the ionic strength does not change due to the stoichiometric formation of NaCl.

Accordingly, 2 mL Eppendorf tubes were charged with stock solutions of the α-keto acid, KCl, NH_4Cl and water in a way that calculated for a total volume of 1 mL the concentration of keto acid = 30 mM and $[NH_4Cl] + [KCl] = 1.5$ M. NaOH was added from a stock solution to achieve the desired degree of deprotonation which was verified by pH monitoring (pH = 10.20). Lastly, dimethyl sulfone in D_2O (50 µL) and H_2O was added to achieve a total volume of 1 mL. 600 µL of each sample was transferred to NMR tubes and the concentration-dependent ^{1}H NMR shifts of the keto acid were analyzed.

The resulting dependency of the chemical shift difference Δδ (in ppm, referenced relative to the CH_3 group of dimethyl sulfone which was set to 3.00 ppm) of the resonance of the keto acid vs. the concentration of NH_3 was analyzed according to eq 8 by non-linear fitting with Origin software.[S7] For the detailed derivation of eq 8, see ref. S8. The only variable in eq 8 is K_I, all other quantities (concentration of NH_3 and **K**) are known from the concentrations used in the titrations.

$$\Delta\delta = \frac{\Delta\delta_{\max}}{[NH_3]}\frac{1}{2}\left\{\left([NH_3] + [\boldsymbol{K}] + \frac{1}{K_I}\right) - \sqrt{\left([NH_3] + [\boldsymbol{K}] + \frac{1}{K_I}\right)^2 + 4[NH_3][\boldsymbol{K}]}\right\} \quad (8)$$

The observed upfield shifts in the ^{1}H NMR titration experiments to determine the equilibrium constants of imine formation (Figure S19/20) could be due either to formation of an imine or a hemiaminal. Differentiating these two options experimentally is complicated due to the rather unfavorable position of the equilibrium. As shown in Figure S33, in-situ IR spectroscopy of **K1** was performed in concentrated ammonia solution (15 M, pH 10.2). The additional vibrations, besides those of **K1,** are in good agreement with those predicted based on a computed IR spectrum for the imine of pyruvate (Figure S33). Additionally, DFT computations of the relative energies indicated that for both pyruvate (**K1**) and α-ketoglutarate (**K4**), the hemiaminal is significantly disfavored compared to the imine (Figure S32). Taken together, the results from IR spectroscopy, computations and the polarographic studies of Zuman[S9] provide compelling evidence for the formation of imines in solution. Thus, we attributed the observed changes in NMR shifts to the formation of the imines and not that of the hemiaminals. Additionally, the binding isotherms at 23 °C provided the equilibrium constants for imine formation for both pyruvate ($K = 0.104 \pm 0.008$) and α-ketoglutarate ($K = 0.060 \pm 0.025$), the former of which is in reasonable agreement with the value reported for pyruvate at 0 °C from polarography ($K = 0.25$ M^{-1}). Analogous determination of the association constants with **K1** and **K3** were not successful. While **K3** is too unstable in aqueous solution, the hemiaminal and a triazine species were already described to dominate in aqueous solutions of glyoxylate and ammonia.[S10]

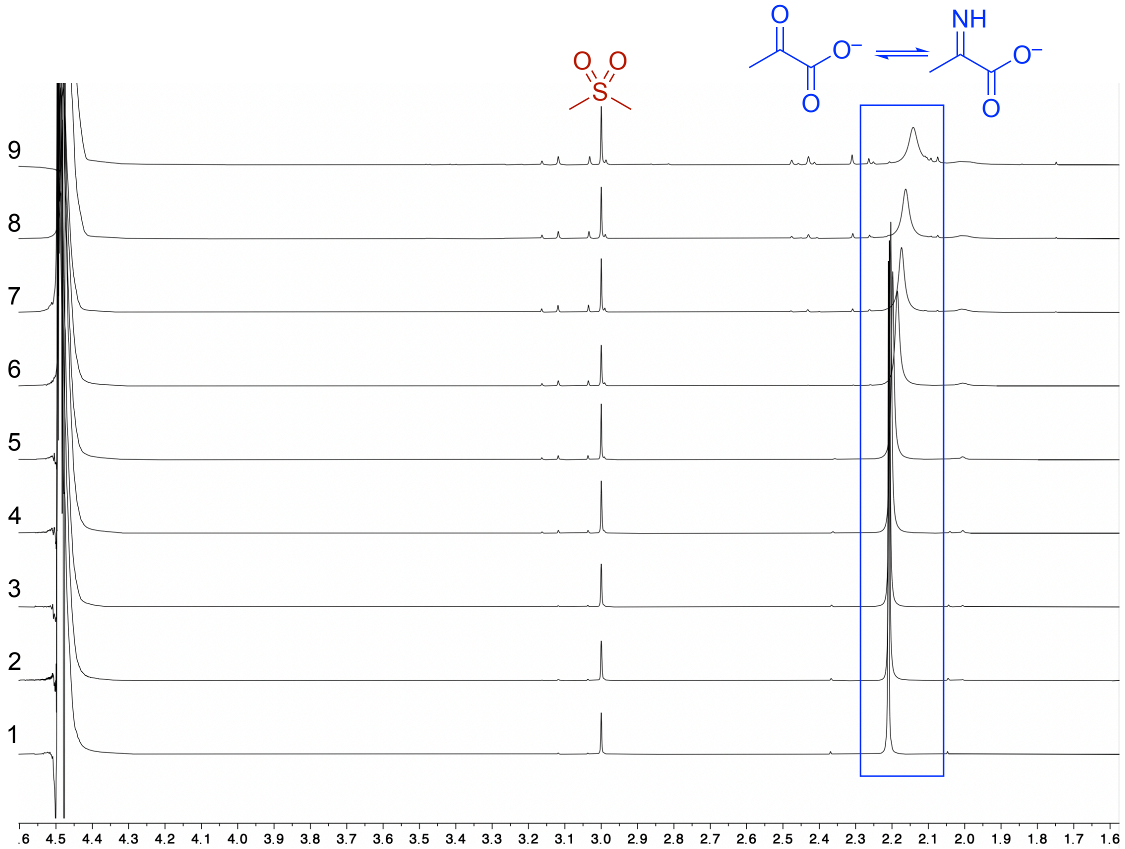

Figure S19. ^{1}H NMR spectra (noesygppr1d, 400 MHz, ns = 16, 23 °C) of the reaction of **K1** (30 mM) with different concentrations of NH_4Cl (increase from bottom to top) at pH 10.20 (RM01-142).

NMR titration for the equilibrium of pyruvate ([**K1**] = 30 mM) with NH_3 (RM01-142)

#	$[NH_3]$ / M	NMR shift δ / ppm	$\Delta\delta$ / ppm
1	0.00	2.2101	0
2	2.70×10^{-2}	2.2088	0.0013
3	5.40×10^{-2}	2.2072	0.0029
4	1.08×10^{-1}	2.2036	0.0065
5	2.16×10^{-1}	2.1982	0.0119
6	4.50×10^{-1}	2.1853	0.0248
7	6.74×10^{-1}	2.1738	0.0363
8	8.99×10^{-1}	2.1625	0.0476
9	1.35	2.1419	0.0682

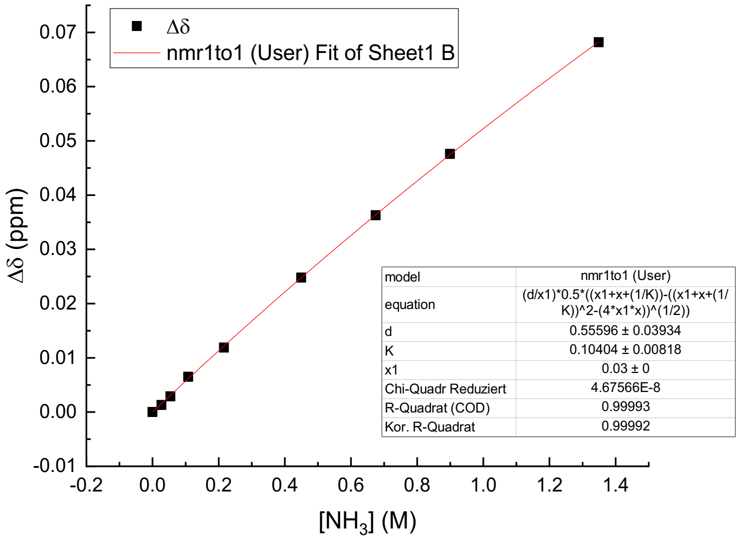

$K_I = 0.104 \pm 0.008$

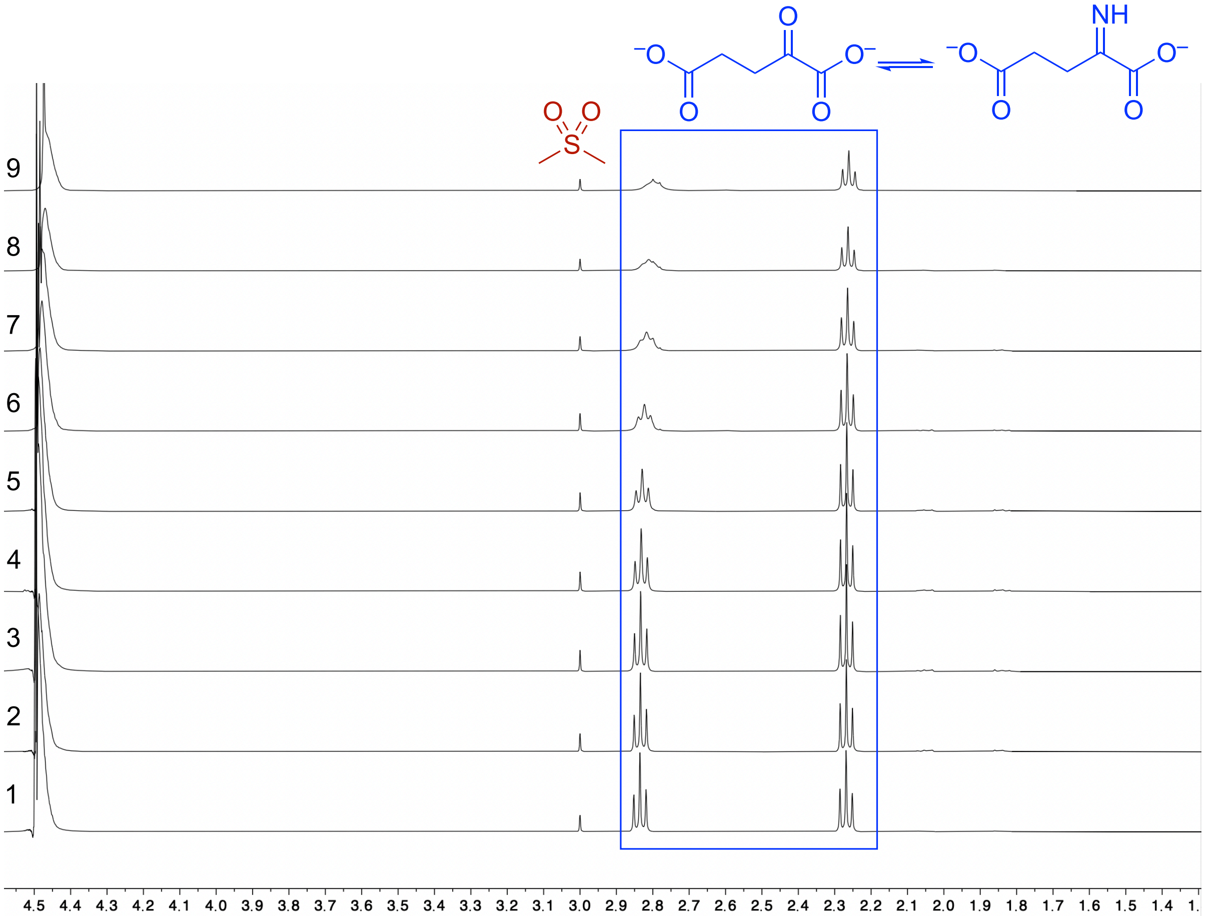

Figure S20. ^{1}H NMR spectra (noesygppr1d, 400 MHz, ns = 16, 23 °C) of the reaction of **K4** (30 mM) with different concentrations of NH_4Cl (increase from bottom to top) at pH 10.20 (RM01-141).

NMR titration for the equilibrium of α-ketoglutarate ([**K4**] = 30 mM) with NH_3 (RM01-141)

#	$[NH_3]$ / M	NMR shift δ / ppm	$\Delta\delta$ / ppm
1	0.00	2.8354	0.0000
2	2.70×10^{-2}	2.8344	0.0010
3	5.40×10^{-2}	2.8334	0.0020
4	1.08×10^{-1}	2.8318	0.0036
5	2.16×10^{-1}	2.8292	0.0062
6	4.50×10^{-1}	2.8229	0.0125
7	6.74×10^{-1}	2.8174	0.0180
8	8.99×10^{-1}	2.8107	0.0247
9	1.35	2.7997	0.0357

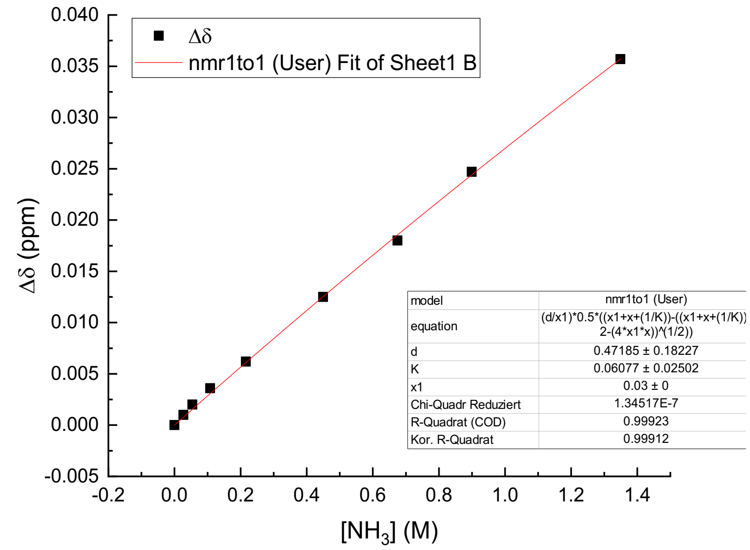

$K_I = 0.061 \pm 0.025$

Kinetics of Reductive Amination – Mechanistic Analysis

Species Distribution

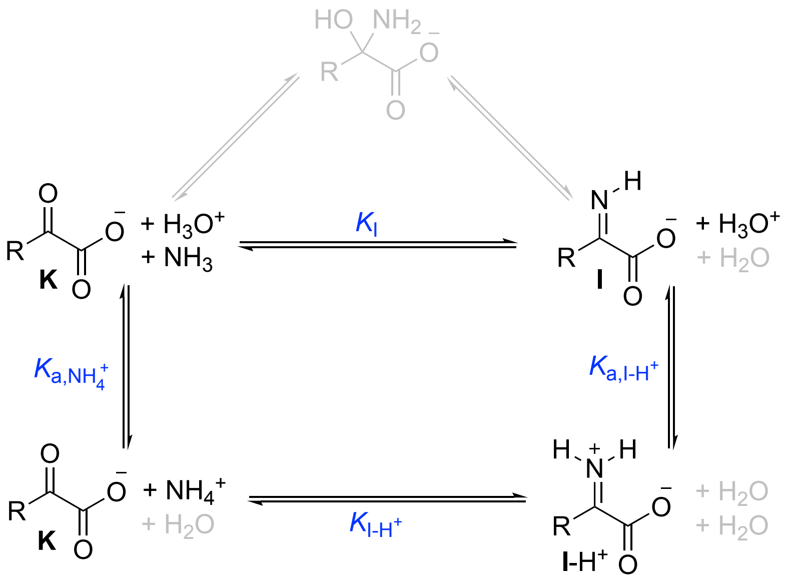

In the scheme above, the following equilibria can be defined:

$$K_{a,NH_4^+} = \frac{[H_3O^+][NH_3]}{[NH_4^+]} \quad (8)$$

$$K_{a,I-H^+} = \frac{[H_3O^+][I]}{[I-H^+]} \quad (9)$$

$$K_I = \frac{[I]}{[NH_3][K]} \quad (10)$$

$$K_{I-H^+} = \frac{[I-H^+]}{[NH_4^+][K]} \quad (11)$$

The equilibria (8) – (11) are related via the following expression:

$$K_{a,NH_4^+}\, K_I = K_{a,I-H^+}\, K_{I-H^+} \quad (12)$$

K_{a,NH_4^+} corresponds to the experimentally known acid dissociation constant for NH_4^+ ions, K_I is accessible by titration experiments (see the section above) and $K_{a,I-H^+}$ can be estimated with relatively high accuracy from experimentally-anchored DFT calculations (see the computational section). The species distribution (based on molar fractions) can be calculated for the system of equilibria (8) – (11) as shown on the example of **I**-H$^+$. The fraction of keto acid that is present as iminium ion **I**-H$^+$ in solution is given as:

$$x_{I-H^+} = \frac{[I-H^+]}{[I-H^+] + [I] + [K]} \quad (13)$$

Substitution of (9) and (10) into (12) gives the following expression:

$$x_{I-H^+} = \frac{\frac{[H_3O^+][I]}{K_{a,I-H^+}}}{\frac{[H_3O^+][I]}{K_{a,I-H^+}} + [I] + \frac{[I]}{K_I[NH_3]}} = \frac{\frac{[H_3O^+]}{K_{a,I-H^+}}}{\frac{[H_3O^+]}{K_{a,I-H^+}} + 1 + \frac{1}{K_I[NH_3]}} \quad (14)$$

The concentration of ammonia [NH_3] at a specific pH value (= concentration of H_3O^+) can be expressed from the Henderson–Hasselbalch equation as a function of the initial concentration of NH_4^+, [NH_4^+]$_0$, after some rearrangement:

$$[NH_3] = \frac{[NH_4^+]_0\, K_{a,NH_4^+}}{[H_3O^+] + K_{a,NH_4^+}} \quad (15)$$

Substitution of (15) into (14) yields an expression for x_{I-H^+}, the molar fraction of iminium ion **I**-H$^+$ for a specific pH and concentration of NH_4^+.

$$x_{I-H^+} = \frac{\dfrac{[H_3O^+]}{K_{a,I-H^+}}}{\dfrac{[H_3O^+]}{K_{a,I-H^+}} + 1 + \dfrac{[H_3O^+] + K_{a,NH_4^+}}{K_I [NH_4^+]_0\, K_{a,NH_4^+}}} \quad (16)$$

*Note: equation (16) will fail to correctly describe the amount of **I**-H$^+$ at more acid solution when protonation of pyruvate (pK_a = 2.49) becomes pronounced.*

Alternatively, the species distribution can be calculated from a simulation of the equilibria (8) – (11), now including the protonation equilibrium of pyruvic acid to pyruvate, with the COPASI package.[S11] Between pH 4 and 14, the results of the analysis with eq (16) and the COPASI package are virtually identical.

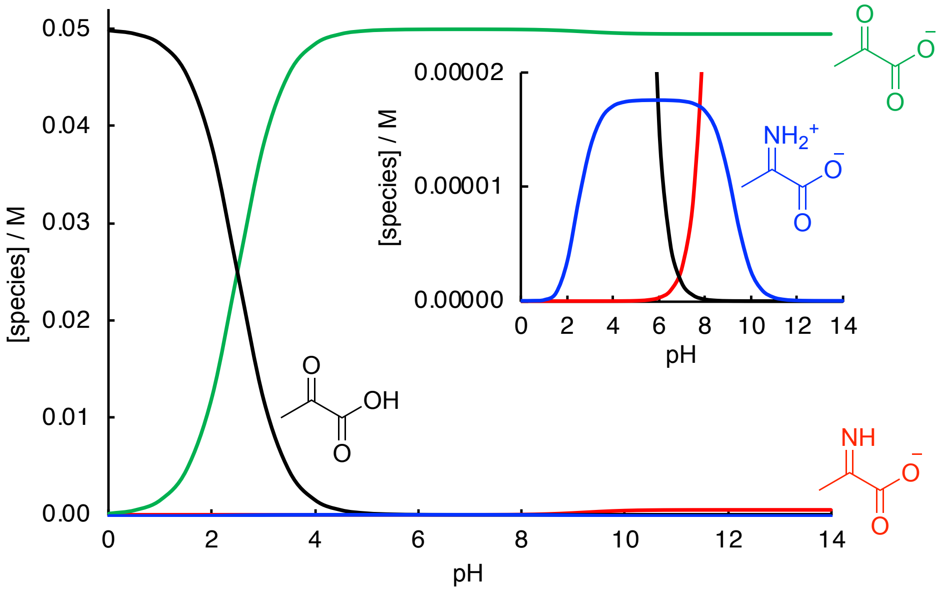

Figure S21. Species distribution for the system pyruvate (50 mM)/ NH_3 (100 mM) as a function of pH with K_I = 0.104, p$K_{a,NH4+}$ = 9.25, p$K_{a,\mathbf{I1}\text{-}H+}$ = 7.78, p$K_{a,\mathbf{K1}}$ = 2.49.

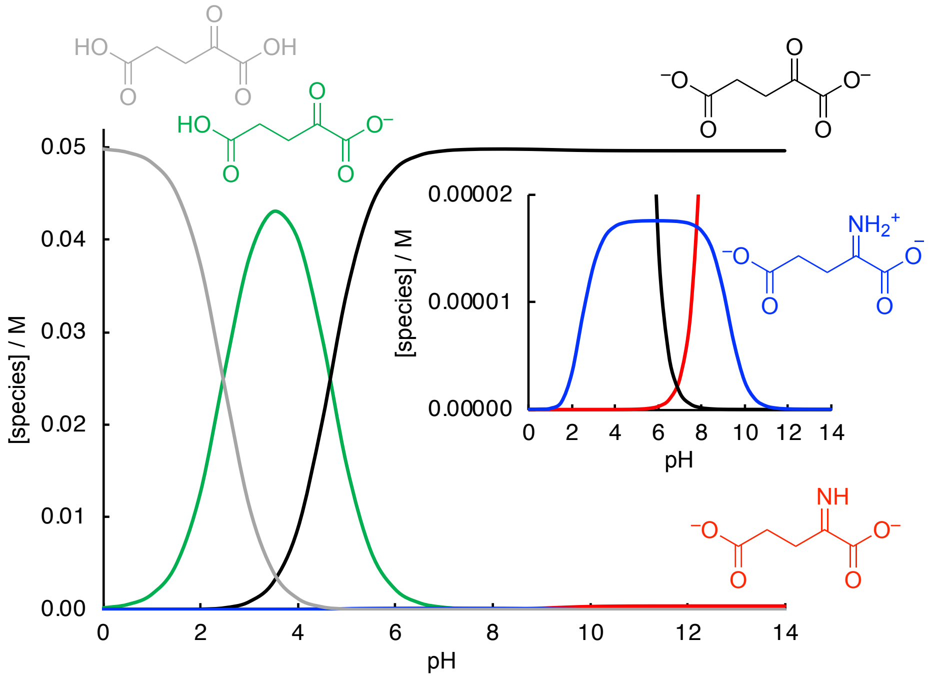

Figure S22. Species distribution for the system α-ketoglutarate (50 mM)/ NH_3 (100 mM) as a function of pH with K_I = 0.061, p$K_{a,NH4+}$ = 9.25, p$K_{a,\mathbf{I1}\text{-}H+}$ = 8.7, p$K_{a1,\mathbf{K4}}$ = 4.66, p$K_{a2,\mathbf{K4}}$ = 2.47. We did not consider the deprotonation of the terminal carboxylate of the iminium ion of **K4** due to the unknown acidity.

Kinetic Model

Note: The following analysis of the kinetics of reductive amination with $NaBH_3CN$ assumes conditions in which cyanohydrin formation can be neglected.

For the reductive amination of a keto acid **K** with BH_3CN^- the following three reactions are relevant:

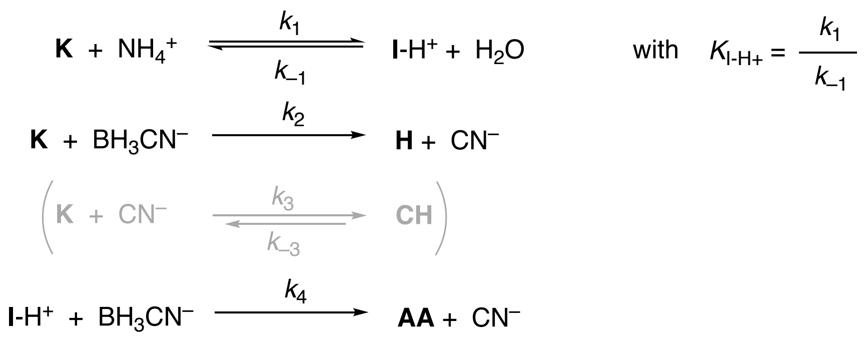

For the disappearance of **K**, the following rate law can be formulated when **CH** formation is neglected:

$$-\frac{d[\boldsymbol{K}]}{dt} = k_2[\boldsymbol{K}][BH_3CN^-] + k_4[\boldsymbol{I} - H^+][BH_3CN^-] \quad (17)$$

The equilibrium between **K** and **I**-H$^+$ is given as follows:

$$K_{I-H^+} = \frac{k_1}{k_{-1}} = \frac{[\boldsymbol{I} - H^+]}{[\boldsymbol{K}][NH_4^+]} \quad (18)$$

$$[\mathbf{I}\text{–H}^+] = K_{I-H^+}[\boldsymbol{K}][NH_4^+] \quad (19)$$

Eq (19) can be inserted into the rate law (17)

$$-\frac{d[\boldsymbol{K}]}{dt} = k_2[\boldsymbol{K}][BH_3CN^-] + k_4K_{I-H^+}[NH_4^+][\boldsymbol{K}][BH_3CN^-] \quad (20)$$

Under large excess of NH_4^+ and BH_3CN^-, the concentration of both species will remain approximately constant during the reaction. Accordingly, pseudo-first order rate constant can be introduced.

$$-\frac{d[\boldsymbol{K}]}{dt} = k_{2\psi}[\boldsymbol{K}] + k_{4\psi}[\boldsymbol{K}] \quad (21)$$

with:

$$k_{2\psi} = k_2[BH_3CN^-] \quad (22)$$
$$k_{4\psi} = k_4K_{I-H^+}[BH_3CN^-][NH_4^+] \quad (23)$$

According to (21), the observed rate k_{obs} will be the following:

$$k_{obs} = k_{2\psi} + k_{4\psi} \quad (24)$$

The rate law of type (22) can be solved analytically for both **K**, but also for **H** and **A**.[S12]

$$[\boldsymbol{K}]_t = [\boldsymbol{K}]_0 e^{-(k_{2\psi}+k_{4\psi})t} \quad (25)$$
$$[\boldsymbol{H}]_t = \frac{k_{2\psi}}{k_{2\psi} + k_{4\psi}} e^{-(k_{2\psi}+k_{4\psi})t} \quad (26)$$
$$[\boldsymbol{A}]_t = \frac{k_{4\psi}}{k_{2\psi} + k_{4\psi}} e^{-(k_{2\psi}+k_{4\psi})t} \quad (27)$$

To determine the underlying values behind $k_{2\psi}$ and $k_{4\psi}$, most conveniently correlations of k_{obs} vs. the concentration of one of the excess components can be performed.

a) For a series of reactions with different concentrations of BH_3CN^- (and constant concentration of NH_4^+), a linear correlation of k_{obs} vs. $[BH_3CN^-]$ should have a slope of $k_2 + k_4K_{I-H^+}[NH_4^+]$.

b) For a series of reactions with different concentrations of NH_4^+ (and constant concentration of BH_3CN^-), a linear correlation of k_{obs} vs. $[NH_4^+]$ should have an intercept corresponding to $k_2[BH_3CN^-]$ and a slope of $k_4K_{I-H^+}[BH_3CN^-]$.

We considered measurements using method a) more reliable as with method b), as b) requires very high concentrations of NH_4^+ to achieve a sufficient increase in the reaction rate relative to the direct reduction of the α-keto acid.

The slope in a correlation for type a) corresponds to $k_2 + k_4K_{I-H^+}[NH_4^+]$, which reflects formation of both hydroxy acid (via k_2) and amino acid (via $k_4K_{I-H^+}[NH_4^+]$). The product ratio of hydroxy and amino acid can accordingly be used to separate the two terms of the sum as discussed in the main text.

Separation of Reaction Rates Based on the Product Ratio

In the main text, we discuss that the product ratio of hydroxy/amino acid should stay constant under the assumption that our kinetic model is correct. However, we observe a small increase of the ratio toward the hydroxy acid with increasing concentration of BH_3CN^- which we attribute to our assumption of fast and reversible imine formation becoming problematic: With higher concentrations of BH_3CN^- the rates of the two reduction reactions (via k_2 and k_4) increase; the rate of the iminium formation k_1, however, is assumed to stay constant. Accordingly, at infinitely high concentration of BH_3CN^-, the reaction would exclusively yield hydroxy acid. At infinitely low concentration of BH_3CN^-, however, the reduction step would become infinitely slow relative to the iminium formation and clearly rate limiting.

We, therefore, proposed to use the linear correlation of the product ratio of hydroxy to amino acid [**H**]/[**A**] to extrapolate the product ratio at [BH_3CN^-] = 0. The ratio [**H**]/[**A**] at [BH_3CN^-] = 0 would correspond to reaction conditions where purely the reduction steps are rate limiting. Accordingly, this ratio should principally be applicable to separate the kinetics into the two reduction terms.

To verify this analysis, we performed our proposed analysis on a simulated dataset generated with COPASI. We simulated the product distribution after complete conversion for with the following three reactions (with the rates k_2 and k_4 taken from Table 2 in the main text; those of k_1 are arbitrarily set so that k_{-1} = 1 s^{-1} while the ratio k_1/k_{-1} equals the experimental value of $K_{I\text{-}H+}$).

$$\mathbf{K} + NH_4^+ \underset{k_{-1} = 1\ s^{-1}}{\overset{k_1 = 0.004\ L\ mol^{-1}\ s^{-1}}{\rightleftharpoons}} \mathbf{I}\text{-}H^+ \ (+ H_2O) \qquad \text{from: } K_{I\text{-}H+} = \frac{k_1}{k_{-1}} = 0.004\ M^{-1}$$

$$\mathbf{K} + BH_3CN^- \xrightarrow{k_2 = 0.005\ L\ mol^{-1}\ s^{-1}} \mathbf{H} + CN^-$$

$$\mathbf{I}\text{-}H^+ + BH_3CN^- \xrightarrow{k_4 = 2\ L\ mol^{-1}\ s^{-1}} \mathbf{A} + CN^-$$

Table S9. Simulations were performed with [**K**] = 0.01 M, [NH_4^+] = 0.01 M and different concentrations of BH_3CN^-.

[BH_3CN^-] / M	[**H**]$_{final}$ / M	[**A**]$_{final}$ / M	[**H**]/[**A**]
1.00 x 10^{-2}	5.58 x 10^{-3}	4.40 x 10^{-3}	1.27
3.00 x 10^{-2}	5.69 x 10^{-3}	4.31 x 10^{-3}	1.32
6.00 x 10^{-2}	5.82 x 10^{-3}	4.18 x 10^{-3}	1.39
1.00 x 10^{-1}	5.99 x 10^{-3}	4.01 x 10^{-3}	1.49
2.00 x 10^{-1}	6.36 x 10^{-3}	3.64 x 10^{-3}	1.74

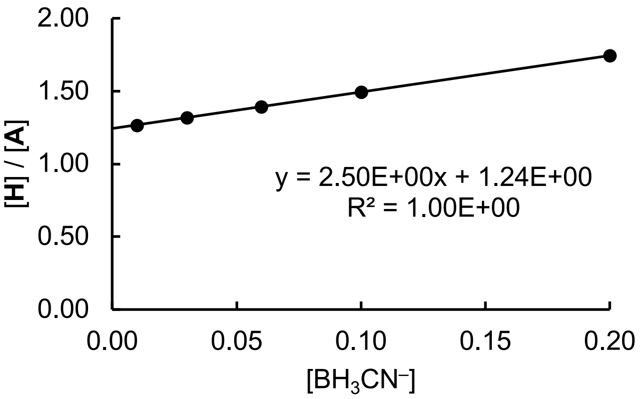

Correlation of [**H**]/[**A**] vs [BH_3CN^-] resulted in a linear correlation where the intercept with the ordinate had a value of [**H**]/[**A**] = 1.243.

According to the kinetic model, the formation of the hydroxy acid is given by k_2, that of the amino acid by $k_4K_{I\text{-}H+}[NH_4^+]$. When only the reduction steps are rate limiting, the ratio [**H**]/[**A**] should, therefore, correspond to the ratio $k_2/(k_4K_{I\text{-}H+}[NH_4^+])$.

$$\frac{[\boldsymbol{H}]}{[\boldsymbol{A}]} = \frac{k_2}{k_4 K_{I-H^+}[NH_4^+]} = \frac{0.005\, L\, mol^{-1}s^{-1}}{2\, L\, mol^{-1}s^{-1}\; 0.004\, L\, mol^{-1}s^{-1}\; 0.5\, mol\, L^{-1}} = 1.25$$

With the numbers used to construct the simulation, [**H**]/[**A**] is expected to have a value of 1.25. This value is in almost perfect agreement with the ratio [**H**]/[**A**] extrapolated from the product ratios at [BH_3CN^-] = 0 obtained as 1.243.

Kinetics with Variation of [BH_3CN^-]

NMR tubes were charged with a solution of dimethyl sulfone in D_2O (50 µL) and a stock solution of the keto acid, ammonium chloride, phosphate buffer and water. Lastly, the corresponding amount of a stock solution of $NaBH_3CN$ was added, the NMR tube sealed, mixed and a timer started. The NMR tube was inserted into the spectrometer which shortly before was locked and shimmed on an NMR sample of similar composition, and the measurement started. The time difference from mixing the sample and acquisition of the first spectrum was noted and used to correct the kinetics. For the kinetics, ^1H NMR spectra were continuously acquired and the time-dependent concentration for the disappearance of the keto acid was determined from the integral ratio relative to the internal standard. Pseudo-first order rate constants were obtained by least-squares fitting as outlined in the general section and correlated against [$NaBH_3CN$] to afford linear correlations.
After every kinetic run, the product ratio of hydroxy and amino acid was determined by quantitative ^1H NMR spectroscopy (d_1 = 30 s, 500 MHz). The product ratio was found to be linearly correlated with [$NaBH_3CN$] and was next used to calculate the product ratio at [$NaBH_3CN$] = 0.

Reaction of pyruvate (K1) with varying amount of $NaBH_3CN$ in the presence of NH_4Cl

The kinetics of pyruvate disappearance in the reaction of pyruvate with $NaBH_3CN$ were followed by ^1H NMR spectroscopy at 20 °C in pH 4 phosphate solution (0.5 M) in H_2O containing 8.3% D_2O (RM02-341).

[**K1**]/ M	[NH_4Cl]/ M	[$NaBH_3CN$]/ M	k_{obs}/ s^{-1}	[Alanine] : [Lactate]
3.30×10^{-3}	5.00×10^{-1}	3.33×10^{-2}	3.42×10^{-4}	1 : 1.83
3.30×10^{-3}	5.00×10^{-1}	5.55×10^{-2}	5.31×10^{-4}	1 : 1.98
3.30×10^{-3}	5.00×10^{-1}	7.77×10^{-2}	7.19×10^{-4}	1 : 2.16
3.30×10^{-3}	5.00×10^{-1}	1.00×10^{-1}	8.81×10^{-4}	1 : 2.33

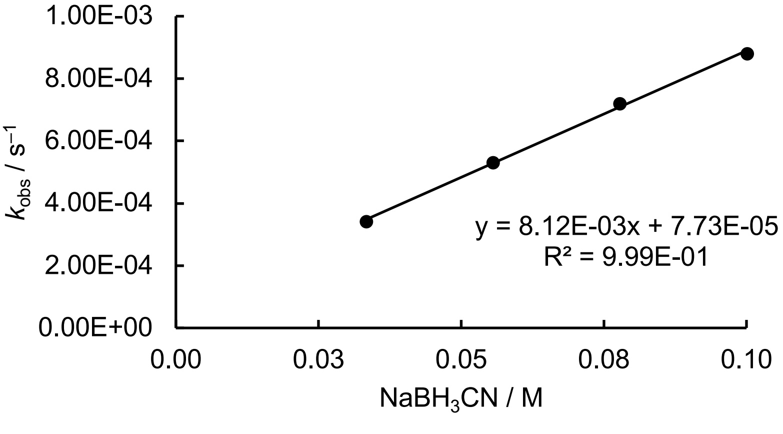

$k_2 + k_4 K_{I-H^+}[NH_4^+]$ = (8.12 ± 0.21) x 10^{-3} L mol^{-1} s^{-1}

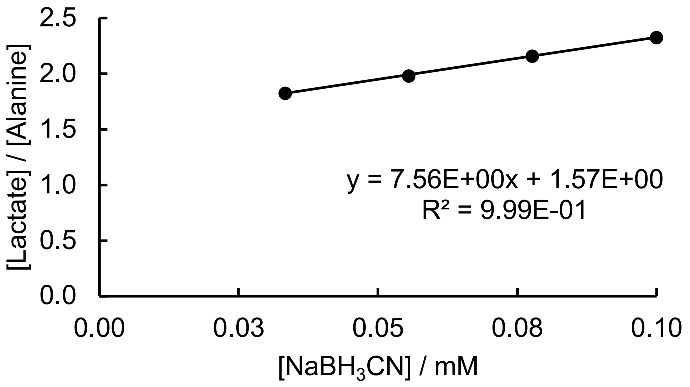

[Lactate] : [Alanine] at $[BH_3CN^-]$ = 0: 1.57 ± 0.01

Assuming an infinitely slow reaction (at $[BH_3CN^-]$ = 0), the individual rates can be calculated from the product ratio:

Rate due to alanine: $k_2 = \frac{1}{1+1.57} * 8.12\ x\ 10^{-3} = 3.16\ x\ 10^{-3}\ L\ mol^{-1}\ s^{-1}$

Rate due to lactate: $k_4 K_{I-H^+}[NH_4^+] = \frac{1.57}{1+1.57} * 8.12\ x\ 10^{-3} = 4.96\ x\ 10^{-3} L\ mol^{-1}\ s^{-1}$

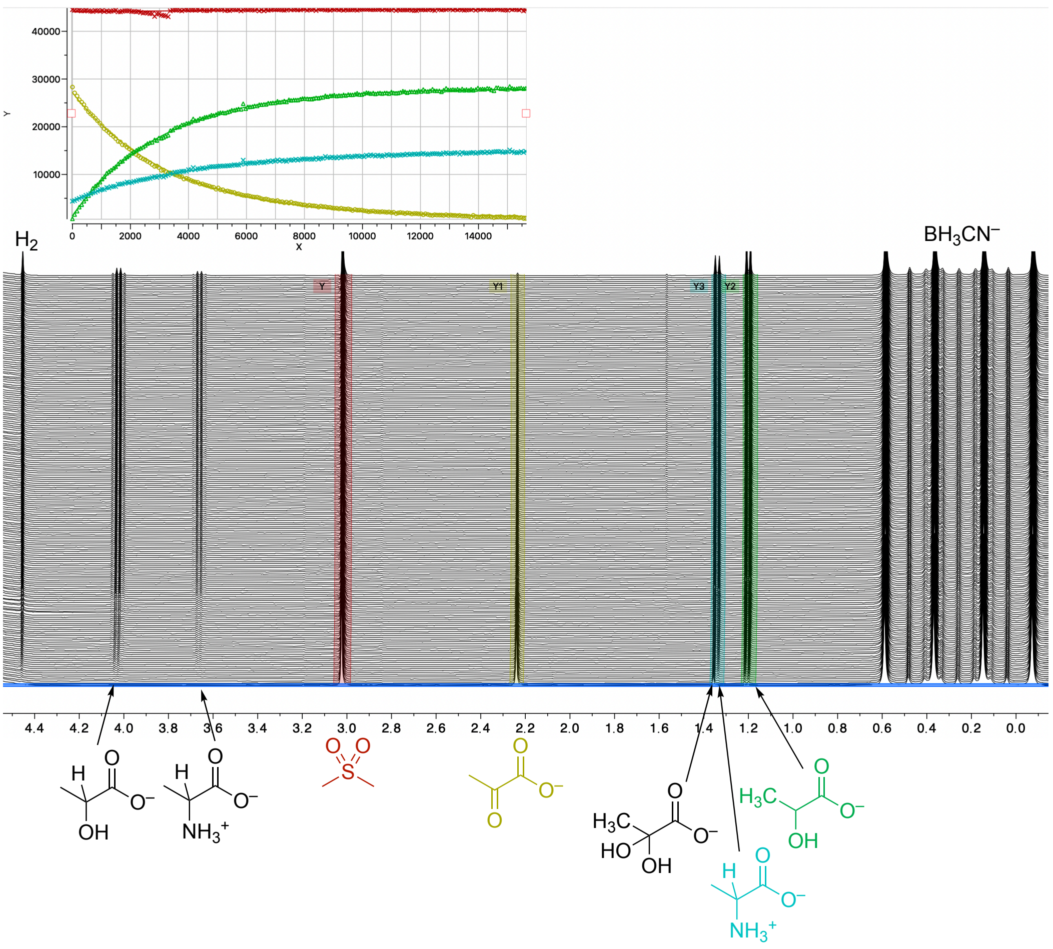

Figure S23. ^{1}H NMR kinetics (noesygppr1d, 400 MHz, ns = 16, 20 °C) of the reaction of **K1** (3.3 mM) with $NaBH_3CN$ (33.3 mM) and NH_4Cl (500 mM) in pH 4 phosphate solution (0.5 M) in H_2O containing 8.3% D_2O (RM02-341-1). *Insert on top: Unprocessed time-dependent peak areas from the data analysis module in MNOVA (correlation lines correspond to a tentative exponential fit).*

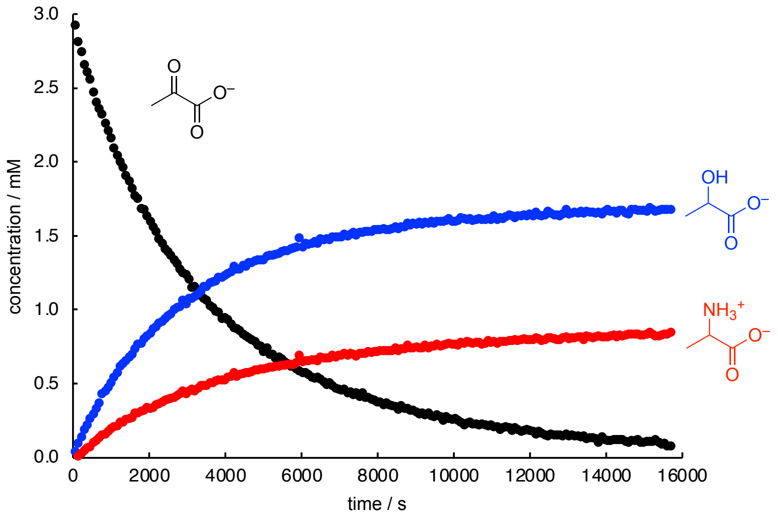

Figure S24. Time-dependent concentrations determined relative to dimethyl sulfone from the ^{1}H NMR kinetics in Figure S23 used for the determination of the rate k_{obs}.

Reaction of glyoxylate (K2) with varying amount of $NaBH_3CN$ in the presence of NH_4Cl

The kinetics of glyoxylate disappearance in the reaction of glyoxylate with $NaBH_3CN$ were followed by ^{1}H NMR spectroscopy at 20 °C in pH 4 phosphate solution (0.5 M) in H_2O containing 8.3% D_2O (RM02-340/346).

[**K2**]/ M	[NH_4Cl]/ M	[$NaBH_3CN$]/ M	k_{obs}/ s^{-1}	[Glycine] : [Glycolate]
3.30×10^{-3}	5.00×10^{-1}	3.33×10^{-2}	3.32×10^{-3}	1 : 1.28
3.30×10^{-3}	5.00×10^{-1}	4.44×10^{-2}	4.09×10^{-3}	1 : 1.33
3.30×10^{-3}	5.00×10^{-1}	5.55×10^{-2}	4.40×10^{-3}	1 : 1.40
3.30×10^{-3}	5.00×10^{-1}	6.66×10^{-2}	4.83×10^{-3}	1 : 1.45
3.30×10^{-3}	5.00×10^{-1}	7.77×10^{-2}	5.22×10^{-3}	1 : 1.48
3.30×10^{-3}	5.00×10^{-1}	8.88×10^{-2}	5.63×10^{-3}	1 : 1.56

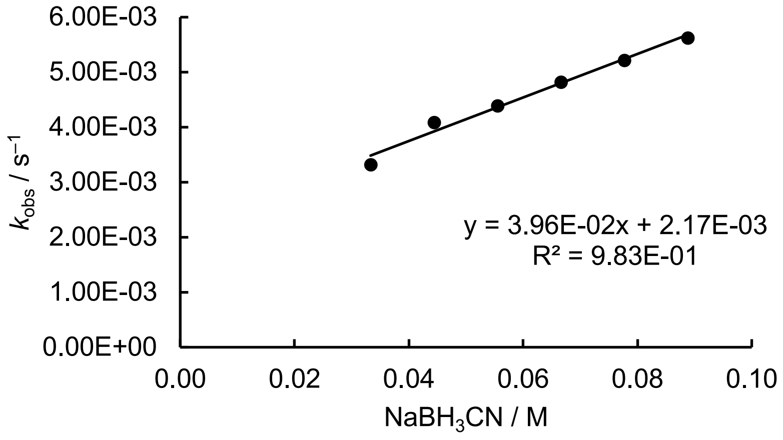

$k_2 + k_4 K_{I-H^+}[NH_4^+]$ = (3.96 ± 0.26) x 10^{-2} L mol^{-1} s^{-1}

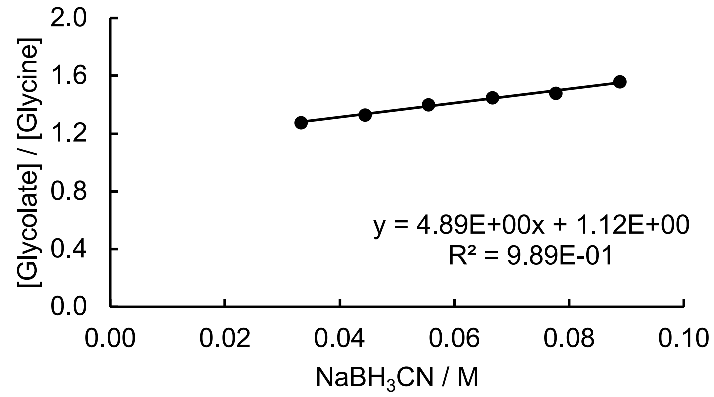

[glycolate] : [glycine] at [BH_3CN^-] = 0: 1.12 ± 0.02

Assuming an infinitely slow reaction (at [BH_3CN^-] = 0), the individual rates can be calculated from the product ratio:

Rate due to glycolate: $k_2 = \frac{1.12}{1+1.12} * 3.96\ x\ 10^{-2} = 2.09\ x\ 10^{-2}\ L\ mol^{-1}\ s^{-1}$

Rate due to glycine: $k_4 K_{I-H^+}[NH_4^+] = \frac{1}{1+1.12} * 8.45\ x\ 10^{-2} = 1.87\ x\ 10^{-2}\ L\ mol^{-1}\ s^{-1}$

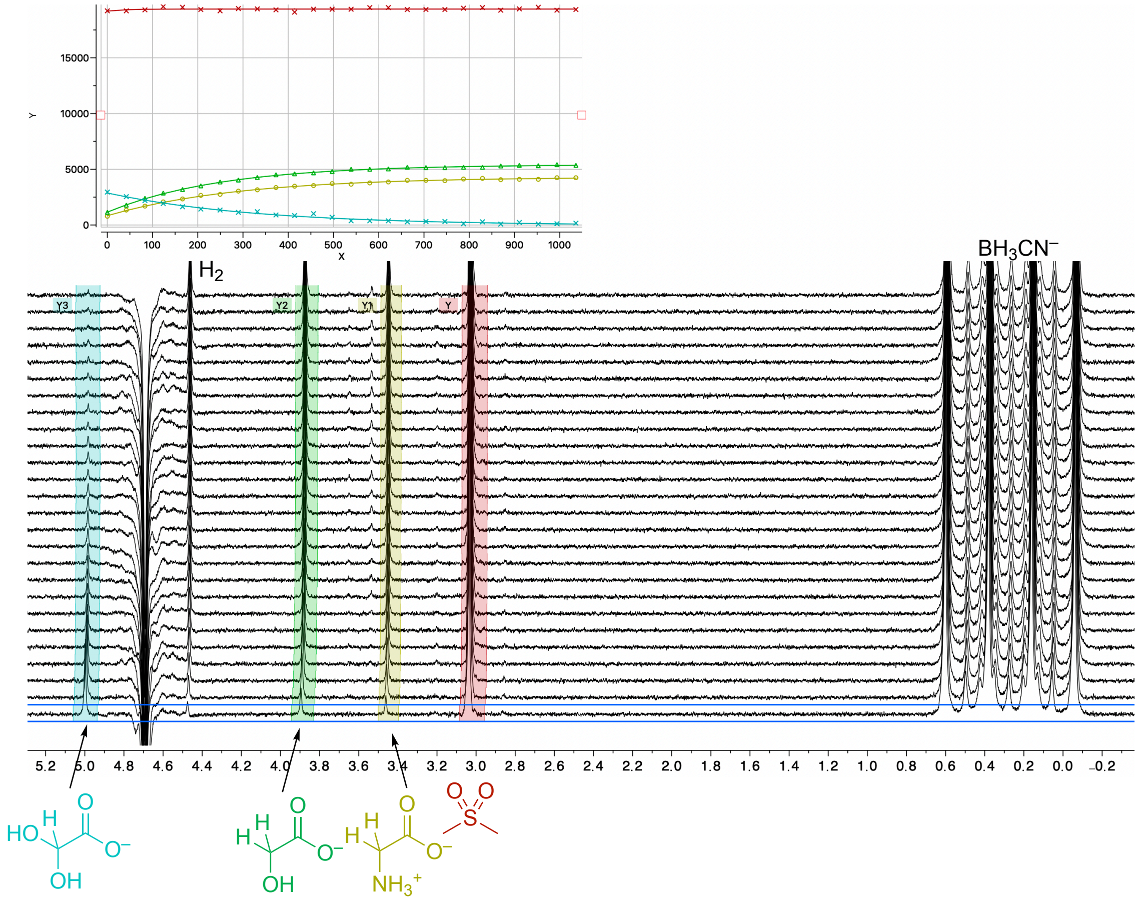

Figure S25. ^{1}H NMR kinetics (noesygppr1d, 400 MHz, ns = 8, 20 °C) of the reaction of **K2** (3.3 mM) with $NaBH_3CN$ (33.3 mM) and NH_4Cl (500 mM) in pH 4 phosphate solution (0.5 M) in H_2O containing 8.3% D_2O (RM02-340-1). *Insert on top: Unprocessed time-dependent peak areas from the data analysis module in MNOVA (correlation lines correspond to a tentative exponential fit).*

Reaction of oxaloacetate (K3) with varying amount of $NaBH_3CN$ in the presence of NH_4Cl

Kinetics of oxaloacetate disappearance in the reaction of oxaloacetate with $NaBH_3CN$ followed by ^1H NMR spectroscopy at 20 °C in pH 5 phosphate solution (0.5 M) in H_2O containing 8.3% D_2O (RM02-345).

Note: For every single NMR measurement, a fresh stock solution of oxaloacetate was prepared as it decomposes rather rapidly in aqueous solution. Due to the rapid decomposition of oxaloacetate, the kinetics for the decomposition in the absence of hydride were included in the linear correlation to determine the reaction rate.

[**K3**]/ M	[$NaBH_3CN$]/ M	[NH_4Cl]/ M	k_{obs}/ s^{-1}	[Aspartate] : [Malate]
3.30×10^{-3}	0.00	5.00×10^{-1}	7.91×10^{-5}	--
3.30×10^{-3}	3.33×10^{-2}	5.00×10^{-1}	6.75×10^{-4}	1 : 6.56
3.30×10^{-3}	5.55×10^{-2}	5.00×10^{-1}	1.15×10^{-3}	1 : 7.14
3.30×10^{-3}	7.77×10^{-2}	5.00×10^{-1}	1.61×10^{-3}	1 : 7.57
3.30×10^{-3}	1.00×10^{-1}	5.00×10^{-1}	1.90×10^{-3}	1 : 8.55
3.30×10^{-3}	1.22×10^{-1}	5.00×10^{-1}	2.49×10^{-3}	1 : 8.58

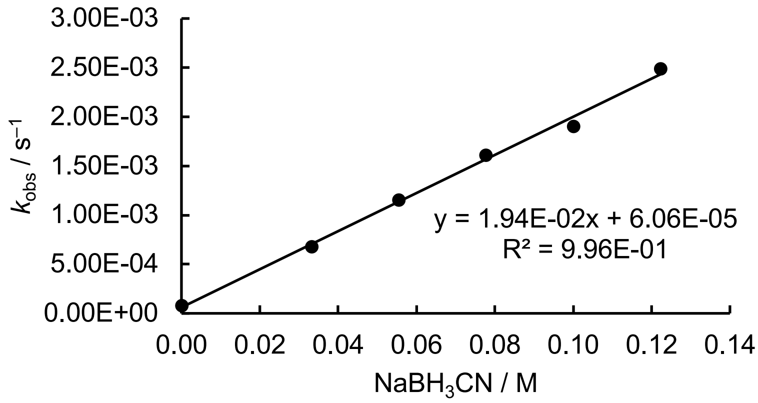

$k_2 + k_4K_{I-H^+}[NH_4^+]$ = (1.94 ± 0.06) x 10^{-2} L mol^{-1} s^{-1}

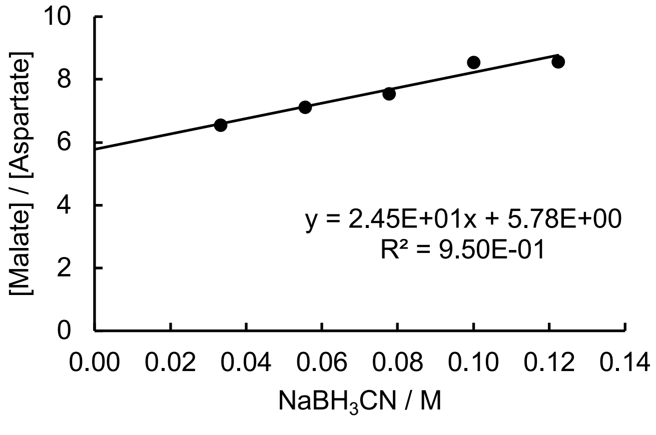

[malate] : [aspartate] at [BH_3CN^-] = 0: 5.78 ± 0.27

Assuming an infinitely slow reaction (at [BH_3CN^-] = 0), the individual rates can be calculated from the product ratio:

Rate due to malate: $k_2 = \frac{5.78}{1+5.78} * 1.94\ x\ 10^{-2} = 1.65\ x\ 10^{-2}\ L\ mol^{-1}\ s^{-1}$

Rate due to aspartate: $k_4 K_{I-H^+}[NH_4^+] = \frac{1}{1+5.78} * 1.94\ x\ 10^{-2} = 2.86\ x\ 10^{-3} L\ mol^{-1}\ s^{-1}$

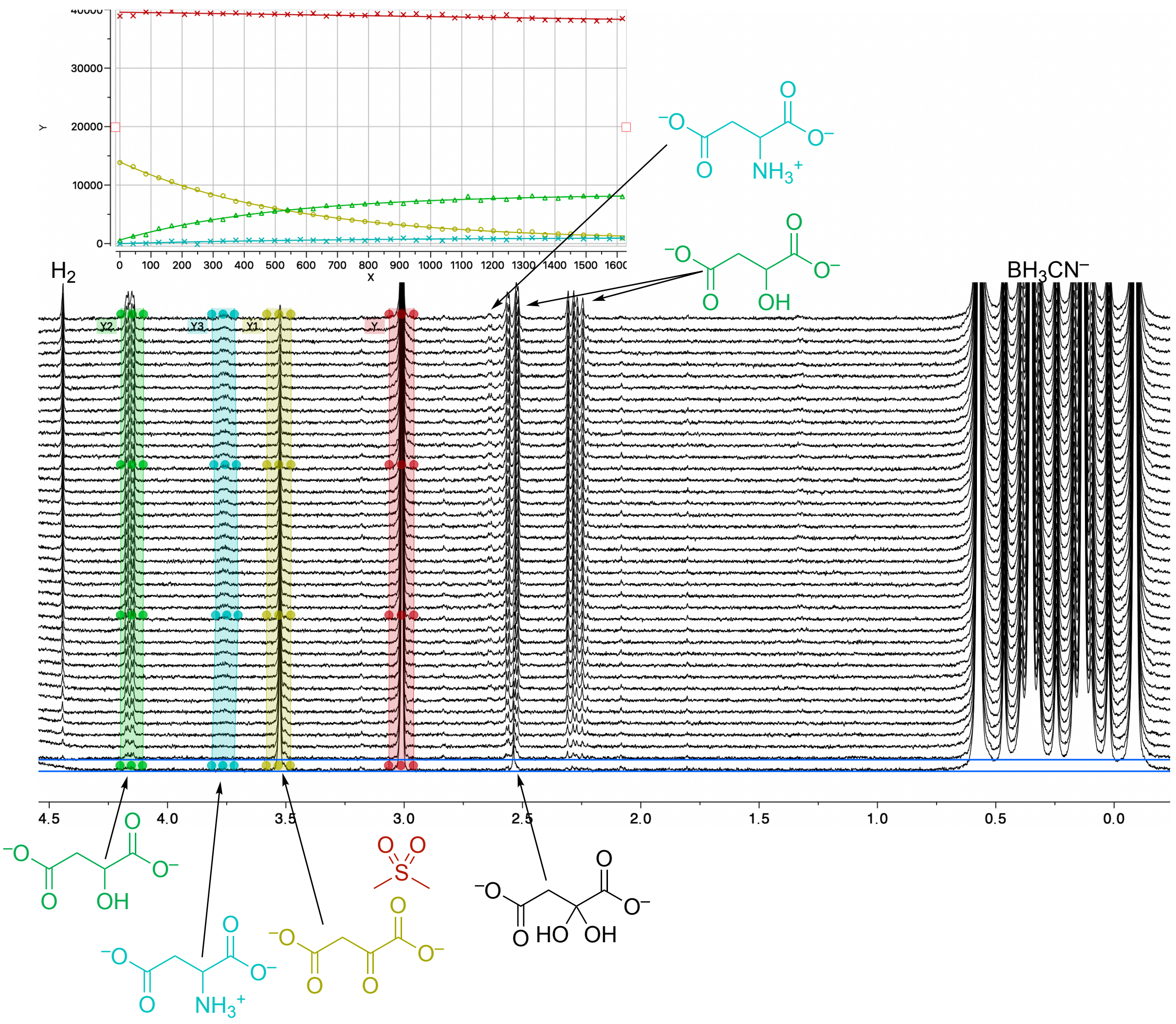

Figure S26. ^{1}H NMR kinetics (noesygppr1d, 400 MHz, ns = 8, 20 °C) of the reaction of **K3** (3.3 mM) with $NaBH_3CN$ (100 mM) and NH_4Cl (500 mM) in pH 5 phosphate solution (0.5 M) in H_2O containing 8.3% D_2O (RM02-345-4). *Insert on top: Unprocessed time-dependent peak areas from the data analysis module in MNOVA (correlation lines correspond to a tentative exponential fit).*

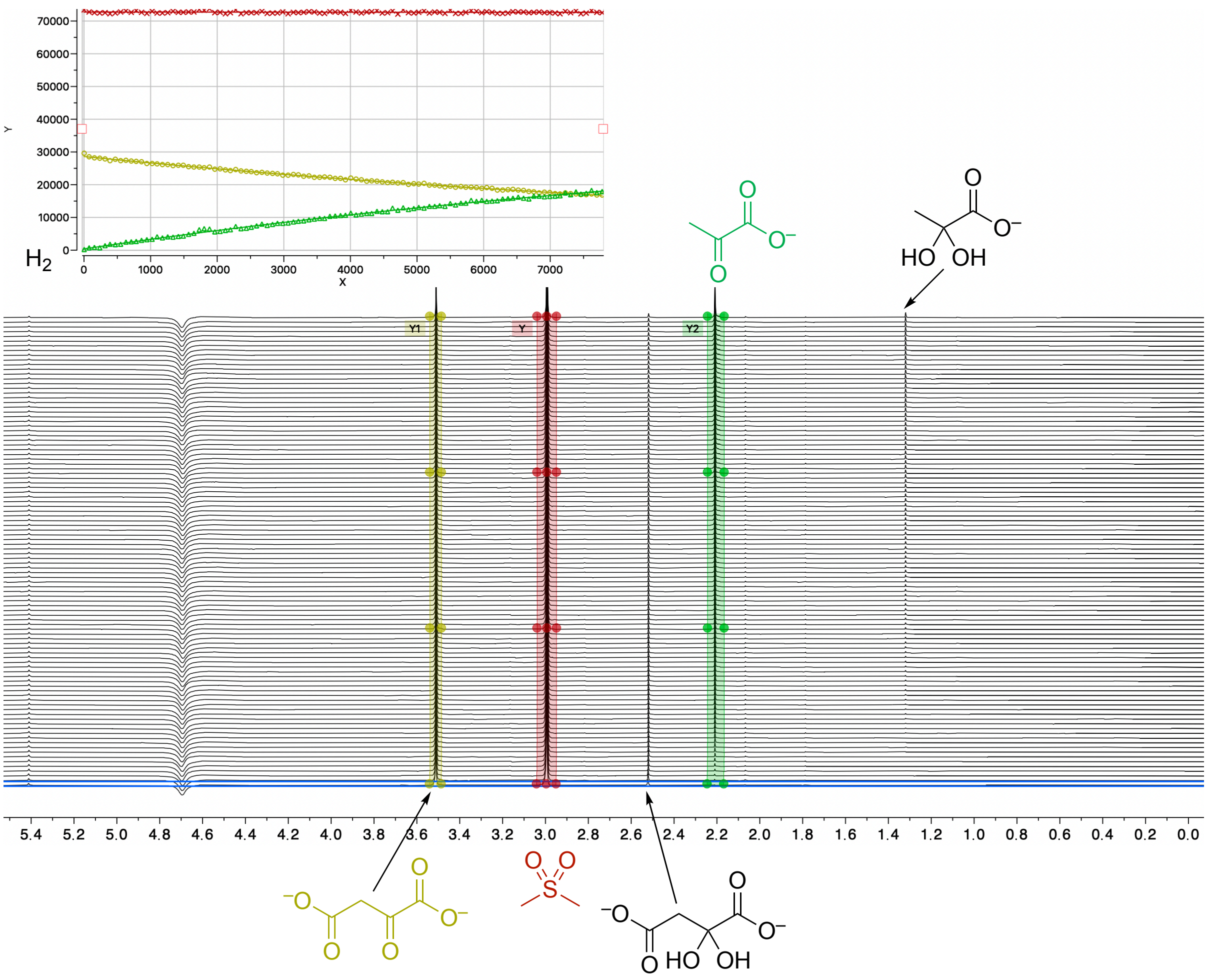

Figure S27. ^{1}H NMR kinetics (noesygppr1d, 400 MHz, ns = 16, 20 °C) of the decomposition reaction of **K3** (3.3 mM) in the presence of NH_4Cl (500 mM) in pH 5 phosphate solution (0.5 M) in H_2O containing 8.3% D_2O (RM02-345-4). *Insert on top: Unprocessed time-dependent peak areas from the data analysis module in MNOVA (correlation lines correspond to a tentative exponential fit).*

Reaction of α-ketoglutarate (K4) with varying amount of $NaBH_3CN$ in the presence of NH_4Cl

Kinetics of α-ketoglutarate disappearance in the reaction of α-ketoglutarate with $NaBH_3CN$ followed by ^1H NMR spectroscopy at 20 °C in pH 5 phosphate solution (0.5 M) in H_2O containing 8.3% D_2O (RM02-339).

[**K4**]/ M	[NH_4Cl]/ M	[$NaBH_3CN$]/ M	k_{obs}/ s^{-1}	[glutamate] : [2-hydroxyglutarate]
3.30×10^{-3}	3.33×10^{-2}	5.00×10^{-1}	2.64×10^{-4}	1 : 5.28
3.30×10^{-3}	5.55×10^{-2}	5.00×10^{-1}	4.83×10^{-4}	1 : 6.25
3.30×10^{-3}	7.77×10^{-2}	5.00×10^{-1}	6.50×10^{-4}	1 : 6.58
3.30×10^{-3}	1.00×10^{-1}	5.00×10^{-1}	8.50×10^{-4}	1 : 7.60

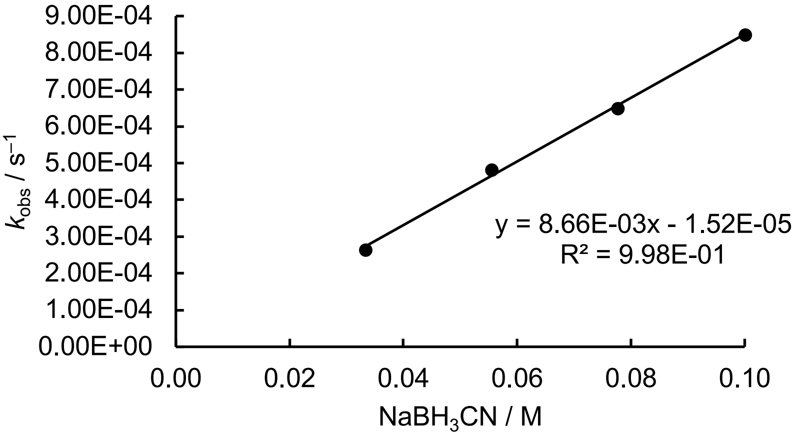

$k_2 + k_4 K_{I-H^+}[NH_4^+]$ = (8.66 ± 0.30) x 10^{-3} L mol^{-1} s^{-1}

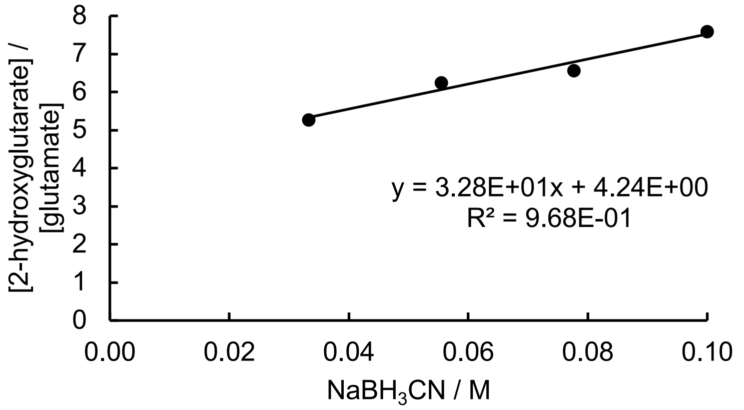

Assuming an infinitely slow reaction (at [BH_3CN^-] = 0), the product ratio of [2-hydroxyglutarate] : [glutamate] is 4.24 : 1; accordingly, the individual rates can be calculated:

[2-hydroxyglutarate] : [glutamate] at [BH_3CN^-] = 0: 4.24 ± 0.30

Assuming an infinitely slow reaction (at [BH₃CN⁻] = 0), the individual rates can be calculated from the product ratio:

Rate due to 2-hydroxyglutarate: $k_2 = \frac{4.24}{1+4.24} * 8.66\ x\ 10^{-3} = 7.01\ x\ 10^{-3}\ L\ mol^{-1}\ s^{-1}$

Rate due to glutamate: $k_4 K_{I-H^+}[NH_4^+] = \frac{1}{1+4.24} * 8.66\ x\ 10^{-3} = 1.65\ x\ 10^{-3} L\ mol^{-1}\ s^{-1}$

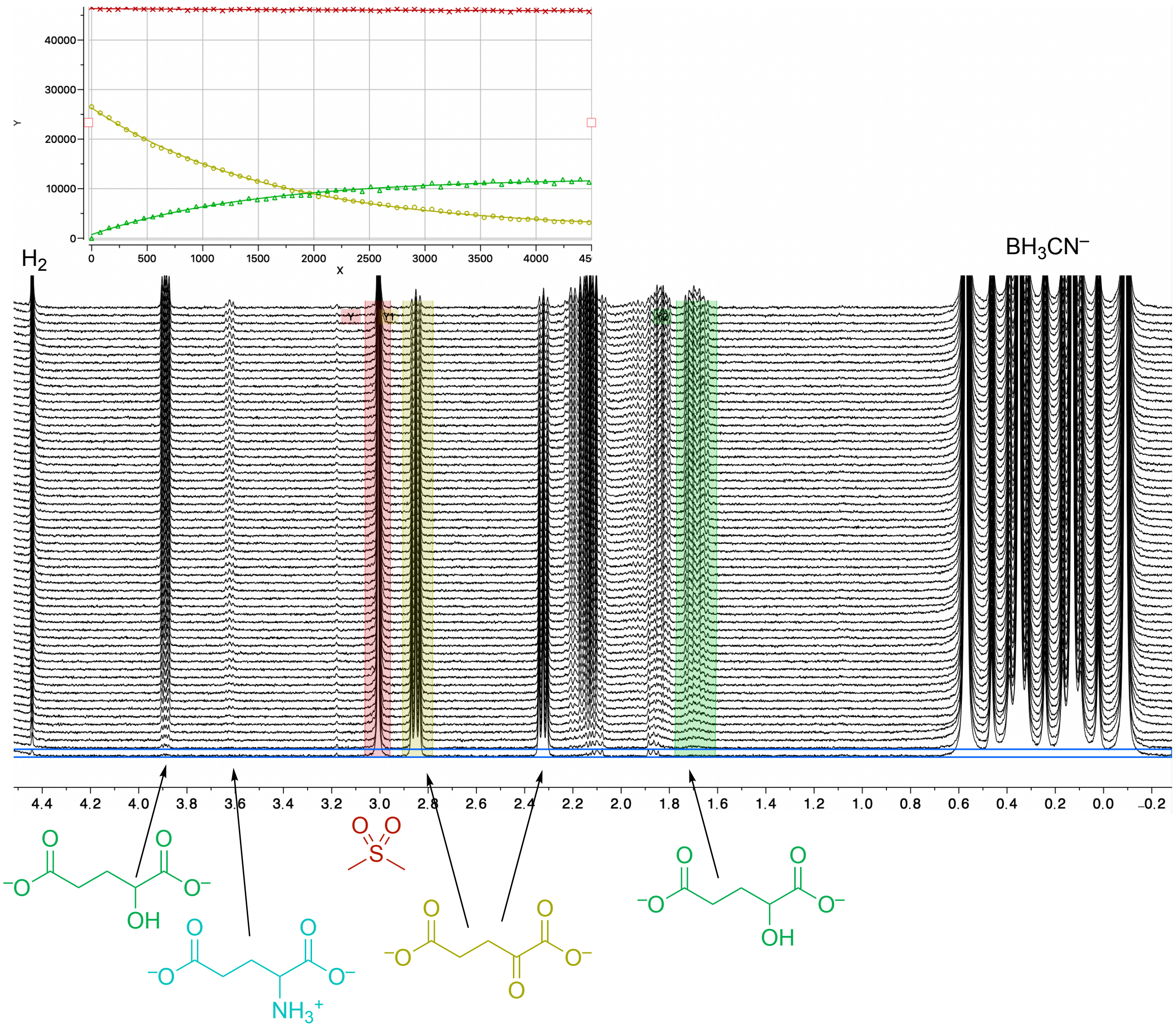

Figure S28. ^{1}H NMR kinetics (noesygppr1d, 400 MHz, ns = 8, 20 °C) of the reaction of **K4** (3.3 mM) with NaBH$_3$CN (77.7 mM) and NH$_4$Cl (500 mM) in pH 5 phosphate solution (0.5 M) in H$_2$O containing 8.3% D$_2$O (RM02-339-3). *Insert on top: Unprocessed time-dependent peak areas from the data analysis module in MNOVA (correlation lines correspond to a tentative exponential fit).*

Competition Experiments for Reductive Amination

Competition experiments were performed for the reaction of **K4**/**K1** and **K4**/**K2** with $NaBH_3CN$ in the presence of NH_4Cl. The competition experiment between **K4**/**K3** was attempted but the analysis showed to be complicated due to pyruvate formed from the decarboxylation of **K3** also reacting to both lactate and alanine.

The full procedure is exemplarily shown for the competition experiment between **K1**/**K4** with $NaBH_3CN$ and was analogously performed for the competition between **K4**/**K2**.

Reaction mixture: A 5 mL glass vial was charged with α-ketoglutarate (disodium salt, 22.70 mg, 0.100 mmol) and sodium pyruvate (11.06 mg, 0.100 mmol). Water (1 mL) was added, and the contents of the vial mixed vigorously.

Reaction samples: NMR tubes were charged with the stock solution of keto acids (96 µL, resulting in ≈ 8 mM final concentration of each keto acid), buffer (30 µL from a 1 M stock solution; resulting in a final concentration of 50 mM), water (311 µL), a solution of NH_4Cl (100µL, 3.0 M in H_2O, pH adjusted to pH 4-7 by the addition of NaCl or NaOH; resulting in a final concentration of 500 mM), a solution of DSS in D_2O of known concentration as integration standard (50 µL) and finally a stock solution of $NaBH_3CN$ (0.138 M, 13 µL) to give a total volume of 600 µL. The NMR tubes were sealed, mixed and kept in an air-conditioned laboratory (20-21 °C). ^1H NMR spectra were recorded after 30 min – 4 h (the reaction time is dependent on the pH). As buffers, 1 M stock solutions of acetate buffer (pH 4 and 5) as well as phosphate buffer (pH 6 and 7) were used.

Control sample: A separate NMR tube was charged with the stock solution of keto acids (96 µL, resulting in 8 mM final concentration of each keto acid), pH 7 phosphate buffer (from a 1 M stock solution, 30 µL), water (424 µL) and a solution of DSS in D_2O of known concentration as integration standard (50 µL).

NMR Analysis was performed analogous as described for the competition experiments of keto acids. However, in the competition reaction of **K1** and **K4** the resonances of alanine (**A1**) and glutamate (**A4**) are overlapping. To deconvolute the signals, the line-fitting function of Mnova was employed and the integral ratio relative to the peak of the standard determined from the individually fitted resonances. Again, integrals were corrected using response factors from quantitative NMR spectroscopy analogously as discussed for the competition experiments for keto acid reduction.

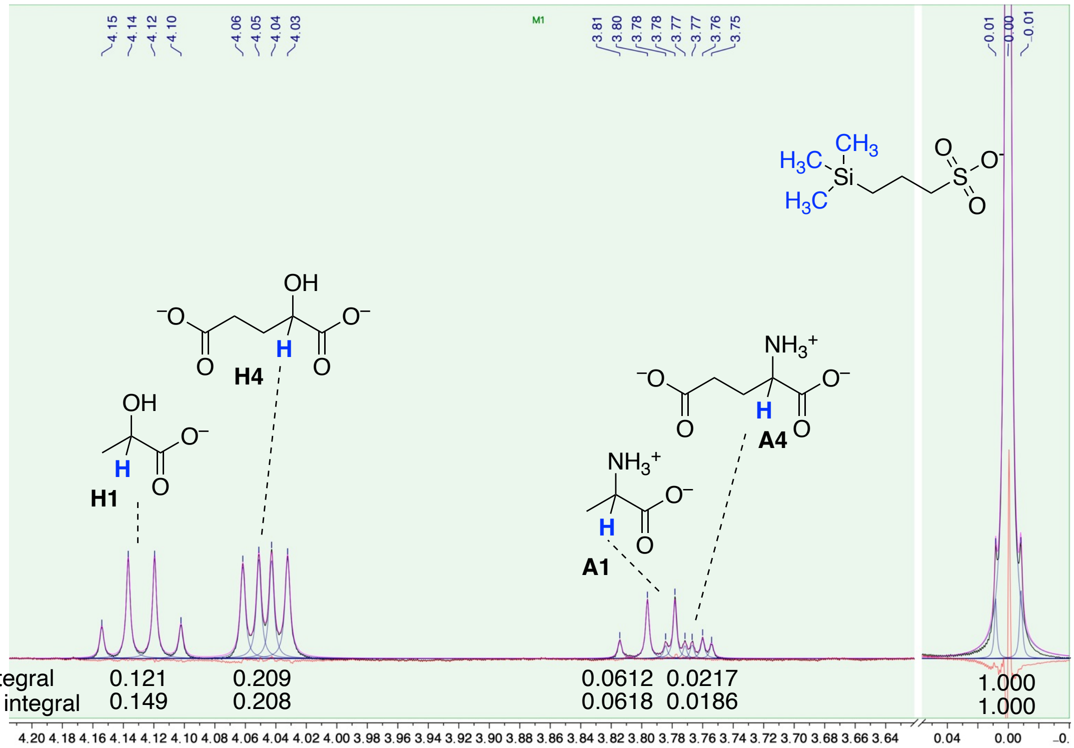

Figure S29. Line-shape analysis in the ^{1}H NMR spectra (400 MHz, 8% D_2O in H_2O) of the competition experiment of the reductive amination of **K4**/**K1** with $NaBH_3CN$ at pH 5 (0.500 M acetate buffer). Based on the areas of the fitted curves, the integral ratio was calculated. The integrals were corrected ("corr. integral") with response factors for all analytes to ensure correct quantification.

Table S10. Normalized and concentration-corrected integrals values before and after reaction for keto and hydroxy acids in the competition experiment of **K1**/**K4** with $NaBH_3CN$ used to calculate the pH-dependent competition constants. For pH 4 and 5 acetate buffer was used; for pH 6 and 7 phosphate buffer. Experiment: RM03-452.

	pH	[buffer] / M	[**K4**]$_0$	[**K1**]$_0$	[**H4**]$_t$	[**A4**]$_t$	[**H1**]$_t$	[**A1**]$_t$
before reaction			0.938	1.147	--	--	--	--
after reaction	4	0.5	--		0.291	0.000	0.229	0.076
	5	0.05			0.177	0.023	0.118	0.066
	5	0.5			0.208	0.019	0.149	0.062
	6	0.5			0.101	0.024	0.089	0.058
	7	0.05			0.097	0.047	0.119	0.103
	7	0.5			0.076	0.041	0.094	0.085

The amount of keto acid after reaction, [**K**]$_t$, was determined for the contributions due to keto acid reduction (to give [**K**]$_{t,HO}$) and reductive amination (to give [**K**]$_{t,AA}$) from the amounts of hydroxy acid ([**H**]$_t$) and amino acid ([**A**]$_t$) after reaction.

$$[\mathbf{K}]_{t,HO} = [\mathbf{K}]_0 - [\mathbf{H}]_t$$
$$[\mathbf{K}]_{t,AA} = [\mathbf{K}]_0 - [\mathbf{A}]_t$$

Table S11. Amounts of keto acids after reaction due to direct reduction ($[\mathbf{K}]_{t,HO}$) or reductive amination ($[\mathbf{K}]_{t,AA}$).

pH	[buffer] / M	$[\mathbf{K4}]_{t,HO}$ = $[\mathbf{K4}]_0 - [\mathbf{H4}]_t$	$[\mathbf{K4}]_{t,AA}$ = $[\mathbf{K4}]_0 - [\mathbf{A4}]_t$	$[\mathbf{K1}]_{t,HO}$ = $[\mathbf{K1}]_0 - [\mathbf{H1}]_t$	$[\mathbf{K1}]_{t,AA}$ = $[\mathbf{K1}]_0 - [\mathbf{A1}]_t$
4	0.5	0.648	0.938	0.918	1.071
5	0.05	0.761	0.915	1.029	1.081
5	0.5	0.730	0.920	0.998	1.085
6	0.5	0.837	0.915	1.058	1.089
7	0.05	0.841	0.891	1.028	1.044
7	0.5	0.862	0.897	1.053	1.062

Next, the logarithm of the ratio of keto acid before and after reaction was determined for all individual contributions.

Table S12. Logarithm of the ratio of keto acid before and after reaction.

pH	[buffer] / M	$\lg([\mathbf{K4}]_0/[\mathbf{K4}]_{t,HO})$	$\lg([\mathbf{K4}]_0/[\mathbf{K4}]_{t,AA})$	$\lg([\mathbf{K1}]_0/[\mathbf{K1}]_{t,HO})$	$\lg([\mathbf{K1}]_0/[\mathbf{K1}]_{t,AA})$
4	0.5	0.1611	0.0000	0.0966	0.0298
5	0.05	0.0909	0.0110	0.0474	0.0256
5	0.5	0.1089	0.0087	0.0606	0.0240
6	0.5	0.0494	0.0111	0.0352	0.0227
7	0.05	0.0476	0.0224	0.0475	0.0408
7	0.5	0.0366	0.0193	0.0373	0.0336

Finally, the competition constants were determined relative to the keto acid reduction of **K4** to give the hydroxy acid **H4** by taking the ratio of the logarithms.

$$\kappa = \frac{k_2\,(\mathbf{K})_x}{k_2\,(\mathbf{K4})_{HO}} = \frac{\lg([\mathbf{K}]_0/[\mathbf{K}]_{t,X})}{\lg([\mathbf{K4}]_0/[\mathbf{K4}]_{t,HO})}$$

Table S13. Competition constants for reduction (to yield hydroxy acids **H**) or reductive amination (to yield amino acids **A**) relative to the keto acid reduction of **K4**.

	$\kappa = \frac{k_2\,(\mathbf{K4})_{reduction}}{k_2\,(\mathbf{K4})_{reduction}}$	$\kappa = \frac{k_2\,(\mathbf{K4})_{red.\ amin.}}{k_2\,(\mathbf{K4})_{reduction}}$	$\kappa = \frac{k_2\,(\mathbf{K1})_{reduction}}{k_2\,(\mathbf{K4})_{reduction}}$	$\kappa = \frac{k_2\,(\mathbf{K1})_{red.\ amin.}}{k_2\,(\mathbf{K4})_{reduction}}$
pH	$\lg([\mathbf{K4}]_0/[\mathbf{K4}]_{t,HO})/\lg([\mathbf{K4}]_0/[\mathbf{K4}]_{t,HO})$	$\lg([\mathbf{K4}]_0/[\mathbf{K4}]_{t,AA})/\lg([\mathbf{K4}]_0/[\mathbf{K4}]_{t,HO})$	$\lg([\mathbf{K1}]_0/[\mathbf{K1}]_{t,HO})/\lg([\mathbf{K4}]_0/[\mathbf{K4}]_{t,HO})$	$\lg([\mathbf{K1}]_0/[\mathbf{K1}]_{t,AA})/\lg([\mathbf{K4}]_0/[\mathbf{K4}]_{t,HO})$
4[a]	1.000	0.000	0.600	0.185
5[b]	1.000	0.121	0.521	0.282
5[a]	1.000	0.080	0.556	0.221
6[a]	1.000	0.224	0.713	0.460
7[b]	1.000	0.471	1.000	0.859
7[a]	1.000	0.527	1.019	0.917

[a] at 0.5 M buffer concentration. [b] at 0.05 M buffer concentration.

Competition experiments of the reductive amination of K4/K2 (RM03-453)

The analysis was done in an analogous way as for the competition experiments of the reductive amination of **K4**/**K1**. However, the amount of glutamate formed was so low, that its concentration could not be accurately determined by NMR. Accordingly, we did not analyze the glutamate formation in these reactions.

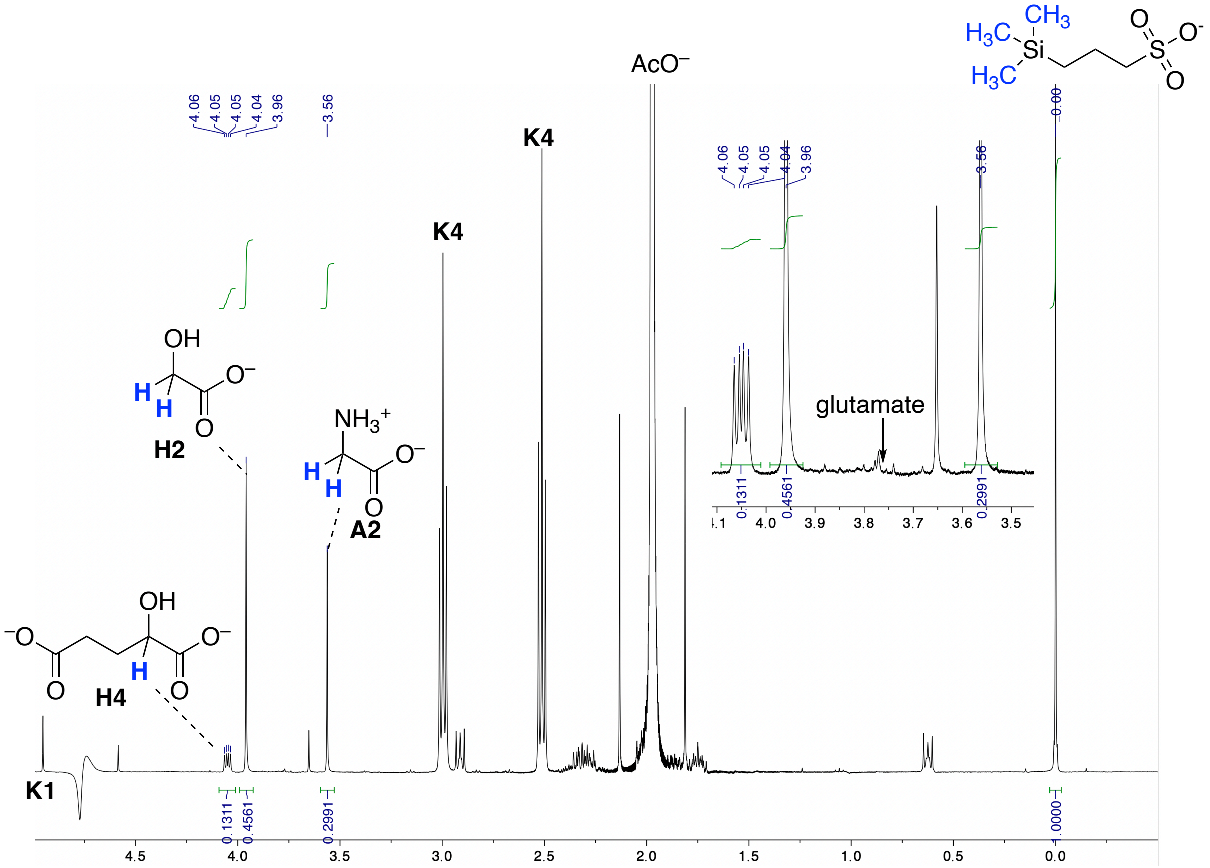

Figure S30. ^{1}H NMR spectra (400 MHz, 8% D_2O in H_2O) of the competition experiment of the reductive amination of **K4**/**K2** with $NaBH_3CN$ at pH 5 (0.50 M acetate buffer). The insert shows a magnification of the region where traces of glutamate would be expected.

Table S14. Normalized and concentration-corrected integrals values before and after reaction for keto and hydroxy acids in the competition experiment of **K2**/**K4** with $NaBH_3CN$ used to calculate the pH-dependent competition constants. For pH 4 and 5 acetate buffer was used; for pH 6 and 7 phosphate buffer. Experiment: RM03-432.

	pH	[buffer] / M	[**K4**]$_0$	[**K2**]$_0$	[**H4**]$_t$	[**H2**]$_t$	[**A2**]$_t$
before reaction			0.945	0.825	--	--	--
after reaction	4	0.5			0.152	0.253	0.143
	5	0.5	--		0.131	0.253	0.151
	6	0.5			0.092	0.269	0.167
	7	0.5			0.046	0.298	0.186

Table S15. Amounts of keto acids after reaction due to direct reduction ($[\mathbf{K}]_{t,HO}$) or reductive amination ($[\mathbf{K}]_{t,AA}$).

pH	[buffer] / M	$[\mathbf{K4}]_{t,HO}$ = $[\mathbf{K4}]_0 - [\mathbf{H4}]_t$	$[\mathbf{K1}]_{t,HO}$ = $[\mathbf{K1}]_0 - [\mathbf{H1}]_t$	$[\mathbf{K1}]_{t,AA}$ = $[\mathbf{K1}]_0 - [\mathbf{A1}]_t$
4	0.5	0.792	0.572	0.682
5	0.5	0.814	0.572	0.674
6	0.5	0.853	0.557	0.659
7	0.5	0.898	0.528	0.639

Table S16. Logarithm of the ratio of keto acid before and after reaction.

pH	[buffer] / M	$\lg([\mathbf{K4}]_0/[\mathbf{K4}]_{t,HO})$	$\lg([\mathbf{K1}]_0/[\mathbf{K1}]_{t,HO})$	$\lg([\mathbf{K1}]_0/[\mathbf{K1}]_{t,AA})$
4	0.5	0.076	0.159	0.083
5	0.5	0.065	0.159	0.088
6	0.5	0.044	0.171	0.098
7	0.5	0.022	0.194	0.111

Table S17. Competition constants for reduction (to yield hydroxy acids) or reductive amination (to yield amino acids) relative to the keto acid reduction of **K4**.

		$\kappa = \frac{k_2\,(\mathbf{K4})_{reduction}}{k_2\,(\mathbf{K4})_{reduction}}$	$\kappa = \frac{k_2\,(\mathbf{K2})_{reduction}}{k_2\,(\mathbf{K4})_{reduction}}$	$\kappa = \frac{k_2\,(\mathbf{K2})_{red.\ amin.}}{k_2\,(\mathbf{K4})_{reduction}}$
pH	[buffer] / M	$\lg([\mathbf{K4}]_0/[\mathbf{K4}]_{t,HO})$/ $\lg([\mathbf{K4}]_0/[\mathbf{K4}]_{t,HO})$	$\lg([\mathbf{K2}]_0/[\mathbf{K2}]_{t,HO})$/ $\lg([\mathbf{K4}]_0/[\mathbf{K4}]_{t,HO})$	$\lg([\mathbf{K2}]_0/[\mathbf{K2}]_{t,AA})$/ $\lg([\mathbf{K4}]_0/[\mathbf{K4}]_{t,HO})$
4	0.5	1.000	2.082	1.085
5	0.5	1.000	2.462	1.359
6	0.5	1.000	3.860	2.212
7	0.5	1.000	8.925	5.098

Thermochemistry of Reductive Amination

Equilibrium constants for many biochemical reactions have been determined from enzymatically catalyzed reactions and a meta-analysis of literature data has been compiled by Miller and Smith-Magowan in Ref. S13.

However, only for the oxidative deamination (the reverse reaction of reductive amination) of glutamate and alanine with NAD^+ equilibrium constants have been determined:

Reported Experimental Equilibria for Oxidative Deamination/Reductive Amination Reactions

Reaction of glutamate with NAD^+:

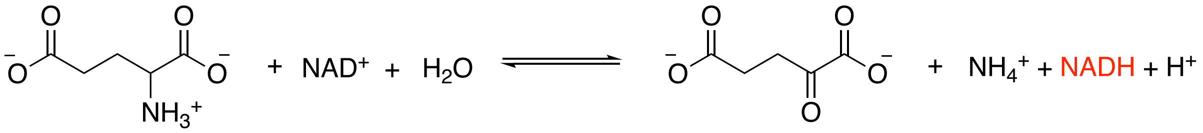

$\Delta G^0 = +74.80 \pm 0.80$ kJ mol^{-1} (25 °C, pH = 7.4).[S13,S14]

Reaction of alanine with NAD^+:

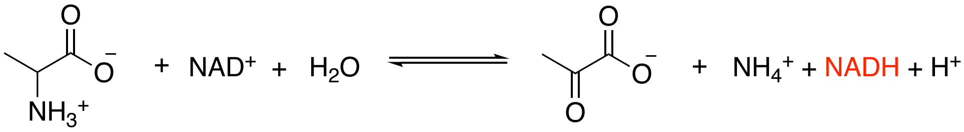

$\Delta G^0 = +75.15 \pm 0.80$ kJ mol^{-1} (25 °C, pH = 7.4).[S13,S15]

To determine the energetics for the analogous oxidative deamination reactions of glycine and aspartate (which have not been experimentally characterized directly), thermodynamic cycles can be employed. Therefore, we can combine the known energetics for the transamination reactions of amino and keto acids with those for the two oxidative deamination reactions shown above. Specifically, we utilize the following two transamination equilibria in our analysis:

Reported Experimental Equilibria for Transamination Reactions

Reaction of glutamate with oxaloacetate:

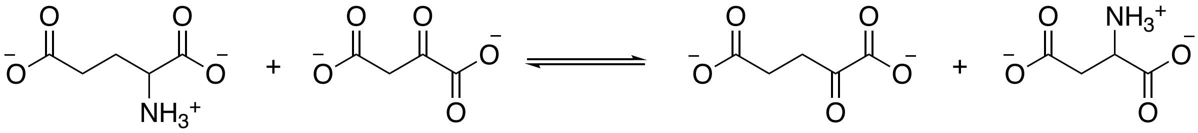

$\Delta G^0 = -4.73 \pm 0.12$ kJ mol^{-1} (25 °C, pH = 7.4).[S16]

Reaction of aspartic acid with glyoxylate hydrate:

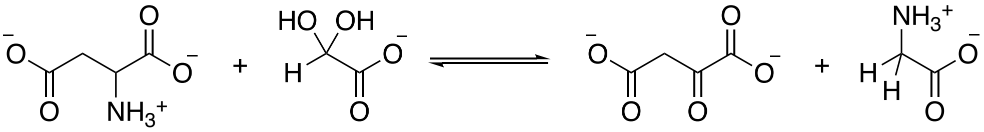

$\Delta G^0 = -10.2 \pm 1.2$ kJ mol^{-1} (precise experimental conditions unknown).[S13]

Combination of the transamination equilibrium of α-ketoglutarate and aspartate with the oxidation of glutamate allows to calculate the energetics for the oxidation of aspartate:

Reaction of aspartate with NAD⁺:

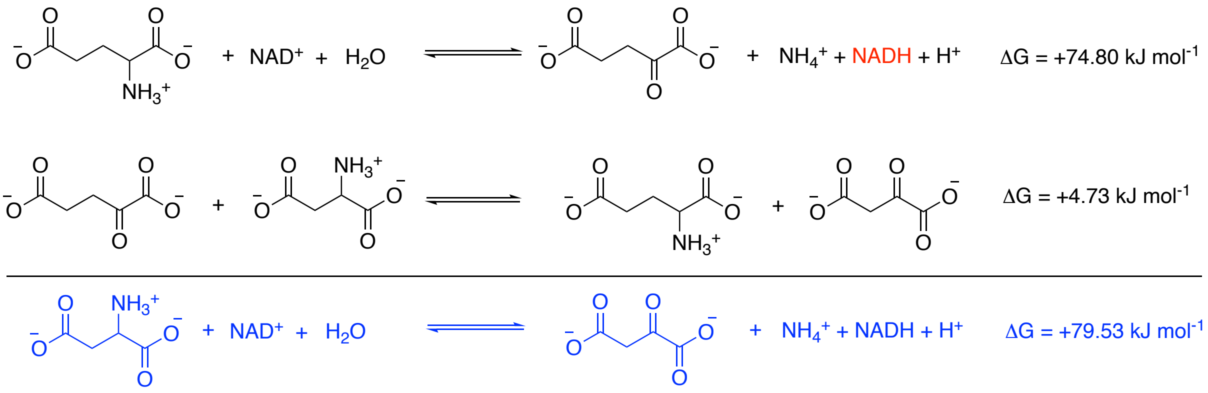

As the transamination equilibrium between aspartate and glyoxylate is known, combination with the oxidative deamination reaction of aspartate affords the equilibrium for the oxidative deamination of glycine:

Reaction of glycine with NAD⁺:

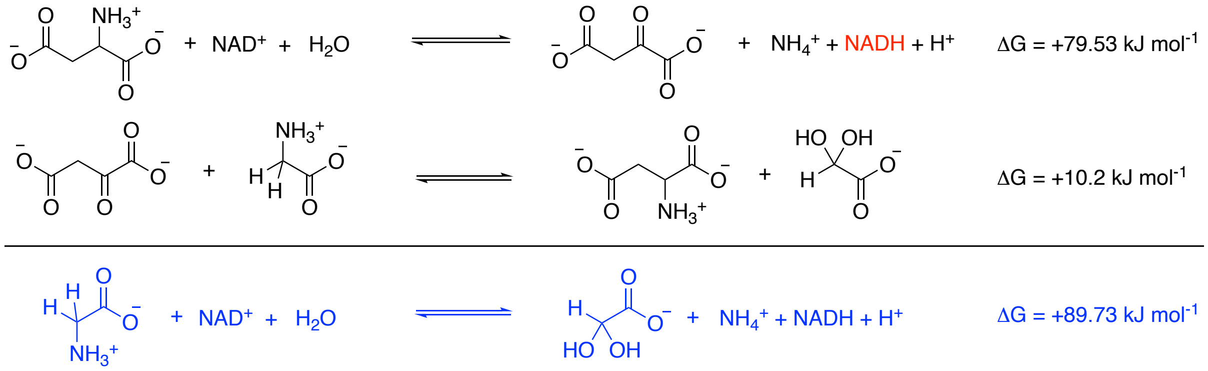

DFT Computations

Initially, all structures were subjected to a conformational search using the OPLS3[S17] force field as implemented in Macromodel.[S18] All obtained conformers were then optimized with the Gaussian 16 software package[S19] at the SMD(water)[S20]/MN15[S21]/def2-TZVPD[S22] level of theory, which was also used for the localization of the transition states. Frequency analysis at the same level considering Grimmes quasi-harmonic corrections were performed to confirm that all structures correspond to minima.[S23] Gibbs energies for unique conformers were Boltzmann weighted and, finally, a free energy change of +7.92 kJ/mol (= R · 298 K · ln(24.47 L mol^{-1}/L mol^{-1})) was applied for their conversion from gas phase (1 atm) to liquid phase (1 M). Structures were visualized with CYLView.[S24]

Computation of p$K_{a,DFT}$ Values

To calculate p$K_{a,DFT}$ values from quantum-chemically calculated energies we applied a procedure outlined by Schlegel that will be described briefly:[S25] p$K_{a,DFT}$ values are related to the Gibbs free energy of deprotonation in aqueous solution according to eq. (28)

$$pK_{a,DFT} = \frac{\Delta G^*_{aq}}{2.303RT} \quad (28)$$

with

$$\Delta G^*_{aq} = G^*_{aq}(A) + G^*_{aq}(H^+) - G^*_{aq}(AH) \quad (29).$$

In eq. (29), $G^*_{aq}(X)$ correspond to the aqueous phase free energies of the acid AH, the anion A and the proton H$^+$. $G^*_{aq}(A)$ and $G^*_{aq}(AH)$ are obtained from DFT calculations. The free energy $G^*_{aq}(H^+)$ can be derived from experiments:

$$G^*_{aq}(H^+) = G^\circ_g(H^+) + \Delta G_{aq,solv}(H^+) - \Delta G^{1atm\rightarrow 1M} \quad (30).$$

In (30), $\Delta G_{aq,solv}(H^+) = -265.9\frac{kcal}{mol} \equiv -1112.5\frac{kJ}{mol}$,[S26] $G^0_g(H^+) = -26.3\frac{kJ}{mol}$,[S27] and $\Delta G^{1atm\rightarrow 1M} = 7.92\frac{kJ}{mol}$ (= R · 298 K · ln(24.47 L mol^{-1}/L mol^{-1})).

Finally, a correlation of p$K_{a,exp}$ vs. p$K_{a,DFT}$ for a series of reference compounds (Tables S18) was used to correct the computational p$K_{a,DFT}$ values of further substrates (Figure S31). Based on the errors obtained from the regression analysis in Figure S31, the prediction accuracy of the acidities e.g. of iminium ions can be approximated under the assumption that there is a random error between p$K_{a,exp}$ and p$K_{a,DFT}$. The major limitations of this approach is the only limited amount of C=N-H acids for which accurate acidity measurements are available. However, the quality of the correlation obtained in Figure S31 using experimental p$K_{a,exp}$ values of multiple different sources is relatively good, thus indicating the feasibility of the approach.

Reference Compounds for pK_a Determination of C=N-H Acids

Table S18. C=N compounds **R** used as reference compounds for the calibration of the pK_a predictions.

Number	Structure	p$K_{a,expt}$	p$K_{a,DFT}$	Reference
**R1**	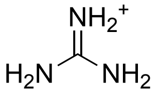	13.6	17.3	Ref. S28
**R2**	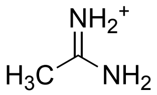	12.41	14.0	Ref. S29
**R3**	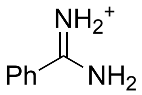	11.6	12.8	Ref. S30
**R4**	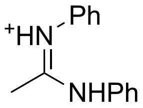	8.298	8.6	Ref. S29
**R5**	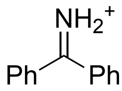	7.2	7.5	Ref. S31
**R6**	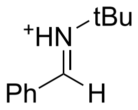	6.7	7.0	Ref. S32
**R7**	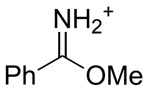	5.6	6.1	Ref. S33
**R8**	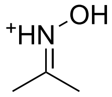	1.92	-0.1	Ref. S34
**R9**	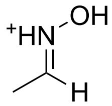	1.75	-3.2	Ref. S35
**R10**	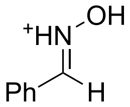	-1.20	-4.5	Ref. S36
**R11 E**	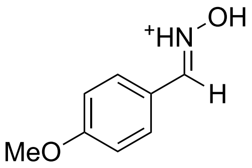	-0.55	-3.2	Ref. S37
**R11 Z**	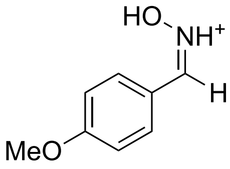	0.80	-1.09	Ref. S37
**R12**	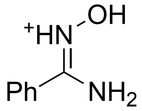	4.85	3.8	Ref. S38
**R13**	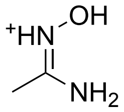	5.78	5.37	Ref. S38

Table S19. Raw data from DFT calculations at the SMD(H_2O)/MN15/def2-TZVPD level (in Hartree) used for the calculation of p$K_{a,DFT}$ values.

Species	Filename	E0(SCF)	G_Grimme	weighting	p$K_{a,DFT}$
**R1**	r1_1.log	-205.717984	-205.658808	0.2402	
	r1_2.log	-205.717931	-205.659895	0.7598	
		weighted	-205.659634		
**R1** anion	r1_an_1.log	-205.239724	-205.191282	0.4653	
	r1_an_2.log	-205.239496	-205.191413	0.5347	
		weighted	-205.191352		17.27
**R2**	r2_1.log	-189.658261	-189.586561	1.0000	
		weighted	-189.586561		
**R2** anion	r2_an_b_1.log	-189.183628	-189.125340	1.0000	
		weighted	-189.125340		14.02
**R3**	r3_1.log	-381.223545	-381.102784	1.0000	
		weighted	-381.102784		
**R3** anion	r3_an_a_1.log	-380.751046	-380.643451	0.2597	
	r3_an_b_1.log	-380.752244	-380.644439	0.7403	
		weighted	-380.644183		12.81
**R4**	r4_a_1.log	-651.324257	-651.101838	0.5243	
	r4_a_2.log	-651.323579	-651.101344	0.3106	
	r4_b_3.log	-651.323015	-651.100748	0.1651	
		weighted	-651.101504		
**R4** anion	r4_an_a_1.log	-650.859522	-650.650966	0.1069	
	r4_an_a_2.log	-650.859955	-650.651405	0.1702	
	r4_an_b_1.log	-650.861291	-650.652599	0.6041	
	r4_an_b_2.log	-650.860058	-650.651065	0.1188	
		weighted	-650.652039		8.61
**R5**	r5_1.log	-556.720389	-556.538472	1.0000	
		weighted	-556.538472		
**R5** anion	r5_an_1.log	-556.260090	-556.091517	1.0000	
		weighted	-556.091517		7.46
**R6**	r6_a_1.log	-482.967922	-482.756760	1.0000	
	r6_b_1.log	-482.955823	-482.743397	0.0000	
		weighted	-482.756760		
**R6** anion	r6_an_a_1.log	-482.508308	-482.310813	0.4892	
	r6_an_a_2.log	-482.507580	-482.310853	0.5108	
	r6_an_b_1.log	-482.494202	-482.296010	0.0000	
		weighted	-482.310834		6.98
**R7**	r7_1.log	-440.336919	-440.200697	0.9952	
	r7_2.log	-440.331263	-440.195665	0.0048	
		weighted	-440.200673		
**R7** anion	r7_an_a_1.log	-439.879023	-439.756113	0.3233	
	r7_an_b_1.log	-439.880312	-439.756809	0.6767	
		weighted	-439.756584		6.14
**R8**	r8_1.log	-248.711259	-248.626949	0.9334	

	r8_2.log	-248.709441	-248.624459	0.0666	
		weighted	-248.626783		
**R8** anion	r8_an_1.log	-248.268987	-248.196343	0.9908	
	r8_an_2.log	-248.264964	-248.191933	0.0092	
		weighted	-248.196303		-0.12
**R9**	r9_a_1.log	-209.422795	-209.363584	0.3184	
	r9_a_2.log	-209.422097	-209.362333	0.0845	
	r9_b_1.log	-209.423403	-209.363931	0.4601	
	r9_b_2.log	-209.421920	-209.362789	0.1370	
		weighted	-209.363529		
**R9** anion	r9_an_a_1.log	-208.986327	-208.939915	0.5017	
	r9_an_a_2.log	-208.983379	-208.936803	0.0185	
	r9_an_b_1.log	-208.986817	-208.939857	0.4722	
	r9_an_b_2.log	-208.982830	-208.935963	0.0076	
		weighted	-208.939800		-3.22
**R10**	r10_a_2.log	-400.990744	-400.881644	0.0863	
	r10_b_1.log	-400.991753	-400.883869	0.9137	
		weighted	-400.883677		
**R10** anion	r10_an_a_1.log	-400.557631	-400.462719	0.9908	
	r10_an_b_1.log	-400.554509	-400.458303	0.0092	
	r10_an_b_2.log	-400.549435	-400.453436	0.0001	
		weighted	-400.462678		-4.48
**R11 (E)**	r11_a_3.log	-515.448168	-515.311154	0.6990	
	r11_a_4.log	-515.448649	-515.310359	0.3010	
		weighted	-515.310914		
**R11 (E)** anion	r11_an_a_1.log	-515.011828	-514.887051	0.3701	
	r11_an_a_2.log	-515.012129	-514.887474	0.5791	
	r11_an_a_3.log	-515.008738	-514.884016	0.0148	
	r11_an_a_4.log	-515.008906	-514.884852	0.0359	
		weighted	-514.887172		-3.22
**R11 (Z)**	r11_b_1.log	-515.450085	-515.312789	0.3615	
	r11_b_2.log	-515.450327	-515.312400	0.2394	
	r11_b_3.log	-515.450339	-515.312272	0.2091	
	r11_b_4.log	-515.450178	-515.312182	0.1900	
		weighted	-515.312472		
**R11 (Z)** anion	r11_an_b_1.log	-515.009513	-514.884285	0.6177	
	r11_an_b_2.log	-515.009592	-514.883826	0.3796	
	r11_an_b_3.log	-515.003950	-514.878638	0.0016	
	r11_an_b_4.log	-515.003931	-514.878305	0.0011	
		weighted	-514.884096		-1.09
**R12**	r12_a_1.log	-456.347619	-456.223640	0.3263	
	r12_a_2.log	-456.347609	-456.224323	0.6731	
	r12_b_1.log	-456.340081	-456.216999	0.0003	
	r12_b_2.log	-456.340946	-456.216957	0.0003	
		weighted	-456.224096		

**R12** anion	r12_an_a_1.log	-455.895227	-455.785100	0.9999	
	r12_an_b_1.log	-455.886283	-455.776454	0.0001	
	r12_an_b_2.log	-455.883089	-455.773270	0.0000	
		weighted	-455.785099		3.80
**R13**	r13_a_1.log	-264.783216	-264.709065	0.9883	
	r13_b_1.log	-264.778969	-264.704883	0.0117	
		weighted	-264.709016		
**R13** anion	r13_an_a_1.log	-264.327566	-264.266605	0.9976	
	r13_an_b_1.log	-264.322006	-264.260932	0.0024	
	r13_an_b_2.log	-264.317526	-264.256067	0.0000	
		weighted	-264.266591		5.37

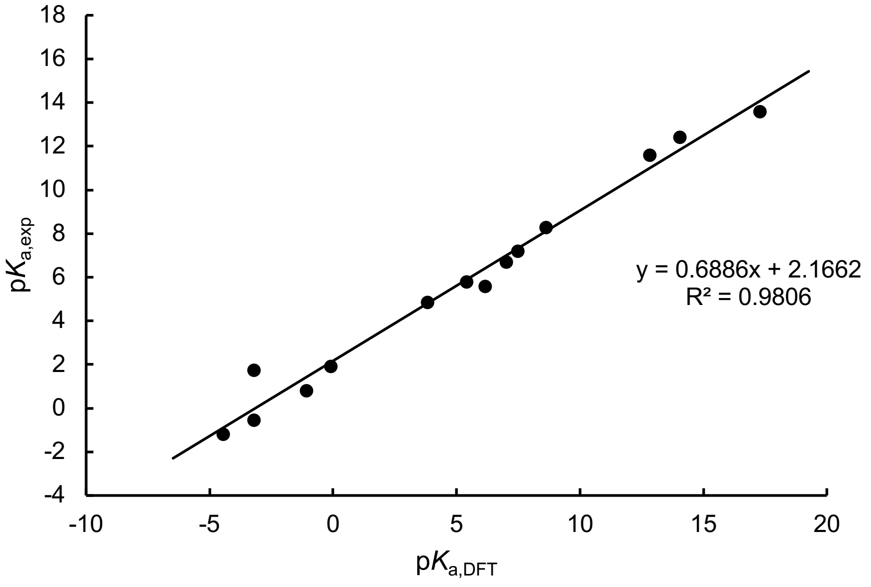

Figure S31. Correlation of p$K_{a,exp}$ vs. p$K_{a,DFT}$ for the reference compounds (Tables S18) used to correct the computational p$K_{a,DFT}$ values of further substrates. Error analysis: Slope: 0.689 ± 0.028; intercept: 2.17 ± 0.23

Acidity of Iminium Ions

Table S20. Raw data from DFT calculations at the SMD(H2O)/MN15/def2-TZVPD level (in Hartree) used for the calculation of p$K_{a,DFT}$ values of iminium ions **I**-H^+.

Species	Filename	E^0 (hartree)	G^0 (hartree)	weighting	p$K_{a,DFT}$	p$K_{a,corrDFT}$[a]
**I1**-H^+	i1_h_1.log	-322.328686	-322.273863	1.0000		
		weighted	-322.273863			
**I1**	i1_1.log	-321.868408	-321.825508	0.9634		
	i1_2.log	-321.864479	-321.822422	0.0366		
		weighted	-321.825395		8.15	7.8 ± 0.3
**I2**-H^+	i2_h_1.log	-283.037615	-283.008755	1.0000		
		weighted	-283.008755			
**I2**	i2_1.log	-282.581760	-282.565461	0.0477		
	i2_2.log	-282.584922	-282.568284	0.9523		
		weighted	-282.568150		4.54	5.3 ± 0.3
**I3**-H^+	i3_h_1.log	-510.354086	-510.300851	1.0000		
		weighted	-510.300851			
**I3**	i3_1.log	-509.887545	-509.845875	0.0261		
	i3_2.log	-509.890916	-509.849098	0.7955		
	i3_5.log	-509.889035	-509.847688	0.1784		
		weighted	-509.848762		9.82	8.9 ± 0.4
**I4**-H^+	i4_h_1.log	-549.628375	-549.548138	1.0000		
		weighted	-549.548138			
**I4**	i4_1.log	-549.164298	-549.096406	0.2895		
	i4_2.log	-549.165433	-549.097114	0.6131		
	i4_3.log	-549.163540	-549.094752	0.0501		
	i4_4.log	-549.162135	-549.093427	0.0123		
	i4_5.log	-549.162268	-549.094167	0.0269		
	i4_6.log	-549.161046	-549.093028	0.0081		
		weighted	-549.096633		9.55	8.7 ± 0.4

[a] calculated from p$K_{a,DFT}$ using the correlation in Figure S31. The error is estimated from a calculation of the error propagation.

Gibbs Energy Profile for Imine Formation

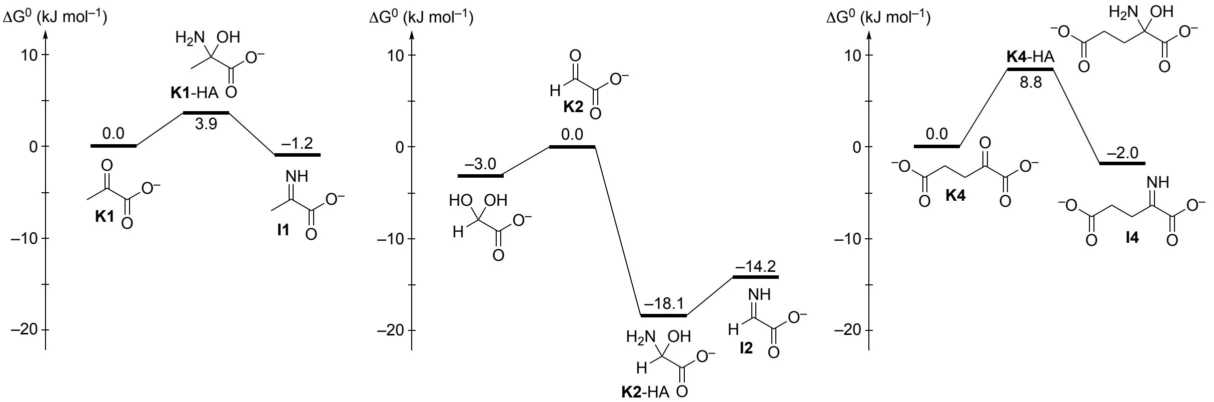

Figure S32. Gibbs energy profile for hemiaminal/imine formation from α-keto acids and ammonia.

Table S21. Raw data from DFT calculations at the SMD(H2O)/MN15/def2-TZVPD level (in Hartree) for formation of imines **I** and relative energies $G^0_{rel.}$ (in kJ mol^{-1}).

Species	Filename	E^0 (hartree)	G^0 (hartree)	weighting	$G^0_{rel.}$
NH_3	nh3.log	-56.516396	-56.501446		
H_2O	h2o.log	-76.397036	-76.394171		
Pyruvate (**K1**) + NH_3					
**K1**	k1_an_1.log	-341.746212	-341.717655	1.0000	
		weighted	-341.717655		
**K1**-HA	k1_ha_1.log	-398.282686	-398.214603		3.9
**I1**	i1_1.log	-321.868408	-321.825508	0.9634	
	i1_2.log	-321.864479	-321.822422	0.0366	
		weighted	-321.825395		-1.2
Glyoxylate (**K2**) + NH_3					
**K2**	k2_an_1.log	-302.458191	-302.455483	0.4996	
**K2**-Hydrate	k2_hydrate_1.log	-378.873065	-378.843619	0.0060	
	k2_hydrate_2.log	-378.878051	-378.847866	0.5405	
	k2_hydrate_3.log	-378.877507	-378.847701	0.4535	
		weighted	-378.847765		-3.0
**K2**-HA	k2_ha_1.log	-359.002943	-358.960814	0.4998	
	k2_ha_2.log	-359.003186	-358.960815	0.5002	
		weighted	-358.960815		-18.1
**I2**	i2_1.log	-282.581760	-282.565461	0.0477	
	i2_2.log	-282.584922	-282.568284	0.9523	
		weighted	-282.568150		-14.2
α-Ketoglutarate (**K2**) + NH_3					
**K4**	k4_an_1.log	-569.042948	-568.988845	0.8150	
	k4_an_2.log	-569.041406	-568.987446	0.1850	
		weighted	-568.988586		
**K4**-HA	k4_ha_1.log	-625.580149	-625.486128	0.0817	
	k4_ha_2.log	-625.579011	-625.486562	0.1294	
	k4_ha_3.log	-625.578623	-625.484601	0.0162	
	k4_ha_4.log	-625.578457	-625.485521	0.0429	
	k4_ha_5.log	-625.578362	-625.485640	0.0487	
	k4_ha_6.log	-625.580597	-625.487221	0.2603	
	k4_ha_8.log	-625.581108	-625.487384	0.3095	
	k4_ha_9.log	-625.575438	-625.481755	0.0008	

	k4_ha_10.log	-625.577223	-625.483707	0.0063	
	k4_ha_12.log	-625.578119	-625.485717	0.0529	
	k4_ha_13.log	-625.578163	-625.485391	0.0374	
	k4_ha_14.log	-625.579820	-625.484449	0.0138	
		weighted	-625.486692		8.8
**I4**	i4_1.log	-549.164298	-549.096406	0.2895	
	i4_2.log	-549.165433	-549.097114	0.6131	
	i4_3.log	-549.163540	-549.094752	0.0501	
	i4_4.log	-549.162135	-549.093427	0.0123	
	i4_5.log	-549.162268	-549.094167	0.0269	
	i4_6.log	-549.161046	-549.093028	0.0081	
		weighted	-549.096633		-2.0

In-Situ IR Spectroscopy

In-situ IR spectroscopy was performed with a Mettler Toledo ReactIR spectrometer equipped with a 6.3 mm diamond ATR probe. Pyruvate was dissolved in aqueous solutions of ammonia prepared from a highly concentrated stock solution. All spectra were subjected to a solvent subtraction of water and a second-order baseline correction.

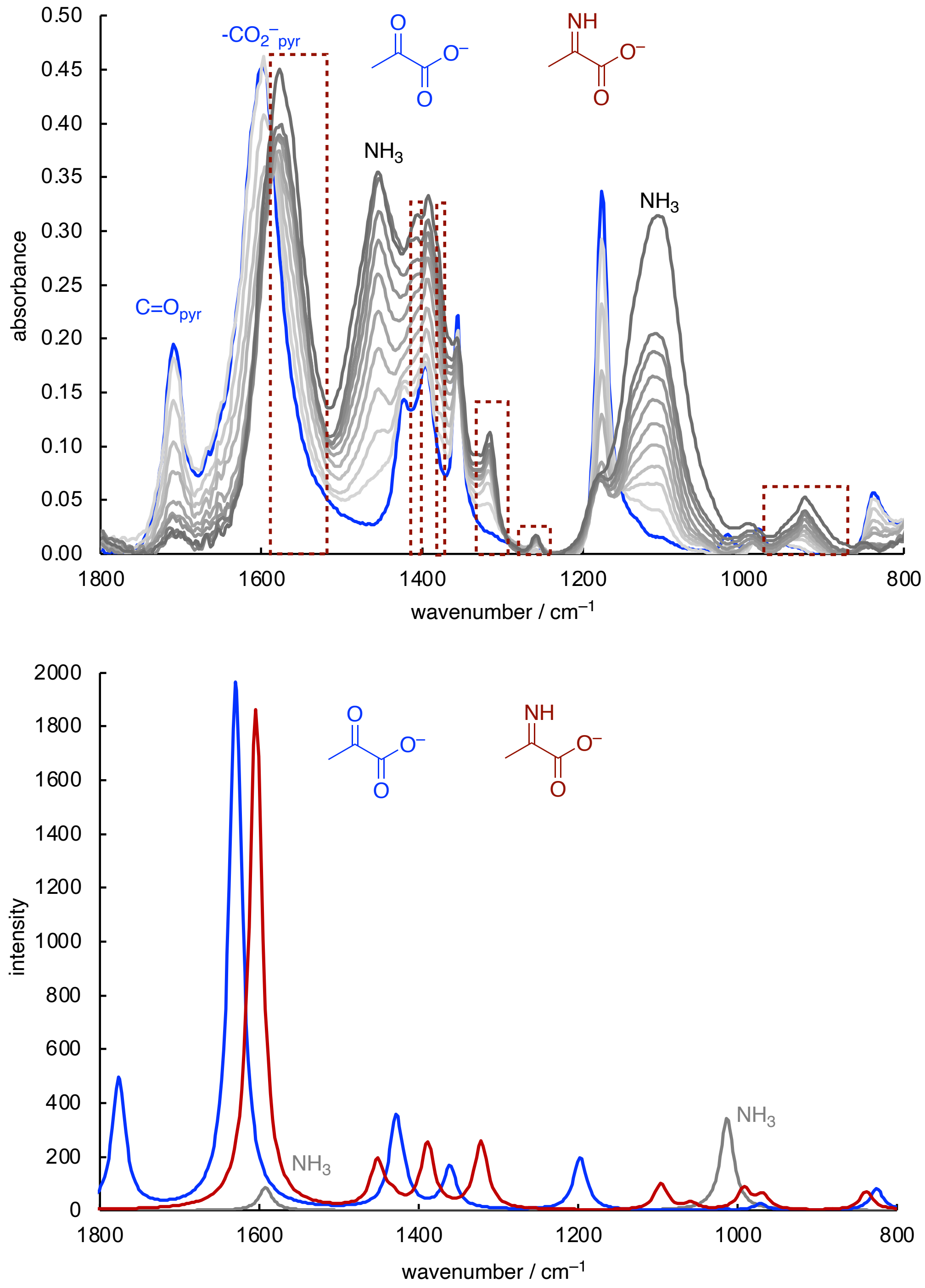

Figure S33. Top: In-situ IR spectra of a solution of pyruvate (2 M) with increasing concentration of an ammonia/ammonium buffer at pH 10.2 (0 – 15 M; spectrum in the absence of ammonia in blue). The vibrations inside the red boxes correspond to newly appearing species with increasing ammonia concentration. Bottom: Computed IR spectra based on structures optimized at the SMD(H_2O)/MN15/def2-TZVPD level of theory (Table S21; structures: i1_1.log; k1_an_1.log; nh3.log).

Geometries of Optimized Structures

Filename (cf. the Table S19-21), cartesian coordinates (in Å), electronic energies (in Hartree), enthalpies (in Hartree), Gibbs free energies (in Hartree; from the rigid-rotor approximation as default in Gaussian. Gibbs energies in Tables S19-21 use Grimmes quasi-harmonic corrections), and the number of the imaginary vibrational frequencies (NImag) for all stationary points at the SMD(H_2O)/MN15/def2-TZVP level of theory.

```
r10_a_2.log
E =       -400.990744
H =       -400.840893
G =       -400.881801
NImag=0
C         0.8424815         1.5174564        -0.4010065
H         0.4909357         2.4938885        -0.7208106
N         2.1134999         1.3788490        -0.2938043
O         3.0271084         2.3386502        -0.5649300
C        -0.1203688         0.4783432        -0.1283728
C         0.2341897        -0.8380309         0.1972745
C        -0.7575372        -1.7677959         0.4433675
C        -2.1006665        -1.3970288         0.3673991
C        -2.4568905        -0.0941319         0.0416575
C        -1.4680672         0.8443414        -0.2100053
H         2.5722627         0.5227471         0.0212868
H         2.5659305         3.0902101        -0.9828042
H         1.2725045        -1.1415842         0.2501565
H        -0.4898018        -2.7861356         0.6912888
H        -2.8700836        -2.1335982         0.5604282
H        -3.4993122         0.1882639        -0.0191088
H        -1.7250852         1.8643557        -0.4685164
```

```
r10_an_a_1.log
E =       -400.557631
H =       -400.421737
G =       -400.463102
NImag=0
C         0.6783174         1.6896372        -0.4491301
H         0.2243803         2.6463258        -0.7047353
N         1.9480083         1.5730653        -0.4161472
O         2.5753657         2.7682578        -0.7336687
C        -0.2086619         0.5641047        -0.1505612
C         0.2724188        -0.7050325         0.1883474
C        -0.6142940        -1.7337836         0.4611277
C        -1.9896057        -1.5124179         0.4000563
C        -2.4739689        -0.2546492         0.0641722
C        -1.5860675         0.7789560        -0.2098996
H         3.5229027         2.5761078        -0.6810349
H         1.3403515        -0.8774373         0.2359066
H        -0.2357162        -2.7138978         0.7228669
H        -2.6777921        -2.3203896         0.6143080
H        -3.5407913        -0.0767837         0.0151600
H        -1.9555471         1.7637369        -0.4728682
```

r10_an_b_1.log
E = -400.554509
H = -400.417901
G = -400.458947
NImag=0

C	0.8515919	1.6176986	-0.3121009
H	0.3156764	2.5515916	-0.4520008
N	2.1198119	1.7719032	-0.3771328
O	2.8448249	0.6079259	-0.2045495
C	0.0001934	0.4445128	-0.0892277
C	0.4316123	-0.8737927	0.1185488
C	-0.4973608	-1.8871443	0.3170846
C	-1.8606781	-1.6141728	0.3132907
C	-2.3011414	-0.3111305	0.1083396
C	-1.3781004	0.7034046	-0.0901801
H	3.7696119	0.8838176	-0.2795074
H	1.4824848	-1.1131110	0.1263070
H	-0.1499891	-2.9002943	0.4762672
H	-2.5752717	-2.4125219	0.4691922
H	-3.3601714	-0.0865531	0.1032805
H	-1.7187946	1.7201662	-0.2502114

r10_an_b_2.log
E = -400.549435
H = -400.413193
G = -400.453711
NImag=0

C	0.9772661	1.5970009	-0.7910142
H	0.5025546	2.3584721	-1.4014174
N	2.2113765	1.8307443	-0.5529372
O	2.8830223	0.9514429	0.2725381
C	0.1307487	0.4851320	-0.3212620
C	0.5546410	-0.8472673	-0.3284393
C	-0.3019446	-1.8540140	0.0997306
C	-1.5834106	-1.5423537	0.5383439
C	-2.0176543	-0.2201296	0.5315454
C	-1.1690704	0.7859557	0.0939761
H	2.2551084	0.3215595	0.6756379
H	1.5406876	-1.1029764	-0.6985830
H	0.0322532	-2.8834861	0.0839721
H	-2.2470522	-2.3291085	0.8740614
H	-3.0199033	0.0245020	0.8593619
H	-1.5059230	1.8159261	0.0715855

r10_b_1.log
E = -400.991753
H = -400.842467
G = -400.884464
NImag=0

C	0.9683718	1.5021159	0.0726237
H	0.8274431	2.3610680	0.7183122
N	2.0097014	1.6173397	-0.6723931
O	2.3796507	0.6800637	-1.5706175
C	0.0169965	0.4262376	0.1569852
C	0.0075248	-0.7237740	-0.6506154

C	-0.9707543	-1.6825911	-0.4544605
C	-1.9367692	-1.5152425	0.5352264
C	-1.9360632	-0.3787223	1.3371006
C	-0.9669048	0.5894871	1.1461219
H	2.5955112	2.4535204	-0.6313410
H	3.2830269	0.9017199	-1.8600331
H	0.7471441	-0.8649841	-1.4224791
H	-0.9829041	-2.5675410	-1.0766886
H	-2.6948191	-2.2746277	0.6787538
H	-2.6881808	-0.2494055	2.1037224
H	-0.9546750	1.4825360	1.7592821

r11_a_3.log
E = -515.448168
H = -515.263737
G = -515.311951
NImag=0

C	0.7486174	0.4242230	-0.3548276
C	1.9379690	1.1954615	-0.5585041
H	2.8999484	0.7089494	-0.4273087
N	1.9727326	2.4392188	-0.8899475
O	3.1032064	3.1662834	-1.0819524
C	-0.5529726	0.9288129	-0.4913886
C	-1.6464128	0.1173467	-0.2768452
C	-1.4522983	-1.2248610	0.0812061
C	-0.1583211	-1.7400817	0.2205917
C	0.9261487	-0.9228856	0.0045072
O	-2.4607178	-2.0801186	0.3075552
C	-3.7971972	-1.6065111	0.1791569
H	1.1470892	3.0173509	-1.0453451
H	3.8729386	2.5880475	-0.9264904
H	-0.7257295	1.9617145	-0.7669685
H	-2.6420366	0.5218924	-0.3862530
H	-0.0361755	-2.7787450	0.4976246
H	1.9317344	-1.3116647	0.1097299
H	-4.4415013	-2.4513697	0.4032874
H	-3.9896631	-0.7999499	0.8882600
H	-3.9868588	-1.2600138	-0.8378879

r11_a_4.log
E = -515.448649
H = -515.263950
G = -515.310941
NImag=0

C	0.5331814	0.6720921	-0.4184319
C	1.4822479	1.7071300	-0.6919562
H	1.1243378	2.7098470	-0.9064803
N	2.7607849	1.5518028	-0.7026120
O	3.6582636	2.5401103	-0.9495333
C	0.8879434	-0.6443583	-0.0652434
C	-0.0878284	-1.5760026	0.1760674
C	-1.4450764	-1.2228637	0.0723709
C	-1.8127334	0.0794726	-0.2733453
C	-0.8208857	1.0130204	-0.5123146
O	-2.3242474	-2.2028387	0.3276865

C	-3.7140596	-1.9081686	0.2405002
H	3.2341608	0.6625857	-0.5444260
H	3.1883323	3.3930635	-0.9028039
H	1.9267020	-0.9364689	0.0281105
H	0.1620203	-2.5919101	0.4523266
H	-2.8514997	0.3650098	-0.3538872
H	-1.0917825	2.0272154	-0.7799594
H	-4.2358457	-2.8296200	0.4809514
H	-3.9885377	-1.1323771	0.9570659
H	-3.9767781	-1.5894418	-0.7694859

r11_an_a_1.log
E = -515.011828
H = -514.840571
G = -514.887807
NImag=0

C	0.2669536	0.9297089	-0.7357713
C	1.2022021	2.0510623	-0.7443312
H	0.7895416	3.0565320	-0.6646560
N	2.4642988	1.8883694	-0.8406574
O	3.1309004	3.1089834	-0.8254511
C	0.6736939	-0.4019353	-0.8315850
C	-0.2501941	-1.4356043	-0.8193780
C	-1.6114081	-1.1416013	-0.7094236
C	-2.0327869	0.1846250	-0.6128610
C	-1.0998797	1.2047743	-0.6263826
O	-2.5846798	-2.0880733	-0.6893475
C	-2.1894752	-3.4476108	-0.7858308
H	4.0688767	2.8831019	-0.9023586
H	1.7278767	-0.6345935	-0.9169931
H	0.0914091	-2.4583452	-0.8950049
H	-3.0918277	0.3932224	-0.5283354
H	-1.4264507	2.2358448	-0.5513859
H	-3.1018253	-4.0367428	-0.7519631
H	-1.6690280	-3.6370307	-1.7270820
H	-1.5448975	-3.7270871	0.0502983

r11_an_a_2.log
E = -515.012129
H = -514.840947
G = -514.888265
NImag=0

C	0.3401345	0.8411357	-0.7453329
C	1.1629467	2.0470865	-0.7417924
H	0.6582971	3.0091918	-0.6579324
N	2.4349956	2.0008021	-0.8329110
O	2.9906449	3.2752576	-0.8072627
C	0.8809782	-0.4487458	-0.8483206
C	0.0591621	-1.5547783	-0.8468742
C	-1.3289966	-1.4023862	-0.7422509
C	-1.8837076	-0.1302713	-0.6391951
C	-1.0414173	0.9769703	-0.6420095
O	-2.0576113	-2.5476079	-0.7492914
C	-3.4680827	-2.4347417	-0.6450630
H	3.9451731	3.1320849	-0.8811124

H	1.9532149	-0.5762861	-0.9296310
H	0.4673220	-2.5546953	-0.9259349
H	-2.9524282	0.0094865	-0.5575144
H	-1.4689617	1.9701479	-0.5619371
H	-3.8614807	-3.4472798	-0.6687730
H	-3.7527829	-1.9552505	0.2941178
H	-3.8769003	-1.8654203	-1.4827789

r11_an_a_3.log
E = -515.008738
H = -514.837448
G = -514.885131
NImag=0

C	0.4221382	0.8410233	-0.7510453
C	1.4365116	1.8916870	-0.7687342
H	1.0858209	2.9240337	-0.6919734
N	2.6837459	1.6404713	-0.8685798
O	3.5121017	2.7477134	-0.8703905
C	0.7326383	-0.5164331	-0.8414430
C	-0.2630807	-1.4809337	-0.8206963
C	-1.5992753	-1.0896952	-0.7075440
C	-1.9240229	0.2639735	-0.6163426
C	-0.9207278	1.2145648	-0.6383226
O	-2.6378503	-1.9634185	-0.6792078
C	-2.3420870	-3.3484150	-0.7699554
H	2.9712491	3.5563917	-0.7916556
H	1.7670493	-0.8243376	-0.9292937
H	0.0042795	-2.5258515	-0.8922615
H	-2.9650887	0.5482854	-0.5292205
H	-1.1721879	2.2667597	-0.5674995
H	-3.2944561	-3.8699557	-0.7298069
H	-1.8403723	-3.5794603	-1.7120529
H	-1.7161857	-3.6694031	0.0654254

r11_an_a_4.log
E = -515.008906
H = -514.838065
G = -514.885927
NImag=0

C	0.6261384	0.6202808	-0.7764485
C	1.8112954	1.4724698	-0.8161127
H	2.7782644	0.9719765	-0.9089086
N	1.7413300	2.7449846	-0.7469172
O	2.9503212	3.4140581	-0.7974930
C	0.7984330	-0.7586142	-0.8582676
C	-0.2847325	-1.6306994	-0.8267481
C	-1.5696432	-1.1089995	-0.7105950
C	-1.7582289	0.2763576	-0.6273042
C	-0.6756793	1.1283429	-0.6598000
O	-2.6935832	-1.8684332	-0.6705280
C	-2.5432973	-3.2770999	-0.7523572
H	3.6792438	2.7701547	-0.8808941
H	1.8015790	-1.1601498	-0.9486575
H	-0.1167773	-2.6964354	-0.8924180
H	-2.7676035	0.6581463	-0.5374730

H	-0.8310808	2.1979723	-0.5948050
H	-3.5438410	-3.6978496	-0.7037866
H	-2.0722147	-3.5648925	-1.6948422
H	-1.9481237	-3.6548701	0.0819566

r11_an_b_1.log
E = -515.009513
H = -514.837762
G = -514.885778
NImag=0

C	0.6908743	0.5761454	-0.2775242
C	1.4950302	1.7940930	-0.3628245
H	1.0106955	2.6267013	-0.8640592
N	2.6741345	2.0898898	0.0377783
O	3.3263918	1.0515056	0.6834012
C	1.0618925	-0.6383105	0.3117155
C	0.1954604	-1.7245245	0.3236020
C	-1.0665387	-1.6163907	-0.2572706
C	-1.4560658	-0.4130546	-0.8503546
C	-0.5879979	0.6574934	-0.8558918
O	-1.9707431	-2.6253931	-0.2891949
C	-1.6073918	-3.8623296	0.3052525
H	4.1894130	1.4179213	0.9235156
H	2.0314698	-0.7519562	0.7689119
H	0.5140947	-2.6476159	0.7872267
H	-2.4392859	-0.3417740	-1.2981674
H	-0.8944315	1.5895341	-1.3172691
H	-2.4568290	-4.5272761	0.1755126
H	-0.7318196	-4.2895432	-0.1880364
H	-1.4020533	-3.7374153	1.3704766

r11_an_b_2.log
E = -515.009592
H = -514.837772
G = -514.884896
NImag=0

C	0.7139239	0.5127621	-0.1885894
C	1.3852099	1.8032771	-0.3365009
H	0.7750715	2.5874296	-0.7752613
N	2.5644070	2.2112108	-0.0519211
O	3.3783275	1.2405315	0.5113139
C	1.2598671	-0.6688714	0.3444620
C	0.5009337	-1.8200226	0.4215370
C	-0.8219493	-1.8349913	-0.0273034
C	-1.3848718	-0.6766560	-0.5593343
C	-0.6129012	0.4728600	-0.6314711
O	-1.4853065	-3.0108438	0.0896587
C	-2.8321352	-3.0591693	-0.3563789
H	4.2199202	1.6924570	0.6673116
H	2.2771800	-0.6928842	0.7000800
H	0.9194532	-2.7307548	0.8316366
H	-2.4059670	-0.6607770	-0.9138131
H	-1.0519472	1.3736135	-1.0456594
H	-3.1793008	-4.0730864	-0.1777586
H	-3.4537569	-2.3573935	0.2036925

H -2.8979581 -2.8342916 -1.4230008

r11_an_b_3.log
E = -515.003950
H = -514.832630
G = -514.879540
NImag=0

C	0.8805460	0.5964607	-0.4531871
C	1.7840345	1.7320243	-0.6915916
H	1.5449888	2.3866945	-1.5239334
N	2.8170389	2.1043219	-0.0357851
O	3.1541903	1.3721290	1.0886186
C	1.3214676	-0.6801953	-0.1029767
C	0.4256427	-1.7297378	0.0677659
C	-0.9373163	-1.5036948	-0.1098453
C	-1.3919562	-0.2343759	-0.4799825
C	-0.4913699	0.7944666	-0.6598942
O	-1.8866330	-2.4592146	0.0435163
C	-1.4628869	-3.7638300	0.4087738
H	2.4244450	0.7669591	1.3222227
H	2.3811195	-0.8788697	0.0090733
H	0.7999491	-2.7093215	0.3295454
H	-2.4535725	-0.0827745	-0.6293987
H	-0.8447134	1.7732871	-0.9629853
H	-2.3619177	-4.3688536	0.4873933
H	-0.8044612	-4.1871570	-0.3527659
H	-0.9469952	-3.7506182	1.3712363

r11_an_b_4.log
E = -515.003931
H = -514.832481
G = -514.878990
NImag=0

C	1.2390896	0.2162610	-0.4730104
C	2.3864691	1.0692908	-0.8150676
H	2.8933826	0.8543151	-1.7507484
N	2.9242055	2.0273778	-0.1600869
O	2.3942963	2.3266041	1.0825477
C	0.0676381	0.6869341	0.1385496
C	-0.9873418	-0.1687798	0.3901642
C	-0.8992036	-1.5175953	0.0392718
C	0.2491121	-1.9994988	-0.5881073
C	1.2961273	-1.1264559	-0.8469904
O	-1.9774769	-2.2858951	0.3297526
C	-1.9305813	-3.6581406	-0.0306842
H	1.7829258	1.6198932	1.3638168
H	-0.0393973	1.7362633	0.3865550
H	-1.8975976	0.1920914	0.8521319
H	0.3309206	-3.0357968	-0.8843230
H	2.1818659	-1.4955096	-1.3513866
H	-2.8754128	-4.0916062	0.2852890
H	-1.8199565	-3.7736067	-1.1110084
H	-1.1083653	-4.1669459	0.4770347

r11_b_1.log
E = -515.450085
H = -515.265535
G = -515.313639
NImag=0

C	0.4989761	0.6087351	-0.5326164
C	1.3405092	1.7276576	-0.8115335
H	0.9020942	2.5808536	-1.3148735
N	2.5920326	1.9029657	-0.5539109
O	3.3435742	0.9662208	0.0714340
C	0.8868311	-0.5809392	0.1091852
C	-0.0268560	-1.5935142	0.3205999
C	-1.3514839	-1.4373402	-0.1065409
C	-1.7535948	-0.2576014	-0.7476570
C	-0.8404248	0.7453301	-0.9548666
O	-2.2972975	-2.3720805	0.0610437
C	-1.9453862	-3.5916308	0.7065846
H	3.0717710	2.7647717	-0.8140641
H	4.2454911	1.3240932	0.1525069
H	1.9021852	-0.7201352	0.4454226
H	0.2898585	-2.5001556	0.8154087
H	-2.7824099	-0.1607797	-1.0683310
H	-1.1430532	1.6603913	-1.4496692
H	-2.8499247	-4.1918155	0.7341124
H	-1.1731397	-4.1168202	0.1425564
H	-1.5988525	-3.4027066	1.7237085

r11_b_2.log
E = -515.450327
H = -515.265720
G = -515.313140
NImag=0

C	0.5334826	0.5477606	-0.4538687
C	1.2653138	1.7085567	-0.8437305
H	0.8404601	2.3449661	-1.6117604
N	2.4000396	2.1511121	-0.4162046
O	3.0833708	1.5516046	0.5888885
C	0.9047235	-0.3782802	0.5462695
C	0.1026138	-1.4597470	0.8099755
C	-1.0895347	-1.6566264	0.0941638
C	-1.4759694	-0.7490680	-0.8969535
C	-0.6656124	0.3366790	-1.1562968
O	-1.8022498	-2.7411459	0.4255515
C	-3.0216539	-2.9918411	-0.2660760
H	2.7968709	3.0235801	-0.7662567
H	4.0265283	1.7548289	0.4559437
H	1.8134658	-0.2456062	1.1115064
H	0.3699236	-2.1788142	1.5731930
H	-2.3911055	-0.8858697	-1.4546128
H	-0.9552028	1.0472532	-1.9212957
H	-3.4266343	-3.9068795	0.1555028
H	-3.7262216	-2.1730170	-0.1127339
H	-2.8376085	-3.1282461	-1.3328051

r11_b_3.log

E = -515.450339
H = -515.265728
G = -515.313019
NImag=0

C	0.5992830	0.5713602	-0.4853469
C	1.2933067	1.7704523	-0.8210514
H	0.7089273	2.6158871	-1.1670312
N	2.5571573	2.0347472	-0.7815570
O	3.4881851	1.1026600	-0.4597645
C	1.1822534	-0.6391018	-0.0476259
C	0.3844467	-1.7128615	0.2553933
C	-1.0121902	-1.6223854	0.1337551
C	-1.6077701	-0.4347050	-0.3024629
C	-0.7997011	0.6408265	-0.6066434
O	-1.7024356	-2.7237908	0.4544786
C	-3.1227538	-2.6914257	0.3529292
H	2.9212921	2.9377119	-1.0868669
H	4.1594616	1.5454101	0.0900340
H	2.2522249	-0.7316909	0.0494545
H	0.8124835	-2.6482472	0.5912910
H	-2.6801151	-0.3508298	-0.4033173
H	-1.2496613	1.5662872	-0.9458460
H	-3.4707828	-3.6733446	0.6590117
H	-3.5380497	-1.9307002	1.0156104
H	-3.4303618	-2.4961597	-0.6754446

r11_b_4.log
E = -515.450178
H = -515.265501
G = -515.312862
NImag=0

C	0.5785680	0.6177268	-0.5844737
C	1.4232436	1.7249544	-0.8899494
H	0.9667861	2.6003500	-1.3371880
N	2.6948732	1.8715177	-0.7173730
O	3.4868427	0.8760143	-0.2489397
C	0.9854516	-0.6099419	-0.0301064
C	0.0624319	-1.6005606	0.2319492
C	-1.2919780	-1.3855612	-0.0566318
C	-1.7129823	-0.1699624	-0.6134973
C	-0.7897080	0.8110780	-0.8725890
O	-2.2479248	-2.2957878	0.1689512
C	-1.8789225	-3.5501781	0.7340480
H	3.1768731	2.7193184	-1.0167638
H	4.1408232	1.2853114	0.3458857
H	2.0242498	-0.7929509	0.1947351
H	0.3935364	-2.5370055	0.6568488
H	-2.7637783	-0.0294327	-0.8294724
H	-1.1065076	1.7534268	-1.3030755
H	-2.7977290	-4.1208014	0.8301852
H	-1.1852782	-4.0782537	0.0782156
H	-1.4279710	-3.4110615	1.7176412

r12_a_1.log
E = -456.347619

H = -456.180166
G = -456.223888
NImag=0

C	0.6922257	1.1470587	-0.4077687
N	0.5699921	1.8450455	-1.5117457
N	1.6798397	1.4023469	0.4299330
O	2.5991835	2.3578947	0.1004242
C	-0.2540845	0.0774928	-0.0617673
C	-0.8205134	-0.6965243	-1.0750844
C	-1.7094611	-1.7084889	-0.7467488
C	-2.0362590	-1.9433710	0.5848168
C	-1.4740174	-1.1680010	1.5932082
C	-0.5798734	-0.1570053	1.2746295
H	1.2626207	2.5454962	-1.7528543
H	-0.2279838	1.7056852	-2.1185690
H	1.9182296	0.7858070	1.2015837
H	2.3868154	3.1536222	0.6203780
H	-0.5515622	-0.5237118	-2.1100094
H	-2.1433910	-2.3162476	-1.5296174
H	-2.7331114	-2.7322385	0.8373946
H	-1.7373240	-1.3453960	2.6275004
H	-0.1602255	0.4640352	2.0563966

r12_a_2.log
E = -456.347609
H = -456.180301
G = -456.224665
NImag=0

C	0.5753007	1.2689943	-0.3060982
N	0.4816285	1.9319952	-1.4350403
N	1.4773455	1.6136185	0.5914893
O	2.1830291	2.7701149	0.4197715
C	-0.3037552	0.1289334	-0.0109303
C	-0.6955504	-0.7209487	-1.0458741
C	-1.5235849	-1.7981208	-0.7705554
C	-1.9646613	-2.0225976	0.5295470
C	-1.5776891	-1.1713349	1.5588265
C	-0.7443007	-0.0948329	1.2939333
H	1.1152021	2.6977767	-1.6360971
H	-0.2496150	1.7065369	-2.0975089
H	1.5874585	1.1329315	1.4798452
H	3.0672733	2.5265015	0.0930098
H	-0.3382742	-0.5529259	-2.0545610
H	-1.8212485	-2.4639940	-1.5696511
H	-2.6141522	-2.8623807	0.7407704
H	-1.9309400	-1.3405282	2.5673552
H	-0.4653661	0.5827609	2.0916683

r12_an_a_1.log
E = -455.895227
H = -455.741732
G = -455.785522
NImag=0

C	0.7282496	1.1098319	-0.0571045
N	1.7105414	1.1766669	0.8823904

N	0.6568361	1.8873125	-1.0837925
O	1.6904449	2.8418680	-1.0338299
C	-0.2947335	0.0478571	0.0704307
C	0.0087420	-1.1518880	0.7147525
C	-0.9512455	-2.1506913	0.8240662
C	-2.2217467	-1.9590468	0.2945768
C	-2.5303158	-0.7620811	-0.3449505
C	-1.5738729	0.2370116	-0.4551270
H	2.3068652	1.9927279	0.8767160
H	1.5414877	0.7429604	1.7791640
H	1.5632372	3.3744105	-1.8303541
H	1.0013630	-1.3162888	1.1164956
H	-0.7032159	-3.0804370	1.3202770
H	-2.9699904	-2.7367094	0.3823603
H	-3.5213017	-0.6036242	-0.7513894
H	-1.8155446	1.1749198	-0.9380815

r12_an_b_1.log
E = -455.886283
H = -455.732914
G = -455.777041
NImag=0

C	0.8304378	1.1798748	-0.3723511
N	0.2465728	2.2090280	-1.0751453
N	2.0622334	1.3041837	-0.0286884
O	2.4906906	0.2749869	0.8294022
C	-0.0631285	0.0449410	-0.0366573
C	0.3272435	-1.2710789	-0.2857170
C	-0.5421214	-2.3173800	-0.0106722
C	-1.8003353	-2.0587864	0.5252530
C	-2.1930610	-0.7488996	0.7735022
C	-1.3308187	0.3018716	0.4840769
H	0.8811699	2.9173385	-1.4255155
H	-0.4840255	1.9393892	-1.7218103
H	3.4040865	0.5145145	1.0366915
H	1.3050453	-1.4689965	-0.7054223
H	-0.2381665	-3.3362580	-0.2147756
H	-2.4737186	-2.8777723	0.7449823
H	-3.1715374	-0.5432657	1.1887660
H	-1.6342668	1.3249092	0.6700811

r12_an_b_2.log
E = -455.883089
H = -455.729945
G = -455.773816
NImag=0

C	0.9172012	1.2595331	-0.4353241
N	0.2804356	2.2647213	-1.1246523
N	2.1595171	1.4123843	-0.1420105
O	2.7223136	0.4530522	0.7116691
C	0.0667434	0.1059198	-0.0494429
C	-1.1560022	0.3268993	0.5844999
C	-1.9687177	-0.7478955	0.9194609
C	-1.5708310	-2.0447238	0.6116425
C	-0.3575577	-2.2680387	-0.0302882

C	0.4633592	-1.1967650	-0.3576320
H	0.8826362	2.9856758	-1.5056268
H	-0.4687008	1.9700337	-1.7384857
H	2.0165683	-0.0992952	1.0955821
H	-1.4611438	1.3397294	0.8171792
H	-2.9133934	-0.5732824	1.4184033
H	-2.2076923	-2.8814664	0.8694556
H	-0.0511325	-3.2758586	-0.2793222
H	1.4066971	-1.3631233	-0.8644078

r12_b_1.log
E = -456.340081
H = -456.173096
G = -456.217431
NImag=0

C	0.8114827	1.2104100	-0.3984929
N	0.4237061	2.0368448	-1.3478982
N	1.9659522	1.4269368	0.2037571
O	2.4884161	0.5407720	1.1021492
C	-0.0526536	0.0779470	-0.0334267
C	-0.2977528	-0.2273799	1.3058702
C	-1.1664184	-1.2608196	1.6192915
C	-1.7712702	-1.9975616	0.6049271
C	-1.5227208	-1.6929509	-0.7283583
C	-0.6710987	-0.6464165	-1.0519524
H	-0.5058193	1.9481839	-1.7388108
H	1.0181509	2.7936967	-1.6693183
H	2.5363335	2.2470934	0.0081423
H	3.0859821	-0.0470291	0.6072765
H	0.1697622	0.3530782	2.0893933
H	-1.3718159	-1.4925624	2.6560239
H	-2.4404364	-2.8105286	0.8563287
H	-1.9915939	-2.2676080	-1.5160906
H	-0.4692058	-0.4044063	-2.0882117

r12_b_2.log
E = -456.340946
H = -456.173568
G = -456.217328
NImag=0

C	0.7531745	1.3267898	-0.1325229
N	0.1877935	2.4718929	-0.4527834
N	2.0723078	1.2632098	-0.0633801
O	2.6878202	0.2356971	0.5987341
C	-0.0889035	0.1499873	0.1204916
C	0.2605538	-1.0990299	-0.3947084
C	-0.5796623	-2.1810507	-0.1861093
C	-1.7525655	-2.0232119	0.5466918
C	-2.0975912	-0.7786674	1.0609476
C	-1.2717226	0.3141995	0.8409490
H	0.7338365	3.3194900	-0.5675067
H	-0.8054300	2.5033573	-0.6462793
H	2.6499915	2.0885541	-0.2130583
H	3.1349167	-0.3087119	-0.0721437
H	1.1658109	-1.2160438	-0.9760076

H	-0.3203802	-3.1483102	-0.5957075
H	-2.4001443	-2.8742299	0.7148630
H	-3.0082061	-0.6580213	1.6325757
H	-1.5298997	1.2859992	1.2435542

r13_a_1.log
E = -264.783216
H = -264.672595
G = -264.709195
NImag=0

C	-1.3720138	-1.2579069	-0.0190621
C	-0.0352187	-0.6218089	-0.0500801
N	1.0480146	-1.3204115	-0.2885326
N	0.0404274	0.6735364	0.1685327
O	1.2390896	1.3109643	0.0148077
H	-1.3559778	-2.0974603	0.6742294
H	-2.1304942	-0.5411881	0.2849943
H	-1.6038673	-1.6354590	-1.0150333
H	1.9565197	-0.8723603	-0.3282421
H	0.9749215	-2.3176398	-0.4448601
H	-0.7858298	1.2578573	0.2598457
H	1.6005289	1.4684769	0.9047006

r13_an_a_1.log
E = -264.327566
H = -264.230619
G = -264.266614
NImag=0

C	-1.3318503	-1.2230600	0.0813039
C	-0.0027704	-0.6020213	-0.1736931
N	1.0980160	-1.2962330	0.2155387
N	0.0323013	0.5384285	-0.7724657
O	1.3774166	0.9575457	-0.9215609
H	-1.3888081	-2.1995604	-0.4022943
H	-1.4762502	-1.3717521	1.1528562
H	-2.1253814	-0.5861727	-0.3024101
H	0.9691632	-2.0671977	0.8546883
H	1.9902156	-0.8225924	0.2304765
H	1.3103477	1.8198153	-1.3521395

r13_an_b_1.log
E = -264.322006
H = -264.225042
G = -264.260943
NImag=0

C	-1.4383377	-1.3551507	0.1086878
C	-0.1597304	-0.5960654	0.0026548
N	0.9852791	-1.3204808	-0.2192153
N	-0.0751172	0.6842061	0.0601751
O	-1.3583191	1.2596138	0.1990925
H	-1.2520897	-2.4265032	0.0675832
H	-1.9435264	-1.1102607	1.0430987
H	-2.1075578	-1.0775198	-0.7065652
H	1.8603671	-0.8269345	-0.0893395

H	0.9794135	-2.2692328	0.1311827
H	-1.1830814	2.2089280	0.2445452

r13_an_b_2.log
E = -264.317526
H = -264.220535
G = -264.256063
NImag=0

C	-1.5492246	-0.9396133	0.1291411
C	-0.1483965	-0.4351771	0.0118831
N	0.8305764	-1.3760769	-0.2006033
N	0.1962224	0.8017650	0.0492700
O	-0.8599864	1.7198367	0.1773268
H	-1.5627812	-2.0267996	0.1049532
H	-2.0029627	-0.6075802	1.0654569
H	-2.1620138	-0.5711595	-0.6970335
H	0.6440960	-2.2962866	0.1760199
H	1.7855292	-1.0581942	-0.0841768
H	-1.7117588	1.2519856	0.2122627

r13_b_1.log
E = -264.778969
H = -264.668530
G = -264.704905
NImag=0

C	-1.3444229	-1.1586890	-0.0498567
C	-0.0252771	-0.4998041	0.0673343
N	1.0858912	-1.1919653	-0.0412778
N	0.0324931	0.7980077	0.2892872
O	-1.1192880	1.5347606	0.2837943
H	-1.2179863	-2.2278335	-0.1952256
H	-1.9258225	-0.9678442	0.8518704
H	-1.8852200	-0.7268572	-0.8920034
H	1.0368958	-2.1912554	-0.1927217
H	1.9985179	-0.7561969	0.0311226
H	0.9006216	1.3272659	0.3144464
H	-1.3860029	1.6579114	1.2113300

r1_1.log
E = -205.717984
H = -205.624450
G = -205.658799
NImag=0

N	-1.1722776	-0.6576462	0.0288386
C	-0.0029465	-0.0270586	0.0010157
N	1.1275487	-0.7174046	0.1082114
N	0.0357802	1.2945599	-0.1340271
H	-2.0361602	-0.1391397	-0.0497502
H	-1.2089077	-1.6619539	0.1340962
H	2.0195806	-0.2432728	0.0855792
H	1.1053102	-1.7226888	0.2089562
H	0.9214818	1.7805871	-0.1580137
H	-0.8205094	1.8254174	-0.2157063

r1_2.log
E = -205.717931
H = -205.624861
G = -205.659915
NImag=0

N	-1.1705059	-0.6621987	-0.0032510
C	-0.0028166	-0.0279777	0.0010240
N	1.1261757	-0.7146912	0.1405644
N	0.0352709	1.2933583	-0.1343262
H	-2.0321601	-0.1450486	-0.1090098
H	-1.2080735	-1.6668249	0.0991601
H	2.0174956	-0.2387400	0.1446946
H	1.1035327	-1.7197224	0.2439145
H	0.9193770	1.7826813	-0.1320378
H	-0.8195958	1.8214640	-0.2408327

r1_an_1.log
E = -205.239724
H = -205.159039
G = -205.191277
NImag=0

N	-1.1818116	-0.5651653	-0.1666972
C	-0.0328557	0.1701606	-0.0114585
N	1.1014939	-0.5866553	0.1536429
N	-0.0726116	1.4597480	-0.0227050
H	-2.0404124	-0.0502959	-0.0251796
H	-1.1815591	-1.5051255	0.2076338
H	1.9805431	-0.1087732	0.0062574
H	1.0744472	-1.5412787	-0.1815556
H	0.8729661	1.8346852	0.0400618

r1_an_2.log
E = -205.239496
H = -205.158928
G = -205.191407
NImag=0

N	-1.1861474	-0.5792040	-0.1285882
C	-0.0370459	0.1554711	0.0240125
N	1.0906400	-0.5957903	0.2451893
N	-0.0572536	1.4381347	-0.1183817
H	-2.0441679	-0.0463280	-0.0906834
H	-1.2231796	-1.4882983	0.3134452
H	0.9707492	-1.5295737	0.6155447
H	1.9017805	-0.0977172	0.5855787
H	0.8861248	1.8109057	-0.0203172

r2_1.log
E = -189.658261
H = -189.552948
G = -189.586987
NImag=0

C	-1.3494700	-0.7224118	0.0914688
C	-0.0681518	0.0147730	-0.0307823

N	1.0661602	-0.6350841	0.1033448
N	-0.0866833	1.3114872	-0.2432361
H	-1.2264249	-1.7549589	-0.2263649
H	-1.6466055	-0.7134973	1.1414224
H	-2.1259922	-0.2360811	-0.4939926
H	1.0562278	-1.6351544	0.2566016
H	1.9602470	-0.1597895	0.0610405
H	0.7674094	1.8543132	-0.2975680
H	-0.9718168	1.7892035	-0.3530341

r2_an_b_1.log
E = -189.183628
H = -189.091822
G = -189.125759
NImag=0

C	-1.3374485	-0.6037944	0.1988081
C	-0.1030953	0.1398313	-0.2116054
N	1.0566518	-0.5529161	-0.0427547
N	-0.0913340	1.3267365	-0.7079582
H	-1.0479198	1.6677839	-0.7854031
H	-1.4025602	-1.5518469	-0.3373873
H	-1.2978248	-0.8314058	1.2656568
H	-2.2271150	-0.0125859	-0.0074807
H	1.9342946	-0.0640580	-0.1522938
H	1.0544512	-1.3895446	0.5218184

r3_1.log
E = -381.223545
H = -381.061703
G = -381.103248
NImag=0

C	1.1561356	1.2412549	-0.3583212
N	0.7175199	2.4780355	-0.2498123
N	2.3882840	0.9986078	-0.7541949
C	0.2604061	0.1190920	-0.0339500
C	0.7858261	-1.0586073	0.4993390
C	-0.0616199	-2.1119873	0.8077366
C	-1.4283196	-1.9965277	0.5775910
C	-1.9517253	-0.8245971	0.0420620
C	-1.1115676	0.2362369	-0.2606750
H	1.2980513	3.2645837	-0.5178493
H	-0.1960759	2.6658464	0.1415357
H	3.0393019	1.7549345	-0.9321397
H	2.6945950	0.0499935	-0.9256247
H	1.8465079	-1.1451750	0.7011154
H	0.3444613	-3.0204653	1.2325095
H	-2.0870521	-2.8217816	0.8161389
H	-3.0138589	-0.7380153	-0.1451330
H	-1.5211698	1.1384715	-0.6982277

r3_an_a_1.log
E = -380.751046
H = -380.602717
G = -380.643969

NImag=0

C	1.3000426	1.2825602	-0.2227808
N	0.8024795	2.3731371	-0.8747585
N	2.5155375	1.1609491	0.1818293
H	3.0359172	2.0214744	-0.0033961
C	0.3432693	0.1588230	-0.0218821
C	-0.7515320	-0.0256448	-0.8667582
C	-1.6222527	-1.0901734	-0.6651752
C	-1.4133171	-1.9732596	0.3873412
C	-0.3259382	-1.7911805	1.2366783
C	0.5483957	-0.7337441	1.0303498
H	1.3831626	3.2001866	-0.9229749
H	-0.1963076	2.5207546	-0.9077632
H	-0.9180217	0.6462765	-1.7000792
H	-2.4622789	-1.2300366	-1.3337889
H	-2.0945970	-2.7998339	0.5460126
H	-0.1611299	-2.4726317	2.0618418
H	1.3947710	-0.5835571	1.6880040

r3_an_b_1.log
E = -380.752244
H = -380.603757
G = -380.644812
NImag=0

C	1.3427453	1.2076844	-0.4784267
N	0.7956277	2.4536303	-0.4155881
N	2.5610734	1.0474510	-0.8598270
H	2.7800396	0.0542879	-0.9197820
C	0.4277708	0.0869995	-0.1196975
C	0.9477439	-1.0737710	0.4535829
C	0.1110658	-2.1317441	0.7805901
C	-1.2547549	-2.0436134	0.5309544
C	-1.7798341	-0.8901614	-0.0400208
C	-0.9443291	0.1737222	-0.3575044
H	1.4170406	3.2412749	-0.5421220
H	-0.0281607	2.6048541	0.1484683
H	2.0092282	-1.1417349	0.6622667
H	0.5243364	-3.0232322	1.2351089
H	-1.9070826	-2.8702739	0.7829409
H	-2.8411902	-0.8179209	-0.2413022
H	-1.3599202	1.0648474	-0.8122417

r4_a_1.log
E = -651.324257
H = -651.047391
G = -651.104363
NImag=0

C	1.1368146	1.2998947	2.3405139
C	0.9641189	1.2686822	0.8666925
N	2.0574944	1.1710551	0.1236302
N	-0.2260839	1.3640862	0.2992205
C	-1.4869880	1.4128294	0.9638876
C	-1.8166247	0.4858565	1.9447642
C	-3.0639481	0.5545899	2.5523595
C	-3.9808143	1.5252397	2.1656583

C	-3.6498248	2.4314517	1.1636557
C	-2.4008206	2.3793743	0.5605854
C	2.1275128	1.0745226	-1.2987323
C	1.4020568	0.1007730	-1.9764695
C	1.5103551	0.0113862	-3.3582059
C	2.3506056	0.8747350	-4.0519037
C	3.0867246	1.8321418	-3.3618640
C	2.9748191	1.9383030	-1.9822700
H	0.3705116	1.9157761	2.8059477
H	1.0605733	0.2875418	2.7388781
H	2.1247580	1.6889425	2.5795312
H	2.9400668	1.2464804	0.6209235
H	-0.2579518	1.4676270	-0.7139468
H	-1.1129564	-0.2901116	2.2166093
H	-3.3225777	-0.1638716	3.3194119
H	-4.9539596	1.5699919	2.6373010
H	-4.3627226	3.1839973	0.8524695
H	-2.1269012	3.0814150	-0.2173909
H	0.7720947	-0.5869213	-1.4253849
H	0.9463781	-0.7444256	-3.8891795
H	2.4367883	0.7974196	-5.1280765
H	3.7472043	2.5014689	-3.8977405
H	3.5387969	2.6808484	-1.4312756

r4_a_2.log
E = -651.323579
H = -651.046751
G = -651.103945
NImag=0

C	1.0703895	0.9221410	2.3472430
C	0.9461111	1.0285556	0.8719310
N	2.0423972	0.8582582	0.1481011
N	-0.2126087	1.2808775	0.2877781
C	-1.4664367	1.4751393	0.9458617
C	-1.9614995	0.5272221	1.8321277
C	-3.1924565	0.7428984	2.4390950
C	-3.9295211	1.8839506	2.1437954
C	-3.4358675	2.8136850	1.2347044
C	-2.2006468	2.6128398	0.6338698
C	2.1264254	0.9006576	-1.2791231
C	1.7888572	2.0633290	-1.9621981
C	1.8859319	2.0902676	-3.3479852
C	2.3344453	0.9701018	-4.0380783
C	2.6863367	-0.1821179	-3.3427994
C	2.5788604	-0.2229200	-1.9591513
H	0.4053387	1.6293312	2.8391430
H	0.7991557	-0.0852186	2.6645389
H	2.1002037	1.1141670	2.6391154
H	2.8821957	0.6029247	0.6595462
H	-0.2162534	1.4083654	-0.7229750
H	-1.3976586	-0.3746374	2.0319953
H	-3.5800609	0.0089131	3.1336780
H	-4.8907369	2.0444752	2.6148773
H	-4.0096183	3.6996373	0.9954573
H	-1.7999558	3.3300955	-0.0719059
H	1.4673512	2.9389770	-1.4107359

H	1.6225025	2.9923150	-3.8846360
H	2.4150765	0.9962514	-5.1171078
H	3.0400201	-1.0538441	-3.8777880
H	2.8430215	-1.1144384	-1.4044745

r4_an_a_1.log
E = -650.859522
H = -650.596658
G = -650.653570
NImag=0

C	2.9504803	0.0240208	2.3213232
C	2.0373942	0.3821825	1.1937321
N	2.5218938	1.0215032	0.1835531
N	0.7086289	0.0619108	1.2911606
C	0.0268210	-0.5901660	2.3387755
C	0.5196839	-1.7398226	2.9546293
C	-0.2112167	-2.3518337	3.9657977
C	-1.4475774	-1.8479102	4.3529833
C	-1.9536263	-0.7201822	3.7146110
C	-1.2203062	-0.0917662	2.7184521
C	1.6975408	1.3191254	-0.9183269
C	1.2429544	0.3088524	-1.7728372
C	0.4887898	0.6211027	-2.8961874
C	0.1693285	1.9448402	-3.1843461
C	0.6159167	2.9530456	-2.3369063
C	1.3765499	2.6454031	-1.2148910
H	3.8347721	0.6531453	2.2594620
H	2.4671868	0.1615851	3.2886651
H	3.2658134	-1.0172471	2.2474010
H	0.1081334	0.4992642	0.5980905
H	1.4576179	-2.1692650	2.6301970
H	0.1844893	-3.2416523	4.4395096
H	-2.0152014	-2.3343333	5.1357997
H	-2.9197725	-0.3212475	3.9975543
H	-1.6045357	0.7937220	2.2249584
H	1.4967280	-0.7213108	-1.5482548
H	0.1507500	-0.1735448	-3.5502672
H	-0.4174152	2.1872247	-4.0613120
H	0.3754775	3.9874491	-2.5507698
H	1.7341009	3.4315047	-0.5601568

r4_an_a_2.log
E = -650.859955
H = -650.597490
G = -650.653048
NImag=0

C	4.1599617	0.8974176	2.2828235
C	2.8273160	0.6982802	1.6278088
N	2.5167667	1.4188915	0.6060623
N	2.0771627	-0.2726260	2.2455007
C	0.6894457	-0.5057477	2.1654240
C	-0.2171290	0.5520249	2.1439616
C	-1.5746338	0.2962535	2.0073496
C	-2.0416845	-1.0132271	1.9333123
C	-1.1382859	-2.0681460	2.0112101

C	0.2233337	-1.8174510	2.1220927
C	1.4006249	1.1745243	-0.2054670
C	1.1360550	-0.0920001	-0.7385047
C	0.0185304	-0.3021027	-1.5340904
C	-0.8461327	0.7491611	-1.8306974
C	-0.5609685	2.0207126	-1.3433089
C	0.5588679	2.2346541	-0.5499139
H	4.7851934	1.5498746	1.6801433
H	4.0276112	1.3332551	3.2748538
H	4.6607527	-0.0646433	2.4116918
H	2.5953479	-0.8940183	2.8559537
H	0.1490979	1.5688441	2.2164563
H	-2.2714723	1.1246875	1.9690893
H	-3.1012844	-1.2087669	1.8289817
H	-1.4916014	-3.0909719	1.9705969
H	0.9362211	-2.6334122	2.1527680
H	1.8016355	-0.9141832	-0.4994978
H	-0.1798142	-1.2933513	-1.9244107
H	-1.7200370	0.5812524	-2.4473526
H	-1.2138233	2.8518592	-1.5814099
H	0.7773421	3.2225546	-0.1608270

r4_an_b_1.log
E = -650.861291
H = -650.598184
G = -650.654976
NImag=0

C	0.6752295	-0.8202516	0.3217215
C	0.0239201	0.5292843	0.2124611
N	0.6353045	1.6583494	0.1686215
N	-1.3450983	0.4588700	0.1969373
C	-2.3031514	1.4763629	0.0712808
C	-2.0594424	2.7091332	-0.5365958
C	-3.0887668	3.6383611	-0.6488284
C	-4.3628320	3.3613748	-0.1701119
C	-4.6074643	2.1267136	0.4246184
C	-3.5891739	1.1950908	0.5455271
C	2.0324931	1.7167679	0.0795297
C	2.7353046	1.1001584	-0.9614699
C	4.1149587	1.2296382	-1.0578449
C	4.8207787	1.9776367	-0.1208983
C	4.1268157	2.6038351	0.9101427
C	2.7465731	2.4837410	1.0052889
H	0.0588222	-1.4890657	0.9229077
H	0.7861218	-1.2657466	-0.6681925
H	1.6615581	-0.7393165	0.7735640
H	-1.7270384	-0.4504530	0.4283405
H	-1.0793796	2.9353117	-0.9252803
H	-2.8848942	4.5901382	-1.1241285
H	-5.1551482	4.0931928	-0.2616225
H	-5.5948063	1.8876323	0.7998844
H	-3.7780156	0.2333042	1.0102487
H	2.1853018	0.5276984	-1.6999373
H	4.6400905	0.7452994	-1.8723843
H	5.8959846	2.0781633	-0.1978274
H	4.6624237	3.1946115	1.6434684

H	2.2051307	2.9809640	1.8016795

r4_an_b_2.log
E = -650.860058
H = -650.597199
G = -650.653403
NImag=0

C	0.1980376	1.7954439	1.2879954
C	0.0999855	1.5967553	-0.1930670
N	1.0886764	1.3178439	-0.9721387
N	-1.1430415	1.6176976	-0.7710519
C	-2.3968315	1.9079548	-0.2065081
C	-2.5883960	2.9275797	0.7265595
C	-3.8602929	3.1826977	1.2248003
C	-4.9571445	2.4521606	0.7825972
C	-4.7708526	1.4578179	-0.1726322
C	-3.5014841	1.1833944	-0.6598337
C	2.4089393	1.3238881	-0.5027644
C	2.9738924	2.4469819	0.1117910
C	4.3054866	2.4438713	0.5068487
C	5.1014572	1.3225388	0.2953718
C	4.5502867	0.2058554	-0.3257279
C	3.2213496	0.2086697	-0.7289567
H	0.2993295	2.8497286	1.5460406
H	-0.6918505	1.4098242	1.7840826
H	1.0763445	1.2726355	1.6627336
H	-1.1595749	1.2652506	-1.7226920
H	-1.7535147	3.5375546	1.0434365
H	-3.9931212	3.9748904	1.9511782
H	-5.9462541	2.6622078	1.1688059
H	-5.6158398	0.8862565	-0.5360374
H	-3.3503497	0.4030308	-1.3970908
H	2.3595496	3.3271741	0.2646464
H	4.7234694	3.3248999	0.9789440
H	6.1395819	1.3221225	0.6026782
H	5.1596972	-0.6722034	-0.5026199
H	2.7955646	-0.6567232	-1.2233891

r4_b_3.log
E = -651.323015
H = -651.046252
G = -651.103632
NImag=0

C	0.5169334	1.5893814	2.1565174
C	0.2180407	0.3434757	1.4108190
N	0.9617614	-0.0323276	0.3794926
N	-0.8363377	-0.4006008	1.7152172
C	-1.7552519	-0.1955160	2.7860615
C	-1.3092389	0.0176284	4.0848194
C	-2.2383994	0.1875714	5.1032780
C	-3.5998940	0.1216234	4.8299796
C	-4.0352910	-0.1182542	3.5309824
C	-3.1141955	-0.2751007	2.5045774
C	2.1412452	0.6029205	-0.1088171
C	3.1833256	0.9387689	0.7469802

C	4.3256219	1.5334016	0.2263536
C	4.4347138	1.7684733	-1.1394982
C	3.3965576	1.4045694	-1.9905655
C	2.2455344	0.8229050	-1.4774023
H	1.0047045	2.3019872	1.4947379
H	-0.4073360	2.0128630	2.5438878
H	1.1822807	1.3858831	2.9958807
H	0.6362030	-0.8279004	-0.1669336
H	-1.0583900	-1.1747631	1.0916211
H	-0.2481686	0.0333601	4.2984211
H	-1.8937419	0.3574351	6.1151024
H	-4.3196122	0.2478036	5.6284839
H	-5.0937003	-0.1787632	3.3131190
H	-3.4384914	-0.4578505	1.4873546
H	3.1080094	0.7219477	1.8048405
H	5.1372214	1.7993887	0.8911755
H	5.3298897	2.2257480	-1.5408521
H	3.4786966	1.5775487	-3.0557827
H	1.4270096	0.5384922	-2.1273515

r5_1.log
E = -556.720389
H = -556.490448
G = -556.539341
NImag=0

C	-0.5198492	1.2055870	0.0502408
N	-1.0101932	2.3973546	-0.1209370
C	-1.4347859	0.0921596	0.2905314
C	-2.6363116	0.3088293	0.9752017
C	-3.5163471	-0.7416829	1.1712458
C	-3.2116810	-2.0055371	0.6727253
C	-2.0216353	-2.2243032	-0.0133398
C	-1.1263522	-1.1824030	-0.1966208
C	0.9286175	1.0222857	-0.0037884
C	1.7064458	1.8353988	-0.8363819
C	3.0820366	1.6814734	-0.8594220
C	3.6885505	0.7307232	-0.0427921
C	2.9202467	-0.0755422	0.7905142
C	1.5409715	0.0588939	0.8048271
H	-0.4036947	3.2085002	-0.2069791
H	-2.0136167	2.5509237	-0.1734598
H	-2.8611525	1.2874385	1.3824718
H	-4.4365687	-0.5789107	1.7163131
H	-3.9040190	-2.8239398	0.8233372
H	-1.7916540	-3.2061277	-0.4051370
H	-0.2049839	-1.3430977	-0.7413942
H	1.2310160	2.5587848	-1.4881315
H	3.6820045	2.2970646	-1.5162984
H	4.7648211	0.6159815	-0.0593678
H	3.3957621	-0.8084078	1.4286120
H	0.9387728	-0.5561469	1.4610296

r5_an_1.log
E = -556.260090
H = -556.043360

G = -556.092725
NImag=0

C	-0.3442872	0.7533168	-0.8775652
N	-0.6862843	1.3512932	-1.9566626
C	-1.3392542	-0.1155516	-0.1955267
C	-1.3621244	-0.2291243	1.1955467
C	-2.3332281	-1.0008621	1.8215511
C	-3.2778332	-1.6821353	1.0622644
C	-3.2535974	-1.5836586	-0.3259910
C	-2.2934835	-0.8003175	-0.9502023
C	1.0124086	0.8899767	-0.2803968
C	1.6541304	-0.2071605	0.2970557
C	2.9479206	-0.0853784	0.7871277
C	3.6037209	1.1395620	0.7268937
C	2.9647325	2.2414606	0.1674185
C	1.6783834	2.1155377	-0.3389135
H	0.1006303	1.8873809	-2.3322187
H	-0.6278460	0.3009694	1.7903135
H	-2.3506274	-1.0711916	2.9017854
H	-4.0285476	-2.2914499	1.5498137
H	-3.9822319	-2.1199274	-0.9209052
H	-2.2672693	-0.7199735	-2.0296725
H	1.1434126	-1.1614956	0.3466430
H	3.4429046	-0.9462543	1.2184057
H	4.6082022	1.2366263	1.1192712
H	3.4670827	3.1997791	0.1297435
H	1.1777857	2.9768779	-0.7669790

r6_a_1.log
E = -482.967922
H = -482.706380
G = -482.758069
NImag=0

C	0.0091308	0.5981663	0.3897248
H	-0.4729111	1.5715455	0.3623901
N	1.2819258	0.5773747	0.2052100
C	-0.8355809	-0.5470947	0.6290375
C	-2.2002197	-0.2911046	0.8039668
C	-3.0790897	-1.3370979	1.0383864
C	-2.5951794	-2.6384107	1.0980323
C	-1.2353151	-2.9003277	0.9243558
C	-0.3542371	-1.8623091	0.6904514
C	2.1241677	1.7863667	-0.0401012
C	2.0069912	2.7200318	1.1556298
C	1.6456079	2.4712133	-1.3117931
C	3.5510953	1.2932159	-0.1993805
H	1.7757352	-0.3164544	0.2230010
H	-2.5563877	0.7309378	0.7535802
H	-4.1340715	-1.1401502	1.1737374
H	-3.2776624	-3.4586425	1.2808466
H	-0.8696021	-3.9172605	0.9727576
H	0.6987228	-2.0777447	0.5572009
H	2.3311146	2.2216033	2.0702439
H	2.6461227	3.5867551	0.9835038
H	0.9816706	3.0717971	1.2829633
H	1.7106983	1.7944770	-2.1649028

H	2.2823084	3.3361155	-1.5012131
H	0.6167550	2.8206534	-1.2089332
H	4.2030933	2.1471833	-0.3809853
H	3.8880414	0.7845878	0.7060649
H	3.6314756	0.6079725	-1.0455755

r6_an_a_1.log
E = -482.508308
H = -482.260906
G = -482.312372
NImag=0

C	0.6597761	-0.0622837	0.1991924
H	1.5117470	-0.6332349	-0.1797646
N	0.6745373	1.2053261	0.2951224
C	-0.4976487	-0.8892979	0.5854327
C	-1.6801733	-0.3428901	1.0914772
C	-2.7374721	-1.1692043	1.4386430
C	-2.6289025	-2.5507443	1.2855717
C	-1.4567431	-3.1021746	0.7831713
C	-0.3970712	-2.2725873	0.4353332
C	1.8579437	1.9729561	-0.1060334
C	3.0242688	1.1458662	-0.6283301
C	2.3092427	2.7632497	1.1187485
C	1.4068212	2.9482470	-1.1893720
H	-1.7627669	0.7301844	1.2096887
H	-3.6510774	-0.7394475	1.8302124
H	-3.4576316	-3.1922061	1.5581854
H	-1.3672993	-4.1744288	0.6623388
H	0.5218629	-2.6942460	0.0423140
H	2.7488161	0.5800780	-1.5217148
H	3.8405817	1.8200973	-0.8936058
H	3.3936549	0.4481248	0.1272071
H	1.4932472	3.3811466	1.4987793
H	3.1439315	3.4149546	0.8514923
H	2.6365698	2.0888729	1.9133366
H	2.2369023	3.6008654	-1.4684896
H	1.0753601	2.4089096	-2.0796222
H	0.5831231	3.5677668	-0.8291149

r6_an_a_2.log
E = -482.507580
H = -482.260324
G = -482.312686
NImag=0

C	0.5200732	0.0471669	0.0368349
H	1.1038364	-0.3593027	-0.7972570
N	0.7655340	1.1901715	0.5381823
C	-0.5541638	-0.8401516	0.5112463
C	-1.4040606	-0.4951682	1.5659239
C	-2.3997559	-1.3691196	1.9722403
C	-2.5602016	-2.5974838	1.3320717
C	-1.7195844	-2.9479802	0.2828908
C	-0.7210771	-2.0708613	-0.1241039
C	1.8774470	1.9716218	-0.0416135
C	1.9639209	3.2834563	0.7241870

C	1.6078965	2.2650022	-1.5138861
C	3.1924814	1.2136308	0.1081975
H	-1.2774969	0.4591513	2.0614150
H	-3.0553885	-1.0967496	2.7899107
H	-3.3398400	-3.2768102	1.6534573
H	-1.8406006	-3.9007209	-0.2169839
H	-0.0595620	-2.3350416	-0.9421829
H	2.1538114	3.0966369	1.7835700
H	2.7755229	3.8990878	0.3303766
H	1.0298346	3.8424000	0.6329926
H	1.5807304	1.3459572	-2.1029449
H	2.3998401	2.9027249	-1.9125643
H	0.6541254	2.7843576	-1.6325510
H	4.0160106	1.8303360	-0.2582505
H	3.3800559	0.9758275	1.1578432
H	3.1816106	0.2836608	-0.4640019

r6_an_b_1.log
E = -482.494202
H = -482.246802
G = -482.297218
NImag=0

C	0.2522537	1.1181388	-0.9731979
H	-0.0530769	1.9586274	-1.6010317
N	1.5054244	0.9117956	-0.9012842
C	-0.8823728	0.3980216	-0.3299386
C	-1.2113644	0.6324466	1.0032708
C	-2.2747203	-0.0451741	1.5899534
C	-3.0179396	-0.9549378	0.8465144
C	-2.7105547	-1.1681162	-0.4941896
C	-1.6549458	-0.4847401	-1.0833681
C	2.1432142	-0.1709207	-0.1336211
C	3.4853167	-0.4267177	-0.8146181
C	1.3812727	-1.4949544	-0.0835181
C	2.4036493	0.3372004	1.2802719
H	-0.6330470	1.3428326	1.5818260
H	-2.5210171	0.1374047	2.6285552
H	-3.8408687	-1.4879211	1.3061014
H	-3.2939672	-1.8671545	-1.0804139
H	-1.4097597	-0.6516648	-2.1261803
H	4.0682074	0.4950213	-0.8662381
H	4.0548085	-1.1708690	-0.2540548
H	3.3362567	-0.8001846	-1.8306763
H	0.5277028	-1.4706747	0.5941218
H	2.0656326	-2.2697920	0.2701151
H	1.0303416	-1.7794589	-1.0787045
H	2.9856801	-0.4058235	1.8305500
H	2.9706832	1.2704087	1.2571324
H	1.4667904	0.5078066	1.8132225

r6_b_1.log
E = -482.955823
H = -482.693858
G = -482.744360
NImag=0

C	0.1316102	1.1044631	-0.6985717
H	-0.1313281	2.0584337	-1.1471053
N	1.3000963	0.6801556	-1.0406534
C	-0.8755679	0.5377027	0.1774006
C	-1.1591415	-0.8278053	0.2807574
C	-2.1895326	-1.2541174	1.1020122
C	-2.9297572	-0.3291326	1.8333374
C	-2.6684252	1.0325017	1.7169682
C	-1.6645252	1.4671806	0.8680164
C	2.1996001	-0.4166539	-0.5739985
C	3.6027690	0.1648528	-0.7285696
C	2.0489162	-1.6192420	-1.4952790
C	1.9725313	-0.7699142	0.8873196
H	1.7660617	1.2760059	-1.7264369
H	-0.6056866	-1.5456573	-0.3078269
H	-2.4203497	-2.3090058	1.1689297
H	-3.7277473	-0.6710949	2.4801009
H	-3.2594678	1.7510986	2.2689261
H	-1.4702865	2.5252515	0.7383846
H	3.7475156	1.0147820	-0.0595266
H	4.3299466	-0.6067192	-0.4777355
H	3.7778239	0.4844694	-1.7585313
H	1.0434056	-2.0369388	-1.4572727
H	2.7566889	-2.3851248	-1.1749209
H	2.2792771	-1.3443532	-2.5258523
H	1.8692932	0.1313377	1.4946197
H	1.1107431	-1.4121030	1.0490595
H	2.8554367	-1.3122731	1.2280483

r7_1.log
E = -440.336919
H = -440.157291
G = -440.201053
NImag=0

C	1.2139454	0.5702499	-0.0010610
N	2.3339105	0.3748670	-0.6405002
C	0.3165203	-0.5262209	0.3687531
C	0.4156863	-1.7764777	-0.2462854
C	-0.4490389	-2.7929497	0.1250960
C	-1.4073460	-2.5680691	1.1085655
C	-1.5072567	-1.3230176	1.7205628
C	-0.6507220	-0.2988421	1.3504128
O	0.8332630	1.7574597	0.3502894
C	1.6720755	2.8962792	0.0900278
H	2.9316913	1.1424621	-0.9307597
H	2.6445952	-0.5646244	-0.8604260
H	1.1416886	-1.9611303	-1.0280930
H	-0.3795887	-3.7589477	-0.3567187
H	-2.0804420	-3.3655078	1.3963661
H	-2.2520683	-1.1504855	2.4859608
H	-0.7185764	0.6703445	1.8250132
H	1.1544163	3.7456947	0.5201019
H	2.6385943	2.7571945	0.5731983
H	1.7863522	3.0295211	-0.9855038

r7_2.log
E = -440.331263
H = -440.151930
G = -440.195992
NImag=0

C	1.2542810	0.5981292	-0.4139231
N	1.7431979	0.4003342	-1.6061108
C	0.4398501	-0.4385102	0.2292723
C	0.8344374	-1.7728867	0.1215814
C	0.0531400	-2.7640524	0.6955655
C	-1.1250063	-2.4250215	1.3518338
C	-1.5253614	-1.0950008	1.4421070
C	-0.7408317	-0.0948926	0.8907544
O	1.5237638	1.7462892	0.1218170
C	1.3521110	2.0238029	1.5273411
H	2.3504634	1.0950512	-2.0313018
H	1.5015124	-0.4340490	-2.1288076
H	1.7572480	-2.0260153	-0.3856170
H	0.3630685	-3.7984530	0.6302854
H	-1.7362969	-3.2009464	1.7945645
H	-2.4496247	-0.8379966	1.9420379
H	-1.0560474	0.9389956	0.9439616
H	0.4051874	2.5355769	1.6745275
H	1.3959263	1.1056159	2.1072077
H	2.1763810	2.6777295	1.7937033

r7_an_a_1.log
E = -439.879023
H = -439.712301
G = -439.757496
NImag=0

C	1.4063578	0.5782799	0.1376898
N	2.6658258	0.3841626	0.1913776
H	3.1976645	1.2251280	-0.0318888
C	0.4320222	-0.5048222	0.4248050
C	0.8990841	-1.7755040	0.7641339
C	0.0061664	-2.8008245	1.0359759
C	-1.3648504	-2.5675464	0.9716928
C	-1.8357128	-1.3044773	0.6343872
C	-0.9422378	-0.2754486	0.3615576
O	0.7966406	1.7388162	-0.1725023
C	1.6231009	2.8564950	-0.4690569
H	1.9658840	-1.9495030	0.8121498
H	0.3783785	-3.7830820	1.2982990
H	-2.0623880	-3.3680927	1.1839403
H	-2.9007854	-1.1172716	0.5828547
H	-1.3142173	0.7055358	0.0995739
H	0.9536349	3.6824654	-0.6901968
H	2.2490785	3.1130044	0.3880060
H	2.2517535	2.6529849	-1.3382986

r7_an_b_1.log
E = -439.880312
H = -439.713517
G = -439.757359

NImag=0

C	0.9535603	0.5278968	-0.1255846
N	2.1238807	0.5589956	-0.6314835
H	2.5137916	-0.3809890	-0.6835292
C	0.2033277	-0.6749035	0.3197037
C	0.5508688	-1.9361634	-0.1651359
C	-0.1353773	-3.0640820	0.2608726
C	-1.1735813	-2.9432895	1.1792584
C	-1.5261075	-1.6891426	1.6639654
C	-0.8453610	-0.5579493	1.2322841
O	0.2551179	1.6581667	0.0673368
C	0.8953699	2.8792468	-0.2861431
H	1.3494606	-2.0395593	-0.8904743
H	0.1368836	-4.0372181	-0.1272288
H	-1.7076369	-3.8240626	1.5129396
H	-2.3327729	-1.5898084	2.3790986
H	-1.1199805	0.4171349	1.6114127
H	0.1922552	3.6708052	-0.0422794
H	1.8168132	3.0066399	0.2820078
H	1.1250879	2.8978818	-1.3514208

r8_1.log
E = -248.711259
H = -248.590025
G = -248.627118
NImag=0

C	-1.2964275	-1.2468988	-0.0051744
C	0.0186231	-0.5804502	-0.0272025
C	1.2765881	-1.3267214	0.1715439
N	0.0545484	0.6913516	-0.2085228
O	1.2320426	1.3633633	-0.1387182
H	-1.2998323	-2.0193951	-0.7763034
H	-1.4057171	-1.7534787	0.9559990
H	-2.1184200	-0.5516809	-0.1600574
H	1.0792576	-2.3942020	0.1940133
H	1.9845896	-1.0817691	-0.6210034
H	1.7285824	-1.0160781	1.1165033
H	-0.7874375	1.2618293	-0.3105708
H	1.2388025	2.0118300	-0.8657067

r8_2.log
E = -248.709441
H = -248.588038
G = -248.624463
NImag=0

C	-1.1728990	-1.3419345	0.0016373
C	0.0093164	-0.4608248	-0.0630860
C	1.3789987	-0.9833220	0.1265737
N	-0.1770768	0.7888453	-0.2933585
O	0.7911387	1.7320941	-0.3772858
H	-1.0513488	-2.1355866	-0.7377316
H	-1.1904168	-1.8167386	0.9845685
H	-2.1012648	-0.8029574	-0.1723487
H	1.3466842	-2.0459673	0.3459783
H	1.9665926	-0.8206895	-0.7821036

H	1.8725837	-0.4555109	0.9470525
H	-1.1045440	1.1917025	-0.4308680
H	1.6593358	1.2892896	-0.3298282

r8_an_1.log
E = -248.268987
H = -248.160261
G = -248.196359
NImag=0

C	1.7266117	-0.7367307	0.0061699
C	0.4009668	-0.0574104	-0.0046058
C	0.3502504	1.4346649	-0.0178840
N	-0.6430041	-0.7949502	-0.0019220
O	-1.8142372	-0.0275042	-0.0129062
H	2.2962538	-0.4219264	0.8836465
H	1.6187362	-1.8200499	0.0152471
H	2.3026658	-0.4372757	-0.8724917
H	1.3552852	1.8518698	-0.0178817
H	-0.1845333	1.7900029	-0.9008577
H	-0.1909314	1.8054085	0.8547980
H	-2.5288999	-0.6792248	-0.0097821

r8_an_2.log
E = -248.264964
H = -248.156258
G = -248.191923
NImag=0

C	1.7126109	-0.7378723	0.0104752
C	0.3833888	-0.0637531	0.0115548
C	0.3448382	1.4312729	0.0430718
N	-0.6541882	-0.8108287	-0.0145288
O	-1.8845395	-0.1590199	-0.0126299
H	2.2742694	-0.4483003	0.9016692
H	1.6077483	-1.8210545	-0.0149783
H	2.2931025	-0.4086707	-0.8544715
H	-0.1833089	1.7931117	0.9299896
H	1.3552207	1.8332658	0.0616748
H	-0.1656815	1.8309579	-0.8378597
H	-1.7576930	0.8063891	0.0093947

r9_a_1.log
E = -209.422796
H = -209.330216
G = -209.363579
NImag=0

C	1.8485142	-0.1042751	0.0007090
C	0.4821407	0.4239939	-0.0279428
H	0.2753503	1.4874763	-0.0721338
N	-0.5352360	-0.3452431	0.0083896
O	-1.7885469	0.1666667	0.0779897
H	2.3808798	0.2688946	-0.8760180
H	2.3560555	0.3046743	0.8769610
H	1.8689556	-1.1911586	0.0260011
H	-0.4571518	-1.3650421	0.0732191

H	-2.3533284	-0.3711509	-0.5063729

r9_a_2.log
E = -209.422097
H = -209.329294
G = -209.362325
NImag=0

C	1.8410667	-0.0996968	0.0432210
C	0.4627138	0.3989864	0.1191428
H	0.2371420	1.3968626	0.4819549
N	-0.5281276	-0.3245521	-0.2317105
O	-1.8296769	0.0353532	-0.1910042
H	2.4225881	0.5785747	-0.5834643
H	2.2758388	-0.0621317	1.0439512
H	1.8881400	-1.1127777	-0.3496013
H	-0.4147063	-1.2759339	-0.5906640
H	-1.8946777	0.8992272	0.2593664

r9_an_a_1.log
E = -208.986327
H = -208.907110
G = -208.939908
NImag=0

C	1.8353387	-0.0842015	-0.0282339
C	0.4212292	0.3157126	0.1693593
H	0.1913161	1.2351283	0.7062058
N	-0.5282153	-0.4032543	-0.2813134
O	-1.7739629	0.1555260	0.0147353
H	2.3724574	0.6945044	-0.5734103
H	2.3311064	-0.1983951	0.9378263
H	1.9040247	-1.0206631	-0.5796523
H	-2.4180523	-0.4583273	-0.3654796

r9_an_a_2.log
E = -208.983379
H = -208.904200
G = -208.936796
NImag=0

C	-1.8261098	-0.0647008	0.0061250
C	-0.3971994	0.3255975	-0.0720540
H	-0.1299712	1.2376011	-0.6097826
N	0.5078108	-0.3840287	0.4749989
O	1.8065190	0.0868438	0.3315316
H	-2.4045703	0.7277335	0.4850050
H	-2.2331185	-0.1970292	-0.9982305
H	-1.9484457	-0.9889637	0.5682450
H	1.7939900	0.9142156	-0.1857863

r9_an_b_1.log
E = -208.986817
H = -208.907063
G = -208.939895
NImag=0

```
C       -0.8316175       -1.2691428       -0.0001102
C        0.4338930       -0.4883832       -0.0002420
H        1.3933370       -0.9923704       -0.0003101
N        0.5151351        0.7848057       -0.0002586
O       -0.7480563        1.3744998       -0.0001204
H       -0.6277746       -2.3366899       -0.0001962
H       -1.4315870       -1.0195181        0.8777327
H       -1.4318399       -1.0194156       -0.8777490
H       -0.5748897        2.3265144       -0.0001461
```

```
r9_an_b_2.log
E =      -208.982830
H =      -208.903319
G =      -208.935962
NImag=0
C       -1.0380370       -0.8269508       -0.0001051
C        0.3763673       -0.3596343       -0.0002551
H        1.1854435       -1.0805299       -0.0003147
N        0.7680627        0.8553699       -0.0003191
O       -0.2316081        1.8158542       -0.0002248
H       -1.0798477       -1.9124370       -0.0000484
H       -1.5704017       -0.4618994        0.8831456
H       -1.5705558       -0.4619848       -0.8832984
H       -1.1096232        1.3925120       -0.0000800
```

```
r9_b_1.log
E =      -209.423403
H =      -209.330487
G =      -209.364121
NImag=0
C        1.5693786       -0.4558396       -0.0314351
C        0.6219847        0.6656504        0.0088681
H        0.9107856        1.7028418       -0.0979879
N       -0.6308040        0.4820197        0.1737324
O       -1.1179700       -0.7703970        0.3051525
H        2.5842228       -0.0968132       -0.1595690
H        1.4852058       -1.0343455        0.8917533
H        1.3004253       -1.1259565       -0.8519203
H       -1.3104177        1.2456799        0.2002353
H       -2.0717759       -0.6967124        0.4875403
```

```
r9_b_2.log
E =      -209.421920
H =      -209.329043
G =      -209.363147
NImag=0
C       -1.0118049       -0.9129764       -0.0000048
C        0.3812981       -0.4411045       -0.0001294
H        1.2345436       -1.1069560       -0.0001152
N        0.6904878        0.7982104       -0.0003005
O       -0.1796000        1.8275637       -0.0003914
H       -1.0434803       -1.9967387        0.0004290
H       -1.5323554       -0.5317100        0.8834869
H       -1.5322012       -0.5324216       -0.8838955
```

H	1.6566213	1.1288045	-0.0004276
H	-1.0885088	1.4702286	-0.0000515

i1_1.log
E = -321.868408
H = -321.789086
G = -321.825978
NImag=0

C	-1.5053573	-0.5690736	0.0950466
C	-0.2378840	0.1722782	-0.1572609
C	1.0801400	-0.5166646	0.2325764
N	-0.2291331	1.3367780	-0.6759422
O	2.1383884	0.1215644	0.0131299
O	0.9878075	-1.6609375	0.7380663
H	0.7508620	1.6365497	-0.7492866
H	-1.4994639	-1.5203496	-0.4403139
H	-1.5987823	-0.8071468	1.1562558
H	-2.3619772	0.0228018	-0.2225712

i1_2.log
E = -321.864479
H = -321.785282
G = -321.823715
NImag=0

C	-1.4968355	-0.5735238	0.1569806
C	-0.2502223	0.1970406	-0.1343001
C	1.0836440	-0.5221053	0.1185880
N	-0.2260554	1.3940857	-0.5740349
O	2.1476424	0.0991596	-0.1133778
O	0.9941459	-1.7007358	0.5468489
H	-1.1829716	1.7394074	-0.6938101
H	-1.5270731	-1.4848217	-0.4427036
H	-1.5135433	-0.8846861	1.2028713
H	-2.3781311	0.0311794	-0.0563623

i1_h_1.log
E = -322.328686
H = -322.236823
G = -322.274490
NImag=0

C	-1.5310647	-0.6161478	0.2666192
C	-0.2682873	0.0556188	-0.0546184
C	1.0929778	-0.6303885	0.1950662
N	-0.2118871	1.2320385	-0.5532498
O	1.0286135	-1.7732887	0.6804231
O	2.0883318	0.0495998	-0.1223441
H	-1.5687650	-1.5598541	-0.2814007
H	-1.5224409	-0.8771782	1.3269069
H	-2.3952504	-0.0001336	0.0301723
H	0.7133688	1.6224582	-0.7455198
H	-1.0475965	1.7765757	-0.7586549

i2_1.log

E = -282.581760
H = -282.532005
G = -282.565798
NImag=0

H	-1.4619955	-0.4601917	0.0000000
C	-0.5623087	0.1575675	0.0000000
C	0.7449757	-0.6173800	0.0000000
N	-0.5925682	1.4291584	0.0000000
O	1.8236420	0.0197046	0.0000000
O	0.6112477	-1.8686169	0.0000000
H	-1.5612930	1.7589581	0.0000000

i2_2.log
E = -282.584922
H = -282.535011
G = -282.568380
NImag=0

H	-1.5980167	-0.4287277	0.0000000
C	-0.6792509	0.1559545	0.0000000
C	0.6186554	-0.6386663	0.0000000
N	-0.7293394	1.4265165	0.0000000
O	1.6910171	0.0115249	0.0000000
O	0.4869957	-1.8871303	0.0000000
H	0.2372388	1.7798284	0.0000000

i2_h_1.log
E = -283.037615
H = -282.974921
G = -283.009030
NImag=0

H	-1.5632352	-0.5735369	0.0000000
C	-0.6158928	-0.0486958	0.0000000
C	0.7167459	-0.7968847	0.0000000
N	-0.5963831	1.2231240	0.0000000
O	1.7283048	-0.0701284	0.0000000
O	0.6125133	-2.0370422	0.0000000
H	0.3188359	1.6847727	0.0000000
H	-1.4458887	1.7859914	0.0000000

i3_1.log
E = -509.887545
H = -509.803250
G = -509.847294
NImag=0

C	-1.4574705	-0.8646934	-0.1596664
C	-0.6716725	-0.1155560	0.9165982
C	0.5836832	0.4900282	0.3692036
C	1.5237890	-0.4756524	-0.3561261
N	0.9256651	1.7124574	0.4914226
O	2.2969847	-0.0125987	-1.2270209
O	1.4414506	-1.6782347	0.0035571
O	-2.2335232	-1.7801730	0.2178054
O	-1.2989081	-0.4887520	-1.3517873
H	-1.3147967	0.6719727	1.3191131

H	-0.4360482	-0.8055126	1.7280551
H	0.2105349	2.2240299	1.0172836

i3_2.log
E = -509.890916
H = -509.806655
G = -509.850323
NImag=0

C	-1.5205038	-0.7817898	-0.0614463
C	-0.5948394	-0.1715732	0.9928907
C	0.6129255	0.4529650	0.3776548
C	1.5671482	-0.4774943	-0.3872037
N	0.8541311	1.7006664	0.4745418
O	2.5211354	0.0664939	-0.9947114
O	1.3182520	-1.7055271	-0.3293635
O	-2.2685750	-1.7262948	0.3012029
O	-1.4998528	-0.2695700	-1.2123771
H	-1.1608419	0.5911629	1.5324084
H	-0.3064935	-0.9501832	1.7000888
H	1.7261640	1.8898704	-0.0349810

i3_5.log
E = -509.889035
H = -509.804937
G = -509.849092
NImag=0

C	-2.1696890	-0.1190417	-0.0203963
C	-0.8710640	-0.9041546	-0.1362483
C	0.3292595	-0.0298037	0.0656167
C	1.7073028	-0.7081824	-0.0274874
N	0.2320336	1.2174525	0.3037180
O	1.7137072	-1.9390107	-0.2647569
O	2.7072568	0.0299735	0.1422985
O	-2.6327802	0.3966937	-1.0729682
O	-2.6926168	-0.0262193	1.1225170
H	-0.8401767	-1.7053530	0.6056235
H	-0.7926426	-1.3721718	-1.1201385
H	1.1840033	1.5904799	0.4020053

i3_h_1.log
E = -510.354086
H = -510.257959
G = -510.302071
NImag=0

C	1.9601408	-0.2069971	-0.2422116
C	0.5394433	-0.7195991	-0.4940946
C	-0.6065863	0.1385076	-0.1408808
C	-2.0523107	-0.3433292	-0.3789658
N	-0.4626333	1.2997041	0.3691588
O	-2.9362540	0.4690013	-0.0413663
O	-2.1653034	-1.4782865	-0.8765680
O	2.1238713	0.9381456	0.2611133
O	2.8727202	-0.9970754	-0.5693947
H	0.4373887	-0.9807572	-1.5517787

H	0.4068392	-1.6721846	0.0276691
H	-1.3032432	1.8369008	0.5877925
H	0.5127668	1.6178166	0.5268309

i4_1.log
E = -549.164298
H = -549.050476
G = -549.097996
NImag=0

O	-2.7123525	1.2721385	-0.1636265
C	-1.6392679	1.9111761	-0.0008648
C	-0.3174920	1.1526895	-0.0032595
O	-1.5692034	3.1630923	0.1580968
C	-0.4591135	-0.3542469	-0.0000924
C	0.8369851	-1.0987915	-0.0045903
C	0.7629072	-2.6363139	0.0259138
N	1.9684992	-0.5127366	-0.0335288
O	1.8554882	-3.2530226	-0.0197659
O	-0.3798425	-3.1483747	0.0962287
H	2.6922557	-1.2420093	-0.0351222
H	0.2419970	1.4840021	-0.8829332
H	0.2621873	1.4899106	0.8590967
H	-1.0363592	-0.6856762	0.8683669
H	-1.0405885	-0.6901374	-0.8639193

i4_2.log
E = -549.165433
H = -549.051751
G = -549.098986
NImag=0

O	-1.8987812	0.9605126	-0.9931019
C	-1.2287741	1.6374718	-0.1673824
C	-0.8106405	0.9774650	1.1417987
O	-0.9156908	2.8509537	-0.3263782
C	-0.6218624	-0.5233861	1.0540286
C	0.5045916	-0.9361036	0.1596171
C	0.7462840	-2.4439305	-0.0270673
N	1.2527475	-0.0863721	-0.4247852
O	-0.0046834	-3.2202122	0.6108865
O	1.6774634	-2.7727917	-0.8018097
H	1.9474598	-0.6085015	-0.9722520
H	0.0975428	1.4658315	1.4977653
H	-1.5985772	1.2020713	1.8670408
H	-0.4357156	-0.9479041	2.0451989
H	-1.5345640	-1.0090042	0.6982408

i4_3.log
E = -549.163540
H = -549.049239
G = -549.096841
NImag=0

O	-1.5675051	0.6409675	-1.3771883
C	-1.2670282	1.1780177	-0.2755833
C	-1.3407921	0.3142937	0.9764458

O	-0.8622540	2.3650549	-0.1381324
C	0.0809039	-0.0692123	1.4186790
C	0.8314954	-0.7164270	0.2975776
C	0.3795853	-2.1070950	-0.1779315
N	1.8084370	-0.1262756	-0.2710787
O	0.8204438	-2.4915329	-1.2879967
O	-0.3705440	-2.7517302	0.5934798
H	2.1408726	-0.7297718	-1.0330506
H	-1.8226576	0.8731996	1.7798072
H	-1.9228843	-0.5832176	0.7680362
H	0.6246654	0.8256833	1.7254471
H	0.0196619	-0.7521544	2.2675890

i4_4.log
E = -549.162135
H = -549.048248
G = -549.095210
NImag=0

O	-2.3237092	2.4192069	0.2113415
C	-2.1274734	1.2173356	-0.1210381
C	-0.6884393	0.8221851	-0.4541080
O	-3.0284894	0.3441931	-0.2244153
C	-0.3802030	-0.6536167	-0.3022770
C	-0.4232771	-1.1639104	1.1097150
C	-0.1184633	-2.6586833	1.2930968
N	-0.6882891	-0.4704622	2.1462482
O	0.1852956	-3.0662761	2.4393497
O	-0.1979430	-3.3640741	0.2555254
H	-0.8816254	0.5053043	1.8989032
H	-0.5256025	1.1161156	-1.4947754
H	-0.0122752	1.4362016	0.1461890
H	-1.0752458	-1.2517687	-0.8953164
H	0.6121400	-0.8823507	-0.7031386

i4_5.log
E = -549.162268
H = -549.048338
G = -549.096751
NImag=0

O	-3.0059635	0.4186683	0.1218273
C	-2.0725276	1.2577088	0.0210976
C	-0.7437212	0.7929656	-0.5744436
O	-2.1591426	2.4737492	0.3484441
C	-0.4274815	-0.6714267	-0.3451902
C	-0.2494762	-1.0504953	1.0980869
C	-0.0447988	-2.5460200	1.3818597
N	-0.2243528	-0.2396129	2.0819778
O	-0.2830797	-2.9685920	2.5379407
O	0.3597097	-3.2352574	0.4108806
H	-0.3468531	0.7278814	1.7662948
H	-0.8079750	0.9806412	-1.6500201
H	0.0533346	1.4385540	-0.1980820
H	-1.2138256	-1.3057137	-0.7629423
H	0.4857533	-0.9516505	-0.8770314

i4_6.log
E = -549.161046
H = -549.046997
G = -549.094885
NImag=0

O	-2.5754721	2.7198009	-0.1706475
C	-1.8832561	1.6913535	-0.4119031
C	-1.5387517	0.8121323	0.7873291
O	-1.4646268	1.3600497	-1.5511483
C	-0.8495964	-0.4888596	0.4335910
C	-0.4703278	-1.3463116	1.6053712
C	0.2665504	-2.6496307	1.2709983
N	-0.7176611	-1.0945818	2.8312217
O	0.0344112	-3.1392233	0.1363048
O	1.0327111	-3.1305186	2.1392652
H	-1.2298356	-0.2123957	2.9225850
H	-0.9136358	1.4166708	1.4532903
H	-2.4698881	0.6270870	1.3304708
H	-1.4815346	-1.0864720	-0.2284137
H	0.0603134	-0.2983009	-0.1441148

i4_h_1.log
E = -549.628375
H = -549.502296
G = -549.550100
NImag=0

O	-1.5711733	2.0622350	1.6596763
C	-1.5037152	1.9118790	0.4001247
C	-0.2855284	1.1715352	-0.1447634
O	-2.3653371	2.3174476	-0.4121510
C	-0.4242082	-0.3501399	-0.1038906
C	-0.3497999	-0.9748011	1.2329429
C	-0.1190175	-2.4927786	1.3830071
N	-0.4794551	-0.3067397	2.3140788
O	0.0129495	-2.8915774	2.5574374
O	-0.1045648	-3.1399779	0.3199312
H	-0.1349573	1.4673730	-1.1813339
H	0.5955961	1.4657207	0.4300829
H	-1.3644207	-0.6879822	-0.5564861
H	0.3577455	-0.8192038	-0.7051098
H	-0.4050385	-0.8262518	3.1911543
H	-0.7459752	0.7094620	2.2800993

h2o.log
E = -76.397036
H = -76.372081
G = -76.394171
NImag=0

H	-0.6202412	1.1036132	1.4534752
H	-0.0334936	-0.2745750	1.7785792
O	0.2152758	0.6240588	1.5258556

k1_an_1.log

```
E =      -341.746212
H =      -341.680848
G =      -341.718495
NImag=0
C       -1.5107754       -0.3475730       -0.1044147
C       -0.2502597        0.3161658        0.3332579
C        1.0938360       -0.3452970       -0.0919070
O       -0.2336343        1.3352733        0.9972057
O        2.1391450        0.2265679        0.2850093
O        0.9947304       -1.3909782       -0.7733465
H       -1.5309175       -1.3718616        0.2725915
H       -2.3761615        0.2060713        0.2524795
H       -1.5246629       -0.4168684       -1.1937756


k1_ha_1.log
E =      -398.282686
H =      -398.175113
G =      -398.215125
NImag=0
C       -1.1129335       -0.8581039       -0.0136582
C        0.2986306       -0.2957605       -0.0680230
C        0.2622631        1.2355049        0.1351308
O        1.0539148       -0.8706246        0.9786047
O        0.7982554        1.6688325        1.1872105
O       -0.2959859        1.9166203       -0.7562736
N        0.9558178       -0.5510005       -1.3415476
H       -1.0644671       -1.9441888       -0.1228416
H       -1.7146629       -0.4484157       -0.8254780
H       -1.5898769       -0.6206143        0.9391743
H        1.3486593       -0.1042196        1.5115853
H        0.7547623       -1.5071683       -1.6319234
H        0.5376231        0.0628386       -2.0383601


k2_an_1.log
E =      -302.458191
H =      -302.422097
G =      -302.455830
NImag=0
C        0.6301220       -0.2986123        0.0001210
C       -0.8552053        0.1082616       -0.0004106
O        0.9996734       -1.4493064       -0.0005317
O       -1.7020779       -0.8091335        0.0000525
O       -1.0428050        1.3470318       -0.0001250
H        1.3446785        0.5405034        0.0008822


k2_ha_1.log
E =      -359.002943
H =      -358.923954
G =      -358.961289
NImag=0
H       -1.0586254       -0.8182998        0.1065272
C       -0.0175683       -0.4811550        0.0400789
C       -0.0227040        1.0561581        0.0728625
O        0.6681318       -0.9749496        1.1688316
```

O	0.5358729	1.5932660	1.0625618
O	-0.5772567	1.6402374	-0.8871891
N	0.6183438	-0.9164565	-1.1877384
H	1.0060271	-0.1718731	1.6143430
H	0.3128846	-1.8552069	-1.4293366
H	0.3222942	-0.2992205	-1.9407409

k2_ha_2.log
E = -359.003186
H = -358.923990
G = -358.961274
NImag=0

H	-0.9942535	-0.8631094	0.0066421
C	0.0450297	-0.5314675	0.0740356
C	0.0474455	1.0088212	0.0786791
O	0.6170479	-0.9921074	1.2757902
O	0.6239647	1.5704200	1.0472351
O	-0.5108696	1.5757942	-0.8900411
N	0.7560466	-1.0862789	-1.0632018
H	0.9189334	-0.1756694	1.7236219
H	0.2052754	-0.9328930	-1.9046763
H	1.6370801	-0.5900098	-1.1901848

k2_hydrate_1.log
E = -378.873065
H = -378.806409
G = -378.844454
NImag=0

C	-0.6354776	-0.0785435	0.3335037
C	0.8756104	0.0407200	0.0402553
O	1.3357952	1.1609218	-0.2757620
O	1.5248858	-1.0245178	0.1815170
H	-0.7548944	-0.2963938	1.4000957
O	-1.1393225	-1.1521406	-0.4255678
H	-1.9770140	-1.4435973	-0.0391463
O	-1.2883030	1.1228762	0.0173776
H	-2.1687949	1.1114892	0.4187288

k2_hydrate_2.log
E = -378.878051
H = -378.811425
G = -378.848290
NImag=0

C	0.6556591	-0.1520020	0.3735959
C	-0.8434357	0.0110750	0.0478434
O	-1.5775698	-0.9775998	0.2660707
O	-1.1843219	1.1258996	-0.4197673
H	0.7812078	-0.5216746	1.3939762
O	1.3043010	1.0840089	0.2567680
H	0.6635245	1.6524261	-0.2176431
O	1.1643820	-1.0953745	-0.5417200
H	2.0243870	-1.4054726	-0.2253399

k2_hydrate_3.log
E = -378.877507
H = -378.811052
G = -378.848486
NImag=0

C	-0.6856895	0.0000476	0.3834103
C	0.8218437	-0.0002154	0.0656981
O	1.3603107	1.1251422	-0.0526311
O	1.3599771	-1.1257462	-0.0524914
H	-0.8236672	-0.0002282	1.4712950
O	-1.2882031	-1.1488363	-0.1555034
H	-0.5519158	-1.7302378	-0.4209531
O	-1.2877655	1.1494465	-0.1548589
H	-0.5512185	1.7304075	-0.4206386

k4_an_1.log
E = -569.042948
H = -568.942851
G = -568.990564
NImag=0

O	0.5979602	2.4370636	-0.9317548
C	-0.3701644	2.5929823	-0.1422058
C	-1.4647553	1.5329040	-0.0993044
O	-0.5072585	3.5735404	0.6419555
C	-1.3536813	0.4771104	-1.1767277
C	-2.4230122	-0.5646841	-1.1106616
C	-2.3877883	-1.6938114	-2.1833990
O	-3.3004614	-0.5662633	-0.2677660
O	-3.2079271	-2.6249219	-2.0323712
O	-1.5412745	-1.5590714	-3.0959765
H	-1.4297716	1.0724002	0.8921368
H	-2.4282971	2.0440431	-0.1570964
H	-1.3858780	0.9162504	-2.1783634
H	-0.3924904	-0.0447424	-1.1356656

k4_an_2.log
E = -569.041406
H = -568.941122
G = -568.989704
NImag=0

O	-0.7599736	3.2819321	-0.7715579
C	-0.1235992	2.2718717	-0.3768521
C	-0.7805549	0.8974723	-0.4773436
O	1.0453578	2.2906979	0.0985540
C	-2.2453016	0.9325747	-0.9039058
C	-2.7823417	-0.4583338	-1.0265005
C	-2.1303456	-1.3869735	-2.0809249
O	-3.6963977	-0.8829106	-0.3415468
O	-2.0614822	-2.5997628	-1.7855229
O	-1.7554509	-0.8291289	-3.1380499
H	-0.1889149	0.3128089	-1.1882241
H	-0.6724369	0.4063248	0.4923175
H	-2.8513604	1.4814759	-0.1831936
H	-2.3242982	1.4205513	-1.8778494

k4_ha_1.log
E = -625.580149
H = -625.438094
G = -625.487528
NImag=0

C	0.2511460	0.3748949	-0.8453804
C	1.6693074	0.5472180	-0.2905881
C	1.9832038	-0.6512225	0.6374253
O	2.5765483	0.4830991	-1.3915442
O	1.9122896	-0.4876817	1.8770908
O	2.2577170	-1.7239823	0.0380458
N	1.8639098	1.7920579	0.4006201
C	-0.8347051	0.2339390	0.2154634
C	-1.0767205	1.4993308	1.0319034
O	-1.2854918	2.5683923	0.3929328
O	-1.0853555	1.4046306	2.2892048
H	0.2541264	-0.5119843	-1.4841644
H	0.0456449	1.2416983	-1.4818174
H	2.7044492	-0.4675220	-1.5588732
H	1.1431329	2.4639770	0.1487461
H	1.8035163	1.6207641	1.4021461
H	-0.6132419	-0.5971521	0.8882689
H	-1.7794769	0.0038430	-0.2848799

k4_ha_10.log
E = -625.577223
H = -625.435187
G = -625.485156
NImag=0

C	0.2916725	0.2004092	-0.8214170
C	1.7080878	0.3360893	-0.2558854
C	1.9499839	-0.8202789	0.7500221
O	2.6328574	0.1299051	-1.3222928
O	1.9850534	-0.5339797	1.9719082
O	2.0481774	-1.9710736	0.2584931
N	1.9379817	1.6117520	0.3762205
C	-0.8044482	0.1779572	0.2382515
C	-1.0027135	1.5046405	0.9637254
O	-1.1689169	2.5336734	0.2504743
O	-1.0190368	1.4975721	2.2241767
H	0.2547870	-0.7222565	-1.4041293
H	0.1320303	1.0380573	-1.5101900
H	2.4407267	0.7840882	-2.0103252
H	1.2366701	2.2910053	0.0908688
H	1.8849856	1.4938565	1.3856972
H	-0.6152926	-0.6120700	0.9687229
H	-1.7550057	-0.0525474	-0.2505211

k4_ha_12.log
E = -625.578118
H = -625.435903
G = -625.488250
NImag=0

C	0.5987471	1.0317993	-0.3884610

C	2.0556492	0.5811967	-0.2654531
C	2.0899814	-0.8662926	0.2886794
O	2.6273752	0.5473679	-1.5628960
O	2.1670645	-1.0255504	1.5285360
O	2.0123877	-1.7821330	-0.5755660
N	2.8152007	1.5302251	0.5162322
C	-0.2006532	1.0488917	0.9021241
C	-1.6343360	1.5506102	0.7531629
O	-2.2920847	1.6865611	1.8235064
O	-2.0844257	1.7873118	-0.3982128
H	0.1204087	0.3635251	-1.1094758
H	0.6170829	2.0301409	-0.8353005
H	2.5137997	-0.3769661	-1.8547139
H	2.3430581	1.6868013	1.4035052
H	3.7215966	1.1251809	0.7446783
H	0.2747431	1.6727441	1.6646637
H	-0.2650951	0.0447860	1.3315909

k4_ha_13.log
E = -625.578163
H = -625.435898
G = -625.487714
NImag=0

C	0.4790010	0.5052662	0.4681256
C	1.8982308	0.3836203	-0.0808661
C	2.4388434	-1.0315091	0.2471703
O	1.9039102	0.5289542	-1.4849912
O	2.7305703	-1.7643426	-0.7363205
O	2.5535978	-1.3347981	1.4577127
N	2.7447539	1.4187340	0.4971904
C	-0.2139776	1.7943000	0.0667242
C	-1.6375054	1.9408172	0.5908447
O	-2.2257144	3.0283660	0.3265201
O	-2.1498686	0.9949795	1.2458980
H	0.5274429	0.4241539	1.5569946
H	-0.0948685	-0.3536886	0.1086222
H	2.2481251	-0.3298191	-1.8078720
H	2.6868226	1.3539020	1.5119577
H	3.7145360	1.2360833	0.2434220
H	-0.2645293	1.8799290	-1.0223132
H	0.3483298	2.6643517	0.4127805

k4_ha_14.log
E = -625.579820
H = -625.437341
G = -625.485603
NImag=0

C	1.0988740	1.2096278	1.1694797
C	2.0113565	0.7164551	0.0392254
C	1.8033754	-0.8155769	-0.0879091
O	1.7277528	1.3737350	-1.1741561
O	2.3230473	-1.5170435	0.8214145
O	1.0964009	-1.2478298	-1.0311241
N	3.3874546	1.0568032	0.3711727
C	-0.2920839	0.5916373	1.2368276

C	-1.2369812	0.8288114	0.0655838
O	-2.3250082	0.2005992	0.0858440
O	-0.9064561	1.6485677	-0.8427419
H	1.0382696	2.2977591	1.0754234
H	1.6089576	0.9909933	2.1114047
H	0.7514403	1.5336835	-1.1880888
H	3.6667172	0.4777843	1.1612481
H	3.9791138	0.7765323	-0.4107046
H	-0.8091521	0.9774197	2.1199744
H	-0.2284786	-0.4901587	1.3872263

k4_ha_2.log
E = -625.579011
H = -625.437192
G = -625.488582
NImag=0

C	0.5514400	0.9938309	-0.4011421
C	2.0023940	0.5640138	-0.1676583
C	2.0075825	-0.8736545	0.4052584
O	2.6580401	0.5208876	-1.4350851
O	1.7931136	-1.7796824	-0.4419543
O	2.1868924	-1.0319571	1.6351825
N	2.7178159	1.4632177	0.6953684
C	-0.3136464	0.9246829	0.8433009
C	-1.7282707	1.4695196	0.6791697
O	-2.1023398	1.8735432	-0.4531215
O	-2.4497783	1.4718152	1.7165319
H	0.1319114	0.3625251	-1.1888530
H	0.5839491	2.0188325	-0.7852357
H	2.4167530	-0.3416854	-1.8165347
H	2.2412179	2.3587500	0.7637354
H	2.7656359	1.0542873	1.6253894
H	0.1531717	1.4705711	1.6692922
H	-0.4137824	-0.1102973	1.1863561

k4_ha_3.log
E = -625.578623
H = -625.436250
G = -625.486597
NImag=0

C	0.1451446	-0.2750887	0.2079326
C	1.5317454	0.2730030	-0.1389637
C	2.6028832	-0.6925916	0.4104228
O	1.6637555	0.3458429	-1.5420750
O	3.2933196	-1.3038471	-0.4469233
O	2.6997718	-0.7898074	1.6554227
N	1.8072385	1.5941922	0.4137332
C	-0.9819481	0.6681187	-0.1941433
C	-1.2226257	1.8208202	0.7786293
O	-1.4278851	2.9670274	0.2890258
O	-1.2405353	1.5576410	2.0115422
H	0.1018594	-0.4651128	1.2827684
H	0.0363336	-1.2370438	-0.3006572
H	2.4055251	-0.2609627	-1.7427535
H	1.1243922	2.2537509	0.0399064

H	1.6641745	1.5580759	1.4223864
H	-1.9209371	0.1082232	-0.2314220
H	-0.8104120	1.0731586	-1.1937317

k4_ha_4.log
E = -625.578457
H = -625.436426
G = -625.487381
NImag=0

C	0.4904211	0.9265999	-0.4187916
C	1.9619145	0.5370915	-0.2446861
C	2.0324816	-0.8623685	0.4107427
O	2.5405791	0.4331907	-1.5436512
O	2.2278478	-0.9348295	1.6457922
O	1.8513873	-1.8267655	-0.3775605
N	2.7032367	1.4968118	0.5269950
C	-0.3115321	0.9066772	0.8741353
C	-1.7035422	1.5080714	0.7164736
O	-1.7933114	2.6416861	0.1695672
O	-2.6837475	0.8487562	1.1590867
H	0.0429484	0.2465575	-1.1496303
H	0.4829263	1.9322346	-0.8508652
H	2.3310543	-0.4715953	-1.8381706
H	2.2206362	2.3917642	0.5540723
H	2.7874985	1.1515581	1.4800812
H	0.2116100	1.4967113	1.6356049
H	-0.4062086	-0.1117518	1.2546043

k4_ha_5.log
E = -625.578362
H = -625.436119
G = -625.487968
NImag=0

C	0.3971361	0.4453385	0.4325973
C	1.8572242	0.3394252	-0.0150028
C	2.3813568	-1.0723645	0.3310389
O	1.9263115	0.5121445	-1.4146771
O	2.4617720	-1.3715201	1.5449437
O	2.6855975	-1.8060242	-0.6450937
N	2.7482991	1.2909913	0.6351349
C	-0.2583520	1.7585731	0.0457420
C	-1.7086819	1.9031554	0.4951540
O	-2.2611052	3.0160108	0.2625763
O	-2.2736167	0.9302862	1.0600242
H	0.3670681	0.3137947	1.5170822
H	-0.1577514	-0.3877161	-0.0098202
H	2.3211534	-0.3238352	-1.7375029
H	2.3468964	2.2258443	0.5887495
H	2.8104425	1.0398611	1.6202216
H	-0.2468363	1.8898950	-1.0397039
H	0.2836859	2.6133402	0.4609361

k4_ha_6.log
E = -625.580597

H = -625.438388
G = -625.488837
NImag=0

C	0.8884276	1.4144677	0.3870003
C	2.0794021	0.6431168	-0.1806815
C	2.1144931	-0.7774379	0.4253095
O	1.9297741	0.5314506	-1.5809337
O	1.9034250	-1.7280930	-0.3708318
O	2.3371351	-0.8653993	1.6553076
N	3.3480248	1.2863698	0.1237289
C	-0.4340995	0.6934728	0.2055228
C	-1.6667083	1.4628028	0.6662723
O	-1.5213434	2.6025012	1.1816706
O	-2.7798884	0.8889133	0.4976603
H	0.8684263	2.3872332	-0.1151753
H	1.0766091	1.5976917	1.4478268
H	1.8777942	-0.4321739	-1.7437244
H	3.2455936	2.2939390	0.0075219
H	3.5518730	1.1226441	1.1081193
H	-0.4313388	-0.2550084	0.7543725
H	-0.5876998	0.4321095	-0.8453661

k4_ha_8.log
E = -625.581108
H = -625.438711
G = -625.488965
NImag=0

C	0.9322628	1.4339875	0.3239550
C	2.0790243	0.6470534	-0.2994770
C	2.1276455	-0.7734575	0.3168151
O	1.8798115	0.5092432	-1.6898939
O	1.9264217	-1.7372040	-0.4696434
O	2.3582947	-0.8536674	1.5465106
N	3.3217923	1.3723631	-0.0841990
C	-0.3897418	0.6941492	0.2473852
C	-1.6031390	1.4745650	0.7388268
O	-2.7187835	0.8875250	0.6516314
O	-1.4401681	2.6346712	1.2000981
H	0.8686223	2.3870718	-0.2079338
H	1.1853357	1.6535224	1.3643674
H	1.7952689	-0.4568192	-1.8258009
H	3.4400103	1.5215316	0.9162427
H	4.1036937	0.7957418	-0.3922917
H	-0.3502776	-0.2290048	0.8359716
H	-0.5956738	0.3860278	-0.7820641

k4_ha_9.log
E = -625.575438
H = -625.433207
G = -625.483704
NImag=0

C	0.1889899	-0.3665437	0.2331905
C	1.6062010	0.1442580	-0.0463088
C	2.5754713	-0.8806220	0.5968280
O	1.8752869	0.1371699	-1.4434039

O	2.8229506	-1.9261251	-0.0555126
O	2.9999992	-0.6176613	1.7497822
N	1.7571463	1.4944871	0.4436035
C	-0.9126695	0.5456532	-0.2850606
C	-1.2291061	1.7656871	0.5752676
O	-1.7002356	2.7754163	-0.0181714
O	-1.0542132	1.6865996	1.8212766
H	0.0751909	-0.5068972	1.3118009
H	0.1134804	-1.3493618	-0.2370147
H	1.4116861	0.9003101	-1.8198011
H	1.2339905	1.6090535	1.3089246
H	2.7357866	1.6840725	0.6469177
H	-1.8469331	-0.0222757	-0.3393053
H	-0.7055225	0.8819795	-1.3041131

nh3.log
E = -56.516396
H = -56.478554
G = -56.501447
NImag=0

N	0.2319351	0.6309166	1.5513305
H	-0.6363073	1.1443341	1.4274128
H	-0.0193563	-0.3255458	1.7851752
H	0.6907839	0.6035312	0.6445725

References

(S1) For a web-tool, see e.g.: https://nicoco007.github.io/Propagation-of-Uncertainty-Calculator/

(S2) Z. Li, H. Jangra, Q. Chen, P. Mayer, A. R. Ofial, H. Zipse, H. Mayr, *J. Am. Chem. Soc.* **2018**, *140*, 5500-5515

(S3) P. Geneste, R. Durand, I. Hugon, C. Reminiac, *J. Org. Chem.* **1979**, *44*, 1971–1973

(S4) J. P. Guthrie, *J. Am. Chem. Soc.* **2000**, *122*, 5529-5538.

(S5) T. P. Silverstein, S. T. Heller, *J. Chem. Edu.* **2017**, *94*, 690-695.

(S6) R. Huisgen, *Angew. Chem., Int. Ed.*, **1970**, *9*, 751–762

(S7) Origin(Pro), Version 2018b. OriginLab Corporation, Northampton, MA, USA.

(S8) a) P. Thordarson, *Chem. Soc. Rev.* **2011**, *40*, 1305-1323. b) P. Thordarson, In Supramolecular Chemistry: From Molecules to Nanomaterials (Eds.: J. W. Steed, P. A. Gale), Wiley: Chichester, UK, 2012, Vol. 2, pp 239-274.

(S9) P. Zuman, *Collect. Czech. Chem. Commun.* **1950**, *15*, 839–873.

(S10) A. J. Hoefnagel, H. van Bekkum, J. A. Peters, *J. Org. Chem.* **1992**, *57*, 3916-3921.

(S11) S. Hoops, S. Sahle, R. Gauges, C. Lee, J. Pahle, N. Simus, M. Singhal, L. Xu, P. Mendes, U. Kummer, *Bioinformatics* **2006**, *22*, 3067-3074.

(S12) M. R. Wright, An Introduction to Chemical Kinetics, John Wiley & Sons, Ltd, 2004, Chichester, pp. 79-80.

(S13) S. L. Miller, D. Magowan-Smith, *J. Phys. Chem. Ref. Data*, **1990**, *19*, 1049–1073.

(S14) P. C. Engel, K. Dalziel, *Biochem. J.* **1967**, *105*, 691-695.

(S15) C. E. Grimshaw, W. W. Cleland, *Biochemistry* **1981**, *20*, 5650-5655.

(S16) H. A. Krebs, *Biochem. J.* **1953**, *54*, 82–86.

(S17) E. Harder, W. Damm, J. Maple, C. Wu, M. Reboul, J. Y. Xiang, L. Wang, D. Lupyan, M. K. Dahlgren, J. L. Knight, J. W. Kaus, D. S. Cerutti, G. Krilov, W. L. Jorgensen, R. Abel, R. A. Friesner, *J. Chem. Theory Comput.* **2016**, *12*, 281–296.

(S18) Schrödinger Release 2019-4: MacroModel, Schrödinger, LLC, New York, NY (2019).

(S19) Gaussian 16, Revision C.01, M. J. Frisch, G. W. Trucks, H. B. Schlegel, G. E. Scuseria, M. A. Robb, J. R. Cheeseman, G. Scalmani, V. Barone, G. A. Petersson, H. Nakatsuji, X. Li, M. Caricato, A. V. Marenich, J. Bloino, B. G. Janesko, R. Gomperts, B. Mennucci, H. P. Hratchian, J. V. Ortiz, A. F. Izmaylov, J. L. Sonnenberg, D. Williams-Young, F. Ding, F. Lipparini, F. Egidi, J. Goings, B. Peng, A. Petrone, T. Henderson, D. Ranasinghe, V. G. Zakrzewski, J. Gao, N. Rega, G. Zheng, W. Liang, M. Hada, M. Ehara, K. Toyota, R. Fukuda, J. Hasegawa, M. Ishida, T. Nakajima, Y. Honda, O. Kitao, H. Nakai, T. Vreven, K. Throssell, J. A. Montgomery, Jr., J. E. Peralta, F. Ogliaro, M. J. Bearpark, J. J. Heyd, E. N. Brothers, K. N. Kudin, V. N. Staroverov, T. A. Keith, R. Kobayashi, J. Normand, K. Raghavachari, A. P. Rendell, J. C. Burant, S. S. Iyengar, J. Tomasi, M. Cossi, J. M. Millam, M. Klene, C. Adamo, R. Cammi, J. W. Ochterski, R. L. Martin, K. Morokuma, O. Farkas, J. B. Foresman, and D. J. Fox, Gaussian, Inc., Wallingford CT, 2016.

(S20) A. V. Marenich, C. J. Cramer, D. G. Truhlar, *J. Phys. Chem. B* **2009**, *113*, 6378–6396.

(S21) H. S. Yu, X. He, S. L. Li, D. G. Truhlar, *Chem. Sci.* **2016**, *7*, 5032-5051.

(S22) F. Weigend, R. Ahlrichs, *Phys. Chem. Chem. Phys.* **2005**, *7*, 3297-3305.

(S23) S. Grimme, *Chem. Eur. J.* **2012**, *18*, 9955–9964.

(S24) Visualisation was performed with CYLview20; Legault, C. Y., Université de Sherbrooke, 2020 (http://www.cylview.org).

(S25) B. Thapa, H. B. Schlegel, *J. Phys. Chem. A.* **2016**, *120*, 5726-5735.

(S26) C. P. Kelly, C. J. Cramer, D. G. Truhlar, *J. Phys. Chem. B.* **2006**, *110*, 16066-16081.

(S27) J. J. Fifen, Z. Dhaouadi, M. Nsangou, *J. Phys. Chem. A.* **2014**, *118*, 11090-11097.

(S28) S. J. Angyal, W. K. Warburton, *J. Chem. Soc.* **1951**, 2492-2494.

(S29) G. Schwarzenbach, K. Lutz, *Helv. Chim. Acta* **1940**, *23*, 1162-1190.

(S30) A. Albert, J. A. Mills, R. Royer, *J. Chem. Soc.* **1947**, 1452-1455.

(S31) J. B. Culbertson, *J. Am. Chem. Soc.* **1951**, *73*, 4818-4823.

(S32) E. H. Cordes, W. P. Jencks, *J. Am. Chem. Soc.* **1963**, *85*, 18, 2843–2848.

(S33) R. H. Hartigan, J. B. Cloke, *J. Am. Chem. Soc.* **1945**, *67*, 709-715.

(S34) J. B. Conant, P. D. Bartlett, *J. Am. Chem. Soc.* **1932**, *54*, 2881.

(S35) N. F. Hall, *J. Am. Chem. Soc.* **1930**, *52*, 5115-5128.

(S36) K. Koehler, W. Sandstrom, E. H. Cordes, *J. Am. Chem. Soc.* **1964**, *86*, 2413-2419

(S37) R. More O'Ferrall, D. O'Brien, *J. Phys. Org. Chem.* **2004**, *17*, 631-640.

(S38) N. Mehio, M. A. Lashely, J. W. Nugent, L. Tucker, B. Correia, C.-L. Do-Thanh, S. Dai, R. D. Hancock, V. S. Bryantsev, *J. Phys. Chem. B.* **2015**, *119*, 3567-3576.
